# The Discovery and Structure‐Activity Evaluation of (+)‐Floyocidin B and Synthetic Analogs

**DOI:** 10.1002/cmdc.202100644

**Published:** 2021-11-17

**Authors:** Yolanda Kleiner, Christoph Pöverlein, Jannike Klädtke, Michael Kurz, Henrik F. König, Jonathan Becker, Sanja Mihajlovic, Florian Zubeil, Michael Marner, Andreas Vilcinskas, Till F. Schäberle, Peter Hammann, Sören M. M. Schuler, Armin Bauer

**Affiliations:** ^1^ Branch for Bioresources Fraunhofer Institute for Molecular Biology and Applied Ecology (IME) (Germany) Ohlebergsweg 12 35392 Giessen Germany; ^2^ Sanofi-Aventis Deutschland GmbH Industriepark Höchst 65926 Frankfurt am Main Germany; ^3^ Biotest AG Landsteinerstraße 5 63303 Dreieich Germany; ^4^ Institute of Organic Chemistry Institute of Inorganic and Analytical Chemistry Justus Liebig University Heinrich-Buff-Ring 17 35392 Giessen Germany; ^5^ Bruker Daltonik GmbH Fahrenheitstraße 4 28359 Bremen Germany; ^6^ Institute for Insect Biotechnology Justus Liebig University Heinrich-Buff-Ring 26–32 35392 Giessen Germany; ^7^ Infectious Diseases – Natural Product Research Evotec International GmbH Marie-Curie-Straße 7 37079 Goettingen Germany

**Keywords:** Natural products, Mycobacterium tuberculosis, total synthesis, structure-activity relationship, biological profiling

## Abstract

Tuberculosis represents one of the ten most common courses of death worldwide and the emergence of multidrug‐resistant *M. tuberculosis* makes the discovery of novel anti‐tuberculosis active structures an urgent priority. Here, we show that (+)‐floyocidin B representing the first example of a novel dihydroisoquinoline class of fungus‐derived natural products, displays promising antitubercular hit properties. (+)‐Floyocidin B was identified by activity‐guided extract screening and its structure was unambiguously determined by total synthesis. The absolute configuration was deduced from a key synthesis intermediate by single crystal X‐ray diffraction analysis. A hit series was generated by the isolation of further natural congeners and the synthesis of analogs of (+)‐floyocidin B. Extensive biological and physicochemical profiling of this series revealed first structure‐activity relationships and set the basis for further optimization and development of this novel antitubercular scaffold.

## Graphical abstract

**Anti‐TB hit series**: Our search for new antitubercular natural products resulted in the discovery of the novel epoxyquinone (+)‐floyocidin B, which structure was unambiguously determined by total synthesis. Biological and physicochemical profiling of (+)‐floyocidin B and synthetic analogs revealed first structure‐activity relationships and set the basis for further optimization and development of this novel antitubercular scaffold.

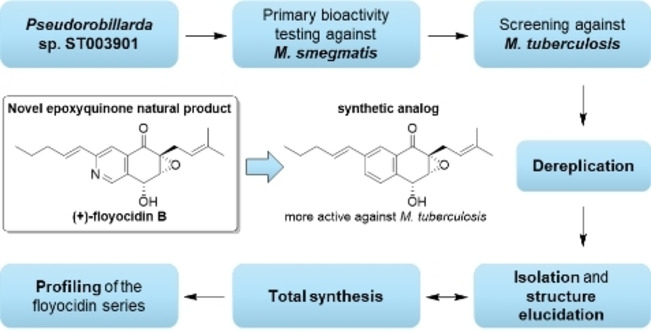

## Introduction

Tuberculosis (TB) remains one of the top causes of mortality associated with infectious diseases. Despite some progress in decreasing the global incidence between 2015 and 2019 by 9 %, a key milestone of the End TB Strategy (20 % reduction between 2015 and 2020) was not met. To date, an enormous health burden with 1.4 million deaths caused by TB and 10 million new cases in 2019 persists. Compliance problems issuing from the long and complex standard treatment schedules for drug‐sensitive TB infections (a combination therapy with 4 drugs over 6 months or longer) have led to the emergence of rifampicin and multi‐drug resistant TB (RR‐TB/MDR‐TB).^[1]^

Therefore, the continuous search for new drugs to establish improved therapy options, either in combination with existing or newly designed schedules, is still urgently needed. Ideally, a new drug should lead to a simpler and shorter treatment and have the potential to overcome resistant TB by acting on a novel target.[^2^, ^3^] While bacterial‐derived drugs, such as streptomycin and rifampicin, have been successfully introduced into the treatment of TB, natural products (NPs) of fungal origin have been less explored and therefore represent a promising starting point for the discovery of structurally novel lead structures acting by new antimycobacterial modes of action.

**Figure 1:**
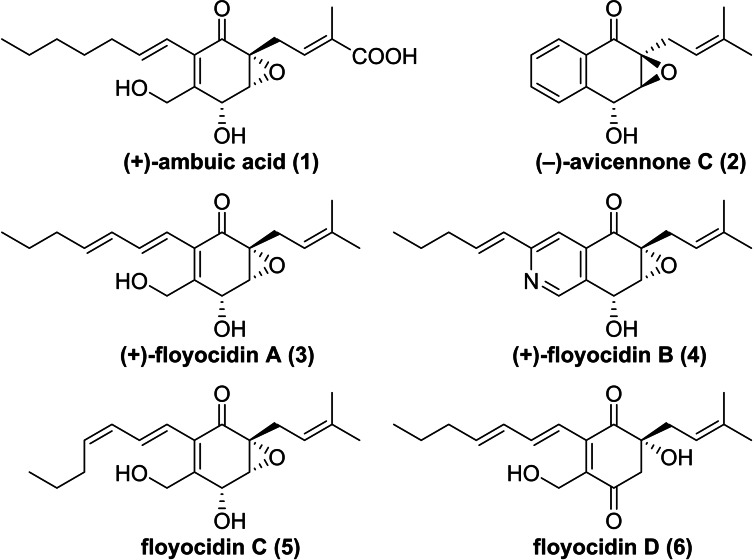
Structures of isolated floyocidins A−D (**3**–**6**) and structurally related NPs (+)‐ambuic acid (**1**) and (−)‐avicennone C (**2**).

Thus, in the context of a public‐private partnership (PPP) between Sanofi and Fraunhofer we have set up a bioactivity‐guided screening campaign to identify new compounds with antibacterial activity in a collection of microbial extracts prepared from the Fraunhofer‐Sanofi strain collection.^[4]^ Utilizing the established microfractionation screening process, fractions of the crude extract of the fungus *Pseudorobillarda* sp. ST003901 showed activities against the surrogate strain *M. smegmatis* (Msm) and the target pathogen *M. tuberculosis* (Mtb). Based on dereplication results, which indicated that the activity was due to the presence of potentially new compounds, a first fermentation campaign was initiated to obtain sufficient amounts of crude extract to isolate and identify the NPs responsible for the activities. Thereby, the two unknown epoxyquinones named (+)‐floyocidin A (**3**) and B (**4**) were isolated and their structures elucidated (Figure 1).

## Results and Discussion

The relative stereochemistry of **3** was established by comparison with the structurally related NP (+)‐ambuic acid (**1**).^[5]^ Proton and carbon chemical shifts as well as homonuclear coupling constants of the common epoxyquinone moiety are almost identical. In case of **4** the coupling constant between H1a and H2 (see Table 1) does not allow an unambiguous differentiation between the *cis*‐ and *trans*‐isomer. A direct comparison with the known NP (−)‐avicennone C (**2**) and its diastereomer^[6]^ is hampered by the different aromatic system. The dihydroisoquinolinone scaffold of **4** displays an unprecedented structural motif within the epoxyquinone NP family.[^7^, ^8^] Isolated **3** and **4** showed both weak activity against Msm with minimum inhibitory concentration (MIC) of 32–16 μg/mL and additionally (+)‐floyocidin B (**4**) exhibits to be active against Mtb (9.4 μg/mL). This Mtb activity qualified **4** as a starting point for an active to hit evaluation program, which includes investigations on structure‐activity relationships (SAR) and profiling with focus on parameters such as activity, pharmacokinetic properties, and toxicity.[^1^, ^2^]

**Table 1:** Comparison of NMR data and optical rotation of isolated and synthesized compounds **4** and **27**.

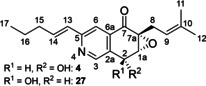						
	Floyocidin‐B (isolated NP)		4 (synthetic)		27 (synthetic)	
Position	^1^H‐NMR	^13^C‐NMR	^1^H‐NMR	^13^C‐NMR	^1^H‐NMR	^13^C‐NMR
	δ_H_ (500 MHz)^[a]^	δ_C_ (125 MHz)^[a]^	δ_H_ (600 MHz)^[a]^	δ_C_ (150 MHz)^[a]^	δ_H_ (500 MHz)^[a]^	δ_C_ (125 MHz)^[a]^
1a	3.82, d (1.9)	61.1	3.83, d (1.9)	61.1	3.82, d (2.3)	61.9
2	5.17, s, br	65.3	5.18, s, br	65.3	5.21, d (2.3)	64.9
2a		133.8		133.8		133.6
3	8.74, s	150.0	8.75, s	149.9	8.61, s	152.5
5		157.5		157.5		158.5
6	7.67, s	117.1	7.68, s	117.1	7.72, s	117.5
6a		137.7		137.7		138.2
7		195.4		195.4		195.5
7a		63.1		63.1		62.1
8	2.81, dd (15.2, 8.0)	27.2	2.82, dd (15.2, 8.0)	27.2	2.84, dd (15.2, 8.0)	27.5
	2.64, dd (15.2, 6.9)		2.65, dd (15.2, 6.9)		2.63, dd (15.2, 6.9)	
9	5.14, m	117.8	5.14, m	117.8	5.15, m	117.8
10		137.0		137.0		137.0
11	1.69, s	18.1	1.70, s	18.1	1.69, s	18.1
12	1.73, s	26.0	1.74, s	26.0	1.73, s	26.0
13	6.55, dt (15.8, 1.5)	130.1	6.56, dt (15.8, 1.5)	130.0	6.56, dt (15.8, 1.5)	130.1
14	6.78, dt (15.8, 7.1)	138.5	6.79, dt (15.8, 7.1)	138.5	6.82, dt (15.8, 7.1)	139.0
15	2.27, m	36.0	2.27, m	36.0	2.27, m	36.0
16	1.55, m	23.2	1.56, m	23.2	1.55, m	23.2
17	0.98, t (7.4)	14.1	0.99, t (7.4)	14.1	0.98, t (7.4)	14.1
7‐OH^[b]^	–	–		–		
specific rotation	${\left[\alpha \right]}_{D}^{24}$ = +160.0 (c 0.03; CHCl_3_)		${\left[\alpha \right]}_{D}^{24}$ = +159.3 (c 0.27; CHCl_3_)		${\left[\alpha \right]}_{D}^{22}$ = +179.3 (c 0.33; CHCl_3_)	
specific rotation of *ent*‐series	–		${\left[\alpha \right]}_{D}^{25}$ =−206.0 (c 0.22; CHCl_3_)		${\left[\alpha \right]}_{D}^{21}$ =−160.3 (c 1.20; CHCl_3_)	

[a] MeOD (δ in ppm, multiplicity, J in Hz). [b] OH group not detectible in MeOD.

To gain access to additional quantities of **4** for initial profiling, a refermentation in liquid culture (8 L) was performed. Besides a significant amount of **3** (55 mg) only traces of **4** (0.8 mg) were obtained as an inseparable mixture of **3**, **4**, and the new compound floyocidin C (**5**), which structure was elucidated as a *Z* double bond isomer of **3**. Additionally, a new derivative named floyocidin D (**6**) was isolated (Figure 1).^[9]^ Due to the structural similarity of **3**, **4**, and **5**, the same stereochemistry is assumed and fermentation kinetic studies indicated **3** as a biosynthetic precursor of **4**. It can be hypothesized that these NPs are biosynthesized by a polyketide synthase (PKS).[^7^, ^10^] In spite of efforts for fermentation optimization, e. g. by variation of media composition, pH value or concentration of oxygen, only low production titers of **4** were observed. Therefore, the focus was switched to total synthesis for structure elucidation and material supply.

**Scheme 1:**
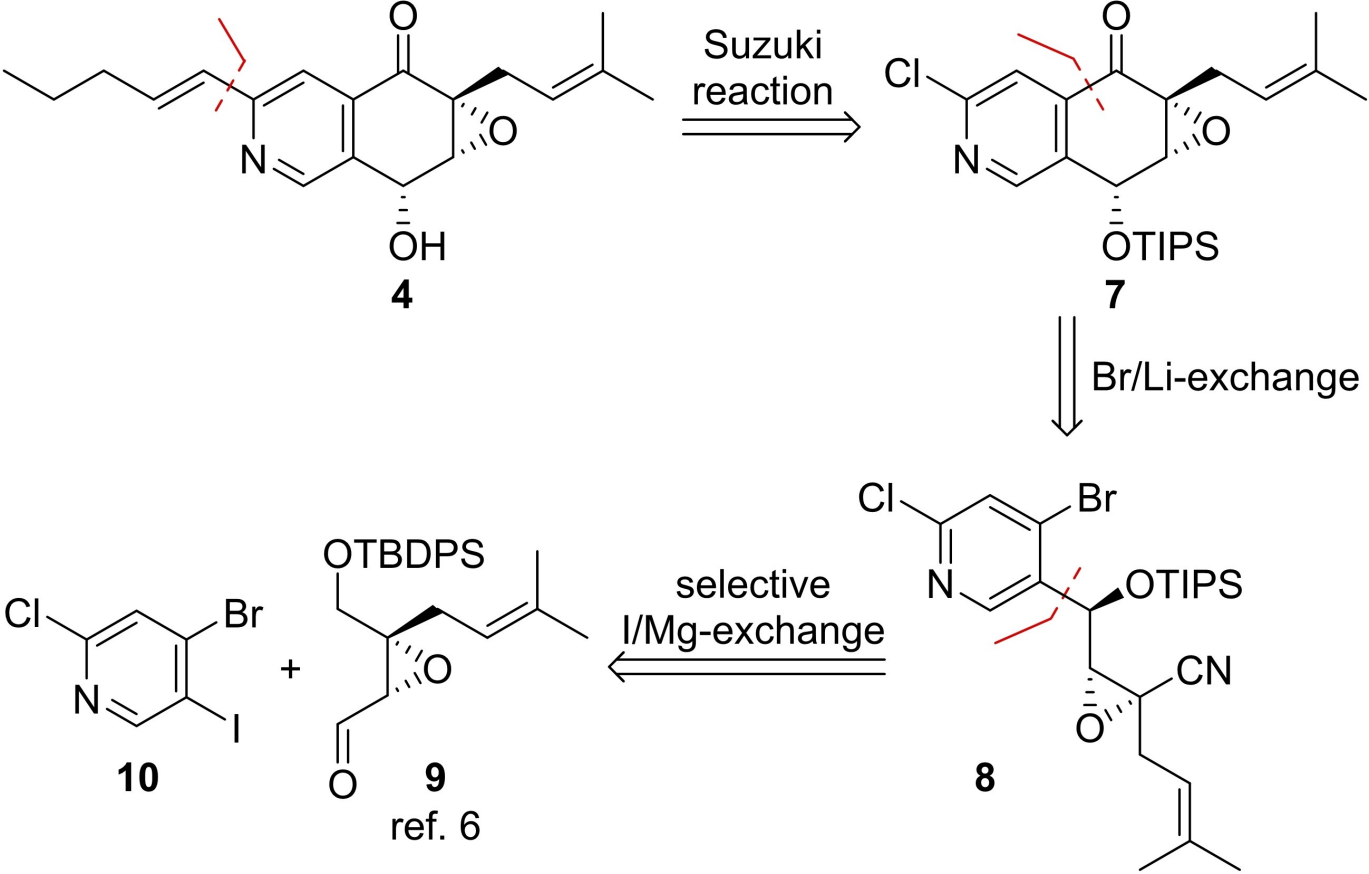
Retrosynthetic analysis of floyocidin B (**4**).

In our retrosynthetic analysis we identified the attachment of the pentenyl side chain *via* a Suzuki reaction as a suitable late stage functionalization of chloropyridine intermediate **7**. This reaction should be fully compatible with all functionalities and should allow for flexible decoration of the scaffold in a potential hit optimization program. The synthesis of **7** was developed in parallel to the synthesis of all four stereoisomers of (−)‐avicennone C (**2**) which served as a simplified model system^[6]^ for route scouting. Thus, ring closure was envisaged *via* selective bromine‐lithium exchange of nitrile **8**, which should be accessible by selective iodine‐magnesium exchange on trihalogenated pyridine **10** and reaction with epoxy aldehyde **9** followed by protecting group manipulations and oxidation to the nitrile (Scheme 1). With this synthetic strategy, all four stereoisomers of (+)‐floyocidin B (**4**) should be synthesized as references to elucidate the absolute stereochemistry and to support SAR investigations.

**Scheme 2:**
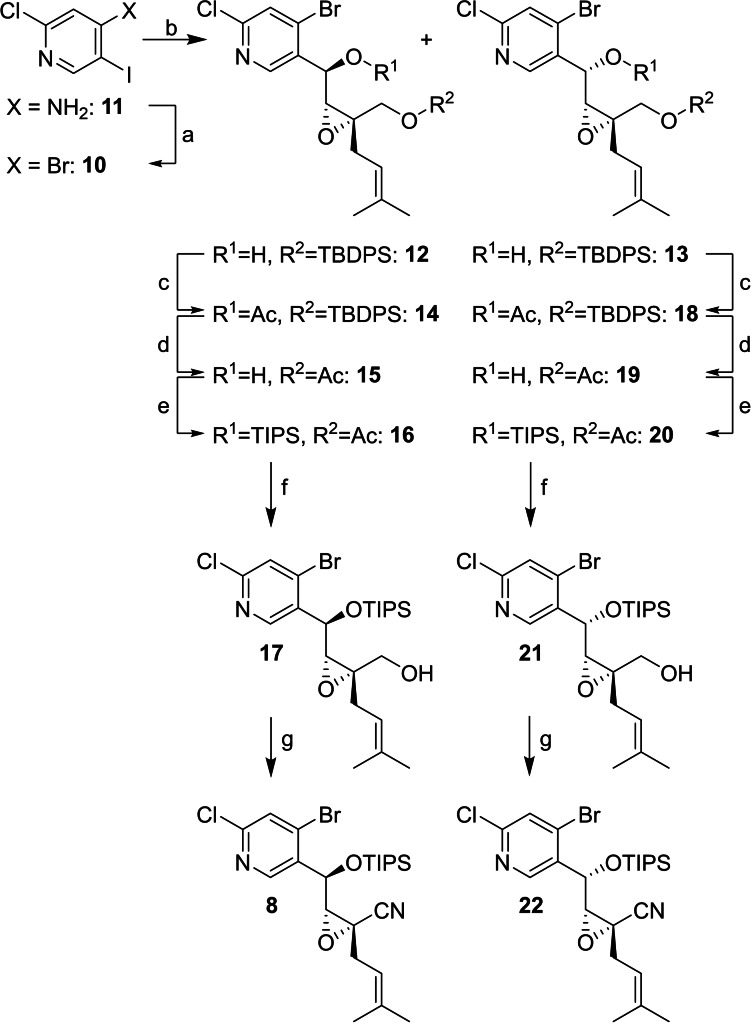
Synthesis of cyclization precursors **8** and **22**: (a) *t*‐BuONO, CuBr_2_, MeCN, 0 °C to rt, 97 %; (b) *i*‐PrMgCl, THF, −40 °C, 45 min; **9**, −40 °C, 1.5 h; 49 % for **12**, 42 % for **13**; (c) Ac_2_O, pyridine, DMAP (0.1 eq.), CH_2_Cl_2_; 97 % for **14**, 98 % for **18**; (d) TBAF, HOAc, THF; 86 % for **15**, 90 % for **19**; (e) TIPSOTf, 2,6‐lutidine, CH_2_Cl_2_; (f) LiOH, THF/H_2_O 5 : 1; 55 % over two steps for **17**, 71 % over two steps for **21**; (g) TEMPO (0.2 eq.), NH_4_OAc, BAIB, MeCN/H_2_O 9 : 1; 70 % for **8**, 60 % for **22**.

In spite of its structural simplicity, no references for the synthesis of 4‐bromo‐2‐chloro‐5‐iodopyridine (**10**) are described in literature. After investigation of different strategies to synthesize this trihalogenated pyridine, we finally identified a convenient access to **10** *via* a modification of the Sandmeyer reaction under anhydrous conditions,^[11]^ which converted commercially available amine **11** into **10** in 5 g scale and excellent yields. As expected iodine‐magnesium exchange with *i*‐PrMgCl occurred with full chemoselectivity and trapping of the Grignard reagent with aldehyde **9** yielded the two diastereomeric alcohols **12** and **13** in good yields. **12** and **13** were acetate protected and the TBDPS group was cleaved by TBAF, which was accompanied by a spontaneous acetate migration to the primary position. Consecutive TIPS protection and saponification of the acetate resulted in primary alcohols **17** and **21**, which were oxidized to nitriles **8** and **22** (Scheme 2). The reaction sequence was performed in close analogy to the total synthesis of (−)‐avicennone C^[6]^ (**2**) with two significant differences: (1) a direct conversion of **12** and **13** to **17** and **21** *via* TIPS protection and selective TBDPS deprotection (not shown) was hampered by very low yields (<20 % over two steps) due to double deprotection of both silyl protecting groups and (2) the acetate migration in the conversion of **14** and **18** to **15** and **19** occurred without addition of a base.

**Figure 2:**
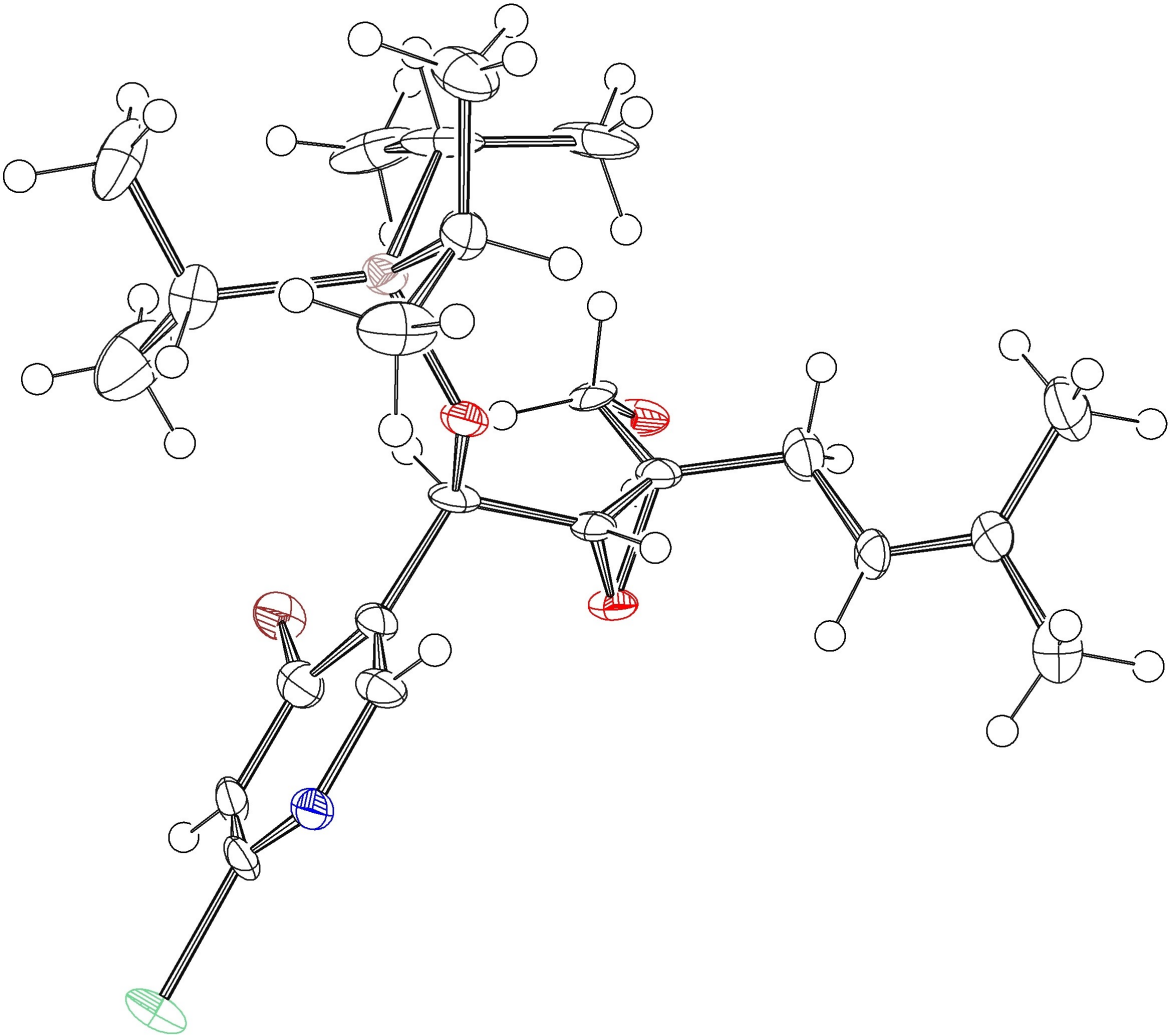
Thermal ellipsoid plot of the molecular structure of **17**. Thermal ellipsoid probability set to 50 %, only mostly occupied disorder part shown.

The absolute stereochemistry of alcohol intermediate **17** was unambiguously assigned by single X‐ray crystal diffraction and the stereochemistry of the other stereoisomers was deduced thereof (Figure 2).

**Scheme 3:**
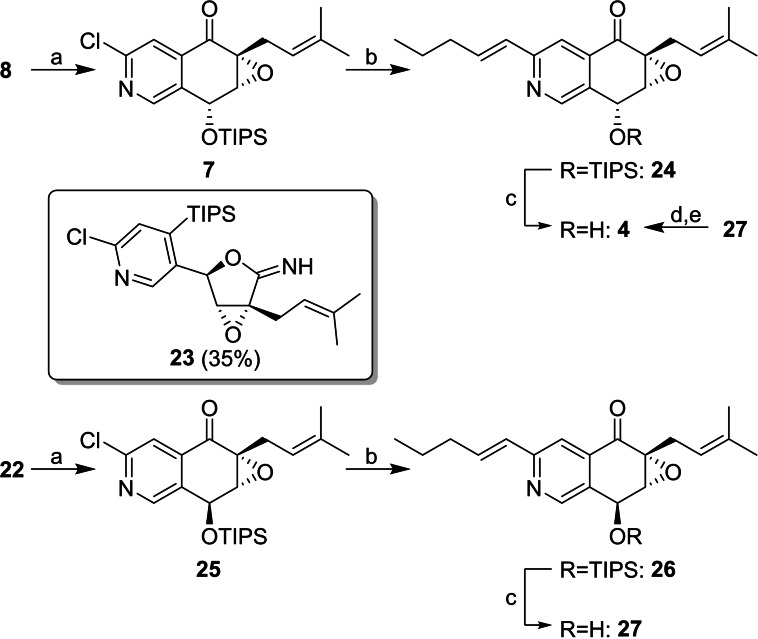
Finalization of the total synthesis of floyocidin B (**4**) and **27**: (a) *n‐*BuLi (1.0 eq.), THF, −100 °C, 20 min; 5 min without cooling; 26 % for **7**, 81 % for **25**; (b) APhos Pd G3 (0.1 eq.), Cs_2_CO_3_, *trans*‐1‐penten‐1‐ylboronic acid pinacol ester, 1,4‐dioxane/H_2_O 8 : 1, 100 °C; 51 % for **24**, 84 % for **26**; (c) TBAF, HOAc, THF; 60 % for **4**, 93 % *ee*; 65 % for **27**, 93 % *ee*; (d) PPh_3_, HOAc, DIAD, THF; 84 %; (e) LiOH, THF/H_2_O 5 : 1; 51 %.

Selective bromine‐lithium exchange at low temperature and intramolecular reaction with the nitrile resulted in **7** and **25**, respectively, after slightly acidic work‐up. While **25** was obtained in good yields and as a single product, conversion of **8** resulted in the formation of desired compound **7** and a second product, which was identified as cyclic imidate **23**. This tendency was also observed for the synthesis of ***ent*** **‐7** and ***ent*** **‐25**. Noteworthy, in the course of the synthesis of avicennone C this cyclization proceeded smoothly for both diastereomers.^[6]^ Carefully monitored Suzuki reaction with *trans*‐1‐penten‐1‐ylboronic acid pinacol ester^[12]^ and final TIPS deprotection yielded all four stereoisomers of floyocidin B. To compensate the loss of yield in the conversion of **8** to **7**, the previously developed recycling strategy *via* Mitsunobu inversion and saponification of the acetate was successfully applied to the conversion of **27** to **4** (Scheme 3).^[6]^

Comparison of NMR spectra, specific rotations (Table 1), and chiral HPLC (see Supporting Information) elucidated the 1a*R*,2*R*,7a*R*‐isomer (**4**) as the correct structure of NP (+)‐floyocidin B.

**Scheme 4:**
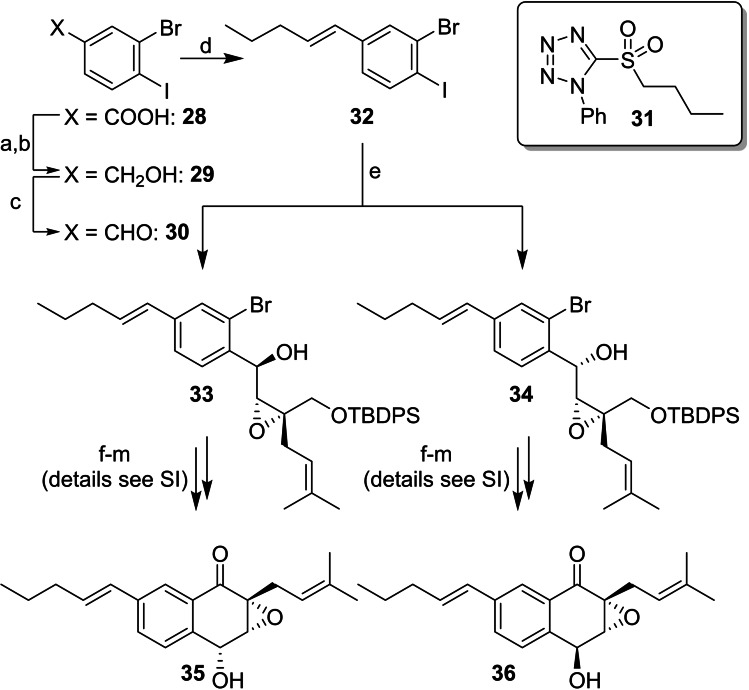
Total synthesis of C‐analogs **35**, ***ent*** **‐35**, **36**, and ***ent*** **‐36** of floyocidin‐B, exemplarily shown for one diastereomeric series: (a) (COCl)_2_, DMF, CH_2_Cl_2_; (b) LiBH_4_, THF; 93 % over two steps; (c) (COCl)_2_, DMSO, CH_2_Cl_2_, −78 °C, 15 min; **29**, −78 °C, 90 min; DIPEA, −78 °C to 0 °C, 30 min; quant. used as a crude; (d) **31**, KHMDS, THF, −55 °C, 1 h 10 min; **32**, −55 °C, 1 h; 75 % (*E*/*Z* 94 : 6); (e) *i*‐PrMgCl, THF, −40 °C, 45 min; **9**, −40 °C, 1.5 h; 42 % for **33**, 42 % for **34**; (f) Ac_2_O, pyridine, DMAP (0.1 eq.), CH_2_Cl_2_; 97 %/99 %; (g) TBAF, HOAc, THF; (h) DBU, CH_2_Cl_2_; 87 %/79 % over two steps; (i) TIPSOTf, 2,6‐lutidine, CH_2_Cl_2_; (j) LiOH, THF/H_2_O 5 : 1; 46 %/63 % over two steps; (k) TEMPO (0.2 eq.), NH_4_OAc, BAIB, MeCN/H_2_O 9 : 1; 48 %/41 %; (l) *n‐*BuLi, THF, −100 °C, 20 min; 5 min without cooling; (m) TBAF, HOAc, THF; 58 % over two steps for **35**, 31 % over two steps for **36**.

To address the side product formation during Suzuki reaction of compounds **7** and **25**, an alternative strategy based on an *E*‐selective formation of the pentenyl side chain at an early stage of the synthesis was developed. Since our first generation synthesis gave access to sufficient amounts of all four stereoisomers of (+)‐floyocidin B (**4**), formal avicennone C‐floyocidin B hybrids **35** and **36** were chosen as attractive targets for both route scouting and investigation of the SAR. These hybrids exhibit punctual mutations of **4** and **27** with increased lipophilicity and were expected to show higher activity on Mtb due to improved cell permeability. Central intermediate **32** was obtained starting from carboxylic acid **28** in four steps. **28** was converted to aldehyde **30** and served as starting material for a Julia‐Kocieński olefination with a high *E*‐selectivity.^[13]^ Selective iodine‐magnesium exchange and reaction with aldehyde **9**, protecting group manipulations, oxidation to the nitrile, cyclisation *via* bromine‐lithium exchange, and final TIPS deprotection were performed in close analogy to the synthesis of floyocidin B (Scheme 4, for details see Supporting Information).

New NPs are often discovered as singletons or a set of compounds with a few congeners. In many cases there are only limited bioactivity data available, which are mainly related to disease‐relevant assays such as activity against a panel of cancer cell lines or pathogenic bacteria. However, from a medicinal chemistry perspective, assessment of a screening hit does not only require data with regard to potency at a target and off‐target effects, but also knowledge of key parameters concerning physicochemical and pharmacokinetic properties and potential liabilities thereof.^[14]^ Ideally, SAR with these parameters that can be established on a small series of structurally related analogs, should allow for an efficient multi‐objective molecular optimization toward a valuable lead structure and clinical development candidate.^[15]^

To this end, the antibacterial activities of NP (+)‐floyocidin A (**3**) from isolation and in total 12 synthesized compounds (all four stereoisomers of (+)‐floyocidin B and (−)‐avicennone C^[6]^ (**2**) as well as hybrids **35**, ***ent*** **‐35**, **36**, and ***ent*** **‐36**) were tested *in vitro* against a panel of seven indicator strains (*E. coli*, *P. aeruginosa*, *M. catarrhalis*, *S. aureus*, *C. albicans*, *M. smegmatis*, *M. tuberculosis*) (Table 2 and see Supporting Information). While none of the tested substrates inhibited the growth of *E. coli*, *P. aeruginosa* or *C. albicans*, only (+)‐floyocidin A (**3**) showed a weak activity against *S. aureus* (64 μg/mL). The stereoisomers of (+)‐floyocidin B (**4**), avicennone C as well as the hybrids inhibited the growth of the surrogate strain *M. smegmatis* with MICs down to 4 μg/mL. Both, isolated and synthetic (+)‐floyocidin B were active against *M. tuberculosis* H37Rv with a MIC of 9.4 μg/mL. The *C*‐analogs **35**, ***ent*** **‐35**, **36**, and ***ent*** **‐36** displayed activity against *M. tuberculosis* H37Rv in the same range, pointing toward a positive correlation between lipophilicity and activity. Furthermore, the data indicate that the presence of a pentenyl side chain and the *syn*‐configuration of the epoxide and hydroxyl group seem to be important for the antibacterial activity. Unfortunately, cytotoxic effects (TC_50_) in both THP‐1 and HepG2 were observed in the same order of magnitude as the antitubercular activity, but the safety index may be improved with additional derivatives (see for example **36**, which is three times less cytotoxic than **4** but still displays the same activity against *M. tuberculosis*). Undoubtedly, identification of the mode of action of both, the antibacterial and off‐target effects should be of high priority in further investigation.

**Table 2:** Bioactivity data of NPs and SAR‐compounds against *M. smegmatis* ATCC607 (Msm) and *M. tuberculosis* H37Rv (Mtb).

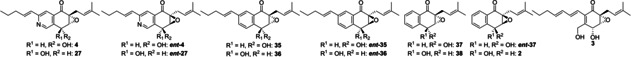													
	MIC [μg/mL]												
	4	27	*ent*‐4	*ent*‐27	35	36	*ent*‐35	*ent*‐36	37	38	*ent*‐37	2	3
Msm	64	16	32	16	32	4	32	8–4	128–64	NA	128–64	NA	32
Mtb	9.4	NA	NA	NA	9.4	9.4	9.4	9.4	NA	NA	NA	NA	NA

[a] NA=not active.

The floyocidins and their analogs displayed a relatively high lipophilicity (log *D* from 2.8 to 4.7), which has turned out to be favorable in other compound optimization programs for a high compound exposure in tissues relevant to tuberculosis (lung, spleen).[^14^, ^16^] The observed lipophilicity range, together with molecular weights around 300 Da and a low number of hydrogen bond donors and acceptors should translate into a good oral bioavailability (a requisite for TB therapy schemes), which is further underlined by the prediction of high human oral resorption through excellent permeability (P_app_) values larger than 40 ⋅ 10^−7^ cm/s in the Caco‐2 intestinal epithelial barrier model^[17]^ (Table 3). Since no increase of the apical to basolateral transport rate in the presence of elacridar was observed in the Caco‐2 assay, the permeability of the compounds is not affected by the PgP efflux transporter.^[18]^ Metabolism was studied in liver microsomes.^[14]^ While the compounds showed moderate to low clearance in human microsomes corresponding to an acceptable stability, rapid metabolization was observed in mice microsomes. This finding may hamper further investigations in mice models of tuberculosis infection and should be addressed early on in compound optimization programs after UPLC‐HRMS and/or NMR‐based identification of the metabolic soft spots.

**Table 3:** Profiling data of NPs and SAR‐compounds.

	Log *D* ^[d]^	Microsomal stability^[d]^		Caco‐2 permeability^[d]^		Cytotoxicity^[d]^	
	pH 7.4	CL_int_ [μL/min/mg protein] (t_1/2_ [min])		P_app_ [10^−7^ cm s^−1^] (recovery [%])		TC_50_ [μM]	
		Human	Mouse	A2B	+Elacridar	THP1	HepG2
3	4.11	[c]	[c]	[b]	[c]	[c]	[c]
4	4.01	72.7 (19.1)	349 (3.97)	41.1 (10.1)	56.2 (14.2)	5.84	4.5
27	4.14	95.8 (14.5)	1500 (0.924)	117 (42.1)	87.7 (38.5)	10.6	10.7
*ent*‐4	3.97	26.9 (51.6)	271 (5.12)	73.5 (23.4)	46.7 (28.7)	8.78	5.97
*ent*‐27	4.16	44.3 (31.3)	764 (1.81)	40.5 (8.69)	64.4 (15.3)	11.5	10.4
35	4.7	45.5 (30.5)	1040 (1.33)	135 (76.0)	181 (29.6)	12.9	9.39
36	4.73	39.7 (34.9)	259 (5.36)	[b] (16.1)	[b] (19.9)	15.1	16.1
*ent*‐35	4.72	40.5 (34.3)	561 (2.47)	162 (37.2)	[b] (46.1)	12.9	14.7
*ent*‐36	4.74	29.9 (46.3)	721 (1.92)	8.55 (14.3)	12.4 (12.0)	18.2	11.3
37	2.82	54.6 (25.4)	[a]	326 (74.8)	279 (80.9)	27.2	30
38	2.84	76.0 (18.2)	1070 (1.29)	620 (127)	331 (132)	30	9.49
*ent*‐37	2.82	73.5 (18.9)	370 (3.75)	360 (81.3)	300 (74.6)	25.2	30
2	2.86	150 (9.26)	[a]	407 (86.7)	393 (83.4)	30	>30

[a] Test compound not detectable by 5 minutes. Unable to determine CL_int_. [b] No data obtained. [c] Not determined. [d] For measurement details and standard deviations of biological data see Supporting Information.

## Conclusion

In conclusion, the floyocidins have been characterized as a novel NP scaffold for the potential lead discovery and optimization of new anti‐tuberculosis drugs. Besides isolation, a flexible and scalable synthetic entry into the floyocidin B stereoisomers in combination with single crystal X‐ray diffraction analysis was key for structure elucidation. The delivery of sufficient amounts of both, the NPs as well as a series of synthetic analogs that allowed for extensive biological profiling, provided insight into first SAR not only regarding antibacterial and cytotoxic activities but also related to pharmacokinetic properties. In this context, metabolic lability and off‐target effects were identified as key liabilities to be addressed in forthcoming multi‐objective compound optimization programs toward clinical development candidates. Further studies of SAR are required to increase the therapeutic window but in particular combination therapy as practiced in the current standard of care may give an opportunity for the introduction of a floyocidin‐based drug to achieve synergistic effects, overcome resistance and achieve a more favorable therapeutic window.

## Experimental Section

**General methods**: All chemicals and solvents/anhydrous solvents were commercially supplied and used without further purification. For heating of reaction mixtures, aluminum flask carriers in different sizes from IKA were used. Reactions were monitored using thin layer chromatography (TLC) or using one of the following LC‐MS systems: 1100 HPLC (Agilent) with DAD and ELSD equipped with MSD (Agilent) ESI quadrupole MS, 1100 HPLC (Agilent) with DAD equipped with Amazon (Bruker) ESI trap MS, 1290 UPLC (Agilent) with DAD or ELSD equipped with micrOTOF (Bruker) ESI TOF MS or 1290 UPLC (Agilent) with DAD and ELSD equipped with maXis II (Bruker) ESI TOF MS. TLC was performed on pre‐coated silica gel glass plates (Merck TLC Silica gel 60 F254) and compounds were detected under UV light (254 nm) and/or by staining with an aqueous solution of KMnO_4_ with K_2_CO_3_ and NaOH or an aqueous solution of phosphomolybdic acid, cerium(IV) sulfate, and H_2_SO_4_ followed by heating with a heat gun. Products were purified by flash column chromatography using silica gel 60 M (Macherey‐Nagel) or by using an automated flash column chromatography system (Biotage® SP4 with ISOLUTE® Flash SI II) equipped with ISOLUTE® Flash SI II columns of different sizes from Biotage, PF‐15SIHC flash columns of different sizes from Interchim or Götec GX flash columns of different sizes from Götec‐Labortechnik (eluents are given in parentheses). The HPLC purifications were performed with a semi‐preparative 1100 HPLC system from Agilent or a semi‐preparative 1200 HPLC system from Agilent using RP columns (flow rate: 3 mL/min; columns and eluents are given in parentheses). The product containing fractions were identified using LC–MS, pooled and concentrated *in vacuo* or freeze‐dried. NMR spectra were recorded on a Bruker AVANCE II WB spectrometer (400 MHz), an AVANCE III HD spectrometer (400 MHz), an AVANCE III spectrometer (500 MHz) equipped with a TCI CryoProbe or an AVANCE III HD spectrometer (600 MHz) with CDCl_3_ or CD_3_OD as solvent with chemical shifts (δ) quoted in parts per million (ppm) and referenced to the solvent signal (δ^1^H/^13^C: CDCl_3_ 7.26/77.16, CD_3_OD 3.31/49.00) or TMS (δ=0.00 ppm in CDCl_3_). Assignment was confirmed based on COSY, HSQC, HMBC, ROESY, and NOESY correlations. The absolute configuration of compound **17** was assigned by X‐ray diﬀraction analysis (for details regarding solvent and method for crystal growth see synthetic procedure). High resolution mass spectrometry was performed on the maXis II (Bruker) ESI TOF MS. Specific rotation was measured by a polarimeter (P 3000 series) from Krüss. Synthetic steps are described in detail for intermediates leading to the NP (+)‐floyocidin B (**4**) and the corresponding stereoisomers are listed afterward.

**Initial fermentation and isolation for structure elucidation**: Strain ST003901 from the Fraunhofer‐Sanofi strain collection^[4]^ was cultivated on solid agar in 10×300 mL Erlenmeyer flasks filled with 75 mL medium comprising 20 g/L malt extract, 2 g/L yeast extract, 10 g/L glucose, 0.5 g/L (NH_4_)_2_HPO_4_, H_2_O (pH=6.0 before autoclaving). After incubation for 11 days as standing cultures at 25 °C, solid agar from fermentation was homogenized in water and extracted with 2x volume of ethyl acetate by stirring overnight. Evaporation of the ethyl acetate layer yielded 157 mg of extract. Pre‐purification was performed by semi‐preparative HPLC (column: Synergy Fusion‐RP 80 Å, 4 μm, 250×10 mm from Phenomenex; Eluent A: H_2_O+0.1 % formic acid, Eluent B: MeCN+0.1 % formic acid, 50 % B at 0 min, 50 % B at 5 min, 90 % B at 25 min, and 100 % B at 26 min). Elution of target compounds **3** and **4** was monitored using LC‐MS and fractionation was performed. Fractions containing the target compounds where pooled and further purified by HPLC separation (column: Jupiter® 4 μm Proteo 90 Å, 250×10 mm from Phenomenex; isocratic with 65 % MeCN in H_2_O+0.1 % formic acid). Two separate fractions yielded (+)‐floyocidin A (**3**, 1.3 mg) and (+)‐floyocidin B (**4**, 0.6 mg) as colorless solids.

**8 L refermentation**: Inoculation of 80 x 300 mL Erlenmeyer flasks filled with 100 mL medium comprising 20 g/L malt extract, 2 g/L yeast extract, 10 g/L glucose, 0.5 g/L (NH_4_)_2_HPO_4_, H_2_O (pH=6.0 before autoclaving) was performed with 5 % (v/v) pre‐culture of strain ST003901. After 13 days incubation at 25 °C with shaking at 180 rpm, the first 4 L of the fermentation were harvested, the remaining culture after 17 days. Cells and culture filtrate were separated by centrifugation and filtration. The filtrates were extracted with ethyl acetate (8 L) and evaporation of the ethyl acetate layer yielded 646 mg of extract. Cells were freeze‐dried and extracted with MeOH (3×250 mL per 4 L fermentation volume). The MeOH extract was concentrated under reduced pressure and the crude extract (6.56 g) was subsequently fractionated by stepwise elution (0 %, 20 %, 40 %, 60 %, 80 %, and 100 % MeOH in H_2_O) over a column (4 x 30 cm) with Amberlite® XAD‐7/XAD‐16 N (1 : 1, 125 g/125 g). Fractions containing target compounds **3** and **4** (80 % and 100 % MeOH in H_2_O) were merged, dried under reduced pressure, and pre‐purified together with dried ethyl acetate extract by semi‐preparative HPLC (column: Synergy Fusion‐RP 80 Å, 4 μm, 250 x 10 mm from Phenomenex; Eluent A: H_2_O+0.1 % formic acid, Eluent B: MeCN+0.1 % formic acid, 55 % B at 0 min, 55 % B at 5 min, 65 % B at 25 min, and 100 % B at 26 min). Fractions containing the target compounds where pooled and further purified by HPLC (column: Nucleodur C18 Gravity SB, 3 μm, 250×10 mm; isocratic with 57 % MeCN in H_2_O+0.1 % formic acid). Besides further (+)‐floyocidin A (**3**) (55 mg), a mixture of floyocidin C (**5**)/floyocidin B (**4**)/ floyocidin A (**3**) (0.8 mg, 2.4 : 1.2 : 1) was obtained. During the purification of nearly pure fractions with floyocidin A by semi‐preparative HPLC (column: Nucleodur C18 Gravity SB, 3 μm, 250×10 mm; isocratic with 57 % MeCN in H_2_O+0.1 % formic acid), formation of a further signal was observed. The structure was elucidated by NMR analysis and the compound was named floyocidin D (**6**).^[19]^

**(+)‐Floyocidin A** ^[20]^ **/ (1*R*,5*R*,6*R*)‐3‐((1*E*,3*E*)‐Hepta‐1,3‐dien‐1‐yl)‐5‐hydroxy‐4‐(hydroxymethyl)‐1‐(3‐methylbut‐2‐en‐1‐yl)‐7‐oxabicyclo[4.1.0]hept‐3‐en‐2‐one (3)**: ^**1**^ **H‐NMR** (MeOD, 500 MHz): δ=6.48 (dd, 1H, *J*=15.7, 10.5 Hz, C_q_‐CH=C*H*), 6.24 (d, 1H, *J=*15.7 Hz, C_q_‐C*H*=CH), 6.13 (dd, 1H, *J*=15.2, 10.4 Hz, C_q_‐CH=CH‐C*H*), 5.80 (dt, 1H, *J*=15.2, 7.1 Hz, CH_2_‐CH_2_‐C*H*), 5.07 (m, 1H, Me_2_C=C*H*), 4.77 (s, br, 1H, C*H*‐OH), 4.48 (d, 1H, *J=*12.9 Hz, C*H*_2_‐OH), 4.41 (d, 1H, *J*=12.9 Hz, C*H*_2_‐OH), 3.67 (d, 1H, *J*=2.8 Hz, epoxy‐C*H*), 2.71 (dd, 1H, *J*=15.2, 7.9 Hz, Me_2_C=CH‐C*H*_2_), 2.48 (dd, 1H, *J*=15.2, 6.9 Hz, Me_2_C=CH‐C*H*_2_), 2.10 (m, 2H, CH_3_‐CH_2_‐C*H*_2_), 1.70 (s, 3H, *E*‐C*H*_3_), 1.65 (s, 3H, *Z*‐C*H*_3_), 1.44 (m, 2H, CH_3_‐C*H*_2_), 0.92 (t, 3H, *J*=7.4 Hz, C*H*_3_‐CH_2_); ^**1**^ **H‐NMR** (CDCl_3_, 400 MHz): δ=6.48 (dd, 1H, *J*=15.7, 10.5 Hz, C_q_‐CH=C*H*), 6.15 (d, 1H, *J*=15.7 Hz, C_q_‐C*H*=CH), 6.09 (dd, 1H, *J*=15.2, 10.5 Hz, C_q_‐CH=CH‐C*H*), 5.81 (dt, 1H, *J*=15.1, 7.0 Hz, CH_2_‐CH_2_‐C*H*), 5.02 (m, 1H, Me_2_C=C*H*), 4.84 (s, br, 1H, C*H*‐OH), 4.66–4.50 (m, 2H, *J*=12.9 Hz, C*H*_2_‐OH), 3.72 (d, 1H, *J*=2.8 Hz, epoxy‐C*H*), 2.78 (dd, 1H, *J*=15.4, 7.9 Hz, Me_2_C=CH‐C*H*_2_), 2.52 (dd, 1H, *J*=15.4, 6.7 Hz, Me_2_C=CH‐C*H*_2_), 2.08 (m, 2H, CH_3_‐CH_2_‐C*H*_2_), 1.70 (s, 3H, *E*‐C*H*_3_), 1.63 (s, 3H, *Z*‐C*H*_3_), 1.42 (m, 2H, CH_3_‐C*H*_2_), 0.90 (t, 3H, *J*=7.4 Hz, C*H*_3_‐CH_2_); ^**13**^ **C‐NMR** (MeOD, 125 MHz): δ=196.7 (*C*=O), 150.2 (OH‐CH_2_‐*C*_q_), 138.0 (C_q_‐CH=*C*H), 138.0 (CH_2_‐CH_2_‐*C*H), 136.5 (*C*_q_Me_2_), 132.3 (C_q_‐CH=CH‐*C*H), 131.8 (*C*_q_‐CH=CH), 122.9 (C_q_‐*C*H=CH), 118.2 (*C*H=CMe_2_), 66.3 (*C*H‐OH), 61.9 (epoxy*‐C*_q_), 60.5 (epoxy‐*C*H), 60.0 (*C*H_2_‐OH), 36.0 (CH_3_‐CH_2_‐*C*H_2_), 27.6 (Me_2_C=CH‐*C*H_2_), 26.0 (*E*‐*C*H_3_), 23.5 (CH_3_‐*C*H_2_), 18.0 (*Z*‐*C*H_3_), 14.0 (*C*H_3_‐CH_2_); ^**13**^ **C‐NMR** (CDCl_3_, 100 MHz): δ=194.6 (*C*=O), 146.7 (OH‐CH_2_‐*C*_q_), 138.3 (C_q_‐CH=*C*H), 137.8 (CH_2_‐CH_2_‐*C*H), 136.3 (*C*_q_Me_2_), 130.8 (C_q_‐CH=CH‐*C*H), 130.4 (*C*_q_‐CH=CH), 120.7 (C_q_‐*C*H=CH), 116.5 (*C*H=CMe_2_), 66.8 (*C*H‐OH), 61.5 (epoxy*‐C*_q_), 61.3 (epoxy‐*C*H), 58.7 (*C*H_2_‐OH), 35.0 (CH_3_‐CH_2_‐*C*H_2_), 26.6 (Me_2_C=CH‐*C*H_2_), 26.0 (*E*‐*C*H_3_), 22.4 (CH_3_‐*C*H_2_), 18.1 (*Z*‐*C*H_3_), 13.8 (*C*H_3_‐CH_2_); **HRMS (ESI)** m/z calcd. for C_19_H_27_O_4_: 319.1904 (M+H)^+^; found: 319.1905 (M+H)^+^; **R_f_** (*n*‐heptane/EA 1 : 1): 0.34; **Specific rotation** ${\left[\alpha \right]}_{D}^{24.3}$ =+174.5 (c=0.90; CHCl_3_).

**(+)‐Floyocidin B** ^[21]^ **/ (1a*R*,2*R*,7a*R*)‐2‐Hydroxy‐7 a‐(3‐methylbut‐2‐en‐1‐yl)‐5‐((*E*)‐pent‐1‐en‐1‐yl)‐1 a,7 a‐dihydrooxire‐no[2,3‐g]isoquinolin‐7(2H)‐one (4)**: ^**1**^ **H‐NMR** (MeOD, 500 MHz): δ=8.74 (s, 1H, N‐C*H*), 7.67 (s, 1H, pentenyl‐C_q_‐C*H*), 6.78 (dt, 1H, *J*=15.8, 7.1 Hz, CH_2_‐C*H*=CH), 6.55 (dt, 1H, *J*=15.8, 1.5 Hz, CH_2_‐CH=C*H*), 5.17 (s, br, 1H, C*H*‐OH), 5.14 (m, 1H, Me_2_C=C*H*), 3.82 (d, 1H, *J*=1.9 Hz, epoxy‐C*H)*, 2.81 (dd, 1H, *J*=15.2, 8.0 Hz, Me_2_C=CH‐C*H*_2_), 2.64 (dd, 1H, *J*=15.2, 6.9 Hz, Me_2_C=CH‐C*H*_2_), 2.27 (m, 2H, C*H*_2_‐CH=CH), 1.73 (s, 3H, *E*‐C*H*_3_), 1.69 (s, 3H, *Z*‐C*H*_3_), 1.55 (m, 2H, CH_3_‐C*H*_2_), 0.98 (t, 3H, *J*=7.4 Hz, C*H*_3_‐CH_2_); ^**13**^ **C‐NMR** (MeOD, 125 MHz): δ=195.4 (*C=*O), 157.5 (N‐*C*_q_), 150.0 (N‐*C*H), 138.5 (CH_2_‐*C*H=CH), 137.7 (Ar‐*C*_q_‐CO), 137.0 (*C*_q_Me_2_), 133.8 (N‐CH‐*C*_q_), 130.1 (CH_2_‐CH=*C*H), 117.8 (*C*H=CMe_2_), 117.1 (pentenyl‐C_q_ *‐C*H*)*, 65.3 (*C*H‐OH), 63.1 (epoxy‐*C*_q_), 61.1 (epoxy‐*C*H), 36.0 (*C*H_2_‐CH=CH), 27.2 (Me_2_C=CH‐*C*H_2_), 26.0 (*E*‐*C*H_3_), 23.2 (CH_3_‐*C*H_2_), 18.1 (*Z*‐*C*H_3_), 14.1 (*C*H_3_‐CH_2_); **HRMS (ESI)** m/z calcd. for C_19_H_24_NO_3_: 314.1751 (M+H)^+^; found: 314.1751 (M+H)^+^; **R_f_** (*n*‐heptane/ethyl acetate 1 : 1): 0.37; **Specific rotation** ${\left[\alpha \right]}_{D}^{24.4}$ =+160.0 (c=0.03; CHCl_3_).

**Floyocidin C** ^[22]^ **/ (1*R*,5*R*,6*R*)‐3‐((1*E*,3*Z*)‐Hepta‐1,3‐dien‐1‐yl)‐5‐hydroxy‐4‐(hydroxymethyl)‐1‐(3‐methylbut‐2‐en‐1‐yl)‐7‐oxabicyclo[4.1.0]hept‐3‐en‐2‐one (5)**: ^**1**^ **H‐NMR** (MeOD, 500 MHz): δ=6.86 (dd, 1H, *J*=15.7, 11.2 Hz, C_q_‐CH=C*H*), 6.32 (d, 1H, *J=*15.5 Hz, C_q_‐C*H*=CH), 6.09 (dd, 1H, *J*=11.2, 10.8 Hz, C_q_‐CH=CH‐C*H*), 5.55 (m, 1H, CH_2_‐CH_2_‐C*H*), 5.07 (m, 1H, Me_2_C=C*H*), 4.77 (s, br, 1H, C*H*‐OH), 4.48 (d, 1H, *J=*12.8 Hz, C*H*_2_‐OH), 4.42 (d, 1H, *J=*12.8 Hz, C*H*_2_‐OH), 3.68 (d, 1H, *J=*2.7 Hz, epoxy‐C*H*), 2.73 (dd, 1H, *J=*15.0, 7.6 Hz, Me_2_C=CH‐C*H*_2_), 2.49 (dd, 1H, *J*=15.0, 6.9 Hz, Me_2_C=CH‐C*H*_2_), 2.20 (m, 2H, CH_3_‐CH_2_‐C*H*_2_), 1.70 (s, 3H, *E*‐C*H*_3_), 1.66 (s, 3H, *Z*‐C*H*_3_), 1.43 (m, 2H, CH_3_‐C*H*_2_), 0.93 (t, 3H, *J=*7.4 Hz, C*H*_3_‐CH_2_); ^**13**^ **C‐NMR** (MeOD, 125 MHz): δ=196.7 (*C*=O), 150.6 (OH‐CH_2_‐*C*_q_), 136.5 (br, *C*_q_Me_2_), 135.1 (CH_2_‐CH_2_‐*C*H), 133.0 (C_q_‐CH=*C*H), 132.0 (*C*_q_‐CH=CH), 130.5 (C_q_‐CH=CH‐*C*H), 124.7 (C_q_‐*C*H=CH), 118.2 (*C*H=CMe_2_), 86.3 (*C*H‐OH), 62.0 (epoxy*‐C*_q_), 60.5 (epoxy‐*C*H), 60.0 (*C*H_2_‐OH), 31.0 (CH_3_‐CH_2_‐*C*H_2_), 27.6 (Me_2_C=CH‐*C*H_2_), 26.0 (*E*‐*C*H_3_), 23.9 (CH_3_‐*C*H_2_), 18.1 (*Z*‐*C*H_3_), 14.0 (*C*H_3_‐CH_2_); **HRMS (ESI)** m/z calcd. for C_19_H_27_O_4_: 319.1904 (M+H)^+^; found: 319.1903 (M+H)^+^; retardation factor and specific rotation was not determined due to low isolated quantity and the mixture of three compounds.

**Floyocidin D** ^[22]^ **/ (*R*)‐3‐((1*E*,3*E*)‐Hepta‐1,3‐dien‐1‐yl)‐5‐hydroxy‐2‐(hydroxymethyl)‐5‐(3‐methylbut‐2‐en‐1‐yl)cyclo‐hex‐2‐ene‐1,4‐dione (6)**: ^**1**^ **H‐NMR** (MeOD, 500 MHz): δ=7.08 (dd, 1H, *J*=15.5, 10.7 Hz, C_q_‐CH=C*H*), 6.60 (d, 1H, *J*=15.5 Hz, C_q_‐C*H*=CH), 6.29 (dd, 1H, *J*=15.1, 10.7 Hz, C_q_‐CH=CH‐C*H*), 6.04 (dt, 1H, *J*=15.1, 7.1 Hz, CH_2_‐CH_2_‐C*H*), 5.14 (m, 1H, Me_2_C=C*H*), 4.49 (d, 1H, *J*=11.7 Hz, C*H*_2_‐OH), 4.43 (d, 1H, *J*=11.7 Hz, C*H*_2_‐OH), 2.99 (d, 1H, *J=*16.6 Hz, C*H*_2_‐C=O), 2.81 (d, 1H, *J*=16.6 Hz, C*H*_2_‐C=O), 2.55 (dd, 1H, *J*=14.5, 8.7 Hz, Me_2_C=CH‐C*H*_2_), 2.38 (dd, 1H, *J*=14.5, 6.5 Hz, Me_2_C=CH‐C*H*_2_), 2.17 (m, 2H, CH_3_‐CH_2_‐C*H*_2_), 1.64 (s, 3H, *E*‐C*H*_3_), 1.48 (s, 3H, *Z*‐C*H*_3_), 1.47 (m, 2H, CH_3_‐C*H*_2_), 0.94 (t, 3H, *J*=7.4 Hz, C*H*_3_‐CH_2_); ^**13**^ **C‐NMR** (MeOD, 125 MHz): δ=203.2 (br, OH‐C_q_‐*C*=O), 196.4 (CH_2_‐*C=*O), 146.5 (*C*_q_‐CH=CH), 144.4 (C_q_‐CH=*C*H), 143.4 (CH_2_‐CH_2_‐*C*H), 142.1 (OH‐CH_2_‐*C*_q_), 136.9 (*C*_q_Me_2_), 133.0 (C_q_‐CH=CH‐*C*H), 122.8 (C_q_‐*C*H=CH), 118.8 (*C*H=CMe_2_), 80.2 (OH‐*C*_q_), 55.1 (*C*H_2_‐OH), 51.5 (*C*H_2_‐C*=*O), 38.7 (Me_2_C=CH‐*C*H_2_), 36.2 (CH_3_‐CH_2_‐*C*H_2_), 26.1 (*E*‐*C*H_3_), 23.2 (CH_3_‐*C*H_2_), 18.1 (*Z*‐*C*H_3_), 14.0 (*C*H_3_‐CH_2_); **HRMS (ESI)** m/z calcd. for C_19_H_27_O_4_: 319.1904 (M+H)^+^; found: 319.1901 (M+H)^+^; retardation factor and specific rotation was not determined due to low isolated quantity and impurity of the compound.

**4‐Bromo‐2‐chloro‐5‐iodopyridine (10)**: To a suspension of copper(II) bromide (4.18 g, 18.7 mmol, 1.20 eq.) in MeCN (60 mL), *t*‐BuONO (90 %, 3.10 mL, 23.4 mmol, 1.50 eq.) was added slowly. The suspension was stirred for 15 min at room temperature and was then cooled to 0 °C. Amine **11** (3.96 g, 15.6 mmol, 1.00 eq.), dissolved in MeCN (40 mL) by gentle warming, was added at 0 °C. After 1 h stirring at 0 °C, the mixture was allowed to warm to room temperature and stirred overnight. After concentration *in vacuo*,^[23]^ aqueous ammonia solution (15 %, 40 mL) was added. The mixture was extracted with DCM (3 x 30 mL) and the combined organic layers were washed with saturated aqueous NaCl (30 mL), dried over MgSO_4_, filtered, and concentrated under reduced pressure.^[23]^ The crude product was purified by flash column chromatography (100 % DCM) to give product **10** (4.84 g, 15.2 mmol, 97 %) as colorless solid. ^**1**^ **H‐NMR** (CDCl_3_, 400 MHz): δ=8.69 (s, 1H, N‐C*H*), 7.65 (s, 1H, ClC‐C*H*); ^**13**^ **C‐NMR** (CDCl_3_, 100 MHz): δ=157.3 (N‐*C*H), 151.6 (*C–*Cl), 141.5 (*C*Br), 128.4 (ClC‐*C*H), 99.1 (*C*I); **HRMS (ESI)** m/z calcd. for C_5_H_3_BrClIN: 317.8177 (M+H)^+^; found: 317.8172 (M+H)^+^; **R_f_** (DCM): 0.62.

**(*R*)‐(4‐Bromo‐6‐chloropyridin‐3‐yl)((2*R*,3*S*)‐3‐(((*tert*‐butyldiphenylsilyl)oxy)methyl)‐3‐(3‐methylbut‐2‐en‐1‐yl) oxiran‐2‐yl)methanol (12) and (*S*)‐(4‐Bromo‐6‐chloropyridin‐3‐yl)((2*R*,3*S*)‐3‐(((*tert*‐butyldiphenylsilyl)oxy)methyl)‐3‐(3‐methylbut‐2‐en‐1‐yl)oxiran‐2‐yl)methanol (13)**: The reaction was carried out in moisture‐free glassware under argon atmosphere. To a solution of iodopyridine **10** (1.91 g, 6.01 mmol, 1.40 eq.) dissolved in anhydrous THF (30 mL), *i*‐PrMgCl in THF (2.0 M, 2.79 mL, 5.58 mmol, 1.30 eq.) was added dropwise at −40 °C. After stirring for 45 min, epoxy aldehyde **9** (1.75 g, 4.29 mmol, 1.00 eq.) in anhydrous THF (10 mL) was added. The reaction mixture was stirred for 1 h 30 min at −40 °C and was then diluted with saturated aqueous NH_4_Cl (50 mL). The mixture was extracted with ethyl acetate (2×150 mL) and the combined organic layers were washed with saturated aqueous NaCl (75 mL), dried over MgSO_4_, filtered, and concentrated under reduced pressure. The crude product was purified by flash column chromatography (0–20 % ethyl acetate in *n*‐heptane) to give addition products **12** (1.25 g, 2.09 mmol, 49 % over two steps) and **13** (1.09 g, 1.89 mmol, 42 % over two steps) as colorless oils. For **12**: ^**1**^ **H‐NMR** (CDCl_3_, 400 MHz): δ=8.54 (s, 1H, N‐C*H*), 7.73–7.66 (m, 4H, phenyl‐*H*), 7.53 (s, 1H, ClC‐C*H*), 7.51–7.38 (m, 6H, phenyl‐*H*), 4.99 (m, 1H, Me_2_C=C*H*), 4.96 (d, 1H, *J*=8.2 Hz, C*H*‐OH), 4.03 (d, 1H, *J*=11.2 Hz, TBDPSO‐C*H*_2_), 3.79 (d, 1H, *J*=11.0 Hz, TBDPSO‐C*H*_2_), 3.29 (s, 1H, O*H*), 3.07 (d, 1H, *J*=7.9 Hz, epoxy‐C*H*), 2.44 (m, 2H, Me_2_C=CH‐C*H*_2_), 1.66 (s, 3H, *E*‐C*H*_3_), 1.57 (s, 3H, *Z*‐C*H*_3_), 1.12 (s, 9H, ^*t*^Bu‐C*H*_3_); ^**13**^ **C‐NMR** (CDCl_3_, 100 MHz): δ=151.3 (Cl–*C*), 149.3 (N‐*C*H), 136.0 (*C*_q_Me_2_), 135.9/135.7 (phenyl‐*C*H), 135.5 (N‐CH‐*C*_q_), 134.6 (*C*‐Br), 132.4/132.2 (phenyl‐*C*_q_), 130.4 (phenyl‐*C*H), 128.2/128.1 (phenyl‐*C*H), 127.7 (ClC‐*C*H*)*, 117.3 (*C*H=CMe_2_), 69.6 (*C*H‐OH), 65.5 (TBDPSO‐*C*H_2_), 64.3 (epoxy‐*C*H), 63.8 (epoxy‐*C*_q_), 32.0 (Me_2_C=CH‐*C*H_2_), 27.1 (^*t*^Bu‐*C*H_3_), 25.9 (*E*‐*C*H_3_), 19.3 (^*t*^Bu‐*C*_q_), 18.1 (*Z*‐*C*H_3_); **HRMS (ESI)** m/z calcd. for C_30_H_36_BrClNO_3_Si: 600.1331 (M+H)^+^; found: 600.1327 (M+H)^+^; **R_f_** (*n*‐heptane/ethyl acetate 4 : 1): 0.32; **Specific rotation** ${\left[\alpha \right]}_{D}^{24.7}$ =+15.1 (c=1.53; CHCl_3_). For **13**: ^**1**^ **H‐NMR** (CDCl_3_, 400 MHz): δ=8.49 (s, 1H, N‐C*H*), 7.72–7.61 (m, 4H, phenyl‐*H*), 7.51 (s, 1H, ClC‐C*H*), 7.49–7.36 (m, 6H, phenyl‐*H*), 4.99 (m, 1H, Me_2_C=C*H*), 4.86 (d, 1H, *J*=6.1 Hz, C*H*‐OH), 3.86 (d, 1H, *J*=11.4 Hz, TBDPSO‐C*H*_2_), 3.80 (d, 1H, *J*=11.5 Hz, TBDPSO‐C*H*_2_), 3.09 (d, 1H, *J*=6.0 Hz, epoxy‐C*H*), 2.81 (d, 1H, *J*=2.6 Hz, O*H*), 2.67 (dd, 1H, *J*=15.0, 8.1 Hz Me_2_C=CH‐C*H*_2_), 2.30 (dd, 1H, *J*=15.0, 6.8 Hz Me_2_C=CH‐C*H*_2_), 1.67 (s, 3H, *E*‐C*H*_3_), 1.57 (s, 3H, *Z*‐C*H*_3_), 1.07 (s, 9H, ^*t*^Bu‐C*H*_3_); ^**13**^ **C‐NMR** (CDCl_3_, 100 MHz): δ=151.4 (Cl−*C*), 149.2 (N‐*C*H), 136.0 (*C*_q_Me_2_), 135.9/135.8 (phenyl‐*C*H), 135.5 (N‐CH‐*C*_q_), 134.0 (*C*‐Br), 133.1/132.9 (phenyl‐*C*_q_), 130.1/130.1 (phenyl‐*C*H), 127.9/127.9 (phenyl‐*C*H), 127.7 (ClC‐*C*H*)*, 117.3 (*C*H=CMe_2_), 67.8 (*C*H‐OH), 65.8 (epoxy‐*C*_q_), 64.0 (TBDPSO‐*C*H_2_), 63.8 (epoxy‐*C*H), 31.3 (Me_2_C=CH‐*C*H_2_), 26.9 (^*t*^Bu‐*C*H_3_), 25.9 (*E*‐*C*H_3_), 19.4 (^*t*^Bu‐*C*_q_), 18.1 (*Z*‐*C*H_3_); **HRMS (ESI)** m/z calcd. for C_30_H_36_BrClNO_3_Si: 600.1331 (M+H)^+^; found: 600.1329 (M+H)^+^; **R_f_** (*n*‐heptane/ethyl acetate 4 : 1): 0.25; **Specific rotation** ${\left[\alpha \right]}_{D}^{24.7}$ =<‐M‐>2.2 (c=1.39; CHCl_3_).

***Ent*** **‐12** (1.21 g, 2.01 mmol, 50 % over two steps) and ***ent*** **‐13** (1.01 g, 1.69 mmol, 42 % over two steps) were synthesized in analogous manner to **12** and **13** starting from epoxy aldehyde ***ent*** **‐9** (1.65 g, 4.05 mmol) and iodide **10** (1.80 g, 5.66 mmol). The NMR data are identical to the ones reported for **12** and **13**. For ***ent*** **‐12**: **HRMS (ESI)** m/z calcd. for C_30_H_36_BrClNO_3_Si: 600.1331 (M+H)^+^; found: 600.1326 (M+H)^+^; **R_f_** (*n*‐heptane/ethyl acetate 4 : 1): 0.32; **Specific rotation** ${\left[\alpha \right]}_{D}^{18.5}$ =−25.5 (c=0.94; CHCl_3_). For ***ent*** **‐13**: **HRMS (ESI)** m/z calcd. for C_30_H_36_BrClNO_3_Si: 600.1331 (M+H)^+^; found: 600.1332 (M+H)^+^; **R_f_** (*n*‐heptane/ethyl acetate 4 : 1): 0.25; **Specific rotation** ${\left[\alpha \right]}_{D}^{18.5}$ =+4.2 (c=1.42; CHCl_3_).

**(*R*)‐(4‐Bromo‐6‐chloropyridin‐3‐yl)((2*R*,3*S*)‐3‐(((*tert*‐butyldiphenylsilyl)oxy)methyl)‐3‐(3‐methylbut‐2‐en‐1‐yl)oxiran‐2‐yl)methyl acetate (14)**: To a solution of alcohol **12** (0.243 g, 0.404 mmol, 1.00 eq.) in anhydrous DCM (7 mL), anhydrous pyridine (0.098 mL, 1.2 mmol, 3.00 eq.), Ac_2_O (0.115 mL, 1.21 mmol, 3.00 eq.) and DMAP (0.005 g, 0.04 mmol, 0.10 eq.) were added. The reaction mixture was stirred for 2 h 30 min until the TLC showed complete conversion. Toluene (10 mL) was added and the reaction mixture was concentrated *in vacuo*. The crude product was purified by flash column chromatography (0–30 % ethyl acetate in *n*‐heptane) to give **14** (0.249 g, 0.387 mmol, 97 %) as a colorless oil. ^**1**^ **H‐NMR** (CDCl_3_, 400 MHz): δ=8.34 (s, 1H, N‐C*H*), 7.70–7.63 (m, 4H, Ar‐*H*), 7.49 (s, 1H, ClC‐C*H*), 7.47–7.34 (m, 6H, Ar‐*H*), 5.79 (d, 1H, *J*=7.9 Hz, C*H*‐OAc), 5.05 (m, 1H, Me_2_C=C*H*), 3.91 (d, 1H, *J*=11.2 Hz, TBDPSO‐C*H*_2_), 3.84 (d, 1H, *J*=11.4 Hz, TBDPSO‐C*H*_2_), 3.21 (d, 1H, *J*=7.9 Hz, epoxy‐C*H*), 2.67 (dd, 1H, *J*=15.1, 7.6 Hz, Me_2_C=CH‐C*H*_2_), 2.41 (dd, 1H, *J*=15.1, 6.9 Hz, Me_2_C=CH‐C*H*_2_), 2.02 (s, 3H, CO‐CH_3_), 1.71 (s, 3H, *E*‐C*H*_3_), 1.62 (s, 3H, *Z*‐C*H*_3_), 1.07 (s, 9H, ^*t*^Bu‐C*H*_3_); ^**13**^ **C‐NMR** (CDCl_3_, 100 MHz): δ=169.1 (C=O), 151.7 (Cl–*C*), 149.5 (N‐*C*H), 135.8 (*C*_q_Me_2_), 135.9/135.8 (Ar‐*C*), 135.5 (*C*‐Br), 133.2/133.1 (Ar‐*C*_q_), 132.9 (N‐CH‐*C*_q_), 130.0 (Ar‐*C*), 128.0 (ClC‐*C*H*)*, 128.0/127.9 (Ar‐*C*), 117.5 (*C*H=CMe_2_), 69.4 (*C*H‐OAc), 65.6 (epoxy‐*C*_q_), 64.0 (TBDPSO‐*C*H_2_), 61.9 (epoxy‐*C*H), 31.1 (Me_2_C=CH‐*C*H_2_), 26.9 (^*t*^Bu‐*C*H_3_), 26.0 (*E*‐*C*H_3_), 20.8 (CO‐*C*H_3_), 19.4 (^*t*^Bu‐*C*_q_), 18.2 (*Z*‐*C*H_3_); **HRMS (ESI)** m/z calcd. for C_32_H_38_BrClNO_4_Si: 642.1437 (M+H)^+^; found: 642.1441 (M+H)^+^; **R_f_** (*n*‐heptane/ethyl acetate 4 : 1): 0.46; **Specific rotation** ${\left[\alpha \right]}_{D}^{22.3}$ =+15.0 (c=1.07; CHCl_3_).

**(*S*)‐(4‐Bromo‐6‐chloropyridin‐3‐yl)((2*R*,3*S*)‐3‐(((*tert*‐butyldiphenylsilyl)oxy)methyl)‐3‐(3‐methylbut‐2‐en‐1‐yl)oxiran‐2‐yl)methyl acetate (18): 18** (0.229 g, 0.356 mmol, 98 %) was synthesized in analogous manner to **14** starting from **13** (0.218 g, 0.363 mmol) and was obtained as colorless oil. ^**1**^ **H‐NMR** (CDCl_3_, 400 MHz): δ=8.32 (s, 1H, N‐C*H*), 7.65–7.56 (m, 4H, Ar‐*H*), 7.48 (s, 1H, ClC‐C*H*), 7.46–7.34 (m, 6H, Ar‐*H*), 5.81 (d, 1H, *J*=8.1 Hz, C*H*‐OAc), 4.92 (m, 1H, Me_2_C=C*H*), 3.76 (d, 1H, *J*=11.4 Hz, TBDPSO‐C*H*_2_), 3.70 (d, 1H, *J*=11.5 Hz, TBDPSO‐C*H*_2_), 3.37 (d, 1H, *J*=8.1 Hz, epoxy‐C*H*), 2.66 (dd, 1H, *J*=15.1, 8.0 Hz, Me_2_C=CH‐C*H*_2_), 2.26 (dd, 1H, *J*=15.0, 6.7 Hz, Me_2_C=CH‐C*H*_2_), 2.04 (s, 3H, CO‐CH_3_), 1.66 (s, 3H, *E*‐C*H*_3_), 1.56 (s, 3H, *Z*‐C*H*_3_), 1.03 (s, 9H, ^*t*^Bu‐C*H*_3_); ^**13**^ **C‐NMR** (CDCl_3_, 100 MHz): δ=169.4 (C=O), 152.0 (Cl–*C*), 149.6 (N‐*C*H), 136.0 (*C*_q_Me_2_), 135.9/135.8 (Ar‐*C*), 135.0 (*C*‐Br), 133.2/132.8 (Ar‐*C*_q_), 132.5 (N‐CH‐*C*_q_), 130.0/130.0 (Ar‐*C*), 128.4 (ClC‐*C*H*)*, 127.9/127.9 (Ar‐*C*), 117.3 (*C*H=CMe_2_), 71.5 (*C*H‐OAc), 64.8 (TBDPSO‐*C*H_2_), 64.6 (epoxy‐*C*_q_), 61.5 (epoxy‐*C*H), 30.8 (Me_2_C=CH‐*C*H_2_), 26.9 (^*t*^Bu‐*C*H_3_), 25.9 (*E*‐*C*H_3_), 20.9 (CO‐*C*H_3_), 19.4 (^*t*^Bu‐*C*_q_), 18.2 (*Z*‐*C*H_3_); **HRMS (ESI)** m/z calcd. for C_32_H_38_BrClNO_4_Si: 642.1437 (M+H)^+^; found: 642.1437 (M+H)^+^; **R_f_** (*n*‐heptane/ethyl acetate 4 : 1): 0.34; **Specific rotation** ${\left[\alpha \right]}_{D}^{22.3}$ =+2.6 (c=1.15; CHCl_3_).

***Ent*** **‐14** (1.25 g, 1.94 mmol, 97 %) and ***ent*** **‐18** (1.06 g, 1.65 mmol, 98 %) were synthesized in analogous manner to **14** and **18** starting from ***ent*** **‐12** (1.20 g, 2.00 mmol) and ***ent*** **‐13** (1.01 g, 1.68 mmol). The NMR data are identical to the ones reported for **14** and **18**. For ***ent*** **‐14**: **HRMS (ESI)** m/z calcd. for C_32_H_38_BrClNO_4_Si: 642.1437 (M+H)^+^; found: 642.1440 (M+H)^+^; **R_f_** (*n*‐heptane/ethyl acetate 4 : 1): 0.46; **Specific rotation** ${\left[\alpha \right]}_{D}^{20.4}$ =−16.0 (c=2.25; CHCl_3_). For ***ent*** **‐18**: **HRMS (ESI)** m/z calcd. for C_32_H_38_BrClNO_4_Si: 642.1437 (M+H)^+^; found: 642.1449 (M+H)^+^; **R_f_** (*n*‐heptane/ethyl acetate 4 : 1): 0.34; **Specific rotation** ${\left[\alpha \right]}_{D}^{20.4}$ =−5.5 (c=1.27; CHCl_3_).

**((2*S*,3*R*)‐3‐((*R*)‐(4‐Bromo‐6‐chloropyridin‐3‐yl)(hydroxy)methyl)‐2‐(3‐methylbut‐2‐en‐1‐yl)oxiran‐2‐yl)methyl acetate (15)**: In a 50 mL falcon tube, acetic acid glacial (0.164 mL, 2.87 mmol, 1.40 eq.) followed by TBAF in THF (1 M in THF, 2.50 mL, 2.46 mmol, 1.20 eq.) were added to a solution of TBDPS‐protected alcohol **14** (1.32 g, 2.05 mmol, 1.00 eq.) in THF (25 mL). After 2 h, a second portion of TBAF (0.20 eq.) was added. Complete consumption of the starting material, which was monitored by TLC, was achieved after 1 h. The acetate migration from secondary to primary hydroxy was monitored by LC–MS. After stirring overnight at room temperature, LC–MS indicated an acyl migration to the primary hydroxy group of >90 %.^[24]^ The reaction mixture was loaded on silica and purified *via* flash column chromatography (*n*‐heptane / ethyl acetate 3 : 1) to give **15** (0.715 g, 1.77 mmol, 86 %) as colorless oil. ^**1**^ **H‐NMR** (CDCl_3_, 400 MHz): δ=8.57 (s, 1H, N‐C*H*), 7.56 (s, 1H, ClC‐C*H*), 5.12 (d, 1H, *J*=8.2 Hz, C*H*‐OH), 5.09 (m, 1H, Me_2_C=C*H*), 4.37 (d, 1H, *J*=12.0 Hz, C*H*_2_‐OAc), 4.28 (d, 1H, *J*=12.2 Hz, C*H*_2_‐OAc), 3.70 (s, br, 1H, O*H*), 3.11 (d, 1H, *J*=8.1 Hz, epoxy‐C*H*), 2.41 (dd, 1H, *J*=15.0, 7.4 Hz, Me_2_C=CH‐C*H*_2_), 2.30 (dd, 1H, *J*=15.0, 7.7 Hz, Me_2_C=CH‐C*H*_2_), 2.15 (s, 3H, CO‐CH_3_), 1.72 (s, 3H, *E*‐C*H*_3_), 1.62 (s, 3H, *Z*‐C*H*_3_); ^**13**^ **C‐NMR** (CDCl_3_, 100 MHz): δ=171.9 (C=O), 151.4 (Cl–*C*), 149.5 (N‐*C*H), 136.4 (*C*_q_Me_2_), 135.4 (N‐CH‐*C*_q_), 134.7 (*C*‐Br), 127.7 (ClC‐*C*H*)*, 116.8 (*C*H=CMe_2_), 68.4 (*C*H‐OH), 65.4 (epoxy‐*C*H), 63.1 (*C*H_2_‐OAc), 61.9 (epoxy‐*C*_q_), 32.0 (Me_2_C=CH‐*C*H_2_), 26.0 (*E*‐*C*H_3_), 21.0 (CO‐*C*H_3_), 18.1 (*Z*‐*C*H_3_); **HRMS (ESI)** m/z calcd. for C_16_H_20_BrClNO_4_: 404.0259 (M+H)^+^; found: 404.0255 (M+H)^+^; **R_f_** (*n*‐heptane/ethyl acetate 2 : 1): 0.31; **Specific rotation** ${\left[\alpha \right]}_{D}^{23.3}$ =−8.5 (c=0.82; CHCl_3_).

**((2*S*,3*R*)‐3‐((*S*)‐(4‐Bromo‐6‐chloropyridin‐3‐yl)(hydroxy)methyl)‐2‐(3‐methylbut‐2‐en‐1‐yl)oxiran‐2‐yl)methyl acetate (19): 19** (0.694 g, 1.71 mmol, 90 %)^[24]^ was synthesized in analogous manner to **15** starting from **18** (1.23 g, 1.91 mmol) and was obtained as colorless oil. ^**1**^ **H‐NMR** (CDCl_3_, 400 MHz): δ=8.56 (s, 1H, N‐C*H*), 7.59 (s, 1H, ClC‐C*H*), 5.07–4.96 (m, 2H, Me_2_C=C*H*, C*H*‐OH), 4.32 (d, 1H, *J*=12.2 Hz, C*H*_2_‐OAc), 4.29 (d, 1H, *J*=12.1 Hz, C*H*_2_‐OAc), 3.16 (d, 1H, *J*=6.2 Hz, epoxy‐C*H*), 2.86 (s, br, 1H, O*H*), 2.55 (dd, 1H, *J*=15.0, 8.1 Hz, Me_2_C=CH‐C*H*_2_), 2.26 (dd, 1H, *J*=15.0, 7.0 Hz, Me_2_C=CH‐C*H*_2_), 2.08 (s, 3H, CO‐CH_3_), 1.70 (s, 3H, *E*‐C*H*_3_), 1.58 (s, 3H, *Z*‐C*H*_3_); ^**13**^ **C‐NMR** (CDCl_3_, 100 MHz): δ=170.7 (C=O), 151.4 (Cl–*C*), 149.3 (N‐*C*H), 136.8 (*C*_q_Me_2_), 135.1 (N‐CH‐*C*_q_), 134.0 (*C*‐Br), 127.8 (ClC‐*C*H*)*, 116.5 (*C*H=CMe_2_), 68.1 (*C*H‐OH), 64.2 (epoxy‐*C*H), 63.9 (*C*H_2_‐OAc), 63.5 (epoxy‐*C*_q_), 31.9 (Me_2_C=CH‐*C*H_2_), 26.0 (*E*‐*C*H_3_), 20.9 (CO‐*C*H_3_), 18.1 (*Z*‐*C*H_3_); **HRMS (ESI)** m/z calcd. for C_16_H_20_BrClNO_4_: 404.0259 (M+H)^+^; found: 404.0255 (M+H)^+^; **R_f_** (*n*‐heptane/ethyl acetate 2 : 1): 0.29; **Specific rotation** ${\left[\alpha \right]}_{D}^{23.3}$ =+12.3 (c=0.98; CHCl_3_).

***Ent*** **‐15** (0.670 g, 1.66 mmol, 86 %)^[24]^ and ***ent*** **‐19** (0.600 g, 1.48 mmol, 90 %)^[24]^ were synthesized in analogous manner to **15** and **19** starting from ***ent*** **‐14** (1.24 g, 1.93 mmol) and ***ent*** **‐18** (1.06 g, 1.65 mmol). The NMR data are identical to the ones reported for **15** and **19**. For ***ent*** **‐15**: **HRMS (ESI)** m/z calcd. for C_16_H_20_BrClNO_4_: 404.0259 (M+H)^+^; found: 404.0258 (M+H)^+^; **R_f_** (*n*‐heptane/ethyl acetate 2 : 1): 0.31; **Specific rotation** ${\left[\alpha \right]}_{D}^{21.2}$ =+5.1 (c=1.17; CHCl_3_). For ***ent*** **‐19**: **HRMS (ESI)** m/z calcd. for C_16_H_20_BrClNO_4_: 404.0259 (M+H)^+^; found: 404.0262 (M+H)^+^; **R_f_** (*n*‐heptane/ethyl acetate 2 : 1): 0.29; **Specific rotation** ${\left[\alpha \right]}_{D}^{21.2}$ =−11.8 (c=1.19; CHCl_3_).

**((2*S*,3*R*)‐3‐((*R*)‐(4‐Bromo‐6‐chloropyridin‐3‐yl)((triisopropylsilyl)oxy)methyl)‐2‐(3‐methylbut‐2‐en‐1‐yl)oxiran‐2‐yl)methanol (17)**: 2,6‐Lutidine (1.20 mL, 10.4 mmol, 6.00 eq.) and then TIPSOTf (1.40 mL, 5.17 mmol, 3.00 eq.) were added to a solution of alcohol **15** (0.698 g, 1.73 mmol, 1.00 eq.) in anhydrous DCM (20 mL) at 0 °C. The reaction mixture was allowed to stir at room temperature. After 1 h 30 min and 4 h, additional portions of 2,6‐lutidine (4.00 eq.) and TIPSOTf (2.00 eq.) were added to drive the reaction to complete conversion of starting material **15**. After stirring overnight at room temperature, the TLC showed complete conversion. Saturated aqueous NH_4_Cl (30 mL) was added. The mixture was extracted with ethyl acetate (2 x 100 mL) and the combined organic layers were washed with saturated aqueous NaCl (70 mL), dried over MgSO_4_, filtered, and concentrated under reduced pressure. The crude product of **16** was pre‐purified by flash column chromatography (0–18 % ethyl acetate in *n*‐heptane).

Pre‐purified crude **16** was dissolved in THF/H_2_O (5 : 1, 60 mL/12 mL) and LiOH ⋅ H_2_O (0.145 g, 3.45 mmol, 2.00 eq.) was added. After stirring for 7 h at room temperature, the TLC showed complete conversion. Saturated aqueous NaHCO_3_ (50 mL) was added. The mixture was extracted with ethyl acetate (2 x 100 mL) and the combined organic layers were washed with saturated aqueous NaCl (70 mL), dried over MgSO_4_, filtered, and concentrated under reduced pressure. The crude product was purified by flash column chromatography (0–25 % ethyl acetate in *n*‐heptane) to give **17** (0.491 g, 0.946 mmol, 55 % over two steps) as a colorless solid. In a 4 mL vial, a small portion of **17** was dissolved in DCM (1 mL) and *n*‐heptane (1.5 mL) was added. The system was left at room temperature for one night. A crystal was obtained for structure elucidation by X‐ray crystal diffraction (see chapter 4 of Supporting Information). ^**1**^ **H‐NMR** (CDCl_3_, 400 MHz): δ=8.57 (s, 1H, N‐C*H*), 7.51 (s, 1H, ClC‐C*H*), 5.29 (d, 1H, *J*=7.7 Hz, C*H*‐OTIPS), 5.08 (m, 1H, Me_2_C=C*H*), 3.94 (s, 2H, C*H*_2_‐OH), 3.09 (d, 1H, *J*=7.9 Hz, epoxy‐C*H*), 2.64 (dd, 1H, *J*=14.7, 8.2 Hz, Me_2_C=CH‐C*H*_2_), 2.12 (dd, 1H, *J*=14.7, 7.0 Hz, Me_2_C=CH‐C*H*_2_), 1.81 (s, br, 1H, O*H*), 1.70 (s, 3H, *E*‐C*H*_3_), 1.63 (s, 3H, *Z*‐C*H*_3_), 1.09–0.96 (m, 21H, TIPS‐*H*); ^**13**^ **C‐NMR** (CDCl_3_, 100 MHz): δ=151.1 (Cl–*C*), 150.3 (N‐*C*H), 137.2 (*C*‐Br or N‐CH‐*C*_q_), 136.1 (*C*_q_Me_2_), 133.8 (N‐CH‐*C*_q_ or *C*‐Br), 127.4 (ClC‐*C*H*)*, 117.5 (*C*H=CMe_2_), 68.3 (*C*H‐OTIPS), 67.3 (epoxy‐*C*H), 65.9 (epoxy‐*C*_q_), 62.2 (*C*H_2_‐OH), 32.0 (Me_2_C=CH‐*C*H_2_), 25.9 (*E*‐*C*H_3_), 18.1 (*Z*‐*C*H_3_), 17.9/17.9 (CH(*C*H_3_)_2_), 12.3 (*C*H(CH_3_)_2_); **HRMS (ESI)** m/z calcd. for C_23_H_38_BrClNO_3_Si: 518.1487 (M+H)^+^; found: 518.1482 (M+H)^+^; **R_f_** (*n*‐heptane/ethyl acetate 4 : 1): 0.32; **Specific rotation** ${\left[\alpha \right]}_{D}^{22.9}$ =−19.3 (c=1.61; CHCl_3_).

**((2*S*,3*R*)‐3‐((*S*)‐(4‐Bromo‐6‐chloropyridin‐3‐yl)((triisopropylsilyl)oxy)methyl)‐2‐(3‐methylbut‐2‐en‐1‐yl)oxiran‐2‐yl)methanol (21): 21** (0.527 g, 1.02 mmol, 71 % over two steps) was synthesized in analogous manner to **17** starting from **19** (0.583 g, 1.44 mmol) and was obtained as colorless oil. ^**1**^ **H‐NMR** (CDCl_3_, 400 MHz): δ=8.50 (s, 1H, N‐C*H*), 7.54 (s, 1H, ClC‐C*H*), 5.20 (d, 1H, *J*=7.7 Hz, C*H*‐OTIPS), 4.93 (m, 1H, Me_2_C=C*H*), 3.72 (dd, 1H, *J*=12.1, 6.2 Hz, C*H*_2_‐OH), 3.66 (dd, 1H, *J*=12.3, 5.7 Hz, C*H*_2_‐OH), 3.31 (d, 1H, *J*=7.7 Hz, epoxy‐C*H*), 2.46 (dd, 1H, *J*=15.0, 7.7 Hz, Me_2_C=CH‐C*H*_2_), 2.14 (dd, 1H, *J*=14.9, 7.2 Hz, Me_2_C=CH‐C*H*_2_), 1.73 (t, 1H, *J*=6.3 Hz, O*H*), 1.64 (s, 3H, *E*‐C*H*_3_), 1.53 (s, 3H, *Z*‐C*H*_3_), 1.15–0.95 (m, 21H, TIPS‐*H*); ^**13**^ **C‐NMR** (CDCl_3_, 100 MHz): δ=151.2 (Cl–*C*), 150.6 (N‐*C*H), 136.4 (*C*‐Br), 136.0 (*C*_q_Me_2_), 133.3 (N‐CH‐*C*_q_), 127.8 (ClC‐*C*H*)*, 117.3 (*C*H=CMe_2_), 71.0 (*C*H‐OTIPS), 66.9 (epoxy‐*C*H), 64.9 (epoxy‐*C*_q_), 62.7 (*C*H_2_‐OH), 32.0 (Me_2_C=CH‐*C*H_2_), 25.9 (*E*‐*C*H_3_), 18.1 (*Z*‐*C*H_3_), 17.9/17.9 (CH(*C*H_3_)_2_), 12.3 (*C*H(CH_3_)_2_); **HRMS (ESI)** m/z calcd. for C_23_H_38_BrClNO_3_Si: 518.1487 (M+H)^+^; found: 518.1490 (M+H)^+^; **R_f_** (*n*‐heptane/ethyl acetate 4 : 1): 0.32; **Specific rotation** ${\left[\alpha \right]}_{D}^{22.9}$ =+6.0 (c=1.34; CHCl_3_).

***Ent*** **‐17** (0.442 g, 0.852 mmol, 53 % over two steps) and ***ent*** **‐21** (0.434 g, 0.836 mmol, 57 % over two steps) were synthesized in analogous manner to **17** and **21** starting from ***ent*** **‐15** (0.655 g, 1.62 mmol) and ***ent*** **‐19** (0.593 g, 1.47 mmol). The NMR data are identical to the ones reported for **17** and **21**. For ***ent*** **‐17**: **HRMS (ESI)** m/z calcd. for C_23_H_38_BrClNO_3_Si: 518.1487 (M+H)^+^; found: 518.1491 (M+H)^+^; **R_f_** (*n*‐heptane/ethyl acetate 4 : 1): 0.32; **Specific rotation** ${\left[\alpha \right]}_{D}^{19.7}$ =+35.9 (c=1.03; CHCl_3_). For ***ent*** **‐21**: **HRMS (ESI)** m/z calcd. for C_23_H_38_BrClNO_3_Si: 518.1487 (M+H)^+^; found: 518.1479 (M+H)^+^; **R_f_** (*n*‐heptane/ethyl acetate4 : 1): 0.32; **Specific rotation** ${\left[\alpha \right]}_{D}^{19.7}$ =−8.5 (c=1.41; CHCl_3_).

**(2*S*,3*R*)‐3‐((*R*)‐(4‐Bromo‐6‐chloropyridin‐3‐yl)((triisopropylsilyl)oxy)methyl)‐2‐(3‐methylbut‐2‐en‐1‐yl)oxirane‐2‐carbonitrile (8)**: Alcohol **17** (0.382 g, 0.735 mmol, 1.00 eq.) was dissolved in MeCN/H_2_O 9 : 1 (27 mL/3.0 mL) and NH_4_OAc (0.227 g, 2.94 mmol, 4.00 eq.), BAIB (0.711 g, 2.21 mmol, 3.00 eq.), as well as TEMPO (0.023 g, 0.15 mmol, 0.20 eq.) were added. The reaction solution was allowed to stir for 6 h 30 min at room temperature, until the TLC and the LC–MS showed complete conversion to the nitrile (n.b. the starting material is first converted to the intermediate aldehyde and then converted to the nitrile in a two‐step fashion). Saturated aqueous Na_2_SO_3_ (40 mL) was added. The mixture was extracted with ethyl acetate (80 mL) and the organic layer was washed with saturated aqueous NaCl (50 mL), dried over MgSO_4_, filtered, and concentrated under reduced pressure. The crude product was purified *via* flash column chromatography (0–15 % ethyl acetate in *n*‐heptane) to give nitrile **8** (0.265 g, 0.515 mmol, 70 %) as a colorless oil. ^**1**^ **H‐NMR** (CDCl_3_, 400 MHz): δ=8.62 (s, 1H, N‐C*H*), 7.56 (s, 1H, ClC‐C*H*), 5.34 (d, 1H, *J*=6.8 Hz, C*H*‐OTIPS), 5.11 (m, 1H, Me_2_C=C*H*), 3.18 (d, 1H, *J*=6.8 Hz, epoxy‐C*H)*, 2.62 (dd, 1H, *J*=15.0, 7.9 Hz, Me_2_C=CH‐C*H*_2_), 2.48 (dd, 1H, *J*=15.1, 7.3 Hz, Me_2_C=CH‐C*H*_2_), 1.75 (s, 3H, *E*‐C*H*_3_), 1.65 (s, 3H, *Z*‐C*H*_3_), 1.21–0.99 (m, 21H, TIPS‐*H*); ^**13**^ **C‐NMR** (CDCl_3_, 100 MHz): δ=151.7 (Cl–*C*), 150.0 (N‐*C*H), 139.3 (*C*_q_Me_2_), 135.7 (N‐CH‐*C*_q_), 134.2 (*C*‐Br), 127.7 (ClC‐*C*H*)*, 116.8 (*C*N), 114.2 (*C*H=CMe_2_), 69.3 (*C*H‐OTIPS), 65.4 (epoxy‐*C*H), 55.0 (epoxy‐*C*_q_), 32.6 (Me_2_C=CH‐*C*H_2_), 26.0 (*E*‐*C*H_3_), 18.2 (*Z*‐*C*H_3_), 18.0/17.9 (CH(*C*H_3_)_2_), 12.2 (*C*H(CH_3_)_2_); **HRMS (ESI)** m/z calcd. for C_23_H_35_BrClN_2_O_2_Si: 513.1334 (M+H)^+^; found: 513.1331 (M+H)^+^; **R_f_** (*n*‐heptane/ethyl acetate 4 : 1): 0.59; **Specific rotation** ${\left[\alpha \right]}_{D}^{19.8}$ =+37.8 (c=1.14; CHCl_3_).

**(2*S*,3*R*)‐3‐((*S*)‐(4‐Bromo‐6‐chloropyridin‐3‐yl)((triisopropylsilyl)oxy)methyl)‐2‐(3‐methylbut‐2‐en‐1‐yl)oxirane‐2‐carbonitrile (22): 22** (0.244 g, 0.475 mmol, 60 %) was synthesized in analogous manner to **8** starting from **21** (0.410 g, 0.789 mmol) and was obtained as colorless oil. ^**1**^ **H‐NMR** (CDCl_3_, 400 MHz): δ=8.55 (s, 1H, N‐C*H*), 7.59 (s, 1H, ClC‐C*H*), 5.14 (d, 1H, *J*=7.7 Hz, C*H*‐OTIPS), 5.03 (m, 1H, Me_2_C=C*H*), 3.40 (d, 1H, *J*=7.6 Hz, epoxy‐C*H)*, 2.52 (dd, 1H, *J*=15.0, 7.6 Hz, Me_2_C=CH‐C*H*_2_), 2.43 (dd, 1H, *J*=15.0, 7.3 Hz, Me_2_C=CH‐C*H*_2_), 1.71 (s, 3H, *E*‐C*H*_3_), 1.58 (s, 3H, *Z*‐C*H*_3_), 1.19–0.96 (m, 21H, TIPS‐*H*); ^**13**^ **C‐NMR** (CDCl_3_, 100 MHz): δ=151.9 (Cl–*C*), 150.7 (N‐*C*H), 139.2 (*C*_q_Me_2_), 134.7 (N‐CH‐*C*_q_), 133.8 (*C*‐Br), 128.2 (ClC‐*C*H*)*, 117.2 (*C*N), 114.2 (*C*H=CMe_2_), 72.1 (*C*H‐OTIPS), 65.1 (epoxy‐*C*H), 53.7 (epoxy‐*C*_q_), 32.9 (Me_2_C=CH‐*C*H_2_), 25.9 (*E*‐*C*H_3_), 18.3 (*Z*‐*C*H_3_), 17.9/17.9 (CH(*C*H_3_)_2_), 12.3(*C*H(CH_3_)_2_); **HRMS (ESI)** m/z calcd. for C_23_H_35_BrClN_2_O_2_Si: 513.1334 (M+H)^+^; found: 513.1329 (M+H)^+^; **R_f_** (*n*‐heptane/ethyl acetate 4 : 1): 0.59; **Specific rotation** ${\left[\alpha \right]}_{D}^{20.2}$ =+10.3 (c=0.97; CHCl_3_).

***Ent*** **‐8** (0.101 g, 0.197 mmol, 25 %) and ***ent*** **‐22** (0.129 g, 0.251 mmol, 31 %) were synthesized in analogous manner^[25]^ to **8** and **22** starting from ***ent*** **‐17** (0.409 g, 0.788 mmol) and ***ent*** **‐21** (0.426 g, 0.821 mmol). The NMR data are identical to the ones reported for **8** and **22**. For ***ent*** **‐8**: **HRMS (ESI)** m/z calcd. for C_23_H_35_BrClN_2_O_2_Si: 513.1334 (M+H)^+^; found: 513.1340 (M+H)^+^; **R_f_** (*n*‐heptane/ethyl acetate 4 : 1): 0.59; **Specific rotation** ${\left[\alpha \right]}_{D}^{21.6}$ =−40.7 (c=0.91; CHCl_3_). For ***ent*** **‐22**: **HRMS (ESI)** m/z calcd. for C_23_H_35_BrClN_2_O_2_Si: 513.1334 (M+H)^+^; found: 513.1329 (M+H)^+^; **R_f_** (*n*‐heptane/ethyl acetate 4 : 1): 0.59; **Specific rotation** ${\left[\alpha \right]}_{D}^{22.3}$ =−5.5 (c=1.09; CHCl_3_).

**(1a*R*,2*R*,7a*R*)‐5‐Chloro‐7 a‐(3‐methylbut‐2‐en‐1‐yl)‐2‐((triisopropylsilyl)oxy)‐1 a,7 a‐dihydrooxireno[2,3‐g]isoquinolin‐7(2*H*)‐one (7)**: The halogen‐lithium exchange was carried out in a moisture‐free glassware under argon atmosphere. To a solution of nitrile **8** (0.154 g, 0.300 mmol, 1.00 eq.) in anhydrous THF (10 mL), prediluted *n*‐BuLi solution (0.30 mmol, 1.00 eq.) was added at −100 °C.^[26]^ The obtained red solution was stirred for 20 min at –100 °C and for 5 min without cooling bath. For hydrolysis of the intermediate imine, aqueous citric acid (10 %, 15 mL) was added and the mixture was extracted with ethyl acetate (2 x 50 mL). The combined organic layers were washed with saturated aqueous NaHCO_3_ (20 mL) as well as with saturated aqueous NaCl (20 mL), dried over MgSO_4_, filtered, and concentrated under reduced pressure. The crude product was purified by flash column chromatography (0–15 % ethyl acetate in *n*‐heptane) to give desired product **7** (0.034 g, 0.077 mmol, 26 %) as colorless oil and the unexpected product **23** (0.046 g, 0.10 mmol, 35 %) as slightly yellow oil. ^**1**^ **H‐NMR** (CDCl_3_, 400 MHz): δ=8.76 (s, 1H, N‐C*H*), 7.65 (s, 1H, ClC‐C*H*), 5.35 (s, br, 1H, C*H*‐OTIPS), 5.07 (m, 1H, Me_2_C=C*H*), 3.79 (d, 1H, *J*=1.5 Hz, epoxy‐C*H)*, 2.89 (dd, 1H, *J*=15.4, 8.1 Hz, Me_2_C=CH‐C*H*_2_), 2.63 (dd, 1H, *J*=15.7, 7.3 Hz, Me_2_C=CH‐C*H*_2_), 1.74 (s, 3H, *E*‐C*H*_3_), 1.67 (s, 3H, *Z*‐C*H*_3_), 1.34–1.14 (m, 21H, TIPS‐*H*); ^**13**^ **C‐NMR** (CDCl_3_, 100 MHz): δ=193.5 (*C=*O), 151.9 (Cl–*C*), 149.9 (N‐*C*H), 138.1 (Ar‐*C*_q_‐CO), 137.2 (*C*_q_Me_2_), 132.5 (N‐CH‐*C*_q_), 120.2 (ClC‐*C*H*)*, 116.0 (*C*H=CMe_2_), 66.3 (*C*H‐OTIPS), 62.2 (epoxy‐*C*_q_), 59.0 (epoxy‐*C*H), 25.9 (Me_2_C=CH‐*C*H_2_), 25.9 (*E*‐*C*H_3_), 18.2 (*Z*‐*C*H_3_), 18.3/18.2, (CH(*C*H_3_)_2_), 12.8 (*C*H(CH_3_)_2_); **HRMS (ESI)** m/z calcd. for C_23_H_35_ClNO_3_Si: 436.2069 (M+H)^+^; found: 436.2066 (M+H)^+^; **R_f_** (*n*‐heptane/ethyl acetate 4 : 1): 0.60; **Specific rotation** ${\left[\alpha \right]}_{D}^{22.7}$ =+9.1 (c=0.12; CHCl_3_).

**(1*R*,4*R*,5*R*)‐4‐(6‐Chloro‐4‐(triisopropylsilyl)pyridin‐3‐yl)‐1‐(3‐methylbut‐2‐en‐1‐yl)‐3,6‐dioxabicyclo[3.1.0]hexan‐2‐ imine (23)**: ^**1**^ **H‐NMR** (CDCl_3_, 400 MHz): 8.13 (s, 1H, N‐C*H*), 7.45 (s, 1H, ClC‐C*H*), 5.35 (s, 1H, pyridine‐C*H*O), 5.16 (m, 1H, Me_2_C=C*H*), 3.78 (s, 1H, epoxy‐C*H*), 3.13–2.92 (m, 1H, Me_2_C=CH‐C*H*_2_), 2.74 (dd, 1H, *J=*15.6, 7.2 Hz, Me_2_C=CH‐C*H*_2_), 1.74 (s, 3H, *E*‐C*H*_3_), 1.66 (s, 3H, *Z*‐C*H*_3_), 1.46 (m, 3H, *C*H(CH_3_)_2_), 1.17–1.06 (m, 18H, CH(*C*H_3_)_2_); ^**13**^ **C‐NMR** (CDCl_3_, 100 MHz): δ=165.2 (N*H*), 152.3 (Cl–*C*), 150.4 (*C*_q_‐TIPS), 148.5 (N‐*C*H), 137.3 (*C*_q_Me_2_), 136.4 (N‐CH‐*C*_q_), 131.1 (ClC‐*C*H), 115.8 (*C*H=CMe_2_), 78.5 (pyridine‐*C*HO), 63.5 (epoxy‐*C*_q_), 63.4 (epoxy‐*C*H), 25.9 (*E*‐*C*H_3_), 24.9 (Me_2_C=CH‐*C*H_2_), 18.9/18.8 (CH(*C*H_3_)_2_), 18.2 (*Z*‐*C*H_3_), 12.5 (*C*H(CH_3_)_2_); **HRMS (ESI)** m/z calcd. for C_23_H_36_ClNO_3_Si: (M+H)^+^, 436.2069; found: 436.2072 (M+H)^+^; **R_f_** (*n*‐heptane/ethyl acetate 4 : 1): 0.28.

**(1a*R*,2*S*,7a*R*)‐5‐Chloro‐7 a‐(3‐methylbut‐2‐en‐1‐yl)‐2‐((triisopropylsilyl)oxy)‐1 a,7 a‐dihydrooxireno[2,3‐g] isoquinolin‐7(2*H*)‐one (25): 25** (0.065 g, 0.15 mmol, 81 %) was synthesized in analogous manner to **7** starting from **22** (0.095 g, 0.18 mmol) and was obtained as colorless oil.^[27]^ ^**1**^ **H‐NMR** (CDCl_3_, 400 MHz): δ=8.47 (s, 1H, N‐C*H*), 7.72 (s, 1H, ClC‐C*H*), 5.42 (d, 1H, *J*=2.7 Hz, C*H*‐OTIPS), 5.10 (m, 1H, Me_2_C=C*H*), 3.82 (d, 1H, *J*=2.7 Hz, epoxy‐C*H)*, 2.97 (dd, 1H, *J*=15.3, 7.9 Hz, Me_2_C=CH‐C*H*_2_), 2.55 (dd, 1H, *J*=15.5, 6.8 Hz, Me_2_C=CH‐C*H*_2_), 1.72 (s, 3H, *E*‐C*H*_3_), 1.68 (s, 3H, *Z*‐C*H*_3_), 1.16–0.96 (m, 21H, TIPS‐*H*); ^**13**^ **C‐NMR** (CDCl_3_, 100 MHz): δ=193.6 (*C=*O), 152.7 (Cl–*C*), 151.2 (N‐*C*H), 139.4 (Ar‐*C*_q_‐CO), 136.6 (*C*_q_Me_2_), 132.7 (N‐CH‐*C*_q_), 121.3 (ClC‐*C*H*)*, 116.2 (*C*H=CMe_2_), 65.5 (*C*H‐OTIPS), 60.9 (epoxy‐*C*_q_), 60.4 (epoxy‐*C*H), 26.4 (Me_2_C=CH‐*C*H_2_), 26.0 (*E*‐*C*H_3_), 18.2 (*Z*‐*C*H_3_), 18.1/18.0 (CH(*C*H_3_)_2_), 12.6 (*C*H(CH_3_)_2_); **HRMS (ESI)** m/z calcd. for C_23_H_35_ClNO_3_Si: 436.2069 (M+H)^+^; found: 436.2070 (M+H)^+^; **R_f_** (*n*‐heptane/ethyl acetate 4 : 1): 0.59; **Specific rotation** ${\left[\alpha \right]}_{D}^{22.7}$ =+64.0 (c=0.47; CHCl_3_).

***Ent*** **‐7** (0.020 g, 0.046 mmol, 23 %) and ***ent*** **‐25** (0.088 g, 0.20 mmol, 81 %) were synthesized in analogous manner to **7** and **25** starting from ***ent*** **‐8** (0.100 g, 0.195 mmol) and ***ent*** **‐22** (0.128 g, 0.249 mmol). The low yield of ***ent*** **‐7** was caused by the formation of ***ent*** **‐23** (0.023 g, 0.052 mmol, 27 %). The NMR data are identical to the ones reported for **7**, **25**, and **23**. For ***ent*** **‐7**: **HRMS (ESI)** m/z calcd. for C_23_H_35_ClNO_3_Si: 436.2069 (M+H)^+^; found: 436.2070 (M+H)^+^; **R_f_** (*n*‐heptane/ethyl acetate 4 : 1): 0.60; **Specific rotation** ${\left[\alpha \right]}_{D}^{22.0}$ =−126.6 (c=0.96; CHCl_3_). For ***ent*** **‐25**: **HRMS (ESI)** m/z calcd. for C_23_H_35_ClNO_3_Si: 436.2069 (M+H)^+^; found: 436.2073 (M+H)^+^; **R_f_** (*n*‐heptane/ethyl acetate 4 : 1): 0.59; **Specific rotation** ${\left[\alpha \right]}_{D}^{21.4}$ =−121.9 (c=0.97; CHCl_3_). For ***ent*** **‐23**: **HRMS (ESI)** m/z calcd. for C_23_H_36_ClNO_3_Si: (M+H)^+^, 436.2069; found: 436.2070 (M+H)^+^; **R_f_** (*n*‐heptane/ethyl acetate 4 : 1): 0.28.

**(1a*R*,2*R*,7a*R*)‐7 a‐(3‐Methylbut‐2‐en‐1‐yl)‐5‐((*E*)‐pent‐1‐en‐1‐yl)‐2‐((triisopropylsilyl)oxy)‐1 a,7 a‐dihydrooxireno[2,3‐g]isoquinolin‐7(2*H*)‐one (24)**: The reaction was carried out under argon atmosphere. **7** (0.017 g, 0.039 mmol, 1.00 eq.) was dissolved in 1,4‐dioxane/H_2_O 8 : 1 (5.00 mL/0.625 mL). *trans*‐1‐Penten‐1‐ylboronic acid pinacol ester (0.018 g, 0.16 mmol, 4.00 eq.) as well as Cs_2_CO_3_ (0.038 g, 0.12 mmol, 3.00 eq.) were added and the solution was degassed by bubbling a stream of argon through it for 15 min. Then APhos Pd G3 (0.001 g, 0.002 mmol, 0.10 eq.) was added. The resulting mixture was stirred for 2 h 30 min at 100 °C,^[28]^ then the reaction mixture was allowed to get to room temperature, diluted with saturated aqueous NH_4_Cl (15 mL) and extracted with ethyl acetate (2×30 mL). The combined organic layers were washed with saturated aqueous NaCl (20 mL), dried over MgSO_4_, filtered, and concentrated under reduced pressure. The crude product was purified by flash column chromatography (0–15 % ethyl acetate in *n*‐heptane) to give **24** (0.009 g, 0.02 mmol, 51 %) as colorless oil. ^**1**^ **H‐NMR** (CDCl_3_, 400 MHz): δ=8.89 (s, 1H, N‐C*H*), 7.56 (s, 1H, pentenyl‐C_q_‐C*H*), 6.79 (dt, 1H, *J*=15.6, 7.1 Hz, CH_2_‐C*H*=CH), 6.52 (dt, 1H, *J*=15.5, 1.3 Hz, CH_2_‐CH=C*H*), 5.36 (s, br, 1H, C*H*‐OTIPS), 5.09 (m, 1H, Me_2_C=C*H*), 3.77 (d, 1H, *J*=1.6 Hz, epoxy‐C*H)*, 2.89 (dd, 1H, *J*=15.3, 8.0 Hz, Me_2_C=CH‐C*H*_2_), 2.63 (dd, 1H, *J*=15.3, 7.0 Hz, Me_2_C=CH‐C*H*_2_), 2.25 (m, 2H, C*H*_2_‐CH=CH), 1.73 (s, 3H, *E*‐C*H*_3_), 1.67 (s, 3H, *Z*‐C*H*_3_), 1.58–1.50 (m, 2H, CH_3_‐C*H*_2_), 1.34–1.09 (m, 21H, TIPS‐*H*), 0.96 (t, 3H, *J=*7.4 Hz, C*H*_3_‐CH_2_); ^**13**^ **C‐NMR** (CDCl_3_, 100 MHz): δ=195.1 (*C=*O), 156.8 (N‐*C*_q_), 149.2 (N‐*C*H), 137.3 (CH_2_‐*C*H=CH), 136.8 (*C*_q_Me_2_), 135.8 (Ar‐*C*_q_‐CO), 131.1 (N‐CH‐*C*_q_), 129.4 (CH_2_‐CH=*C*H), 116.4 (*C*H=CMe_2_), 116.3 (pentenyl‐C_q_ *‐C*H*)*, 66.5 (*C*H‐OTIPS), 62.1 (epoxy‐*C*_q_), 59.1 (epoxy‐*C*H), 35.1 (*C*H_2_‐CH=CH), 26.1 (Me_2_C=CH‐*C*H_2_), 25.9 (*E*‐*C*H_3_), 22.2 (CH_3_‐*C*H_2_), 18.2 (*Z*‐*C*H_3_), 18.3/18.2 (CH(*C*H_3_)_2_), 12.9 (*C*H(CH_3_)_2_), 13.9 (*C*H_3_‐CH_2_); **HRMS (ESI)** m/z calcd. for C_28_H_44_NO_3_Si: 470.3085 (M+H)^+^; found: 470.3079 (M+H)^+^; **R_f_** (*n*‐heptane/ethyl acetate 8 : 1): 0.41; **Specific rotation** ${\left[\alpha \right]}_{D}^{22.0}$ =+146.2 (c=0.47; CHCl_3_).

**(1a*R*,2*S*,7a*R*)‐7 a‐(3‐Methylbut‐2‐en‐1‐yl)‐5‐((*E*)‐pent‐1‐en‐1‐yl)‐2‐((triisopropylsilyl)oxy)‐1 a,7 a‐dihydrooxireno[2,3‐g]isoquinolin‐7(2*H*)‐one (26): 26** (0.102 g, 0.217 mmol, 84 %) was synthesized in analogous manner to **24** starting from **25** (0.114 g, 0.261 mmol) and was obtained as colorless oil. ^**1**^ **H‐NMR** (CDCl_3_, 600 MHz): δ=8.60 (s, 1H, N‐C*H*), 7.63 (s, 1H, pentenyl‐C_q_‐C*H*), 6.79 (dt, 1H, *J*=15.7, 7.0 Hz, CH_2_‐C*H*=CH), 6.53 (dt, 1H, *J*=15.7, 1.5 Hz, CH_2_‐CH=C*H*), 5.41 (d, 1H, *J*=2.6 Hz, C*H*‐OTIPS), 5.12 (m, 1H, Me_2_C=C*H*), 3.79 (d, 1H, *J*=2.6 Hz, epoxy‐C*H)*, 2.98 (dd, 1H, *J*=15.2, 7.9 Hz, Me_2_C=CH‐C*H*_2_), 2.55 (dd, 1H, *J*=15.4, 6.8 Hz, Me_2_C=CH‐C*H*_2_), 2.26 (m, 2H, C*H*_2_‐CH=CH), 1.72 (s, 3H, *E*‐C*H*_3_), 1.68 (s, 3H, *Z*‐C*H*_3_), 1.53 (m, 2H, CH_3_‐C*H*_2_), 1.07–0.98 (m, 21H, TIPS‐*H*), 0.96 (t, 3H, *J*=7.4 Hz, C*H*_3_‐CH_2_); ^**13**^ **C‐NMR** (CDCl_3_, 100 MHz): δ=195.2 (*C=*O), 157.9 (N‐*C*_q_), 150.9 (N‐*C*H), 137.9 (CH_2_‐*C*H=CH), 137.0 (Ar‐*C*_q_‐CO), 136.3 (*C*_q_Me_2_), 131.3 (N‐CH‐*C*_q_), 129.5 (CH_2_‐CH=*C*H), 116.9 (pentenyl‐C_q_ *‐C*H*)*, 116.7 (*C*H=CMe_2_), 65.8 (*C*H‐OTIPS), 60.9 (epoxy‐*C*_q_), 60.5 (epoxy‐*C*H), 35.1 (*C*H_2_‐CH=CH), 26.6 (Me_2_C=CH‐*C*H_2_), 26.0 (*E*‐*C*H_3_), 22.2 (CH_3_‐*C*H_2_), 18.2 (*Z*‐*C*H_3_), 18.2/18.1 (CH(*C*H_3_)_2_), 12.7 (*C*H(CH_3_)_2_), 13.9 (*C*H_3_‐CH_2_); **HRMS (ESI)** m/z calcd. for C_28_H_44_NO_3_Si: (M+H)^+^, 470.3085; found: 470.3127 (M+H)^+^; **R_f_** (*n*‐heptane/ethyl acetate 10 : 1): 0.27; **Specific rotation** ${\left[\alpha \right]}_{D}^{22.0}$ =+179.3 (c=0.33; CHCl_3_).

***Ent*** **‐24** (0.012 g, 0.026 mmol, 54 %) and ***ent*** **‐26** (0.066 g, 0.141 mmol, 70 %) were synthesized in analogous manner to **24** and **26** starting from ***ent*** **‐7** (0.021 g, 0.047 mmol) and ***ent*** **‐25** (0.088 g, 0.20 mmol). The NMR data are identical to the ones reported for **24** and **26**. For ***ent*** **‐24**: **HRMS (ESI)** m/z calcd. for C_28_H_44_NO_3_Si: 470.3085 (M+H)^+^; found: 470.3086 (M+H)^+^; **R_f_** (*n*‐heptane/ethyl acetate 8 : 1): 0.41; **Specific rotation** ${\left[\alpha \right]}_{D}^{21.3}$ =−130.2 (c=0.57; CHCl_3_). For ***ent*** **‐26**: **HRMS (ESI)** m/z calcd. for C_28_H_44_NO_3_Si: 470.3085 (M+H)^+^; found: 470.3097 (M+H)^+^; **R_f_** (*n*‐heptane/ethyl acetate 10 : 1): 0.27; **Specific rotation** ${\left[\alpha \right]}_{D}^{22.0}$ =−119.1 (c=0.98; CHCl_3_).

**(1a*R*,2*R*,7a*R*)‐2‐Hydroxy‐7 a‐(3‐methylbut‐2‐en‐1‐yl)‐5‐((*E*)‐pent‐1‐en‐1‐yl)‐1 a,7 a‐dihydrooxireno[2,3‐g]isoquinolin‐7(2*H*)‐one (4)**: *Condition 1*: In a 15 mL falcon tube, TBAF (1 M in THF, 0.030 mL, 0.030 mmol, 1.20 eq.), followed by glacial acetic acid (0.001 mL, 0.03 mmol, 1.00 eq.) were added to a solution of **24** (0.012 g, 0.025 mmol, 1.00 eq.) in THF (1.5 mL). After 1 h 30 min reaction time, the TLC showed complete conversion of the starting material. The reaction mixture was loaded on silica and purified *via* flash column chromatography (*n*‐heptane/ ethyl acetate 3 : 1, 5 g silica). Product **4** (0.005 g, 0.02 mmol, 60 %) was obtained as colorless solid.

*Condition 2 (For synthesis and structure of **SI1** see Supporting Information)*: To a solution of **SI1** (0.027 g, 0.077 mmol, in THF/H_2_O (5 : 1, 4.0 mL/0.8 mL) LiOH ⋅ H_2_O (0.007 g, 0.2 mmol, 2.00 eq.) was added. The TLC showed a complete conversion after 1 h 30 min reaction time. Saturated aqueous NaHCO_3_ (20 mL) was added. The mixture was extracted with ethyl acetate (2×30 mL) and the combined organic layers were washed with saturated aqueous NaCl (20 mL), dried over MgSO_4_, filtered, and concentrated under reduced pressure. The crude product was purified by flash column chromatography (0–75 % ethyl acetate in *n*‐heptane) to give **4** (0.012 g, 0.040 mmol, 51 %) as a colorless solid. ^**1**^ **H‐NMR** (MeOD, 600 MHz): δ=8.75 (s, 1H, N‐C*H*), 7.68 (s, 1H, pentenyl‐C_q_‐C*H*), 6.79 (dt, 1H, *J*=15.8, 7.1 Hz, CH_2_‐C*H*=CH), 6.56 (dt, 1H, *J*=15.8, 1.5 Hz, CH_2_‐CH=C*H*), 5.18 (s, br, 1H, C*H*‐OH), 5.14 (m, 1H, Me_2_C=C*H*), 3.83 (d, 1H, *J*=1.9 Hz, epoxy‐C*H)*, 2.82 (dd, 1H, *J*=15.2, 8.0 Hz, Me_2_C=CH‐C*H*_2_), 2.65 (dd, 1H, *J*=15.2, 6.9 Hz, Me_2_C=CH‐C*H*_2_), 2.27 (m, 2H, C*H*_2_‐CH=CH), 1.74 (s, 3H, *E*‐C*H*_3_), 1.70 (s, 3H, *Z*‐C*H*_3_), 1.56 (m, 2H, CH_3_‐C*H*_2_), 0.99 (t, 3H, *J*=7.4 Hz, C*H*_3_‐CH_2_); ^**13**^ **C‐NMR** (MeOD, 150 MHz): δ=195.4 (*C=*O), 157.5 (N‐*C*_q_), 149.9 (N‐*C*H), 138.5 (CH_2_‐*C*H=CH), 137.7 (Ar‐*C*_q_‐CO), 137.0 (*C*_q_Me_2_), 133.8 (N‐CH‐*C*_q_), 130.0 (CH_2_‐CH=*C*H), 117.8 (*C*H=CMe_2_), 117.1 (pentenyl‐C_q_ *‐C*H*)*, 65.3 (*C*H‐OH), 63.1 (epoxy‐*C*_q_), 61.1 (epoxy‐*C*H), 36.0 (*C*H_2_‐CH=CH), 27.2 (Me_2_C=CH‐*C*H_2_), 26.0 (*E*‐*C*H_3_), 23.2 (CH_3_‐*C*H_2_), 18.1 (*Z*‐*C*H_3_), 14.1 (*C*H_3_‐CH_2_); ^**1**^ **H‐NMR** (CDCl_3_, 400 MHz): δ=8.90 (s, 1H, N‐C*H*), 7.63 (s, 1H, pentenyl‐C_q_‐C*H*), 6.83 (dt, 1H, *J*=15.8, 7.1 Hz, CH_2_‐C*H*=CH), 6.55 (dt, 1H, *J*=15.8, 1.5 Hz, CH_2_‐CH=C*H*), 5.18 (s, br, 1H, C*H*‐OH), 5.08 (m, 1H, Me_2_C=C*H*), 3.88 (d, 1H, *J*=2.1 Hz, epoxy‐C*H)*, 2.91 (dd, 1H, *J*=15.3, 8.0 Hz, Me_2_C=CH‐C*H*_2_), 2.70 (s, br, 1H, O*H*), 2.62 (dd, 1H, *J*=15.4, 6.9 Hz, Me_2_C=CH‐C*H*_2_), 2.26 (m, 2H, C*H*_2_‐CH=CH), 1.73 (s, 3H, *E*‐C*H*_3_), 1.38 (s, 3H, *Z*‐C*H*_3_), 1.54 (m, 2H, CH_3_‐C*H*_2_), 0.96 (t, 3H, *J=*7.4 Hz, C*H*_3_‐CH_2_); ^**13**^ **C‐NMR** (CDCl_3_, 100 MHz): δ=193.9 (*C=*O), 156.9 (N‐*C*_q_), 149.4 (N‐*C*H), 138.4 (CH_2_‐*C*H=CH), 137.0 (*C*_q_Me_2_), 135.8 (Ar‐*C*_q_‐CO), 131.0 (N‐CH‐*C*_q_), 128.8 (CH_2_‐CH=*C*H), 116.5 (pentenyl‐C_q_ *‐C*H*)*, 116.0 (*C*H=CMe_2_), 65.0 (*C*H‐OH), 62.8 (epoxy‐*C*_q_), 59.7 (epoxy‐*C*H), 35.1 (*C*H_2_‐CH=CH), 26.5 (Me_2_C=CH‐*C*H_2_), 26.0 (*E*‐*C*H_3_), 22.2 (CH_3_‐*C*H_2_), 18.2 (*Z*‐*C*H_3_), 13.9 (*C*H_3_‐CH_2_); **HRMS (ESI)** m/z calcd. for C_19_H_24_NO_3_: 314.1751 (M+H)^+^; found: 314.1751 (M+H)^+^; **R_f_** (*n*‐heptane/ethyl acetate 1 : 1): 0.37; **chiral HPLC** (Chiralpak IC; hexane:isopropyl alcohol 85 : 15): **4** (*t*_R_=13.8 min) : ***ent*** **‐4** (*t*_R_=11.1 min) 96.6 : 3.4 (93 % *ee*); **Specific rotation** ${\left[\alpha \right]}_{D}^{24.2}$ =+159.3 (c=0.27; CHCl_3_).

**(1a*R*,2*S*,7a*R*)‐2‐Hydroxy‐7 a‐(3‐methylbut‐2‐en‐1‐yl)‐5‐((*E*)‐pent‐1‐en‐1‐yl)‐1 a,7 a‐dihydrooxireno[2,3‐g]isoquinolin‐7(2*H*)‐one (27): 27** (0.044 g, 0.14 mmol, 65 %) was synthesized in analogous manner to **4** utilizing condition 1 starting from **26** (0.102 g, 0.217 mmol) and was obtained as colorless oil. ^**1**^ **H‐NMR** (MeOD, 500 MHz): δ=8.61 (s, 1H, N‐C*H*), 7.72 (s, 1H, pentenyl‐C_q_‐C*H*), 6.82 (dt, 1H, *J*=15.8, 7.1 Hz, CH_2_‐C*H*=CH), 6.56 (dt, 1H, *J*=15.8, 1.5 Hz, CH_2_‐CH=C*H*), 5.21 (d, 1H, *J*=2.3 Hz, C*H*‐OH), 5.15 (m, 1H, Me_2_C=C*H*), 3.82 (d, 1H, *J*=2.3 Hz, epoxy‐C*H)*, 2.84 (dd, 1H, *J*=15.2, 8.0 Hz, Me_2_C=CH‐C*H*_2_), 2.63 (dd, 1H, *J*=15.2, 6.9 Hz, Me_2_C=CH‐C*H*_2_), 2.27 (m, 2H, C*H*_2_‐CH=CH), 1.73 (s, 3H, *E*‐C*H*_3_), 1.69 (s, 3H, *Z*‐C*H*_3_), 1.55 (m, 2H, CH_3_‐C*H*_2_), 0.98 (t, 3H, *J=*7.4 Hz, C*H*_3_‐CH_2_); ^**13**^ **C‐NMR** (MeOD, 125 MHz): δ=195.5 (*C=*O), 158.5 (N‐*C*_q_), 152.5 (N‐*C*H), 139.0 (CH_2_‐*C*H=CH), 138.2 (Ar‐*C*_q_‐CO), 137.0 (*C*_q_Me_2_), 133.6 (N‐CH‐*C*_q_), 130.1 (CH_2_‐CH=*C*H), 117.8 (*C*H=CMe_2_), 117.5 (pentenyl‐C_q_ *‐C*H*)*, 64.9 (*C*H‐OH), 62.1 (epoxy‐*C*_q_), 61.9 (epoxy‐*C*H), 36.0 (*C*H_2_‐CH=CH), 27.5 (Me_2_C=CH‐*C*H_2_), 26.0 (*E*‐*C*H_3_), 23.2 (CH_3_‐*C*H_2_), 18.1 (*Z*‐*C*H_3_), 14.1 (*C*H_3_‐CH_2_); ^**1**^ **H‐NMR** (CDCl_3_, 400 MHz): δ=8.53 (s, 1H, N‐C*H*), 7.62 (s, 1H, pentenyl‐C_q_‐C*H*), 6.76 (dt, 1H, *J*=15.7, 7.0 Hz, CH_2_‐C*H*=CH), 6.46 (dt, 1H, *J*=15.7, 1.6 Hz, CH_2_‐CH=C*H*), 5.27 (d, 1H, *J*=2.3 Hz, C*H*‐OH), 5.09 (m, 1H, Me_2_C=C*H*), 3.85 (d, 1H, *J*=2.3 Hz, epoxy‐C*H*), 3.18 (s, br, 1H, O*H*), 2.84 (dd, 1H, *J*=15.5, 7.9 Hz, Me_2_C=CH‐C*H*_2_), 2.64 (dd, 1H, *J*=15.4, 6.7 Hz, Me_2_C=CH‐C*H*_2_), 2.24 (m, 2H, C*H*_2_‐CH=CH), 1.73 (s, 3H, *E*‐C*H*_3_), 1.68 (s, 3H, *Z*‐C*H*_3_), 1.53 (m, 2H, CH_3_‐C*H*_2_), 0.96 (t, 3H, *J=*7.4 Hz, C*H*_3_‐CH_2_); ^**13**^ **C‐NMR** (CDCl_3_, 100 MHz): δ=194.5 (*C=*O), 157.9 (N‐*C*_q_), 151.1 (N‐*C*H), 138.8 (CH_2_‐*C*H=CH), 136.8 (Ar‐*C*_q_‐CO), 136.6 (*C*_q_Me_2_), 131.1 (N‐CH‐*C*_q_), 128.9 (CH_2_‐CH=*C*H), 116.9 (pentenyl‐C_q_ *‐C*H*)*, 116.1 (*C*H=CMe_2_), 64.6 (*C*H‐OH), 61.1 (epoxy‐*C*_q_), 60.4 (epoxy‐*C*H), 35.1 (*C*H_2_‐CH=CH), 26.5 (Me_2_C=CH‐*C*H_2_), 26.0 (*E*‐*C*H_3_), 22.2 (CH_3_‐*C*H_2_), 18.2 (*Z*‐*C*H_3_), 13.9 (*C*H_3_‐CH_2_); **HRMS (ESI)** m/z calcd. for C_19_H_24_NO_3_: 314.1751 (M+H)^+^; found: 314.1755 (M+H)^+^; **R_f_** (*n*‐heptane/ethyl acetate 1 : 1): 0.44; **chiral HPLC** (Chiralpak IC; hexane:isopropyl alcohol 85 : 15): **27** (*t*_R_=6.6 min) : ***ent*** **‐27** (*t*_R_=4.9 min) 96.5 : 3.5 (93 % *ee*); **Specific rotation** ${\left[\alpha \right]}_{D}^{22.0}$ =+179.3 (c=0.33; CHCl_3_).

***Ent*** **‐4** (0.005 g, 0.01 mmol, 59 %) and ***ent*** **‐27** (0.032 g, 0.10 mmol, 74 %) were synthesized in analogous manner to **4** and **27** utilizing condition 1 and starting from ***ent*** **‐24** (0.012 g, 0.026 mmol) and ***ent*** **‐26** (0.066 g, 0.14 mmol). The NMR data are identical to the ones reported for **4** and **27**. For ***ent*** **‐4**: **HRMS (ESI)** m/z calcd. for C_19_H_24_NO_3_: 314.1751 (M+H)^+^; found: 314.1750 (M+H)^+^; **R_f_** (*n*‐heptane/ethyl acetate 1 : 1): 0.37; **chiral HPLC** (Chiralpak IC; hexane:isopropyl alcohol 85 : 15): ***ent*** **‐4** (*t*_R_=11.1 min): **4** (*t*_R_=13.8 min) 98.5 : 1.5 (97 % *ee*); **Specific rotation** ${\left[\alpha \right]}_{D}^{25.1}$ =−206.0 (c=0.22; CHCl_3_). For ***ent*** **‐27**: **HRMS (ESI)** m/z calcd. for C_19_H_24_NO_3_: 314.1751 (M+H)^+^; found: 314.1748 (M+H)^+^; **R_f_** (*n*‐heptane/ethyl acetate 1 : 1): 0.44; **chiral HPLC** (Chiralpak IC; hexane:isopropyl alcohol 85 : 15): ***ent*** **‐27** (*t*_R_=4.9 min) : **27** (*t*_R_=6.6 min) 96.5 : 3.5 (93 % *ee*); **Specific rotation** ${\left[\alpha \right]}_{D}^{21.3}$ =−160.3 (c=1.20; CHCl_3_).

## Conflict of interest

The authors declare no conflict of interest.

## Supporting information

As a service to our authors and readers, this journal provides supporting information supplied by the authors. Such materials are peer reviewed and may be re‐organized for online delivery, but are not copy‐edited or typeset. Technical support issues arising from supporting information (other than missing files) should be addressed to the authors.

Supporting Information
