# Supplementary material for: The Discovery and Structure‐Activity Evaluation of (+)‐Floyocidin B and Synthetic Analogs
Source: ChemMedChem. 2021 Nov 17;17(6):e202100644. doi: 10.1002/cmdc.202100644 (PMC9298916; doi:10.1002/cmdc.202100644)
Supplement: Supplementary file 1 — Supporting Information [file CMDC-17-0-s001.pdf]

ChemMedChem

Supporting Information

The Discovery and Structure-Activity Evaluation of (+)-Floyocidin B and Synthetic Analogs

Yolanda Kleiner, Christoph Pöverlein, Jannike Klädtke, Michael Kurz, Henrik F. König, Jonathan Becker, Sanja Mihajlovic, Florian Zubeil, Michael Marner, Andreas Vilcinskas, Till F. Schäberle, Peter Hammann, Sören M. M. Schuler,* and Armin Bauer*

Table of Contents

1. Experimental Procedures 2

1.1. Synthetic procedures and compound characterization 2

1.2. Confirmation of the absolute stereochemistry of (+)-floyocidin B (4) by chiral HPLC 14

2. Biological and medicinal-chemical profiling 15

2.3. Antimicrobial activities 15

2.4. Assay descriptions for the profiling of all four stereoisomers of (+)-floyocidin B and (−)-avicennone C, the avicennone C-floyocidin B hybrids 35, *ent*-35, 36 and *ent*-36 as well as the natural product (+)-floyocidin A. 16

2.4.1. Determination of log$D_{7.4}$ 16

2.4.2. Microsomal stability 16

2.4.3. Cellular permeability assay utilizing Caco2 cell line 16

2.4.4. Cytotoxicity on THP1 and HepG2 cells 17

3. Biosynthetic hypothesis of floyocidins 18

4. Crystallographic data collection and refinement details 20

5. NMR spectra 22

6. Determination of the enantiomeric excesses (*ee*) 65

1. Experimental Procedures

For the avicennone C-floyocidin B hybrids, the synthetic steps are described in details for the series with the same absolute stereochemistry of the epoxide as (+)-floyocidin B (**4**) and the corresponding stereoisomers are listed afterwards.

1.1. Synthetic procedures and compound characterization

(1a*R*,2*R*,7a*R*)-7a-(3-methylbut-2-en-1-yl)-7-oxo-5-((*E*)-pent-1-en-1-yl)-1a,2,7,7a-tetrahydrooxireno[2,3-g] isoquinolin-2-yl acetate (SI1):

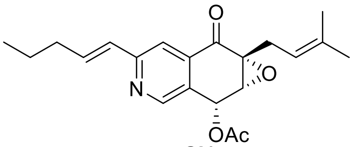

SI1

Alcohol **27** (0.016 g, 0.051 mmol, 1.00 eq.) was dissolved in anhydrous THF (5 mL). PPh_3 (0.040 g, 0.15 mmol, 3.00 eq.) and HOAc (0.018 mL, 0.31 mmol, 6.00 eq.) were added. Then DIAD (0.030 mL, 0.15 mmol, 3.00 eq.) was added at 0 °C. The reaction solution was stirred at room temperature for 1 h 10 min until TLC showed complete conversion of the starting material. The reaction mixture was loaded on silica and was then purified *via* flash column chromatography (0–40% ethyl acetate in *n*-heptane) to give **SI1** (0.015 g; 0.043 mmol; 84%) as colorless oil.

^{1}H-NMR ($CDCl_3$, 400 MHz): δ = 8.54 (s, 1H, N-C*H*), 7.64 (s, 1H, pentenyl-C_q-C*H*), 6.82 (dt, 1H, *J* = 15.7, 7.0 Hz, CH_2-C*H*=CH), 6.53 (dt, 1H, *J* = 15.7, 1.5 Hz, CH_2-CH=C*H*), 6.36 (s, br, 1H, C*H*-OAc), 5.06 (m, 1H, Me_2C=C*H*), 3.89 (d, 1H, *J* = 2.0 Hz, epoxy-C*H*), 2.91 (dd, 1H, *J* = 15.5, 8.0 Hz, Me_2C=CH-CH_2), 2.63 (dd, 1H, *J* = 15.5, 7.0 Hz, Me_2C=CH-CH_2), 2.32 (s, 3H, CO-CH_3), 2.26 (m, 2H, CH_2-CH=CH), 1.73 (s, 3H, *E*-CH_3), 1.68 (s, 3H, *Z*-CH_3), 1.53 (m, 2H, CH_3-CH_2), 0.96 (t, 3H, *J* = 7.3 Hz, CH_3-CH_2); **^{13}C-NMR** ($CDCl_3$, 100 MHz): δ = 193.5 (*C*=O), 170.9 (*C*O-CH_3), 157.4 (N-C_q), 148.5 (N-*C*H), 138.2 (CH_2-*C*H=CH), 137.2 ($C_q$$Me_2$), 136.2 (Ar-$C_q$-CO), 129.1 ($CH_2$-CH=*C*H), 126.8 (N-CH-$C_q$), 116.6 (pentenyl-$C_q$-*C*H), 115.7 (*C*H=C$Me_2$), 66.3 (*C*H-OAc), 61.8 (epoxy-C_q), 56.5 (epoxy-*C*H), 35.1 (*C*H_2-CH=CH), 26.2 (Me_2C=CH-*C*H_2), 26.0 (*E*-*C*H_3), 22.2 (CH_3-*C*H_2), 21.1 (CO-*C*H_3), 18.2 (*Z*-*C*H_3), 13.9 (*C*H_3-CH_2); **HRMS (ESI)** m/z calcd. for $C_{21}H_{26}NO_4$: 356.1856 $(M+H)^+$; found: 356.1853 $(M+H)^+$; **R_f** (*n*-heptane/ethyl acetate 2:1): 0.37; **Specific rotation** $[\alpha]_D^{25.6}$ = +193.8 (c = 0.61; $CHCl_3$).

(3-Bromo-4-iodophenyl)methanol (29):

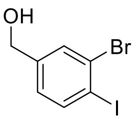

29

3-Bromo-4-iodobenzoic acid (4.98 g, 15.2 mmol, 1.00 eq.) was suspended in anhydrous DCM (10 mL) and $(COCl)_2$ (1.90 mL, 22.2 mmol, 1.46 eq.) as well as two drops of anhydrous DMF were added. The resulting mixture was stirred at room temperature for 1 h 45 min and was then concentrated *in vacuo*. The residue was dissolved in anhydrous THF (10 mL) and $LiBH_4$ (0.334 g, 15.3 mmol, 1.00 eq.) was added. After 3 h 30 min stirring at room temperature, saturated aqueous NH_4Cl (20 mL) was added. The mixture was extracted with ethyl acetate (2 x 100 mL) and the combined organic layers were washed with saturated aqueous NaCl (100 mL), dried over $MgSO_4$, filtered, and concentrated under reduced pressure. The crude product was purified by flash column chromatography (*n*-heptane/ethyl acetate 10:1 to 2:1) to give **29** (4.42 g, 14.1 mmol, 93% over two steps) as colorless solid.

^{1}H-NMR ($CDCl_3$, 400 MHz): δ = 7.82 (d, 1H, *J* = 8.1 Hz, IC_q-C*H*), 7.63 (d, 1H, *J* = 1.9 Hz, BrC_q-C*H*), 6.98 (dd, 1H, *J* = 8.1, 1.9 Hz, CH-C*H*-C_q-CH_2), 4.62 (s, 2H, CH_2), 1.88 (s, br, 1H, O*H*); **^{13}C-NMR** ($CDCl_3$, 100 MHz): δ = 142.9 (BrC_q), 140.4 (IC_q-*C*H), 131.1 (BrC_q-*C*H), 130.0 (CH_2-C_q), 126.9 (CH-*C*H-C_q-CH_2), 99.7 (IC_q), 64.0 (*C*H_2); **HRMS (ESI)** m/z calcd. for C_7H_5BrI: 294.8614 $(M-H_2O+H)^+$; found: 294.8611 $(M-H_2O+H)^+$; **R_f** (*n*-heptane/ethyl acetate 4:1): 0.17.

3-Bromo-4-iodobenzaldehyde (30):

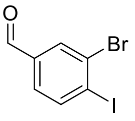

30

The reaction was carried out in moisture-free glassware under argon atmosphere. To a solution of $(COCl)_2$ (1.70 mL, 22.0 mmol, 1.80 eq.) in anhydrous DCM (90 mL), anhydrous DMSO (2.70 mL, 38.0 mmol, 3.20 eq.) was added carefully at −78 °C. After stirring for 15 min, alcohol **29** (3.73 g, 11.9 mmol, 1.00 eq.) dissolved in anhydrous DCM (72 mL) was added. The reaction mixture was stirred for 1 h 30 min at −78 °C. Then DIPEA (10.4 mL, 59.7 mmol, 5.01 eq.) was added and the solution was stirred for 30 min at −78 °C and for further 30 min without cooling bath. A mixture of toluene/saturated aqueous NH_4Cl (3:1, 240 mL) was added to the reaction mixture. The aqueous layer was separated and the organic layer was washed again with saturated aqueous NH_4Cl (3 x 80 mL), with saturated aqueous $NaHCO_3$ (80 mL), and with saturated aqueous NaCl (80 mL). The organic layer was dried over $MgSO_4$, filtered, and concentrated under reduced pressure to yield aldehyde **30** (11.9 mmol, quant.) as beige solid which was used in the next stage without further purification. For full characterization a small portion of the crude product was purified by flash column chromatography (0–5% ethyl acetate in *n*-heptane).

^{1}H-NMR ($CDCl_3$, 400 MHz): δ = 9.91 (s, 1H, C*H*O), 8.10–8.03 (m, 2H, IC_q-C*H* and BrC_q-C*H*), 7.47 (dd, 1H, *J* = 8.1, 1.9 Hz, CH-C*H*-C_q-CHO); **^{13}C-NMR** ($CDCl_3$, 100 MHz): δ = 190.2 (*C*HO), 141.3 (IC_q-*C*H), 137.5 (Ar-C_q), 133.3 (BrC_q-C*H*), 131.2 (Ar-C_q), 128.5 (CH-*C*H-C_q-CH_2), 109.9 (IC_q); **HRMS (ESI)** m/z calcd. for C_7H_5BrIO: 310.8563 $(M+H)^+$; found: 310.8566 $(M+H)^+$; **R_f** (*n*-heptane/ethyl acetate 4:1): 0.50.

5-(Butylsulfonyl)-1-phenyl-1*H*-tetrazole (31):

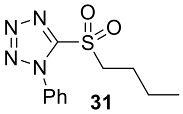

31

1-Phenyl-1*H*-tetrazol-5-thiol (5.00 g, 28.1 mmol, 1.00 eq.) was dissolved in DMF (15 mL). 1-Bromobutane (3.00 mL, 28.1 mmol, 1.00 eq.) and KOH (2.46 g, 43.9 mmol, 1.50 eq.) were added. After stirring at room temperature for 2 h, the TLC showed some remaining starting material, so a second portion of 1-bromobutane (0.600 mL, 5.60 mmol, 0.20 eq.) was added. The reaction mixture was stirred at room temperature for further 3 h, until the TLC indicated a complete conversion. H_2O (50 mL) was added and the mixture was extracted twice with ethyl acetate (150 mL and 100 mL). The combined organic layers were washed with saturated aqueous NaCl (50 mL), dried over $MgSO_4$, filtered, and concentrated under reduced pressure.

The residue was dissolved in DCM (100 mL) and *m*-CPBA (≥ 70%, 21.8 g, 88.3 mmol, 3.04 eq.) was added carefully at 0 °C. The suspension was allowed to stir overnight at room temperature and then saturated aqueous $Na_2S_2O_3$-Lsg. (60 mL) was added. The mixture was extracted with Et_2O (2 x 300 mL) and the combined organic layers were washed with 1 M aqueous NaOH (2 x 100 mL) and saturated aqueous NaCl (150 mL), dried over $MgSO_4$, filtered, and concentrated under reduced pressure to yield **31** (6.92 g, 26.0 mmol, 93% over two steps) as slightly yellow oil, which was used in the next step without further purification.

^{1}H-NMR ($CDCl_3$, 400 MHz): δ = 7.75–7.55 (m, 5H, Ar-*H*), 3.79–3.67 (m, 2H, SO_2-CH_2), 1.99–1.88 (m, 2H, SO_2-CH_2-CH_2), 1.54 (m, 2H, CH_2-CH_3), 0.98 (t, 3H, *J* = 7.4 Hz, CH_3); **^{13}C-NMR** ($CDCl_3$, 100 MHz): δ = 153.6 (SO_2-C_q), 133.2 (Ar-C_q), 131.6 (Ar-*C*H), 129.8 (Ar-*C*H), 125.2 (Ar-*C*H), 55.9 (SO_2-CH_2), 24.0 (SO_2-CH_2-CH_2), 21.6 (*C*H_2-CH_3), 13.5 (*C*H_3); **HRMS (ESI)** m/z calcd. for $C_{11}H_{15}N_4O_2S$: 267.0910 $(M+H)^+$; found: 267.0910 $(M+H)^+$; **R_f** (*n*-heptane/ethyl acetate 4:1): 0.38.

(*E*)-2-Bromo-1-iodo-4-(pent-1-en-1-yl)benzene (32):

The reaction was carried out in moisture-free glassware under argon atmosphere. To a solution of **31** (2.30 g, 8.63 mmol, 1.10 eq.) in anhydrous THF (20 mL) KHMDS (0.7 M in toluene, 13.8 mL, 9.50 mmol, 1.20 eq.) was added dropwise at −55 °C. After 1 h 10 min reaction time, aldehyde **30** (2.44 g, 7.85 mmol, 1.00 eq.) in anhydrous THF (12 mL) was added. The reaction mixture was stirred for 1 h at −55 °C and was then diluted with saturated aqueous NH_4Cl (80 mL). The mixture was extracted with ethyl acetate (2 x 150 mL) and the combined organic layers were washed with saturated aqueous NaCl (100 mL), dried over $MgSO_4$, filtered, and concentrated under reduced pressure. The crude product was purified by flash

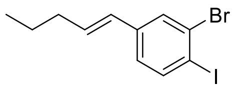

32

column chromatography (*n*-heptane/ethyl acetate 10:1 to 9:1) to give **32** (2.07 g, 5.90 mmol, 75%, *E*/*Z* = 94:6) as colorless oil.

Prior to the described synthesis of the Julia-Kocieński-olefination, a base screening was performed on 0.650 mmol scale to evaluate isolated yield and *E*/*Z*-ratios (see Table S1). KHMDS was chosen due to the observed high *E*-selectivity.

***Table S1.* Screening of reaction conditions.**

	Reaction conditions	Isolated yield	*E*/*Z*-ratio[a]
A	i. **31**, LDA, THF, −55 °C, 1 h 10 min<br>ii. **30**, −55 °C, 1 h	58%	79/21
B	i. **31**, LiHMDS, THF, −55 °C, 1 h 10 min<br>ii. **30**, −55 °C, 1 h	70%	78/22
C	i. **31**, NaHMDS, THF, −55 °C, 1 h 10 min<br>ii. **30**, −55 °C, 1 h	72%	90/10
D	i. **31**, KHMDS, THF, −55 °C, 1 h 10 min<br>ii. **30**, −55 °C, 1 h	60%	96/4

[a] Assignment of the E/Z-ratios was done by integrating the ^{1}H-NMR signals of the alkene protons.

^{1}H-NMR ($CDCl_3$, 400 MHz): δ = <u>*E*-isomer:</u> 7.73 (d, 1H, *J* = 8.2 Hz, IC_q-C*H*), 7.59 (d, 1H, *J* = 2.0 Hz, BrC_q-C*H*), 6.95 (dd, 1H, *J* = 8.3, 2.0 Hz, IC_q-CH-C*H*), 6.31–6.19 (m, 2H, C*H*=C*H*), 2.21–2.14 (m, 2H, CH_2-CH=CH), 1.49 (m, 2H, CH_3-CH_2), 0.95 (t, 3H, *J* = 7.4 Hz, CH_3-CH_2); <u>*Z*-isomer:</u> 7.78 (d, 1H, *J* = 8.2 Hz, IC_q-C*H*), 7.52 (d, 1H, *J* = 2.0 Hz, BrC_q-C*H*), 6.89 (dd, 1H, *J* = 8.2, 2.0 Hz, IC_q-CH-C*H*), 6.31–6.19 (m, 1H, CH_2-CH=C*H*), 5.73 (td, 1H, *J* = 11.7, 7.3 Hz, CH_2-C*H*=CH), 2.30–2.21 (m, 2H, CH_2-CH=CH), 1.49 (m, 2H, CH_3-CH_2), 0.93 (t, 3H, *J* = 7.4 Hz, CH_3-CH_2); **^{13}C-NMR** ($CDCl_3$, 100 MHz): δ = <u>*E*-isomer:</u> 140.2 (IC_q-*C*H), 139.9 (BrC_q), 133.7 (CH_2-*C*H=CH), 130.1 (BrC_q-*C*H), 130.0 (pentenyl-C_q), 127.9 (CH_2-CH=*C*H), 126.1 (IC_q-CH-*C*H), 98.1 (IC_q), 35.2 (*C*H_2-CH=CH), 22.4 (CH_3-*C*H_2), 13.9 (*C*H_3-CH_2); <u>*Z*-isomer:</u> 139.9 (IC_q-*C*H), 139.6 (BrC_q), 135.3 (CH_2-*C*H=CH), 132.9 (BrC_q-*C*H), 129.6 (pentenyl-C_q), 128.9 (IC_q-CH-*C*H), 126.8 (CH_2-CH=*C*H), 98.3 (IC_q), 30.7 (*C*H_2-CH=CH), 23.1 (CH_3-*C*H_2), 13.9 (*C*H_3-CH_2); **HRMS (ESI)** m/z calcd. for $C_{11}H_{13}BrI$: 350.9240 $(M+H)^+$; found: no isonisation was observed; **R_f** (100% *n*-heptane): 0.72.

(*R*)-(2-Bromo-4-((*E*)-pent-1-en-1-yl)phenyl)((2*R*,3*S*)-3-(((*tert*-butyldiphenylsilyl)oxy)methyl)-3-(3-methylbut-2-en-1-yl)oxiran-2-yl)methanol (33) and (*S*)-(2-Bromo-4-((*E*)-pent-1-en-1-yl)phenyl)((2*R*,3*S*)-3-(((*tert*-butyldiphenylsilyl) oxy)methyl)-3-(3-methylbut-2-en-1-yl)oxiran-2-yl)methanol (34):

The reaction was carried out in moisture-free glassware under argon atmosphere. To a solution of iodobenzene **32** (1.73 g, 4.91 mmol, 1.20 eq.) dissolved in anhydrous THF (20 mL), *i*-PrMgCl (2.0 M in THF, 2.46 mL, 4.91 mmol, 1.20 eq.) was added dropwise at −40 °C. After stirring for 45 min, epoxy aldehyde **9** (1.67 g, 4.09 mmol, 1.00 eq.) in anhydrous THF (15 mL) was added. The reaction mixture was stirred for 1 h 30 min at −40 °C and was then diluted with saturated aqueous NH_4Cl (50 mL). The mixture was extracted with ethyl acetate (2 x 150 mL) and the combined organic layers were washed with saturated aqueous NaCl (75 mL), dried over $MgSO_4$, filtered, and concentrated under reduced pressure. The crude product was purified by flash column chromatography (0–15% ethyl acetate in *n*-heptane) to give addition products **33** (1.09 g, 1.72 mmol, 42% over two steps, *E*/*Z* ≈ 90:10) and **34** (1.09 g, 1.71 mmol, 42% over two steps, *E*/*Z* ≈ 95:5) as colorless oils.

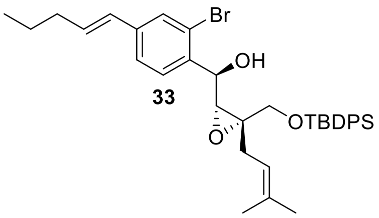

33

For **33**: **^{1}H-NMR** ($CDCl_3$, 400 MHz): δ = 7.75–7.68 (m, 4H, phenyl-*H*), 7.51 (d, 1H, *J* = 1.6 Hz, BrC_q-C*H*), 7.50–7.37 (m, 7H, pentenyl-C_q-CH-C*H* and phenyl-*H*), 7.29 (dd, 1H, *J* = 8.1, 1.6 Hz, pentenyl-C_q-C*H*-CH), 6.36–6.17 (m, 2H, CH_2=CH_2), 5.08–4.99 (m, 2H, Me_2C=C*H* and C*H*-OH), 4.01 (d, 1H, *J* = 11.1 Hz, TBDPSO-CH_2), 3.84 (d, 1H, *J* = 11.1 Hz, TBDPSO-CH_2), 3.13 (d, 1H, *J* = 7.8 Hz, epoxy-C*H*), 2.96 (d, 1H, *J* = 2.5 Hz, O*H*), 2.45 (d, 2H, *J* = 7.2 Hz, Me_2C=CH-CH_2), 2.19 (m, 2H, CH_2-CH=CH), 1.66 (s, 3H, *E*-CH_3), 1.57 (s, 3H, *Z*-CH_3), 1.50 (m, 2H, CH_3-CH_2), 1.12 (s, 9H, ^{t}Bu-CH_3), 0.95 (t, 3H, *J* = 7.3 Hz, CH_3-CH_2); **^{13}C-NMR** ($CDCl_3$, 100 MHz): δ = 139.6 (pentenyl-C_q), 138.2 (BrC_q-C_q), 135.9/135.8 (phenyl-*C*H), 135.5 ($C_q$$Me_2$), 133.0 ($CH_2$-*C*H=CH), 132.7/132.5 (phenyl-C_q), 130.2/130.2 (phenyl-*C*H), 130.1 (BrC_q-*C*H), 128.3 (CH_2-CH=*C*H), 128.1 (pentenyl-C_q-CH-*C*H), 128.1/128.0 (phenyl-*C*H), 125.5 (pentenyl-C_q-*C*H-CH), 123.1 (BrC_q), 117.8 (*C*H=CMe_2), 70.7 (*C*H-OH), 65.4 (TBDPSO-*C*H_2), 64.6 (epoxy-C_q), 63.9 (epoxy-*C*H), 35.2 (*C*H_2-CH=CH), 32.1 (Me_2C=CH-*C*H_2), 27.1 (^{t}Bu-*C*H_3), 25.9 (*E*-*C*H_3), 22.5 (CH_3-*C*H_2), 19.4 (^{t}Bu-C_q), 18.1 (*Z*-*C*H_3), 13.9 (*C*H_3-CH_2); **HRMS (ESI)** m/z calcd. for $C_{36}H_{44}BrO_2Si$: 615.2288 $(M-H_2O+H)^+$; found: 615.2289 $(M-H_2O+H)^+$; **R_f** (*n*-heptane/ethyl acetate 4:1): 0.44; **Specific rotation** $[\alpha]_D^{21.1}$ = +22.9 (c = 0.96; $CHCl_3$).

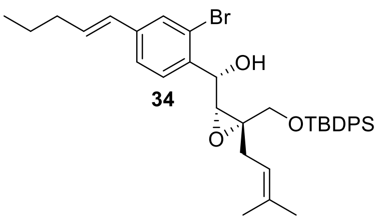

34

For **34**: **^{1}H-NMR** ($CDCl_3$, 400 MHz): δ = 7.72–7.61 (m, 4H, phenyl-*H*), 7.48 (d, 1H, *J* = 1.6 Hz, BrC_q-C*H*), 7.47–7.35 (m, 7H, pentenyl-C_q-CH-C*H* and phenyl-*H*), 7.28 (dd, 1H, *J* = 8.1, 1.6 Hz, pentenyl-C_q-C*H*-CH), 6.35–6.18 (m, 2H, CH_2=CH_2), 5.02 (m, 1H, Me_2C=C*H*), 4.83 (dd, 1H, *J* = 6.2, 3.7 Hz, C*H*-OH), 3.90 (d, 1H, *J* = 11.4 Hz, TBDPSO-CH_2), 3.80 (d, 1H, *J* = 11.5 Hz, TBDPSO-CH_2), 3.11 (d, 1H, *J* = 6.1 Hz, epoxy-C*H*), 2.72 (dd, 1H, *J* = 14.9, 7.9 Hz, Me_2C=CH-CH_2), 2.55 (m, 1H, O*H*), 2.28 (dd, 1H, *J* = 14.7, 6.9 Hz, Me_2C=CH-CH_2), 2.19 (m, 2H, CH_2-CH=CH), 1.67 (s, 3H, *E*-CH_3), 1.58 (s, 3H, *Z*-CH_3), 1.49 (m, 2H, CH_3-CH_2), 1.07 (s, 9H, ^{t}Bu-CH_3), 0.95 (t, 3H, *J* = 7.4 Hz, CH_3-CH_2); **^{13}C-NMR** ($CDCl_3$, 100 MHz): δ = 139.7 (pentenyl-C_q), 138.5 (BrC_q-C_q), 136.0/135.8 (phenyl-*C*H), 135.4 ($C_q$$Me_2$), 133.4 ($CH_2$-*C*H=CH), 133.2/133.1 (phenyl-C_q), 130.1 (BrC_q-*C*H), 130.0/129.9 (phenyl-*C*H), 128.2 (CH_2-CH=*C*H), 128.1 (pentenyl-C_q-CH-*C*H), 127.9/127.8 (phenyl-*C*H), 125.4 (pentenyl-C_q-*C*H-CH), 122.4 (BrC_q), 117.9 (*C*H=CMe_2), 69.4 (*C*H-OH), 65.5 (epoxy-C_q), 64.7 (epoxy-*C*H), 64.3 (TBDPSO-*C*H_2), 35.2 (*C*H_2-CH=CH), 31.4 (Me_2C=CH-*C*H_2), 27.0 (^{t}Bu-CH_3), 25.9 (*E*-*C*H_3), 22.5 (CH_3-*C*H_2), 19.4 (^{t}Bu-C_q), 18.1 (*Z*-*C*H_3), 13.8 (*C*H_3-CH_2); **HRMS (ESI)** m/z calcd. for $C_{36}H_{44}BrO_2Si$: 615.2288 $(M-H_2O+H)^+$; found: 615.2298 $(M-H_2O+H)^+$; **R_f** (*n*-heptane/ethyl acetate 4:1): 0.44; **Specific rotation** $[\alpha]_D^{21.1}$ = −6.9 (c = 1.01; $CHCl_3$).

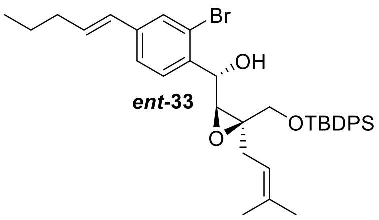

***ent*-33**

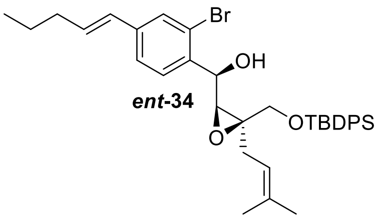

***ent*-34**

***Ent*-33** (0.833 g, 1.31 mmol, 37% over two steps, *E*/*Z* ≈ 90:10) and ***ent*-34** (0.947 g, 1.49 mmol, 43% over two steps, *E*/*Z* ≈ 95:5) were synthesized in analogous manner starting from epoxy aldehyde ***ent*-9** (1.43 g, 3.51 mmol) and iodid **32** (1.79 g, 5.10 mmol). The NMR data are identical to the ones reported for **33** and **34**.

For ***ent*-33**: **HRMS (ESI)** m/z calcd. for $C_{36}H_{44}BrO_2Si$: 615.2288 $(M-H_2O+H)^+$; found: 615.2290 $(M-H_2O+H)^+$; **R_f** (*n*-heptane/ethyl acetate 4:1): 0.44; **Specific rotation** $[\alpha]_D^{21.3}$ = −17.4 (c = 1.44; $CHCl_3$).

For ***ent*-34**: **HRMS (ESI)** m/z calcd. for $C_{36}H_{44}BrO_2Si$: 615.2288 $(M-H_2O+H)^+$; found: 615.2298 $(M-H_2O+H)^+$; **R_f** (*n*-heptane/ethyl acetate 4:1): 0.31; **Specific rotation** $[\alpha]_D^{21.3}$ = +2.8 (c = 1.71; $CHCl_3$).

Assignment of the stereochemistry of compound **33** and **34** was achieved *via* conclusion by analogy (Table S2). Therefore, both retardation factors of **33** and **34** (R^3) and the ^{1}H-NMR-signals of the protons of the oxirane where compared with **12** and **13** (R^2) and the intermediates **SI2** and **SI3** (R^1) of the synthesis of avicennone C.[1] The absolute stereochemistry of

[1] Kleiner, Y.; Hammann, P.; Becker, J.; Bauer, A.; Pöverlein, C.; Schuler, S. M. M. *J. Org. Chem.* **2020**, *85*, 13108–13120.

12, **13**, **SI2** and **SI3** was unambiguously determined by single X-ray crystal diffraction of crystal intermediates in the course of their total synthesis.

***Table S2*. Comparison of retardation factors and ^{1}H-NMR-signals of protons of the epoxide for the assignment of the stereochemistry of 33 and 34.**

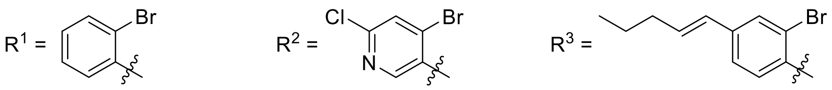

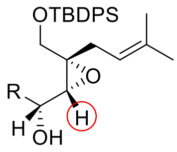

SI2, 12 or **33**

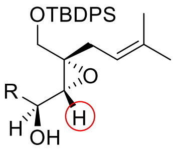

SI3, 13 or **34**

compound	SI2 (R = R^1)	12 (R = R^2)	33 (R = R^3)	SI3 (R = R^1)	13 (R = R^2)	34 (R = R^3)
**NMR-data**[a]	3.07, d, 7.9	3.15, d, 7.8	3.13, d, 7.8	3.09, d, 6.0	3.11, d, 6.1	3.11, d, 6.1
**R*f*** [b]	0.32	0.37	0.44	0.25	0.34	0.31

[a] The NMR-spectra were recorded in $CDCl_3$: δ_H (400 MHz), multiplicity, *J* (epoxy-C*H*) in [Hz].

[b] Eluent for determination of retardation factors (R*f*): *n*-heptane/ethyl acetate 4:1.

(*R*)-(2-Bromo-4-((*E*)-pent-1-en-1-yl)phenyl)((2*R*,3*S*)-3-(((*tert*-butyldiphenylsilyl)oxy)methyl)-3-(3-methylbut-2-en-1-yl)oxiran-2-yl)methyl acetate (SI4):

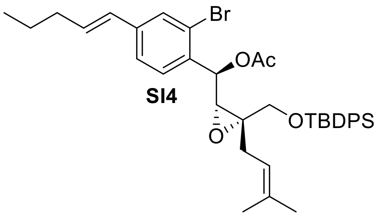

To a solution of alcohol **33** (1.07 g, 1.68 mmol, 1.00 eq.) in anhydrous DCM (20 mL) anhydrous pyridine (0.407 mL, 5,04 mmol, 3.00 eq.), Ac_2O (0.477 mL, 5.04 mmol, 3.00 eq.), and DMAP (0.021 g, 0.17 mmol, 0.10 eq.) were added. The reaction mixture was stirred for 2 h until the TLC showed complete conversion. Toluene (40 mL) was added and the reaction mixture was concentrated *in vacuo*. The crude product was purified by flash column chromatography (0–10% ethyl acetate in *n*-heptane) to give **SI4** (1.10 g, 1.63 mmol, 97%, *E*/*Z* > 90:10) as a colorless oil.

^{1}H-NMR ($CDCl_3$, 400 MHz): δ = 7.73–7.63 (m, 4H, phenyl-*H*), 7.48 (d, 1H, *J* = 1.6 Hz, BrC$_q$-CH), 7.46–7.29 (m, 7H, pentenyl-C$_q$-CH-C*H* and phenyl-*H*), 7.24 (dd, 1H, *J* = 8.1, 1.6 Hz, pentenyl-C$_q$-C*H*-CH), 6.33–6.16 (m, 2H, C*H*$_2$=C*H*$_2$), 5.92 (d, 1H, *J* = 7.6 Hz, C*H*-OAc), 5.07 (m, 1H, Me$_2$C=C*H*), 4.00 (d, 1H, *J* = 11.2 Hz, TBDPSO-C*H*$_2$), 3.86 (d, 1H, *J* = 11.2 Hz, TBDPSO-C*H*$_2$), 3.19 (d, 1H, *J* = 7.6 Hz, epoxy-C*H*), 2.71 (dd, 1H, *J* = 15.0, 7.7 Hz, Me$_2$C=CH-C*H*$_2$), 2.36 (dd, 1H, *J* = 15.0, 6.8 Hz, Me$_2$C=CH-C*H*$_2$), 2.18 (m, 2H, C*H*$_2$-CH=CH), 1.97 (s, 3H, CO-C*H*$_3$), 1.69 (s, 3H, *E*-C*H*$_3$), 1.61 (s, 3H, *Z*-C*H*$_3$), 1.49 (m, 2H, CH$_3$-C*H*$_2$), 1.07 (s, 9H, ^{t}Bu-C*H*$_3$), 0.95 (t, 3H, *J* = 7.4 Hz, C*H*$_3$-CH$_2$); **^{13}C-NMR** ($CDCl_3$, 100 MHz): δ = 169.1 (C=O), 140.1 (pentenyl-*C*$_q$), 135.9/135.8 (phenyl-*C*H), 135.3 (BrC$_q$-*C*$_q$ or *C*$_q$Me$_2$), 135.2 (BrC$_q$-*C*$_q$ or *C*$_q$Me$_2$), 133.4 (CH$_2$-*C*H=CH), 133.4/133.2 (phenyl-*C*$_q$), 130.4 (BrC$_q$-*C*H), 129.8 (phenyl-*C*H), 128.8 (pentenyl-C$_q$-CH-*C*H), 128.2 (CH$_2$-CH=*C*H), 127.9/127.8 (phenyl-*C*H), 125.3 (pentenyl-C$_q$-*C*H-CH), 123.8 (Br*C*$_q$), 118.1 (*C*H=CMe$_2$), 71.2 (*C*H-OAc), 65.3 (epoxy-*C*$_q$), 64.1 (TBDPSO-*C*H$_2$), 62.4 (epoxy-*C*H), 35.2 (*C*H$_2$-CH=CH), 31.3 (Me$_2$C=CH-*C*H$_2$), 27.0 (^{t}Bu-*C*H$_3$), 25.9 (*E*-*C*H$_3$), 22.5 (CH$_3$-*C*H$_2$), 20.9 (CO-*C*H$_3$), 19.4 (^{t}Bu-*C*$_q$), 18.2 (*Z*-*C*H$_3$), 13.8 (*C*H$_3$-CH$_2$); **HRMS (ESI)** m/z calcd. for $C_{38}H_{48}BrO_4Si$: 675.2500 $(M+H)^+$; found: 675.2515 $(M+H)^+$; **R$_f$** (*n*-heptane/ethyl acetate 4:1): 0.56; **Specific rotation** $[\alpha]_D^{21.2}$ = +9.4 (c = 1.18; $CHCl_3$).

(*S*)-(2-Bromo-4-((*E*)-pent-1-en-1-yl)phenyl)((2*R*,3*S*)-3-(((*tert*-butyldiphenylsilyl)oxy)methyl)-3-(3-methylbut-2-en-1-yl)oxiran-2-yl)methyl acetate (SI5):

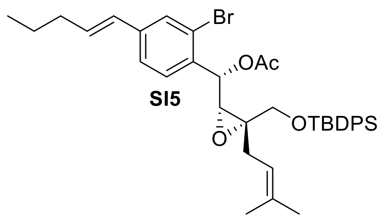

SI5

SI5 (1.12 g, 1.65 mmol, 99%, *E*/*Z* ≈ 93:7) was synthesized in analogous manner starting from **34** (1.06 g, 1.67 mmol) and was obtained as colorless oil.

^{1}H-NMR ($CDCl_3$, 400 MHz): δ = 7.68–7.57 (m, 4H, phenyl-*H*), 7.45 (d, 1H, *J* = 1.6 Hz, BrC_q-CH), 7.44–7.32 (m, 6H, phenyl-*H*), 7.26 (d, 1H, *J* = 8.1 Hz, pentenyl-C_q-CH-C*H*), 7.21 (dd, 1H, *J* = 8.1, 1.6 Hz, pentenyl-C_q-C*H*-CH), 6.31–6.16 (m, 2H, CH_2=CH_2), 5.83 (d, 1H, *J* = 8.1 Hz, C*H*-OAc), 4.93 (m, 1H, Me_2C=C*H*), 3.80 (d, 1H, *J* = 11.5 Hz, TBDPSO-CH_2), 3.76 (d, 1H, *J* = 11.5 Hz, TBDPSO-CH_2), 3.34 (d, 1H, *J* = 8.1 Hz, epoxy-C*H*), 2.69 (dd, 1H, *J* = 15.0, 7.9 Hz, Me_2C=CH-CH_2), 2.26 (dd, 1H, *J* = 14.9, 6.7 Hz, Me_2C=CH-CH_2), 2.18 (m, 2H, CH_2-CH=CH), 2.02 (s, 3H, CO-CH_3), 1.64 (s, 3H, *E*-CH_3), 1.54 (s, 3H, *Z*-CH_3), 1.49 (m, 2H, CH_3-CH_2), 1.04 (s, 9H, ^{t}Bu-CH_3), 0.94 (t, 3H, *J* = 7.4 Hz, CH_3-CH_2); **^{13}C-NMR** ($CDCl_3$, 100 MHz): δ = 169.5 (C=O), 140.2 (pentenyl-C_q), 135.9/135.8 (phenyl-*C*H), 135.4 ($C_q$$Me_2$), 134.9 (Br$C_q$-$C_q$), 133.7 ($CH_2$-*C*H=CH), 133.5/133.1 (phenyl-C_q), 130.6 (BrC_q-*C*H), 129.9/129.8 (phenyl-*C*H), 129.1 (pentenyl-C_q-CH-*C*H), 128.1 (CH_2-CH=*C*H), 127.8/127.8 (phenyl-*C*H), 125.2 (pentenyl-C_q-*C*H-CH), 123.2 (BrC_q), 117.9 (*C*H=CMe_2), 73.5 (*C*H-OAc), 65.0 (TBDPSO-*C*H_2), 64.7 (epoxy-C_q), 62.4 (epoxy-*C*H), 35.2 (*C*H_2-CH=CH), 30.9 (Me_2C=CH-*C*H_2), 27.0 (^{t}Bu-*C*H_3), 25.9 (*E*-*C*H_3), 22.5 (CH_3-*C*H_2), 21.1 (CO-*C*H_3), 19.4 (^{t}Bu-C_q), 18.1 (*Z*-*C*H_3), 13.8 (*C*H_3-CH_2); **HRMS (ESI)** m/z calcd. for $C_{38}H_{48}BrO_4Si$: 675.2500 $(M+H)^+$; found: 675.2515 $(M+H)^+$; **R_f** (*n*-heptane/ethyl acetate 4:1): 0.42; **Specific rotation** $[\alpha]_D^{21.2}$ = +1.9 (c = 1.05; $CHCl_3$).

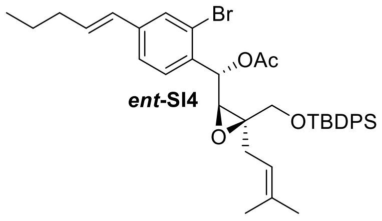

***ent*-SI4**

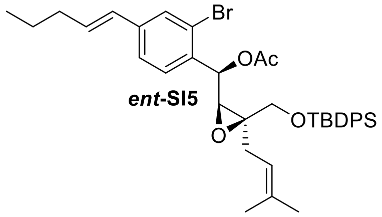

***ent*-SI5**

***Ent*-SI4** (0.864 g, 1.28 mmol, 97%, *E*/*Z* ≈ 95:5) and ***ent*-SI5** (0.918 g, 1.36 mmol, 91%, *E*/*Z* ≈ 95:5) were synthesized in analogous manner starting from ***ent*-33** (0.832 g, 1.31 mmol, 1.00 eq.) and ***ent*-34** (0.942 g, 1.49 mmol). The NMR data are identical to the ones reported for **SI4** and **SI5**.

For ***ent*-SI4**: **HRMS (ESI)** m/z calcd. for $C_{38}H_{48}BrO_4Si$: 675.2500 $(M+H)^+$; found: 675.2522 $(M+H)^+$; **R_f** (*n*-heptane/ethyl acetate 4:1): 0.56; **Specific rotation** $[\alpha]_D^{21.2}$ = −5.5 (c = 1.26; $CHCl_3$).

For ***ent*-SI5**: **HRMS (ESI)** m/z calcd. for $C_{38}H_{48}BrO_4Si$: 675.2500 $(M+H)^+$; found: 675.2518 $(M+H)^+$; **R_f** (*n*-heptane/ethyl acetate 4:1): 0.42; **Specific rotation** $[\alpha]_D^{21.2}$ = −0.76 (c = 1.32; $CHCl_3$).

((2*S*,3*R*)-3-((*R*)-(2-Bromo-4-((*E*)-pent-1-en-1-yl)phenyl)(hydroxy)methyl)-2-(3-methylbut-2-en-1-yl)oxiran-2-yl)methyl acetate (SI6):

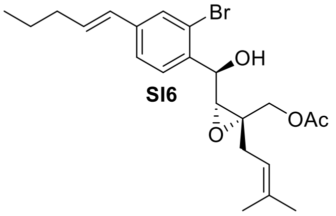

SI6

In a 50 mL falcon tube, glacial acetic acid (0.145 mL, 2.53 mmol, 1.60 eq.) followed by TBAF (1 M in THF, 2.22 mL, 2.22 mmol, 1.40 eq.) were added to a solution of TBDPS-protected alcohol **SI4** (1.07 g, 1.58 mmol, 1.00 eq.) in THF (21 mL). After 2 h, a second portion of glacial acetic acid (0.40 eq.) and TBAF (0.20 eq.) and, after further 2 h, a third portion of glacial acetic acid (0.40 eq.) and TBAF (0.20 eq.) were added to achieve complete conversion, which was monitored by TLC. The reaction mixture was loaded on silica and was then prepurified by flash column chromatography (0–20% ethyl acetate in *n*-heptane) to give an inseparable mixture of the two regioisomers with acetate on the primary or secondary hydroxy group, respectively.

To complete the acetate migration, the product mixture was dissolved in anhydrous DCM (15 mL) and DBU (0.199 mL, 1.33 mmol, 0.84 eq.) was added. After stirring at room temperature for 1 h 30 min, further DBU (0.28 eq.) was added. 2 h

later, the LC-MS indicated an acyl migration to the primary hydroxy group of > 90%.[2] Saturated aqueous NH_4Cl (30 mL) was added. The mixture was extracted twice with ethyl acetate (150 mL and 50 mL) and the combined organic layers were washed with saturated aqueous NaCl (50 mL), dried over $MgSO_4$, filtered, and concentrated under reduced pressure to yield **SI6** (0.601 g, 1.37 mmol, 87% over two steps, *E*/*Z* ≈ 93:7) as colorless oil.

^{1}H-NMR ($CDCl_3$, 400 MHz): δ = 7.53 (d, 1H, *J* = 1.7 Hz, BrC$_q$-C*H*), 7.50 (d, 1H, *J* = 8.1 Hz, pentenyl-C$_q$-CH-C*H*), 7.32 (dd, 1H, *J* = 8.1, 1.7 Hz, pentenyl-C$_q$-C*H*-CH), 6.36–6.18 (m, 2H, C*H*$_2$=C*H*$_2$), 5.17 (dd, 1H, *J* = 7.4, 1.7 Hz, C*H*-OH), 5.09 (m, 1H, Me$_2$C=C*H*), 4.39 (d, 1H, *J* = 12.0 Hz, C*H*$_2$-OAc), 4.34 (d, 1H, *J* = 12.0 Hz, C*H*$_2$-OAc), 3.19 (d, 1H, *J* = 7.4 Hz, epoxy-C*H*), 3.10 (d, 1H, *J* = 2.6 Hz, O*H*), 2.36 (m, 2H, Me$_2$C=CH-C*H*$_2$), 2.19 (m, 2H, C*H*$_2$-CH=CH), 2.12 (s, 3H, CO-C*H*$_3$), 1.70 (s, 3H, *E*-C*H*$_3$), 1.60 (s, 3H, *Z*-C*H*$_3$), 1.49 (m, 2H, CH$_3$-C*H*$_2$), 0.95 (t, 3H, *J* = 7.4 Hz, C*H*$_3$-CH$_2$); **^{13}C-NMR** ($CDCl_3$, 100 MHz): δ = 171.4 (C=O), 139.8 (pentenyl-*C*$_q$), 137.9 (BrC$_q$-*C*$_q$), 136.0 (*C*$_q$Me$_2$), 133.1 (CH$_2$-*C*H=CH), 130.2 (BrC$_q$-*C*H), 128.3 (pentenyl-C$_q$-CH-*C*H), 128.2 (CH$_2$-CH=*C*H), 125.5 (pentenyl-C$_q$-*C*H-CH), 123.0 (Br*C*$_q$), 117.3 (*C*H=CMe$_2$), 69.7 (*C*H-OH), 65.1 (epoxy-*C*H), 63.6 (*C*H$_2$-OAc), 62.0 (epoxy-*C*$_q$), 35.2 (*C*H$_2$-CH=CH), 32.2 (Me$_2$C=CH-*C*H$_2$), 26.0 (*E*-*C*H$_3$), 22.5 (CH$_3$-*C*H$_2$), 21.0 (CO-*C*H$_3$), 18.1 (*Z*-*C*H$_3$), 13.8 (*C*H$_3$-CH$_2$); **HRMS (ESI)** m/z calcd. for $C_{22}H_{29}BrO_4Na$: 459.1141 $(M+Na)^+$; found: 459.1157 $(M+Na)^+$; **R$_f$** (*n*-heptane/ethyl acetate 2:1): 0.44; **Specific rotation** $[\alpha]_D^{21.9}$ = −10.9 (c = 1.01; $CHCl_3$).

((2*S*,3*R*)-3-((*S*)-(2-Bromo-4-((*E*)-pent-1-en-1-yl)phenyl)(hydroxy)methyl)-2-(3-methylbut-2-en-1-yl)oxiran-2-yl)methyl acetate (SI7):

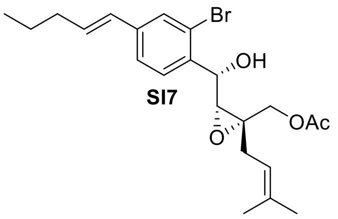

SI7

SI7 was synthesized in analogous manner starting from **SI5** (1.09 g, 1.61 mmol). Purification by flash column chromatography (0–30% ethyl acetate in *n*-heptane) after acetate migration yielded **SI7**[2] (0.561 g, 1.28 mmol, 79% over two steps, *E*/*Z* ≈ 92:8) as colorless oil.

^{1}H-NMR ($CDCl_3$, 400 MHz): δ = 7.53 (d, 1H, *J* = 1.5 Hz, BrC$_q$-*C*H), 7.49 (d, 1H, *J* = 8.1 Hz, pentenyl-C$_q$-CH-C*H*), 7.32 (dd, 1H, *J* = 8.1, 1.5 Hz, pentenyl-C$_q$-C*H*-CH), 6.37–6.20 (m, 2H, C*H*$_2$=C*H*$_2$), 5.04 (m, 1H, Me$_2$C=C*H*), 5.00 (dd, 1H, *J* = 6.5 Hz, C*H*-OH), 4.33 (d, 1H, *J* = 12.1 Hz, C*H*$_2$-OAc), 4.25 (d, 1H, *J* = 12.1 Hz, C*H*$_2$-OAc), 3.18 (d, 1H, *J* = 6.6, 3.2 Hz epoxy-C*H*), 2.69 (d, 1H, *J* = 3.4 Hz, *J* = O*H*), 2.55 (dd, 1H, *J* = 14.9, 7.9 Hz, Me$_2$C=CH-C*H*$_2$), 2.26–2.15 (m, 3H, Me$_2$C=CH-C*H*$_2$ and C*H*$_2$-CH=CH), 2.05 (s, 3H, CO-C*H*$_3$), 1.69 (s, 3H, *E*-C*H*$_3$), 1.57 (s, 3H, *Z*-C*H*$_3$), 1.50 (m, 2H, CH$_3$-C*H*$_2$), 0.95 (t, 3H, *J* = 7.4 Hz, C*H*$_3$-CH$_2$); **^{13}C-NMR** ($CDCl_3$, 100 MHz): δ = 170.7 (C=O), 139.9 (pentenyl-*C*$_q$), 138.1 (BrC$_q$-*C*$_q$), 136.2 (*C*$_q$Me$_2$), 133.4 (CH$_2$-*C*H=CH), 130.2 (BrC$_q$-*C*H), 128.2 (pentenyl-C$_q$-CH-*C*H), 128.1 (CH$_2$-CH=*C*H), 125.5 (pentenyl-C$_q$-*C*H-CH), 122.5 (Br*C*$_q$), 117.0 (*C*H=CMe$_2$), 69.8 (*C*H-OH), 65.1 (epoxy-*C*H), 64.3 (*C*H$_2$-OAc), 63.1 (epoxy-*C*$_q$), 35.2 (*C*H$_2$-CH=CH), 32.0 (Me$_2$C=CH-*C*H$_2$), 25.9 (*E*-*C*H$_3$), 22.5 (CH$_3$-*C*H$_2$), 20.9 (CO-*C*H$_3$), 18.0 (*Z*-*C*H$_3$), 13.8 (*C*H$_3$-CH$_2$); **HRMS (ESI)** m/z calcd. for $C_{22}H_{30}BrO_4$: 437.1322 $(M+H)^+$; found: 437.1335 $(M+H)^+$; **R$_f$** (*n*-heptane/ethyl acetate 2:1): 0.41; **Specific rotation** $[\alpha]_D^{21.9}$ = −4.2 (c = 0.94; $CHCl_3$).

[2] A stagnation of the acyl migration was observed after stirring overnight and the reaction was therefore stopped. The two regioisomers could not be separated by chromatography. The minor isomer was finally removed on a later stage after TIPS-protection and saponification by flash chromatography.

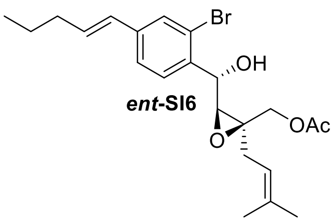

ent-**SI6**

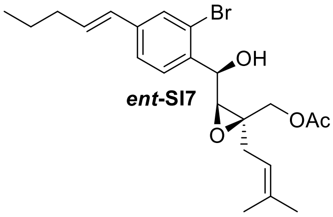

ent-**SI7**

***Ent*-SI6**[2] (0.476 g, 1.09 mmol, 87% over two steps, *E*/*Z* ≈ 93:7) and ***ent*-SI7**[2] (0.508 g, 1.16 mmol, 86% over two steps, *E*/*Z* ≈ 95:5) were synthesized in analogous manner starting from ***ent*-SI4** (0.844 g, 1.25 mmol) and ***ent*-SI5** (0.918 g, 1.36 mmol) and were obtained as colorless oils. The NMR data are identical to the ones reported for **SI6** and **SI7**.

For ***ent*-SI6**: **HRMS (ESI)** m/z calcd. for $C_{22}H_{29}BrO_4Na$: 459.1141 $(M+Na)^+$; found: 459.1140 $(M+Na)^+$; **R$_f$** (*n*-heptane/ethyl acetate 2:1): 0.44; **Specific rotation** $[\alpha]_D^{21.2}$ = +14.2 (c = 1.34; $CHCl_3$).

For ***ent*-SI7**: **HRMS (ESI)** m/z calcd. for $C_{22}H_{29}BrO_4Na$: 459.1141 $(M+Na)^+$; found: 459.1148 $(M+Na)^+$; **R$_f$** (*n*-heptane/ethyl acetate 2:1): 0.41; **Specific rotation** $[\alpha]_D^{23.3}$ = +0.8 (c = 1.25; $CHCl_3$).

((2*S*,3*R*)-3-((*R*)-(2-Bromo-4-((*E*)-pent-1-en-1-yl)phenyl)((triisopropylsilyl)oxy)methyl)-2-(3-methylbut-2-en-1-yl)oxiran-2-yl)methanol (SI8):

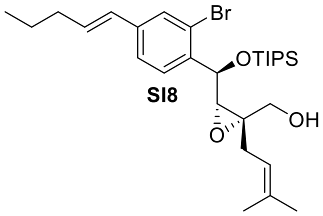

SI8

2,6-Lutidine (0.920 mL, 7.90 mmol, 4.00 eq.) and then TIPSOTf (1.06 mL, 3.95 mmol, 3.00 eq.) were added to a solution of alcohol **SI6** (0.576 g, 1.32 mmol, 1.00 eq.) in anhydrous DCM (20 mL) at 0 °C. The reaction mixture was stirred at room temperature for 2 h 30 min. Because the TLC showed remaining starting material, 2,6-lutidine (3.00 eq.) and TIPSOTf (1.50 eq.) were added. After stirring for further 1h 30 min at room temperature, the TLC showed complete conversion. Saturated aqueous NH_4Cl (30 mL) was added. The mixture was extracted twice with ethyl acetate (150 mL and 50 mL) and the combined organic layers were washed with saturated aqueous NaCl (50 mL), dried over $MgSO_4$, filtered, and concentrated under reduced pressure. The TIPS and acetate protected crude product was purified twice by flash column chromatography (0–10% and 0–3% ethyl acetate in *n*-heptane).

Prepurified crude product (0.540 g, 0.91 mmol, 1.00 eq.) was dissolved in THF/H_2O (5:1, 20 mL/4 mL) and LiOH · H_2O (0.076 g, 1.82 mmol, 2.00 eq.) was added. After stirring at room temperature overnight, a second portion of LiOH · H_2O (1.11 eq.) was added to complete conversion. The reaction mixture was allowed to stir for further 1 h 30 min at room temperature until TLC showed complete conversion. Saturated aqueous $NaHCO_3$ (30 mL) was added. The mixture was extracted twice with ethyl acetate (100 mL and 50 mL) and the combined organic layers were washed with saturated aqueous NaCl (50 mL), dried over $MgSO_4$, filtered, and concentrated under reduced pressure. The crude product was purified by flash column chromatography (0–10% ethyl acetate in *n*-heptane to give **SI8** (0.335 g, 0.611 mmol, 46% over two steps, *E*/*Z* ≈ 95:5) as a colorless oil.

^{1}H-NMR ($CDCl_3$, 400 MHz): δ = 7.52 (d, 1H, *J* = 8.1 Hz, pentenyl-C_q-CH-C*H*), 7.48 (d, 1H, *J* = 1.6 Hz, BrC_q-C*H*), 7.32 (dd, 1H, *J* = 8.1, 1.6 Hz, pentenyl-C_q-C*H*-CH), 6.35–6.18 (m, 2H, C*H*$_2$=C*H*$_2$), 5.32 (d, 1H, *J* = 7.0 Hz, C*H*-OTIPS), 5.08 (m, 1H, Me_2C=C*H*), 4.01 (dd, 1H, *J* = 11.9, 5.1 Hz, C*H*$_2$-OH), 3.92 (dd, 1H, *J* = 11.9, 8.1 Hz, C*H*$_2$-OH), 3.07 (d, 1H, *J* = 7.0 Hz, epoxy-C*H*), 2.65 (dd, 1H, *J* = 14.6, 8.3 Hz, Me_2C=CH-C*H*$_2$), 2.19 (m, 2H, C*H*$_2$-CH=CH), 2.07 (dd, 1H, *J* = 14.6, 7.0 Hz, Me_2C=CH-C*H*$_2$), 1.82 (dd, 1H, *J* = 8.1, 5.2 Hz, O*H*), 1.68 (s, 3H, *E*-C*H*$_3$), 1.61 (s, 3H, *Z*-C*H*$_3$), 1.49 (m, 2H, CH_3-C*H*$_2$), 1.12–0.91 (m, 24H, TIPS-*H* and C*H*$_3$-CH_2); **^{13}C-NMR** ($CDCl_3$, 100 MHz): δ = 139.9 (BrC_q-*C*$_q$), 139.5 (pentenyl-*C*$_q$), 135.6 (*C*$_q$$Me_2$), 132.8 ($CH_2$-*C*H=CH), 129.9 (Br$C_q$-*C*H), 128.8 (pentenyl-$C_q$-CH-*C*H), 128.4 ($CH_2$-CH=*C*H), 125.5 (pentenyl-C_q-*C*H-CH), 122.4 (Br*C*$_q$), 118.1 (*C*H=CMe_2), 69.7 (*C*H-OTIPS), 67.5 (epoxy-*C*H), 65.5 (epoxy-*C*$_q$), 62.4 (*C*H_2-OH), 35.2 (*C*H_2-CH=CH), 32.4 (Me_2C=CH-*C*H_2), 25.9 (*E*-*C*H_3), 22.6 (CH_3-*C*H_2), 18.0 (*Z*-*C*H_3), 18.0/17.9 (CH(*C*H_3)$_2$), 13.9 (*C*H_3-CH_2), 12.3 (*C*H(CH_3)$_2$); **HRMS (ESI)** m/z calcd. for $C_{29}H_{46}BrO_2Si$: 533.2445 $(M+H)^+$; found: 533.2456 $(M+H)^+$; **R$_f$** (*n*-heptane/ethyl acetate 4:1): 0.41; **Specific rotation** $[\alpha]_D^{22.6}$ = −32.0 (c = 1.37; $CHCl_3$).

((2*S*,3*R*)-3-((*S*)-(2-Bromo-4-((*E*)-pent-1-en-1-yl)phenyl)((triisopropylsilyl)oxy)methyl)-2-(3-methylbut-2-en-1-yl)oxiran-2-yl)methanol (SI9):

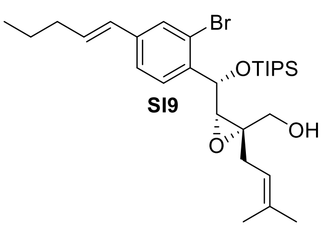

SI9 (0.407 g, 0.738 mmol, 63% over two steps, *E*/*Z* ≈ 94:6) was synthesized in analogous manner starting from **SI7** (1.06 g, 1.67 mmol) and was obtained as colorless oil.

^{1}H-NMR ($CDCl_3$, 400 MHz): δ = 7.51–7.47 (m, 2H, pentenyl-C_q-CH-C*H* and BrC_q-C*H*), 7.32 (dd, 1H, *J* = 8.1, 1.6 Hz, pentenyl-C_q-C*H*-CH), 6.36–6.20 (m, 2H, C*H*$_2$=C*H*$_2$), 5.12 (d, 1H, *J* = 7.8 Hz, C*H*-OTIPS), 4.95 (m, 1H, Me_2C=C*H*), 3.79 (dd, 1H, *J* = 12.1, 6.5 Hz, C*H*$_2$-OH), 3.66 (dd, 1H, *J* = 12.1, 6.5 Hz, C*H*$_2$-OH), 3.24 (d, 1H, *J* = 7.7 Hz, epoxy-C*H*), 2.45 (dd, 1H, *J* = 14.8, 7.7 Hz, Me_2C=CH-C*H*$_2$), 2.24–2.12 (m, 3H, C*H*$_2$-CH=CH and Me_2C=CH-C*H*$_2$), 1.69–1.60 (m, 1H, O*H*), 1.62 (s, 3H, *E*-C*H*$_3$), 1.55–1.44 (m, 2H, CH_3-C*H*$_2$), 1.51 (s, 3H, *Z*-C*H*$_3$), 1.17–0.92 (m, 24H, TIPS-*H* and C*H*$_3$-CH_2); **^{13}C-NMR** ($CDCl_3$, 100 MHz): δ = 139.6 (pentenyl-*C*$_q$), 139.3 (BrC_q-*C*$_q$), 135.4 (*C*$_q$Me$_2$), 133.2 (CH_2-*C*H=CH), 130.1 (BrC_q-*C*H), 129.6 (pentenyl-C_q-CH-C*H*), 128.2 (CH_2-CH=*C*H), 125.5 (pentenyl-C_q-C*H*-CH), 121.6 (Br*C*$_q$), 117.9 (*C*H=CMe$_2$), 72.3 (*C*H-OTIPS), 68.0 (epoxy-*C*H), 64.8 (epoxy-*C*$_q$), 63.3 (*C*H$_2$-OH), 35.2 (*C*H$_2$-CH=CH), 32.0 (Me_2C=CH-*C*H$_2$), 25.9 (*E*-*C*H$_3$), 22.5 (CH_3-*C*H$_2$), 18.0 (*Z*-*C*H$_3$), 18.0/17.9 (CH(*C*H$_3$)$_2$), 13.9 (*C*H$_3$-CH_2), 12.4 (*C*H(CH$_3$)$_2$); **HRMS (ESI)** m/z calcd. for $C_{29}H_{46}BrO_2Si$: 533.2445 $(M+H)^+$; found: 533.2453 $(M+H)^+$; **R$_f$** (*n*-heptane/ethyl acetate 4:1): 0.48; **Specific rotation** $[\alpha]_D^{22.6}$ = +11.0 (c = 1.18; $CHCl_3$).

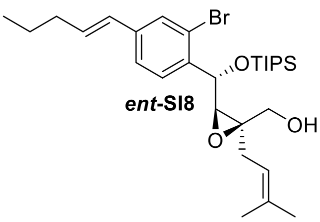

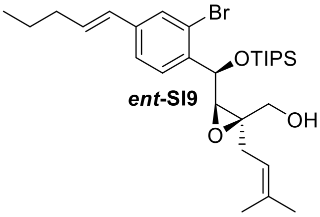

***Ent*-SI8** (0.267 g, 0.488 mmol, 56% over two steps, *E*/*Z* ≈ 95:5) and ***ent*-SI9** (0.337 g, 0.611 mmol, 56% over two steps, *E*/*Z* ≈ 95:5) were synthesized in analogous manner starting from ***ent*-SI6** (0.476 g, 1.09 mmol) and ***ent*-SI7** (0.480 g, 1.10 mmol). The NMR data are identical to the ones reported for **SI8** and **SI9**.

For ***ent*-SI8**: **HRMS (ESI)** m/z calcd. for $C_{29}H_{46}BrO_2Si$: 533.2445 $(M+H)^+$; found: 533.2460 $(M+H)^+$; **R$_f$** (*n*-heptane/ethyl acetate 4:1): 0.41; **Specific rotation** $[\alpha]_D^{21.4}$ = +33.0 (c = 1.54; $CHCl_3$).

For ***ent*-SI9**: **HRMS (ESI)** m/z calcd. for $C_{29}H_{46}BrO_2Si$: 533.2445 $(M+H)^+$; found: 533.2462 $(M+H)^+$; **R$_f$** (*n*-heptane/ethyl acetate 4:1): 0.48; **Specific rotation** $[\alpha]_D^{21.4}$ = −9.7 (c = 1.54; $CHCl_3$).

(2*S*,3*R*)-3-((*R*)-(2-Bromo-4-((*E*)-pent-1-en-1-yl)phenyl)((triisopropylsilyl)oxy)methyl)-2-(3-methylbut-2-en-1-yl)oxirane-2-carbonitrile (SI10):

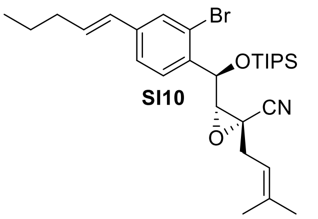

Alcohol **SI8** (0.335 g, 0.611 mmol, 1.00 eq.) was dissolved in MeCN/H_2O 9:1 (36 mL/4.0 mL) and NH_4OAc (0.187 g, 2.43 mmol, 4.00 eq.), BIAB (0.587 g, 1.82 mmol, 3.00 eq.), and TEMPO (0.019 g, 0.012 mmol, 0.20 eq.) were added. After stirring overnight at room temperature, a second portion of NH_4OAc (2.00 eq.), BIAB (1.50 eq.), and TEMPO (0.10 eq.) was added. The reaction solution was stirred further 2 h at room temperature, until the TLC and the LC-MS showed a complete conversion to the nitrile (n.b. the starting material is first converted to the intermediate aldehyde and then converted to the nitrile in a two-step fashion). Saturated aqueous Na_2SO_3 (30 mL) was added. The mixture was extracted twice with ethyl acetate (150 mL and 70 mL) and the combined organic layers were washed with saturated aqueous NaCl (50 mL), dried over $MgSO_4$, filtered, and concentrated under reduced pressure. The crude product was purified *via* flash column chromatography (0–3% ethyl acetate in *n*-heptane) to give nitrile **SI10** (0.158 g, 0.289 mmol, 48%, *E*/*Z* ≈ 92:8)[3] as a colorless oil.

[3] The impurity was estimated to approx. 7% and could not be fully separated from the inseparable *E*/*Z*-mixture at this stage.

^{1}H-NMR ($CDCl_3$, 400 MHz): δ = 7.63 (d, 1H, *J* = 8.1 Hz, pentenyl-C_q-CH-C*H*), 7.51 (d, 1H, *J* = 1.6 Hz, BrC_q-*C*H), 7.34 (dd, 1H, *J* = 8.1, 1.6 Hz, pentenyl-C_q-C*H*-CH), 6.37–6.21 (m, 2H, CH_2=CH_2), 5.46 (d, 1H, *J* = 5.4 Hz, C*H*-OTIPS), 5.07 (m, 1H, Me_2C=C*H*), 3.16 (d, 1H, *J* = 5.4 Hz, epoxy-C*H*), 2.57 (dd, 1H, *J* = 14.9, 7.8 Hz, Me_2C=CH-CH_2), 2.40 (dd, 1H, *J* = 14.6, 7.0 Hz, Me_2C=CH-CH_2), 2.24–2.15 (m, 2H, CH_2-CH=CH), 1.70 (s, 3H, *E*-CH_3), 1.58 (s, 3H, *Z*-CH_3), 1.50 (m, 2H, CH_3-CH_2), 1.20–0.99 (m, 21H, TIPS-*H*), 0.96 (t, 3H, *J* = 7.4, CH_3-CH_2); **^{13}C-NMR** ($CDCl_3$, 100 MHz): δ = 139.9 (pentenyl-C_q), 138.6 ($C_q$$Me_2$), 138.2 (Br$C_q$-$C_q$), 133.2 ($CH_2$-*C*H=CH), 130.0 (Br$C_q$-*C*H), 128.9 (pentenyl-$C_q$-CH-C*H*), 128.3 ($CH_2$-CH=*C*H), 125.5 (pentenyl-C_q-C*H*-CH), 122.3 (BrC_q), 117.4 (*C*N), 114.8 (*C*H=CMe$_2$), 70.1 (*C*H-OTIPS), 65.5 (epoxy-*C*H), 53.0 (epoxy-C_q), 35.2 (*C*H_2-CH=CH), 33.2 (Me_2C=CH-*C*H_2), 25.9 (*E*-*C*H_3), 22.5 (CH_3-*C*H_2), 18.1 (*Z*-*C*H_3), 18.0/17.9 (CH(CH_3)$_2$), 13.9 (*C*H_3-CH_2), 12.3 (*C*H(CH_3)$_2$); **HRMS (ESI)** m/z calcd. for $C_{29}H_{45}BrNO_2Si$: 546.2397 $(M+H)^+$; $)^+$; found: no ionisation was observed; **R$_f$** (*n*-heptane/ethyl acetate 20:1): 0.49; **Specific rotation** was not determined due to *E*/*Z*-mixture and significant amount of impurities.[3]

(2*S*,3*R*)-3-((*S*)-(2-Bromo-4-((*E*)-pent-1-en-1-yl)phenyl)((triisopropylsilyl)oxy)methyl)-2-(3-methylbut-2-en-1-yl)oxirane-2-carbonitrile (SI11):

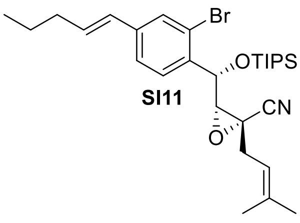

SI11 (0.166 g, 0.304 mmol, 41%, *E*/*Z* ≈ 90:10)[4] was synthesized in analogous manner starting from **SI9** (0.407 g, 0.738 mmol) and was obtained as colorless oil.

^{1}H-NMR ($CDCl_3$, 400 MHz): δ = 7.54 (d, 1H, *J* = 1.6 Hz, BrC_q-*C*H), 7.50 (d, 1H, *J* = 8.1 Hz, pentenyl-C_q-CH-C*H*), 7.32 (dd, 1H, *J* = 8.1, 1.6 Hz, pentenyl-C_q-C*H*-CH), 6.36–6.23 (m, 2H, CH_2=CH_2), 5.14 (d, 1H, *J* = 7.6 Hz, C*H*-OTIPS), 5.05 (m, 1H, Me_2C=C*H*), 3.38 (d, 1H, *J* = 7.7 Hz, epoxy-C*H*), 2.55–2.35 (m, 2H, Me_2C=CH-CH_2), 2.25–2.15 (m, 2H, CH_2-CH=CH), 1.69 (s, 3H, *E*-CH_3), 1.55 (s, 3H, *Z*-CH_3), 1.55–1.45 (m, 2H, CH_3-CH_2), 1.19–0.92 (m, 24H, TIPS-*H* and CH_3-CH_2); **^{13}C-NMR** ($CDCl_3$, 100 MHz): δ = 140.0 (pentenyl-C_q), 138.6 ($C_q$$Me_2$), 137.6 (Br$C_q$-$C_q$), 133.4 ($CH_2$-*C*H=CH), 130.6 (Br$C_q$-*C*H), 130.0 (pentenyl-$C_q$-CH-C*H*), 128.2 ($CH_2$-CH=*C*H), 125.3 (pentenyl-C_q-C*H*-CH), 122.1 (BrC_q), 117.5 (*C*N), 114.6 (*C*H=CMe$_2$), 73.5 (*C*H-OTIPS), 65.9 (epoxy-*C*H), 53.8 (epoxy-C_q), 35.2 (*C*H_2-CH=CH), 33.1 (Me_2C=CH-*C*H_2), 25.9 (*E*-*C*H_3), 22.5 (CH_3-*C*H_2), 18.2 (*Z*-*C*H_3), 18.0/17.9, 13.9 (CH(CH_3)$_2$), 12.4 (*C*H(CH_3)$_2$); **HRMS (ESI)** m/z calcd. for $C_{29}H_{45}BrNO_2Si$: 546.2397 $(M+H)^+$; $)^+$; found: no ionisation was observed; **R$_f$** (*n*-heptane/ethyl acetate 20:1): 0.49; **Specific rotation** was not determined due to *E*/*Z*-mixture and significant amount of impurities.[4]

***ent*-SI10** (0.122 g, 0.223 mmol, 48%, *E*/*Z* ≈ 93:7)[5] and ***ent*-SI11** (0.217 g, 0.400 mmol, 68%, *E*/*Z* ≈ 94:6)[6] were synthesized in analogous manner starting from ***ent*-SI8** (0.255 g, 0.462 mmol) and ***ent*-SI9** (0.320 g, 0.580 mmol). The NMR data are identical to the ones reported for **SI10** and **SI11**.

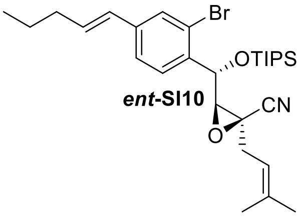

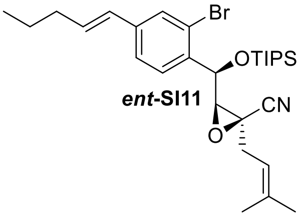

For ***ent*-SI10**: **HRMS (ESI)** m/z calcd. for $C_{29}H_{45}BrNO_2Si$: 546.2397 $(M+H)^+$; found: no ionisation was observed; **R$_f$** (*n*-heptane/ethyl acetate 20:1): 0.49; **Specific rotation** was not determined due to *E*/*Z*-mixture and significant amount of impurities.[5]

For ***ent*-SI11**: **HRMS (ESI)** m/z calcd. for $C_{29}H_{45}BrNO_2Si$: 546.2397 $(M+H)^+$; $)^+$; found: no ionisation was observed; **R$_f$** (*n*-heptane/ethyl acetate 20:1): 0.49; **Specific rotation** was not determined due to *E*/*Z*-mixture and significant amount of impurities.[6]

---

[4] The impurity was estimated to approx. 17% and could not be fully separated from the inseparable *E*/*Z*-mixture at this stage.
[5] The impurity was estimated to approx. 8% and could not be fully separated from the inseparable *E*/*Z*-mixture at this stage.
[6] The impurity was estimated to approx. 6% and could not be fully separated from the inseparable *E*/*Z*-mixture at this stage.

(1a*R*,7*R*,7a*R*)-7-Hydroxy-1a-(3-methylbut-2-en-1-yl)-4-((*E*)-pent-1-en-1-yl)-7,7a-dihydronaphtho[2,3-b]oxiren-2(1a*H*)-one (35):

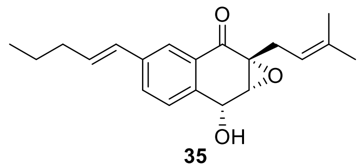

35

The halogen-lithium exchange was carried out in a moisture-free glassware under argon atmosphere. To a solution of nitrile **SI10** (0.080 g, 0.15 mmol, 1.00 eq.) in anhydrous THF (15 mL), prediluted *n*-BuLi solution (0.14 mmol, 1.00 eq.) was added dropwise at −100°C.[7] The obtained orange solution was stirred for 10 min at −100 ºC and for 5 min without cooling bath. For hydrolysis of the intermediate imine, aqueous citric acid (10%, 80 mL) was added and the mixture was extracted with ethyl acetate (100 mL and 50 mL). The combined organic layers were washed with saturated aqueous $NaHCO_3$ (80 mL) as well as with saturated aqueous NaCl (80 mL), dried over $MgSO_4$, filtered, and concentrated under reduced pressure. The crude product was purified by flash column chromatography (0–3% ethyl acetate in *n*-heptane), to give TIPS-protected **35** (0.059 g, 0.13 mmol) which was directly deprotected in the next step.

In a 50 mL falcon tube, glacial acetic acid (0.011 mL, 0.18 mmol, 1.40 eq.) followed by TBAF in THF (1 M in THF, 0.152 mL, 1.52 mmol, 1.20 eq.) were added to a solution of TIPS-protected **35** in THF (5 mL). After 1 h, a second portion of TBAF (0.40 eq.) and glacial acetic acid (0.60 eq.) and after further 1 h, a third portion of TBAF (0.50 eq.) and glacial acetic acid (0.70 eq.) were added to complete conversion of the starting material which was monitored by TLC. The reaction mixture was allowed to stir overnight at room temperature and was then loaded on silica and filtered through a small silica column (25% ethyl acetate in *n*-heptane). The product containing fractions were collected, concentrated *in vacuo* and the residue was purified *via* flash column chromatography (0–35% ethyl acetate in *n*-heptane) to give **35** (0.027 g, 0.085 mmol, 58% over two steps, *E*/*Z* ≈ 89:11) as colorless oil.

^{1}H-NMR ($CDCl_3$, 400 MHz): δ = 7.86–7.83 (m, 1H, pentenyl-C_q-C*H*-C_q), 7.59–7.55 (m, 2H, pentenyl-C_q-CH-C*H* and pentenyl-C_q-C*H*-CH), 6.43–6.25 (m, 2H, C*H*$_2$=C*H*$_2$), 5.10 (m, 1H, Me_2C=C*H*), 5.08–5.01 (m, 1H, C*H*-OH), 3.83 (d, 1H, *J* = 2.2 Hz, epoxy-C*H*), 2.92 (dd, 1H, *J* = 15.3, 7.9 Hz, Me_2C=CH-C*H*$_2$), 2.61 (dd, 1H, *J* = 15.4, 7.0 Hz, Me_2C=CH-C*H*$_2$), 2.43 (s, br, 1H, O*H*), 2.20 (m, 2H, C*H*$_2$-CH=CH), 1.73 (s, 3H, *E*-C*H*$_3$), 1.68 (s, 3H, *Z*-C*H*$_3$), 1.50 (m, 2H, CH_3-C*H*$_2$), 0.95 (t, 3H, *J* = 7.4 Hz, C*H*$_3$-CH_2); **^{13}C-NMR** ($CDCl_3$, 100 MHz): δ = 194.2 (*C*=O), 138.5 (pentenyl-*C*$_q$), 138.1 (*C*$_q$-CH-OH), 136.4 (*C*$_q$$Me_2$), 133.1 ($CH_2$-*C*H=CH), 131.7 (pentenyl-C_q-*C*H-CH), 129.1 (Ar-*C*$_q$-CO), 128.6 (CH_2-CH=*C*H), 127.4 (pentenyl-C_q-CH-*C*H), 124.6 (pentenyl-C_q-*C*H-C_q), 116.6 (*C*H=CMe_2), 66.3 (*C*H-OH), 62.5 (epoxy-*C*$_q$), 59.6 (epoxy-*C*H), 35.2 (*C*H$_2$-CH=CH), 26.8 (Me_2C=CH-*C*H$_2$), 26.0 (*E*-*C*H$_3$), 22.5 (CH_3-*C*H$_2$), 18.2 (*Z*-*C*H$_3$), 13.8 (*C*H$_3$-CH_2); **HRMS (ESI)** m/z calcd. for $C_{20}H_{25}O_3$: 313.1798 $(M+H)^+$; found: 313.1800 $(M+H)^+$; **R_f** (*n*-heptane/ethyl acetate 2:1): 0.41; **Specific rotation** $[\alpha]_D^{24.0}$ = +212.9 (c = 0.63; $CHCl_3$).

(1a*R*,7*S*,7a*R*)-7-Hydroxy-1a-(3-methylbut-2-en-1-yl)-4-((*E*)-pent-1-en-1-yl)-7,7a-dihydronaphtho[2,3-b]oxiren-2(1a*H*)-one (36):

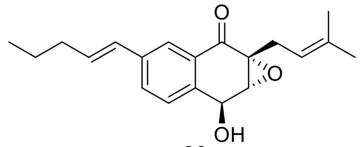

36

36 (0.017 g, 0.054 mmol, 31% over two steps, *E*/*Z* ≈ 90:10) was synthesized in analogous manner starting from **SI11** (0.093 g, 0.17 mmol) and was obtained as colorless oil.

^{1}H-NMR ($CDCl_3$, 400 MHz): δ = 7.74 (d, 1H, *J* = 1.8 Hz, pentenyl-C_q-C*H*-C_q), 7.51 (dd, 1H, *J* = 8.0, 1.9 Hz, pentenyl-C_q-C*H*-CH), 7.33 (d, 1H, *J* = 7.9 Hz, pentenyl-C_q-CH-C*H*), 6.41–6.21 (m, 2H, C*H*$_2$=C*H*$_2$), 5.17 (d, 1H, *J* = 2.0 Hz, C*H*-OH), 5.13 (m, 1H, Me_2C=C*H*), 3.82 (d, 1H, *J* = 2.3 Hz, epoxy-C*H*), 2.90 (dd, 1H, *J* = 15.4, 7.8 Hz, Me_2C=CH-C*H*$_2$), 2.66 (dd, 1H, *J* = 15.4, 6.7 Hz, Me_2C=CH-C*H*$_2$), 2.43 (s, br, 1H, O*H*), 2.20 (m, 2H, C*H*$_2$-CH=CH), 1.73 (s, 3H, *E*-C*H*$_3$), 1.69 (s, 3H, *Z*-C*H*$_3$), 1.50 (m, 2H, CH_3-C*H*$_2$), 0.96 (t, 3H, *J* = 7.4 Hz, C*H*$_3$-CH_2); **^{13}C-NMR** ($CDCl_3$, 100 MHz): δ = 195.2 (*C*=O), 139.5 (pentenyl-*C*$_q$), 137.8 (*C*$_q$-CH-OH), 136.3 (*C*$_q$$Me_2$), 133.5 ($CH_2$-*C*H=CH), 131.7 (pentenyl-C_q-*C*H-CH), 130.0 (pentenyl-C_q-CH-*C*H), 129.8 (Ar-*C*$_q$-CO), 128.6 (CH_2-CH=*C*H), 124.7 (pentenyl-C_q-*C*H-C_q), 116.7 (*C*H=CMe_2),

[7] A 2.5 M solution of *n*-BuLi in hexane from a commercially supplier was diluted by a factor of four at −78 °C with anhydrous THF for a better handling. For the −100 °C cooling bath a mixture of ethanol and liquid N_2 was used.

66.9 (*C*H-OH), 60.7 (epoxy-C_q), 60.2 (epoxy-*C*H), 35.2 (*C*H_2-CH=CH), 26.7 (Me_2C=CH-*C*H_2), 26.0 (*E*-*C*H_3), 22.5 (CH_3-*C*H_2), 18.2 (*Z*-*C*H_3), 13.9 (*C*H_3-CH_2); **HRMS (ESI)** m/z calcd. for $C_{20}H_{25}O_3$: 313.1798 $(M+H)^+$; found: 313.1802 $(M+H)^+$; **R_f** (*n*-heptane/ethyl acetate 2:1): 0.38; **Specific rotation** $[\alpha]_D^{24.0}$ = +145.3 (c = 0.36; $CHCl_3$).

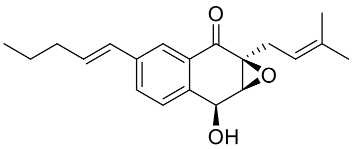

***ent*-35**

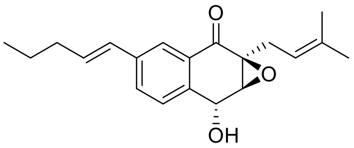

***ent*-36**

***Ent*-35** (0.007 g, 0.02 mmol, 16% over two steps, *E*/*Z* ≈ 93:7) and ***ent*-36** (0.009 g, 0.03 mmol, 19% over two steps, *E*/*Z* ≈ 99:1) were synthesized in analogous manner starting from ***ent*-SI10** (0.074 g, 0.13 mmol) and ***ent*-SI11** (0.081 g, 0.15 mmol). The NMR data are identical to the ones reported for **35** and **36**.

For ***ent*-35**: **HRMS (ESI)** m/z calcd. for $C_{20}H_{25}O_3$: 313.1798 $(M+H)^+$; found: 313.1808 $(M+H)^+$; **R_f** (*n*-heptane/ethyl acetate 2:1): 0.41; **Specific rotation** $[\alpha]_D^{21.9}$ = −149.6 (c = 0.35; $CHCl_3$).

For ***ent*-36**: **HRMS (ESI)** m/z calcd. for $C_{20}H_{25}O_3$: 313.1798 $(M+H)^+$; found: 313.1808 $(M+H)^+$; **R_f** (*n*-heptane/ethyl acetate 2:1): 0.38; **Specific rotation** $[\alpha]_D^{21.9}$ = −191.0 (c = 0.60; $CHCl_3$).

1.2. Confirmation of the absolute stereochemistry of (+)-floyocidin B (4) by chiral HPLC

Operator:HPLC-A Timebase:HPLC_1 Sequence:Y.KLEINER

Page 1-1
1.12.2020 2:21 PM

106 SF008-B_NP

CHIRALPAK IC | 1ml/min. | 85% Hexan, 15% IPA

Sample Name:	**SF008-B_NP**	*Injection Volume:*	**5,0**
Vial Number:	**108**	*Channel:*	**UV_VIS_1**
Sample Type:	**unknown**	*Wavelength:*	**254**
Control Program:	**Analyse03_F10_A85_B00_C15**	*Bandwidth:*	**1**
Quantif. Method:	**default**	*Dilution Factor:*	**1,0000**
Recording Time:	**1.12.2020 13:28**	*Sample Weight:*	**1,0000**
Run Time (min):	**20,00**	*Sample Amount:*	**1,0000**

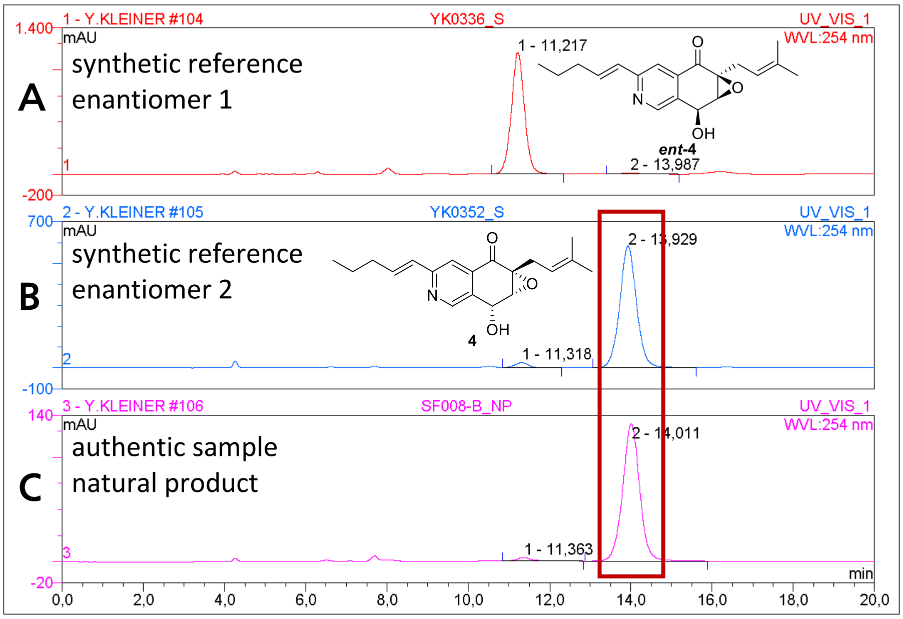

No.	Ret.Time min	Peak Name	Height mAU	Area mAU*min	Rel.Area %	Amount	Type
1	11,36	n.a.	3,245	1,670	2,54	n.a.	BMB
2	14,01	n.a.	131,368	63,949	97,46	n.a.	BMB
**Total:**			134,613	65,618	100,00	0,000	

default/Integration

Chromeleon (c) Dionex 1996-2001
Version 6.80 SR15 Build 4656 (243203)

Figure S1. **Chiral HPLC analysis for confirmation of correct absolute configuration *via* comparison of isolated NP (C) with synthesized enantiomers (A and B).**

2. Biological and medicinal-chemical profiling

2.3. Antimicrobial activities

Growth inhibitory potency was determined by micro-broth dilution assays. Each compound was dissolved in DMSO and tested in triplicate. For assays against *E. coli* ATCC25922, *P. aeruginosa* ATCC27859 and *S. aureus* ATCC25923, pre-cultures were incubated overnight at 37 °C and 180 rpm before being diluted to a concentration of $5 \cdot 10^5$ cells/mL in cation adjusted Mueller Hinton II medium (CAMH). The cell suspension for *E. coli* ATCC25922 was additionally prepared in CAMH supplemented with 44 mM sodium bicarbonate. Dilution series of rifampicin (Sigma), tetracycline (Sigma), and gentamycin (Sigma) were used as positive controls (64-0.03 µg/mL). After incubation for 18 h (37 °C, 180 rpm, 85% relative humidity (r.H.)), cell growth was assessed by measuring the turbidity with a microplate spectrophotometer at 600 nm (LUMIstar® Omega BMG Labtech). The pre-culture of *M. smegmatis* ATCC607 was incubated in Brain-Heart Infusion broth for 48 h at 37 °C and 180 rpm. The assay cell density was adjusted as described above in CAMH. Isoniazid was used instead of gentamycin as a third positive control. *M. smegmatis* assays were incubated for 48 h (37 °C, 180 rpm, 85% r.H.) before cell viability was assessed *via* ATP quantification (BacTiter-Glo™, Promega) according to the manufacturer's instructions. Assays against *M. tuberculosis* H37Rv were carried out in the L3 approved laboratories of Evotec (Lyon, France). Tests were done in Middlebrook 7H9 broth medium and incubated at 37 °C and 5% CO_2 for 6 days. Bacterial viability was determined by CellTiter-Blue™ (Promega) according to the manufacturer's instructions. *C. albicans* FH2173 was incubated for 48 h at 28 °C and 180 rpm and diluted to $1 \cdot 10^6$ cells/mL in CAMH. Assays were incubated for 48 h at 37 °C and 180 rpm. Tebuconazole (Cayman Chemical Company), amphotericin B (Sigma), and nystatin (Sigma) were used as positive control (64–0.03 µg/mL). Readout was done by ATP quantification. Compound showing at least 80% relative growth inhibition of the indicator strain were considered bioactive at the respective concentration. Compounds exhibiting no activity at 128 µg/mL were scored "not active" (NA).

Table S3. **Bioactivity data of synthesized NP and SAR-compounds against several pathogens.**[a]

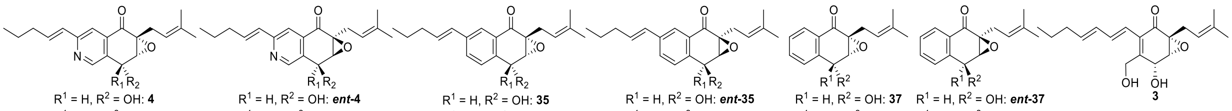

R^1 = H, R^2 = OH: **4**; R^1 = OH, R^2 = H: **27**
R^1 = H, R^2 = OH: ***ent*-4**; R^1 = OH, R^2 = H: ***ent*-27**
R^1 = H, R^2 = OH: **35**; R^1 = OH, R^2 = H: **36**
R^1 = H, R^2 = OH: ***ent*-35**; R^1 = OH, R^2 = H: ***ent*-36**
R^1 = H, R^2 = OH: **37**; R^1 = OH, R^2 = H: **38**
R^1 = H, R^2 = OH: ***ent*-37**; R^1 = OH, R^2 = H: **2**
3

	MIC [µg/mL]												
**Compound**	**4**	**27**	***ent*-4**	***ent*-27**	**35**	**36**	***ent*-35**	***ent*-36**	**37**	**38**	***ent*-37**	**2**	**3**
E. coli **ATCC25922**	NA	NA	NA	NA	NA	NA	NA	NA	NA	NA	NA	NA	NA
E. coli **ATCC25922 MHC**	NA	NA	NA	NA	NA	NA	NA	NA	NA	NA	128	NA	NA
P. aeruginosa **ATCC27853**	NA	NA	NA	NA	NA	NA	NA	NA	NA	NA	NA	NA	NA
S. aureus **ATCC25923**	NA	NA	NA	NA	NA	NA	NA	NA	NA	NA	NA	NA	64
M. catarrhalis **ATCC25238I**	16	16–8	16	8	64	8	32	16–8	128	128	64–32	64	32–16
C. albicans **FH2173**	NA	NA	NA	NA	NA	NA	NA	NA	128	NA	128	128	NA

[a] NA = not active.

2.4. Assay descriptions for the profiling of all four stereoisomers of (+)-floyocidin B and (−)-avicennone C,[1] the avicennone C-floyocidin B hybrids 35, *ent*-35, 36 and *ent*-36 as well as the natural product (+)-floyocidin A.

2.4.1. Determination of $logD_{7.4}$

LogD values at pH 7.4 were determined by a standardized HPLC method derived from by Genieser *et al*.[8] The calculation of the logD value for measured compounds is performed by comparison of the retention times with standard compounds of known distribution coefficients between 1-octanol and water at pH 7.4.

2.4.2. Microsomal stability

Pooled liver microsomes are purchased. Microsomes are stored at –80 °C prior to use. Microsomes (final protein concentration 0.5 mg/mL), 0.1 M phosphate buffer pH 7.4, and test compound (final substrate concentration 1 µM; final DMSO concentration 0.25%) are pre-incubated at 37 °C prior to the addition of NADPH (final concentration 1 mM) to initiate the reaction. A minus cofactor control incubation is included for each compound tested where 0.1 M phosphate buffer pH 7.4 is added instead of NADPH. Two control compounds are included. All incubations are performed singularly for each test compound. Each compound is incubated for 0, 5, 15, 30, and 45 min. The control is incubated for 45 min only. The reactions are stopped by transferring incubate into acetonitrile at the appropriate time points, in a 1:3 ratio. The termination plates are centrifuged at 3,000 rpm for 20 min at 4 °C to precipitate the protein.

From a plot of ln peak area ratio (compound peak area/internal standard peak area) against time, the gradient of the line is determined. Subsequently, half-life and intrinsic clearance are calculated using the equations below:

Elimination rate constant (k) = (- gradient)

$$\text{Half-life } (t_{½})(\text{min}) = \frac{0.693}{k}$$

$$\text{Intrinsic clearance } (CL_{int})(\mu L/\text{min/mg protein}) = \frac{V \text{ x } 0.693}{t_{½}}$$

where V = Incubation volume (µL)/Microsomal protein (mg)

Relevant control compounds are assessed, ensuring intrinsic clearance values fall within the specified limits (if available).

2.4.3. Cellular permeability assay utilizing Caco2 cell line

Caco-2 cells obtained from the ATCC are used between passage numbers 40 - 60. Cells are seeded onto Transwell plates at 1 x 10^5 cells/cm^2. The cells are cultured in DMEM and media is changed every two or three days. On day 20 the permeability study is performed. Cell culture and assay incubations are carried out at 37 °C in an atmosphere of 5% CO_2 with a relative humidity of 95%. On the day of the assay, the monolayers are prepared by rinsing both apical and basolateral surfaces twice with Hanks Balanced Salt Solution (HBSS) at the desired pH warmed to 37 °C. Cells are then incubated with HBSS at the desired pH in both apical and basolateral compartments for 40 min to stabilize physiological parameters. The dosing solutions are prepared by diluting test compound with assay buffer to give the desired test compound incubation concentration (10 µM). At 120 min the apical compartment inserts and the companion plates are separated and apical and basolateral samples diluted for analysis. Test compound permeability is assessed in duplicate. Test and control compounds are quantified by LC-MS/MS cassette analysis using a 7-point calibration with appropriate dilution of the samples. The starting concentration (C_0) is determined from the dosing solution and the experimental recovery calculated

[8] Krass, J. D., Jastorff, B., and Genieser, H.-G. *Anal. Chem.* **1997**, *69,* 2575–2581.

from C_0 and both apical and basolateral compartment concentrations. Four control compounds are screened alongside the test compounds.

The permeability coefficient (P_{app}) for each compound is calculated from the following equation:

$$P_{app} = \left(\frac{dQ/dt}{C_0 \times A}\right)$$

Where dQ/dt is the rate of permeation of the drug across the cells, C_0 is the donor compartment concentration at time zero and A is the area of the cell monolayer. C_0 is obtained from analysis of the dosing solution.

For bi-directional experiments, an efflux ratio (ER) is calculated from mean A-B and B-A data. This is derived from:

$$ER = \frac{P_{app(B-A)}}{P_{app(A-B)}}$$

Table S4. **Results for cellular permeability assays utilizing Caco2 cell lines with standard deviations (SD) for P_{app}.**

	P_{app} [10^{-7} cm s^{-1}] (recovery [%])			
	A2B		+ Elacridar	
**3**	(a)		(b)	
**4**	41.1 (10.1)	SD: 3.78	56.2 (14.2)	SD: 6.55
**27**	117 (42.1)	SD: 3.47	87.7 (38.5)	SD: 3.40
***ent*-4**	73.5 (23.4)	SD: 9.38	46.7 (28.7)	SD: 3.12
***ent*-27**	40.5 (8.69)	SD: 3.56	64.4 (15.3)	SD: 3.71
**35**	135 (76.0)	SD: 26.2	181 (29.6)	(c)
**36**	(a) (16.1)		(a) (19.9)	
***ent*-35**	162 (37.2)	(c)	(a) (46.1)	
***ent*-36**	8.55 (14.3)	SD: .16	12.4 (12.0)	SD: 9.83
**37**	326 (74.8)	SD: 22.5	279 (80.9)	SD: 5.39
**38**	620 (127)	SD: 17.4	331 (132)	SD: 156
***ent*-37**	360 (81.3)	SD: 23.6	300 (74.6)	SD: 6.89
**2**	407 (86.7)	SD: 22.3	393 (83.4)	SD: 9.70

[a] No data obtained. [b] Not determined. [c] Only one value obtained.

2.4.4. Cytotoxicity on THP1 and HepG2 cells

After 4 hours of HepG2 cell line (provided by ATCC) plating at 2.500 cells /well in 384-w plates, compounds were added to the cells. After an incubation at 37 °C for 40 h, the CellTiterGlo assay (provided by Promega) was performed. Luminescence measurement is related to the cell viability. Compound activity is determined by using the control wells with no treatment as control (100% of growth). The assay was performed identically with THP1 cells. All incubations were performed in duplicate.

3. Biosynthetic hypothesis of floyocidins

The structure of the floyocidins indicates a polyketide synthase (PKS)-dependent biosynthesis.[9] Hence, as proposed for (+)-ambuic acid (**1**) and its derivatives,[10] the backbone structure can be build up from one acetyl-CoA (**SI12**) and six malonyl-CoA (**SI13**) building blocks (Scheme S1). The nascent linear polyketide chain **SI14** undergoes an intramolecular cyclization that can be catalyzed by a cyclase, resulting in the six-membered ring present in (+)-floyocidin A (**3**). In contrast to (+)-ambuic acid (**1**), an additional *E*-double bond in the hepta-1,3-dien-1-yl chain is located, indicating ketoreduction and dehydration taking place at the second acetate unit, yielding this C=C double bond. The prenyl side chain is presumably acetyl-CoA-derived, too. It can be deduced from dimethylallyl pyrophosphate (DMAPP, **SI16**), which is an isomer of isopentenyl pyrophosphate (IPP). Furthermore, an oxygenase introduces the keto group to the ring. The epoxide of **3** is expected to result from an epoxidase-catalyzed oxidation of the corresponding double bond. The order of the decorating steps remains unclear until now. The primary hydroxyl group of **3** represents the position of attachment to the carrier protein during polyketide assembly.

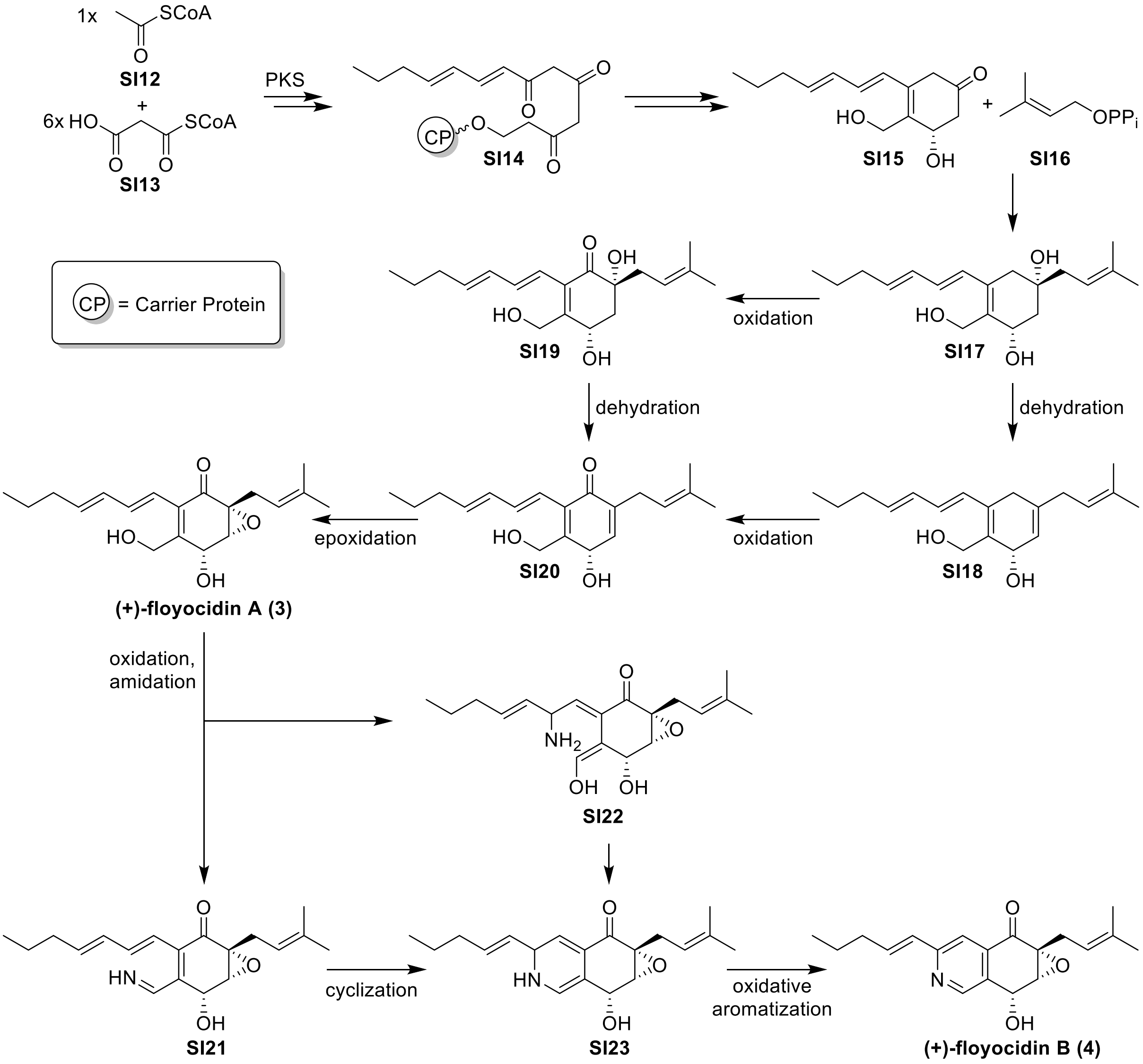

***Scheme S1*. Plausible biosynthetic pathway of (+)-floyocidin A and (+)-floyocidin B.**

[9] Yuan, C.; Ding, G.; Wang, H.-Y.; Guo, Y.-H.; Shang, H.; Ma, X.-J.; Zou, Z.-M. *BioMed Res. Int*. **2017**, 6961928.
[10] Yu, X.; Gao, Y.; Frank, M.; Mándi, A.; Kurtán, T.; Müller, W. E. G.; Kalscheuer, R.; Guo, Z.; Zou, K.; Liu, Z.; Proksch, P. *Tetrahedron* **2021**, *79*, 131876.

For the formation of the dihydroisoquinolinone scaffold of (+)-floyocidin B (**4**), a nitrogen atom has to be introduced. Hence, the putative precursor **SI23** could be accessible by a selective oxidation of the terminal hydroxyl group and followed by an amidation-cyclization cascade. Alternatively, an amidase could add an ammonia equivalent to the hepta-1,3-dien-1-yl side chain, which then could be oxidatively cyclized. Final oxidative aromatization would complete the proposed biosynthesis of (+)-floyocidin B (**4**).

4. Crystallographic data collection and refinement details

Diffraction data were collected at low temperatures (100K) using φ- and ω-scans on a BRUKER D8 Venture System equipped with dual IµS microfocus sources, a PHOTON100 detector and an OXFORD CRYOSYSTEMS 700 low temperature system. Mo-K_α radiation with a wavelength of 0.71073 Å and a collimating Quazar multilayer mirror were used.

Semi-empirical absorption corrections from equivalents were applied using SADABS.[11] The structure was solved by direct methods using SHELXT[12] and refined against F^2 on all data by full-matrix least squares using SHELXL.[13] All non-hydrogen atoms were refined anisotropically O-H hydrogen atoms were located in the Fourier difference map and set to ideal distances and C-H hydrogen atoms were positioned at geometrically calculated positions and refined using a riding model. The isotropic displacement parameters of all hydrogen atoms were fixed to 1.2x or 1.5x (CH_3 and OH hydrogens) the U_{eq} value of the atoms they are linked to. The crystallographic data were deposited with the Cambridge Crystallographic Database as 2075537 and can be obtained free of charge.[14]

The structure of **17** was solved in the orthorhombic space group $C222_1$. The asymmetric unit contains one molecule of **17**, exhibiting full molecule disorder. The disorder was modelled with the help of same distances restraints, similarity restraints on anisotropic displacement parameters, restraints to a common plane[15] and advanced rigid bond restraints.[16] Some disordered atoms with close coordinates were set to the same anisotropic displacement parameters. The disorder ratio was allowed to refine freely and converged to 0.520(2). The absolute structure was confirmed by classical Flack x (0.011(8)) and the Parsons parameter (0.014(2)).[17]

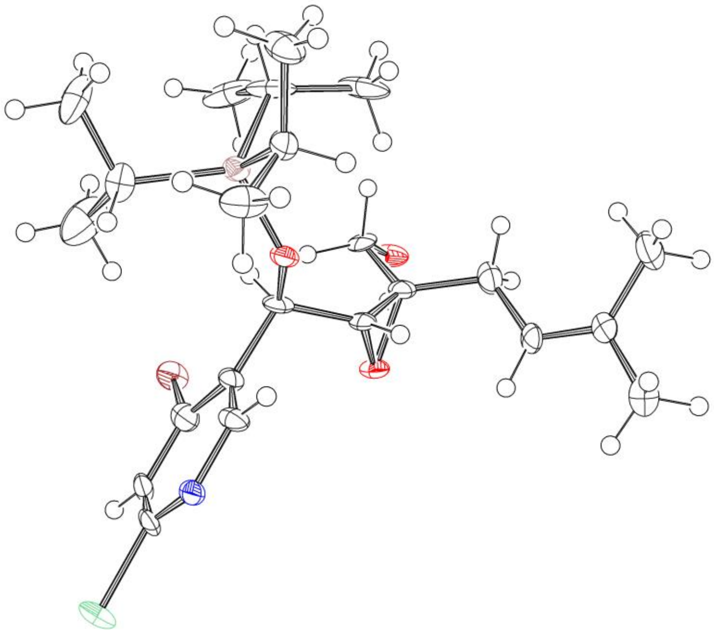

Figure S2. **Thermal ellipsoid plot of the molecular structure of 17. Thermal ellipsoid probability set to 50%, only most occupied disorder part shown.**

[11] Krause, L.; Herbst-Irmer, R.; Sheldrick, G. M.; Stalke, D. J. *Appl. Cryst.* **2015**, *48*, 3–10.
[12] Sheldrick, G. M. *Acta Cryst. A* **2015**, *71*, 3–8.
[13] Sheldrick, G. M. *Acta Cryst. C* **2015**, *71*, 3–8.
[14] https://www.ccdc.cam.ac.uk/structures/
[15] Müller, P. *Cryst. Rev.* **2009**, *15*, 57–83.
[16] Thorn, A.; Dittrich, B.; Sheldrick, G. M. *Acta Cryst. A* **2012**, *68*, 448–451.
[17] Parsons, H.; Flack, H. D.; Wagner, T. *Acta Cryst. A* **2013**, *69*, 249-259.

***Table S5*. Crystal data and structure refinement for 17.**

CCDC No	2075537	
Empirical formula	$C_{23} H_{37} Br Cl N O_3 Si$	
Formula weight	518.98	
Temperature	100(2) K	
Wavelength	0.71073 Å	
Crystal system	Orthorhombic	
Space group	$C222_1$	
Unit cell dimensions	a = 11.0712(5) Å	α= 90°.
	b = 20.0242(9) Å	β= 90°.
	c = 23.2586(11) Å	γ = 90°.
Volume	5156.2(4) Å^3	
Z	8	
Density (calculated)	1.337 Mg/m^3	
Absorption coefficient	1.768 mm^{-1}	
F(000)	2176	
Crystal size	0.309 x 0.095 x 0.072 mm^3	
Theta range for data collection	2.034 to 28.279°.	
Index ranges	-14≤ h≤ 14, -26≤ k≤ 26, -31≤ l≤ 31	
Reflections collected	178371	
Independent reflections	6417 [R(int) = 0.0474]	
Completeness to theta = 25.242°	100.0%	
Absorption correction	Semi-empirical from equivalents	
Refinement method	Full-matrix least-squares on F^2	
Data / restraints / parameters	6417 / 1397 / 529	
Goodness-of-fit on F^2	1.055	
Final R indices [I>2σ(I)]	R_1 = 0.0324, wR_2 = 0.0672	
R indices (all data)	R_1 = 0.0364, wR_2 = 0.0691	
Absolute structure parameter	0.014(2)	
Extinction coefficient	0.00150(13)	
Largest diff. peak and hole	0.737 and -0.658 e.Å^{-3}	

5. NMR spectra

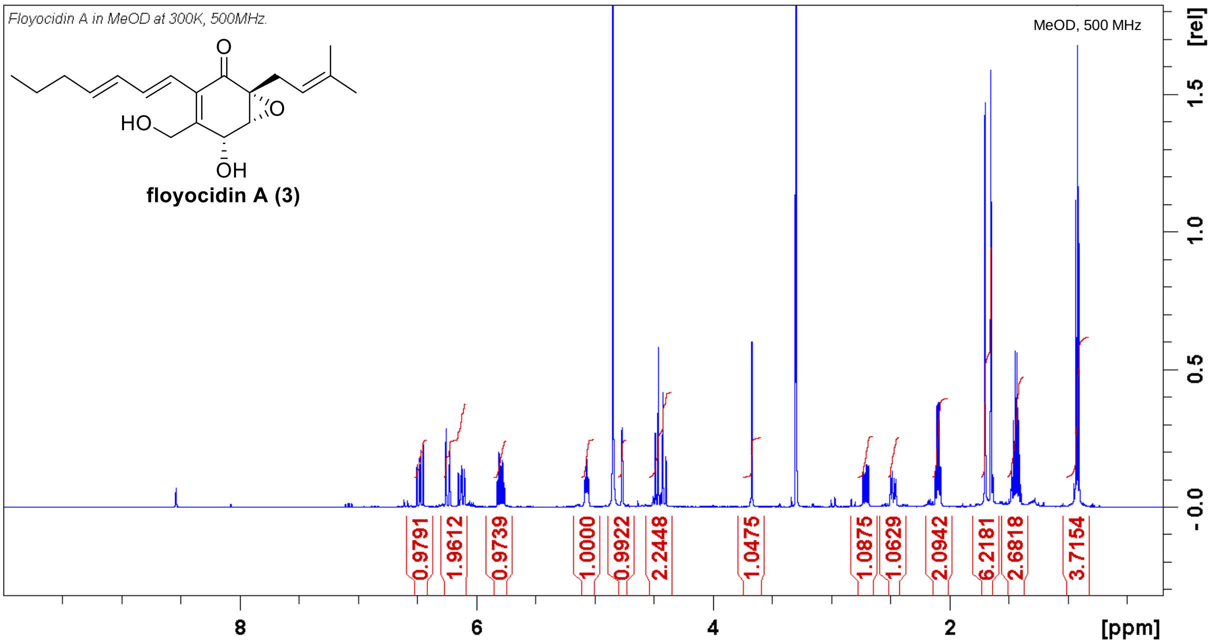

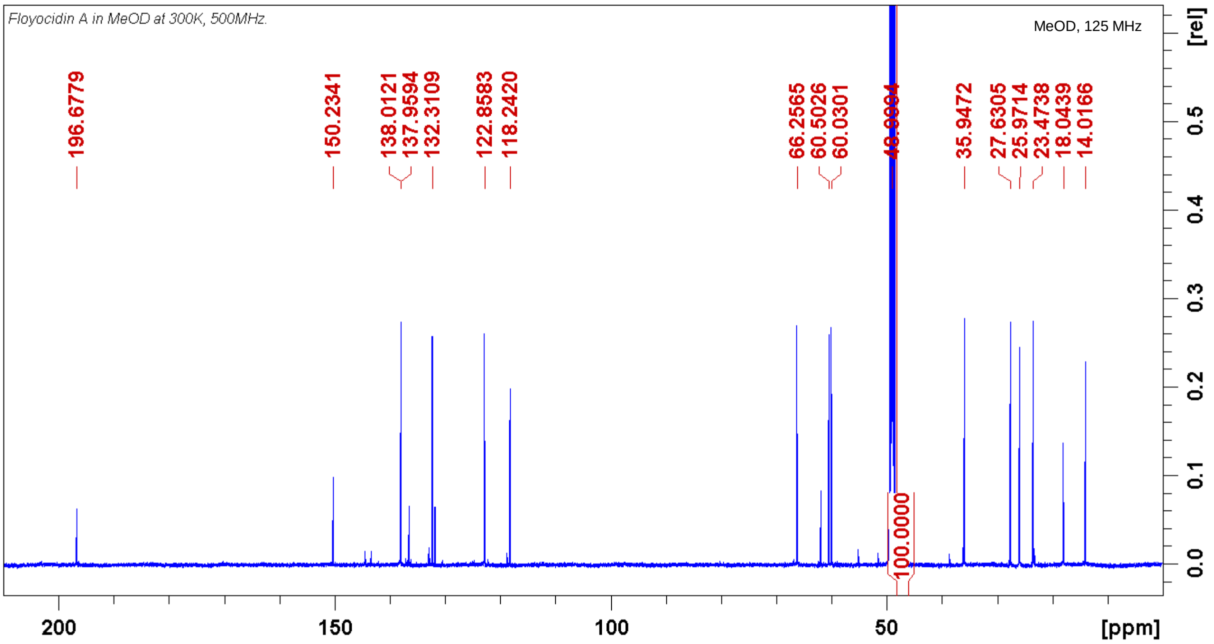

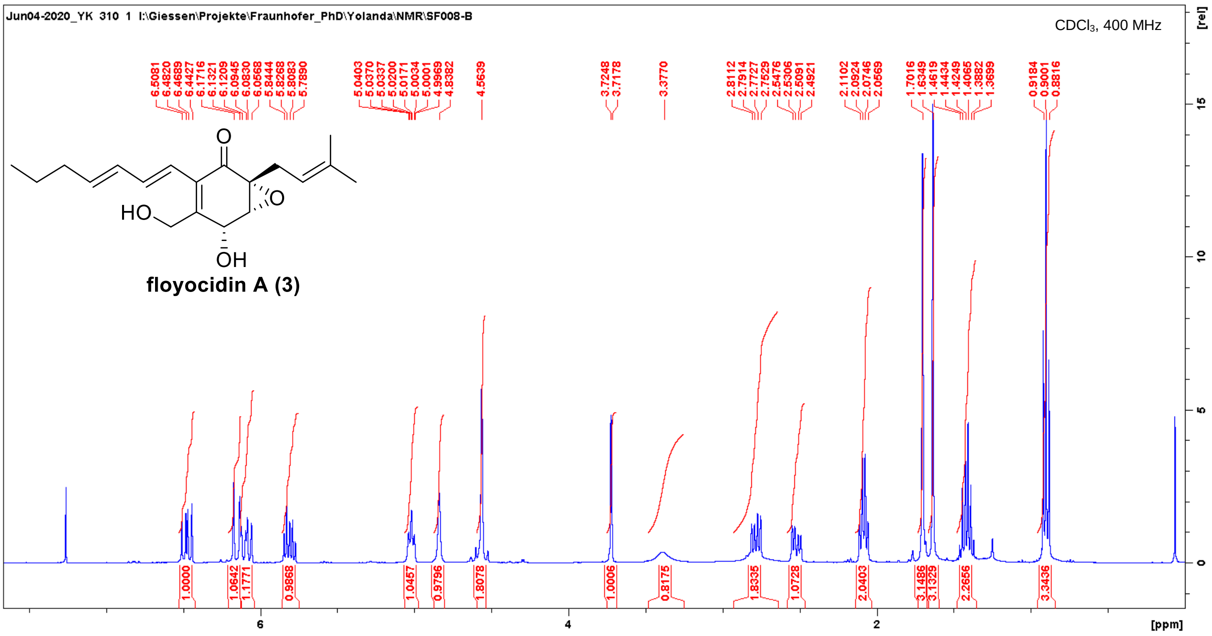
Jun04-2020_YK 310 1 I:\Giessen\Projekte\Fraunhofer_PhD\Yolanda\NMR\SF008-B
CDCl3, 400 MHz
HO
OH
O
O
floyocidin A (3)
[ppm]
[rel]

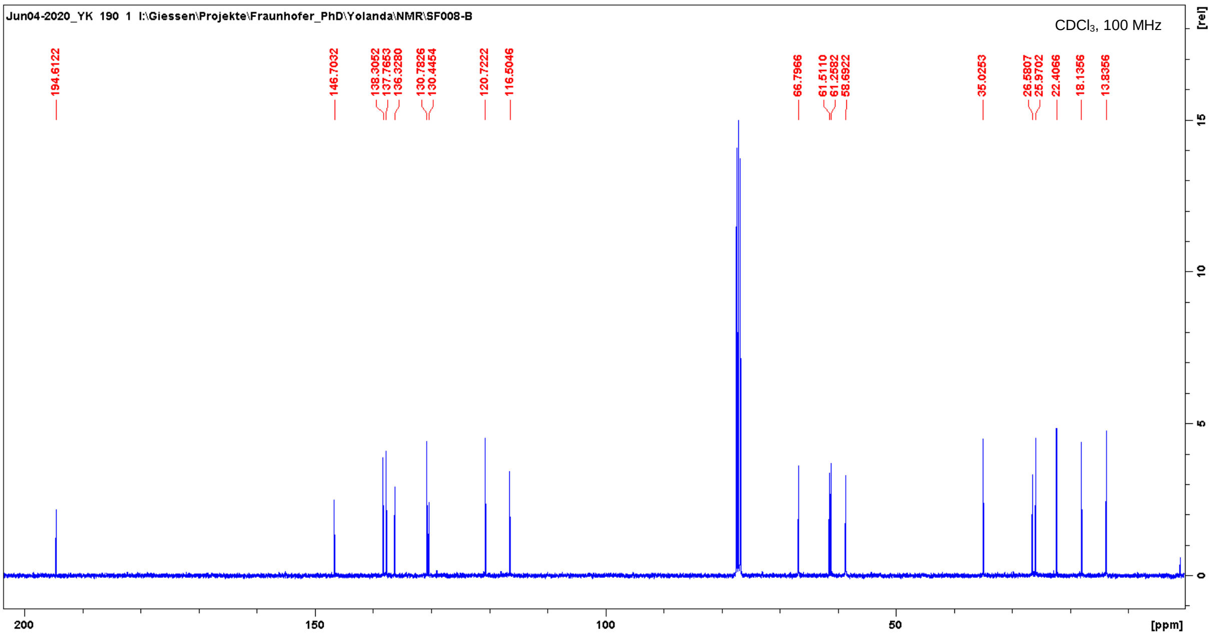
Jun04-2020_YK 190 1 I:\Giessen\Projekte\Fraunhofer_PhD\Yolanda\NMR\SF008-B
CDCl3, 100 MHz
194.6122
146.7032
138.3052
137.7653
136.3280
130.7826
130.4454
120.7222
116.5046
66.7966
61.5110
61.2582
58.6922
35.0253
26.5807
25.9702
22.4066
18.1356
13.8356
[ppm]
[rel]

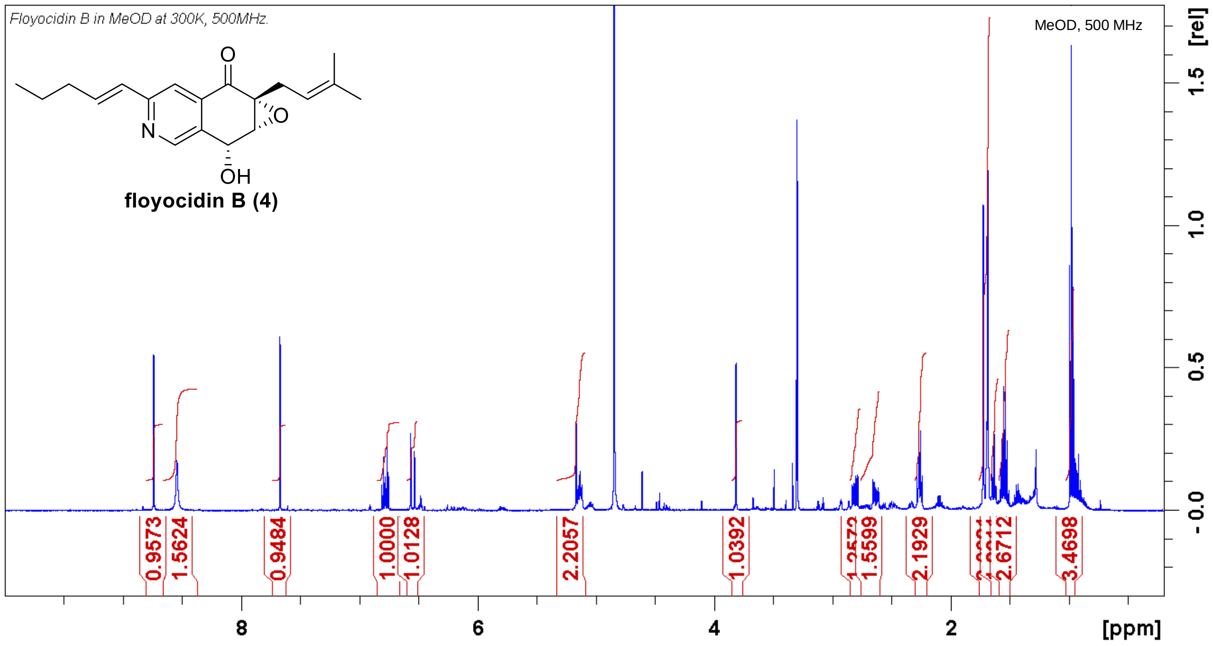
Floyocidin B in MeOD at 300K, 500MHz.
MeOD, 500 MHz
floyocidin B (4)
0.9573
1.5624
0.9484
1.0000
1.0128
2.2057
1.0392
1.5599
2.1929
2.6712
3.4698
[ppm]
[rel]

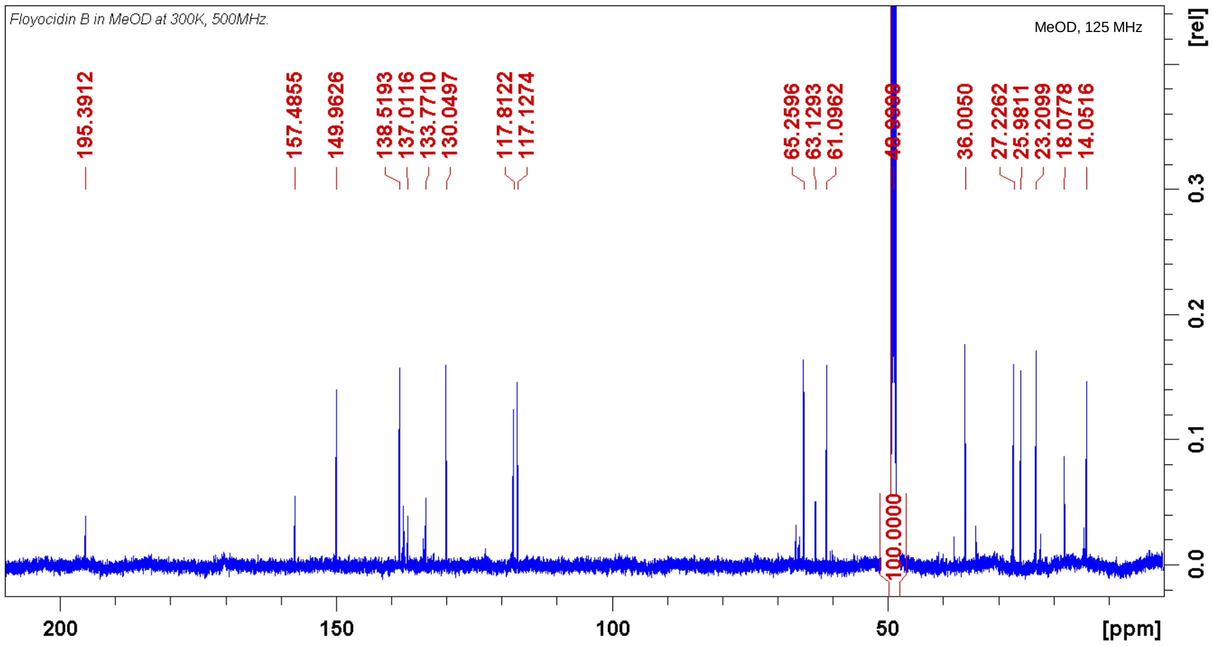
Floyocidin B in MeOD at 300K, 500MHz.
MeOD, 125 MHz
195.3912
157.4855
149.9626
138.5193
137.0116
133.7710
130.0497
117.8122
117.1274
65.2596
63.1293
61.0962
36.0050
27.2262
25.9811
23.2099
18.0778
14.0516
100.0000
[ppm]
[rel]

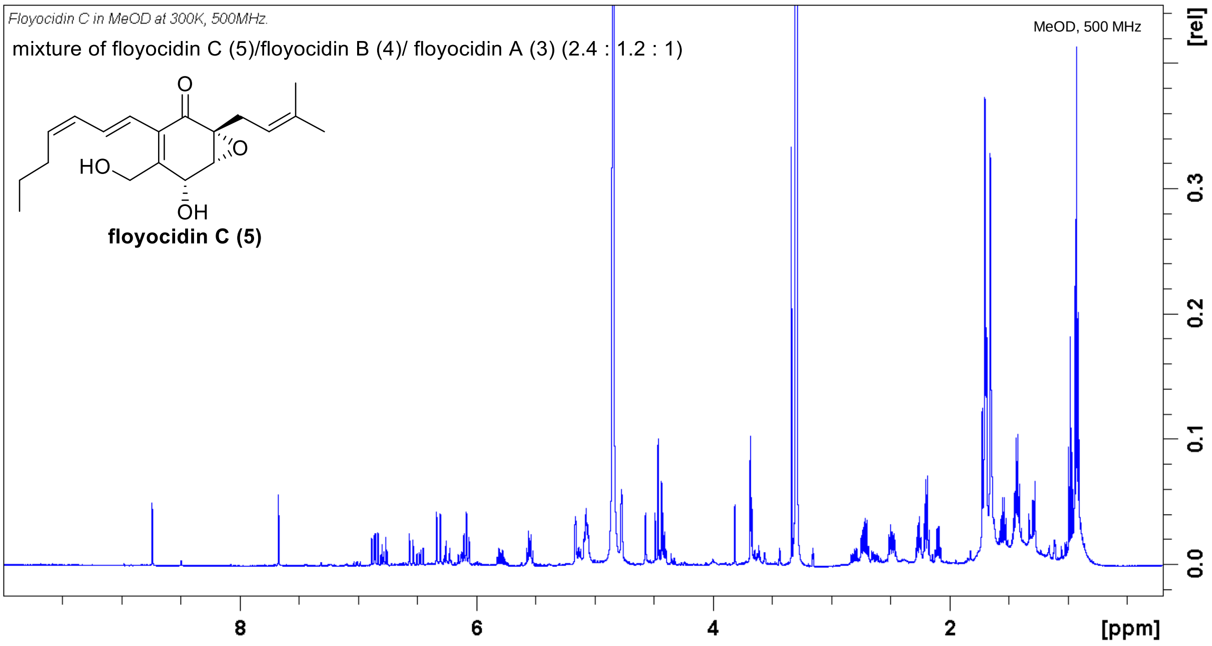
Floyocidin C in MeOD at 300K, 500MHz.
MeOD, 500 MHz
mixture of floyocidin C (5)/floyocidin B (4)/ floyocidin A (3) (2.4 : 1.2 : 1)
O
O
HO
OH
floyocidin C (5)
[rel]
0.3
0.2
0.1
0.0
8
6
4
2
[ppm]

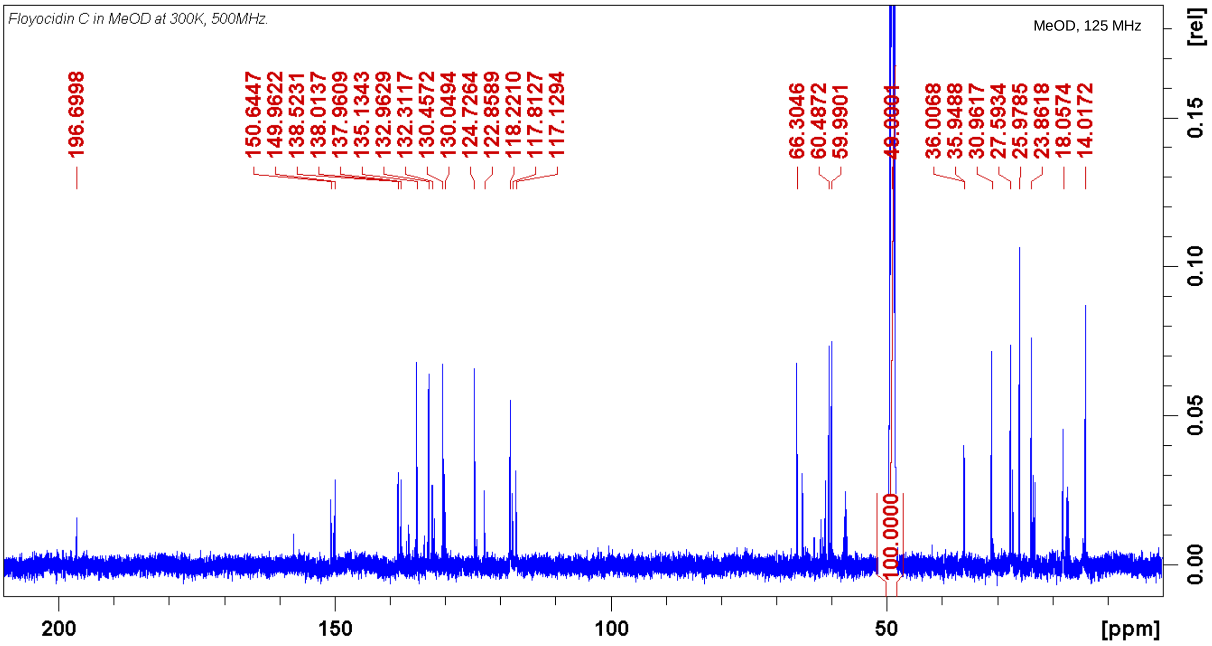
Floyocidin C in MeOD at 300K, 500MHz.
MeOD, 125 MHz
196.6998
150.6447
149.9622
138.5231
138.0137
137.9609
135.1343
132.9629
132.3117
130.4572
130.0494
124.7264
122.8589
118.2210
117.8127
117.1294
66.3046
60.4872
59.9901
49.0001
36.0068
35.9488
30.9617
27.5934
25.9785
23.8618
18.0574
14.0172
100.0000
[rel]
0.15
0.10
0.05
0.00
200
150
100
50
[ppm]

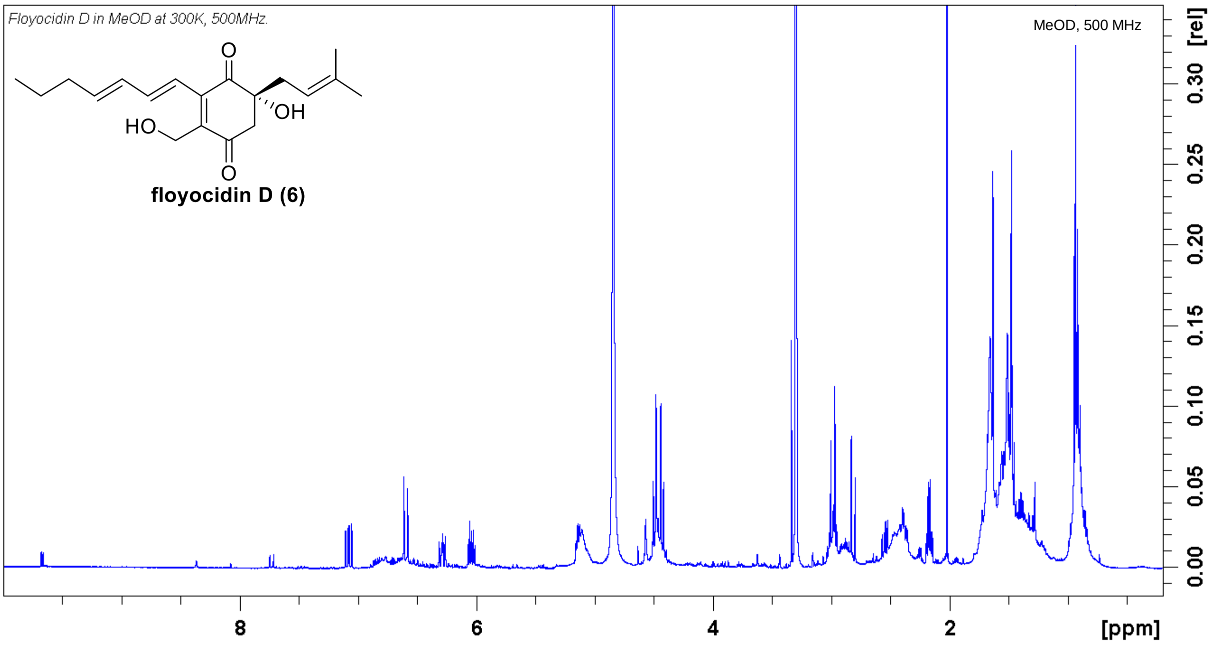

Floyocidin D in MeOD at 300K, 500MHz.
MeOD, 500 MHz
HO
OH
O
O
floyocidin D (6)
[rel]
0.00
0.05
0.10
0.15
0.20
0.25
0.30
8
6
4
2
[ppm]

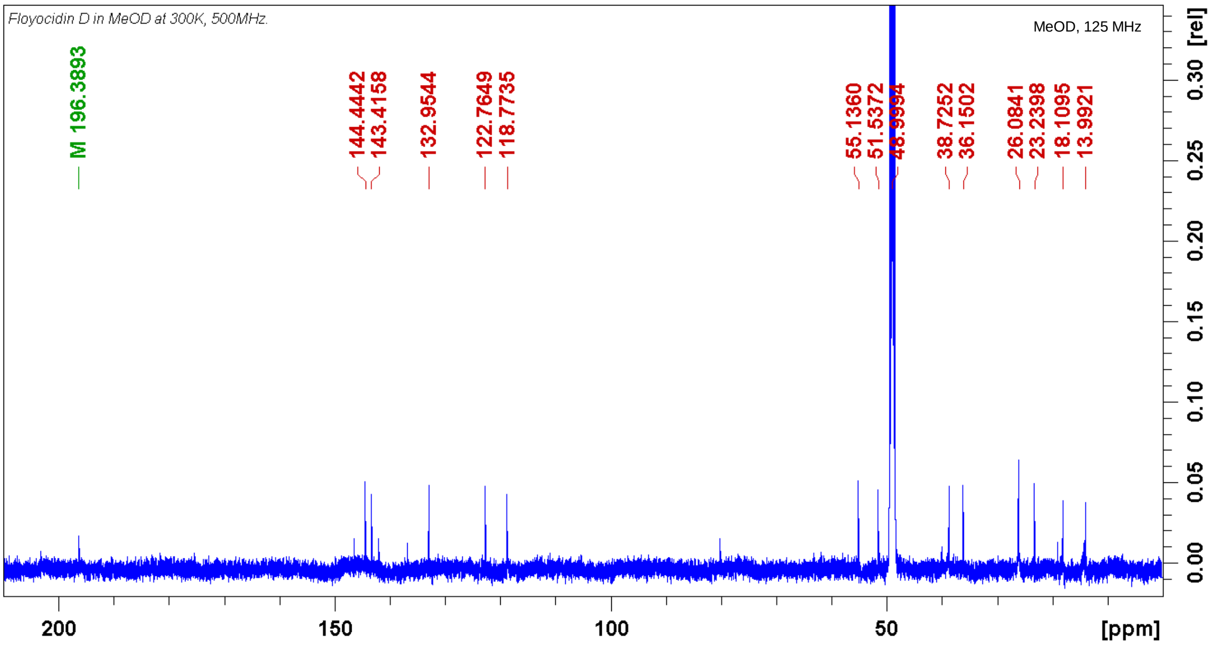

Floyocidin D in MeOD at 300K, 500MHz.
MeOD, 125 MHz
M 196.3893
144.4442
143.4158
132.9544
122.7649
118.7735
55.1360
51.5372
48.9994
38.7252
36.1502
26.0841
23.2398
18.1095
13.9921
[rel]
0.00
0.05
0.10
0.15
0.20
0.25
0.30
200
150
100
50
[ppm]

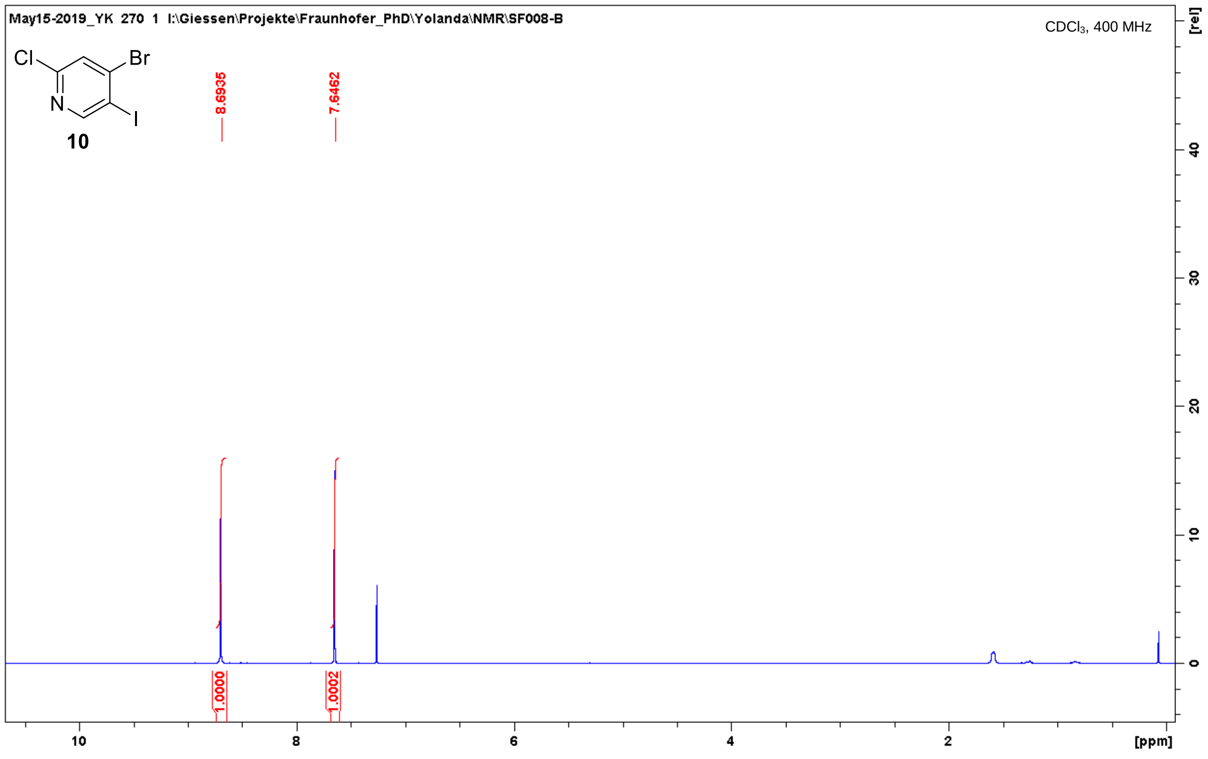
May15-2019_YK 270 1 I:\Giessen\Projekte\Fraunhofer_PhD\Yolanda\NMR\SF008-B
CDCl3, 400 MHz
Cl
Br
N
I
10
8.6935
7.6462
1.0000
1.0002
[rel]
[ppm]

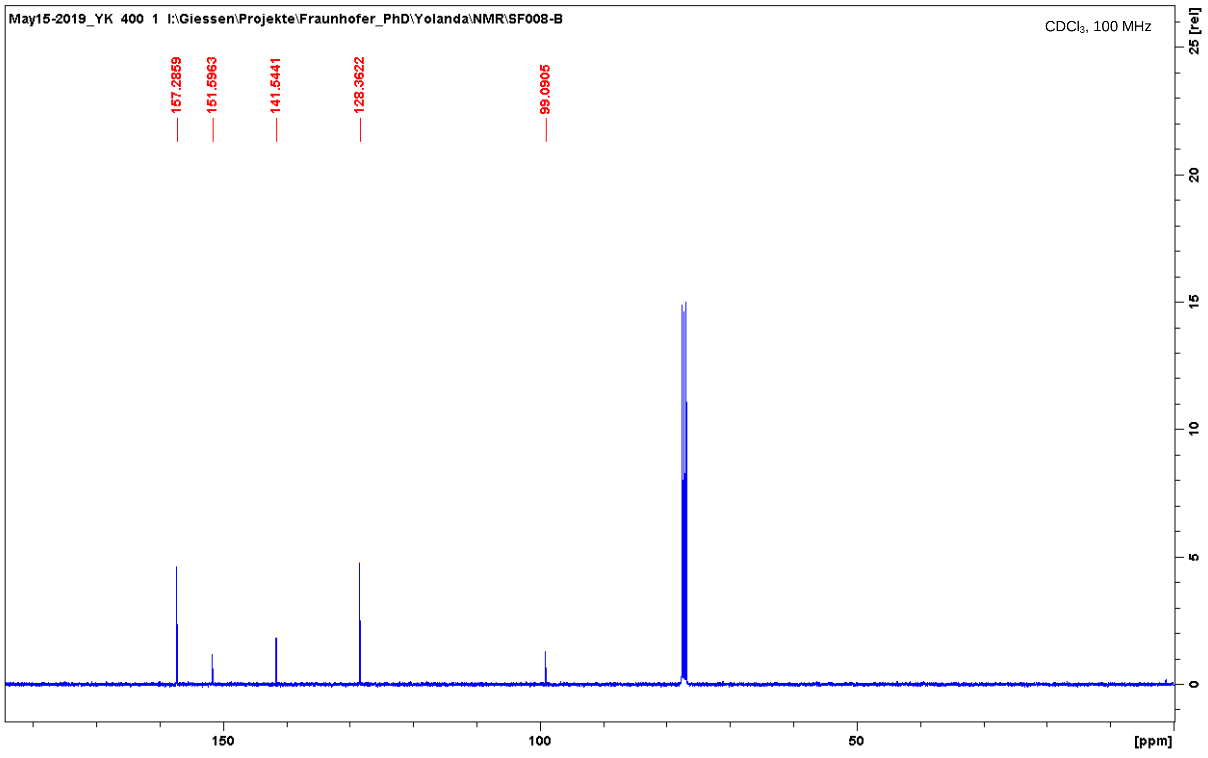
May15-2019_YK 400 1 I:\Giessen\Projekte\Fraunhofer_PhD\Yolanda\NMR\SF008-B
CDCl3, 100 MHz
157.2859
151.5963
141.5441
128.3622
99.0905
[rel]
[ppm]

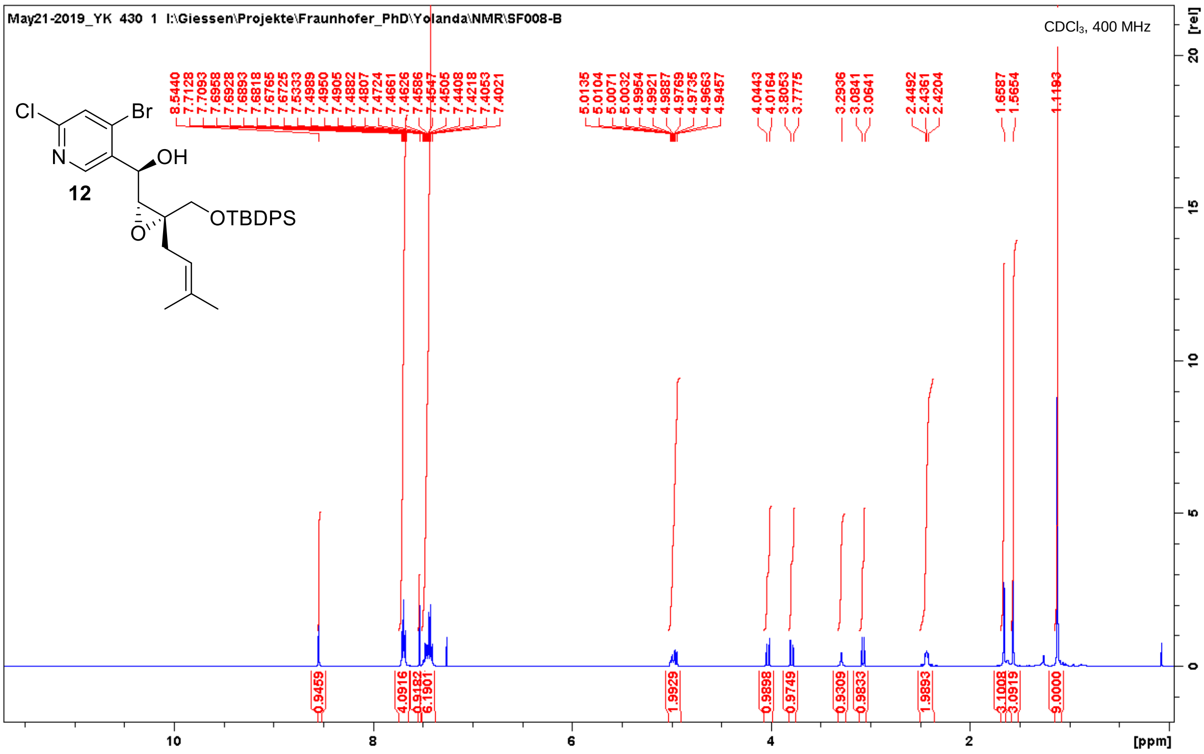
May21-2019_YK 430 1 I:\Giessen\Projekte\Fraunhofer_PhD\Yolanda\NMR\SF008-B
CDCl3, 400 MHz
Cl
Br
N
OH
12
O
OTBDPS
[rel]
[ppm]

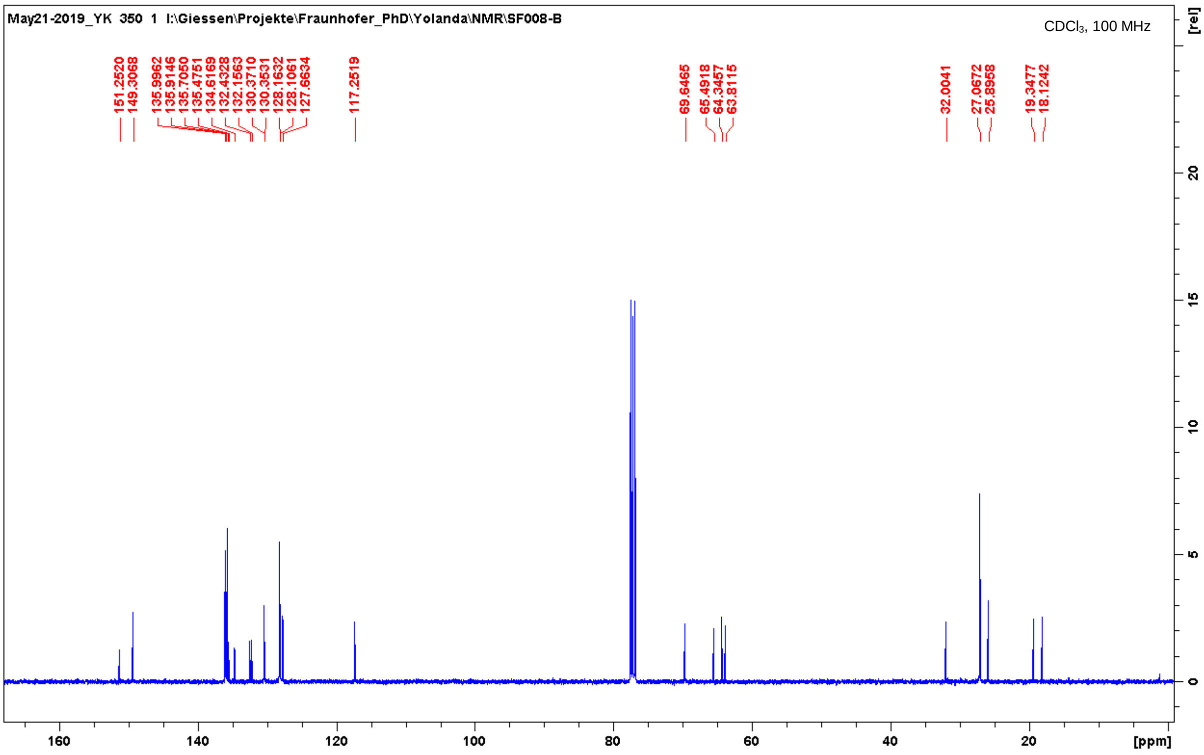
May21-2019_YK 350 1 I:\Giessen\Projekte\Fraunhofer_PhD\Yolanda\NMR\SF008-B
CDCl3, 100 MHz
[rel]
[ppm]

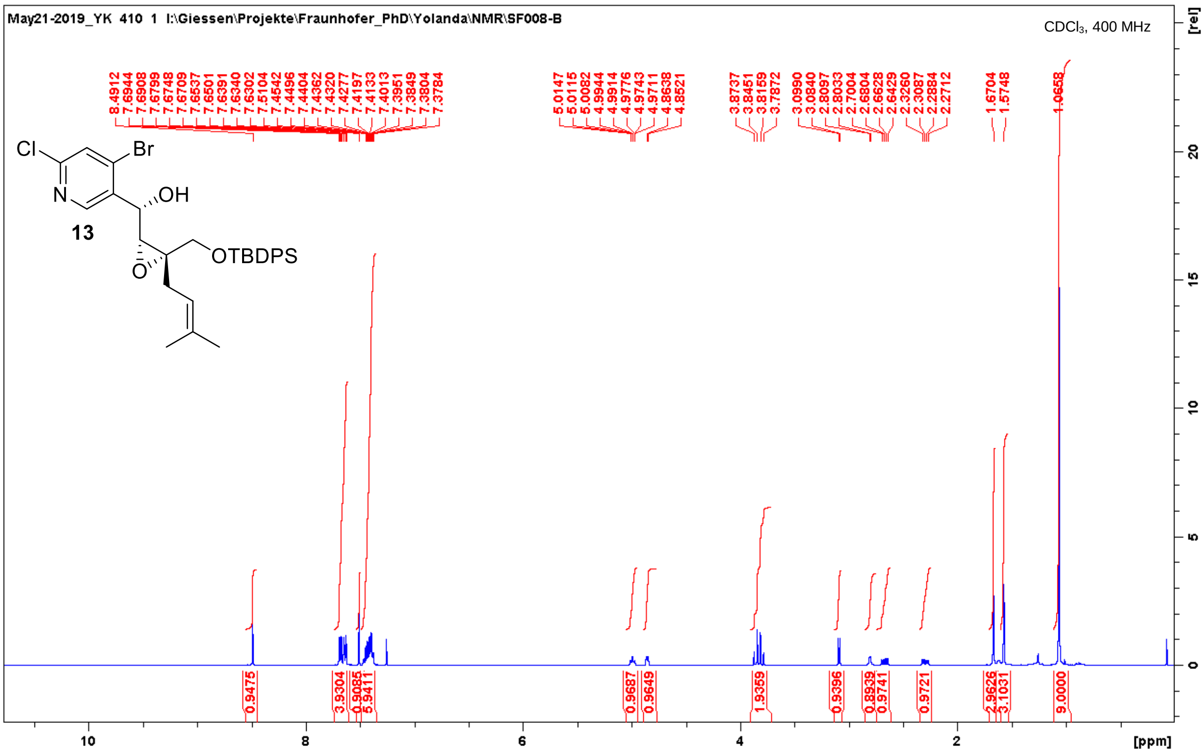
May21-2019_YK 410 1 I:\Giessen\Projekte\Fraunhofer_PhD\Yolanda\NMR\SF008-B
CDCl3, 400 MHz
Cl
Br
N
OH
13
O
OTBDPS
8.4912 7.6944 7.6908 7.6799 7.6748 7.6709 7.6537 7.6501 7.6391 7.6340 7.6302 7.5104 7.4542 7.4496 7.4404 7.4362 7.4320 7.4277 7.4197 7.4133 7.4013 7.3951 7.3849 7.3804 7.3784
5.0147 5.0115 5.0082 4.9944 4.9914 4.9776 4.9743 4.9711 4.8638 4.8521
3.8737 3.8451 3.8159 3.7872
3.0990 3.0840 2.8097 2.8033 2.7004 2.6804 2.6628 2.6429 2.3260 2.3087 2.2884 2.2712
1.6704 1.5748
1.0658
0.9475 3.9304 0.9085 5.9411 0.9687 0.9649 1.9359 0.9396 0.8939 0.9741 0.9721 2.9626 3.1031 9.0000
[ppm]
[rel]

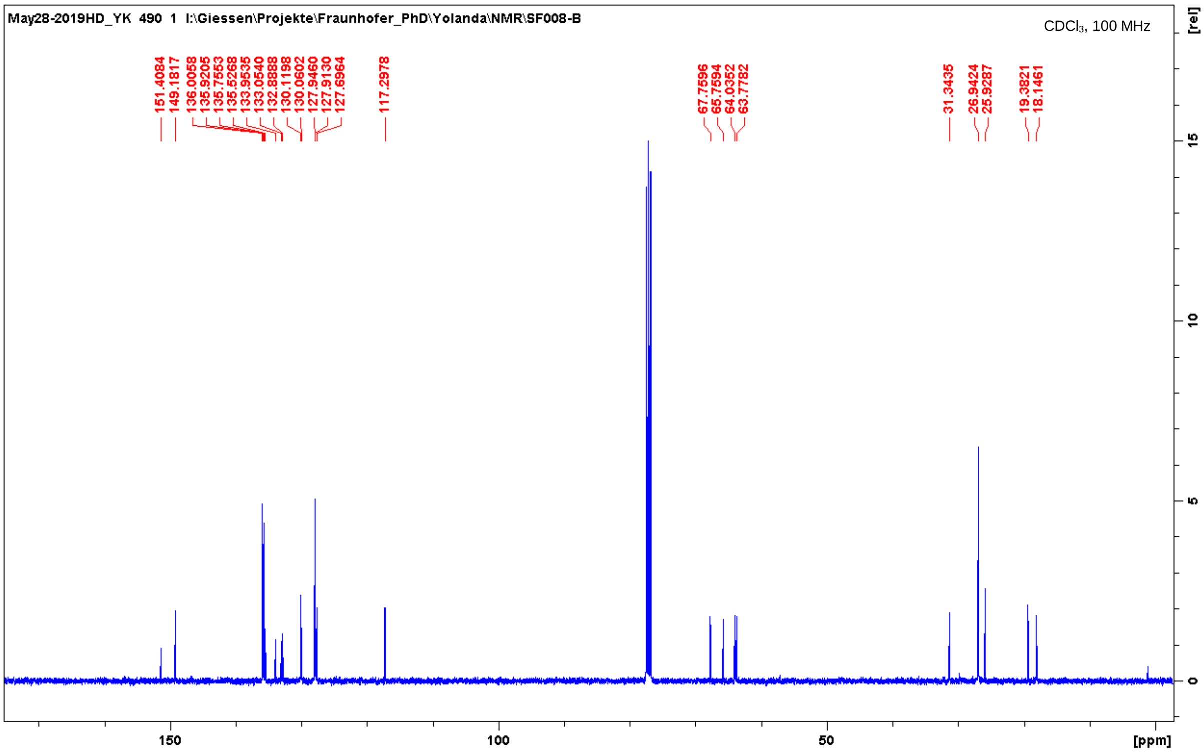
May28-2019HD_YK 490 1 I:\Giessen\Projekte\Fraunhofer_PhD\Yolanda\NMR\SF008-B
CDCl3, 100 MHz
151.4084 149.1817 136.0058 135.9205 135.7553 135.5268 133.9535 133.0540 132.8888 130.1198 130.0602 127.9460 127.9130 127.6964 117.2978
67.7596 65.7594 64.0352 63.7782
31.3435 26.9424 25.9287 19.3821 18.1461
[ppm]
[rel]

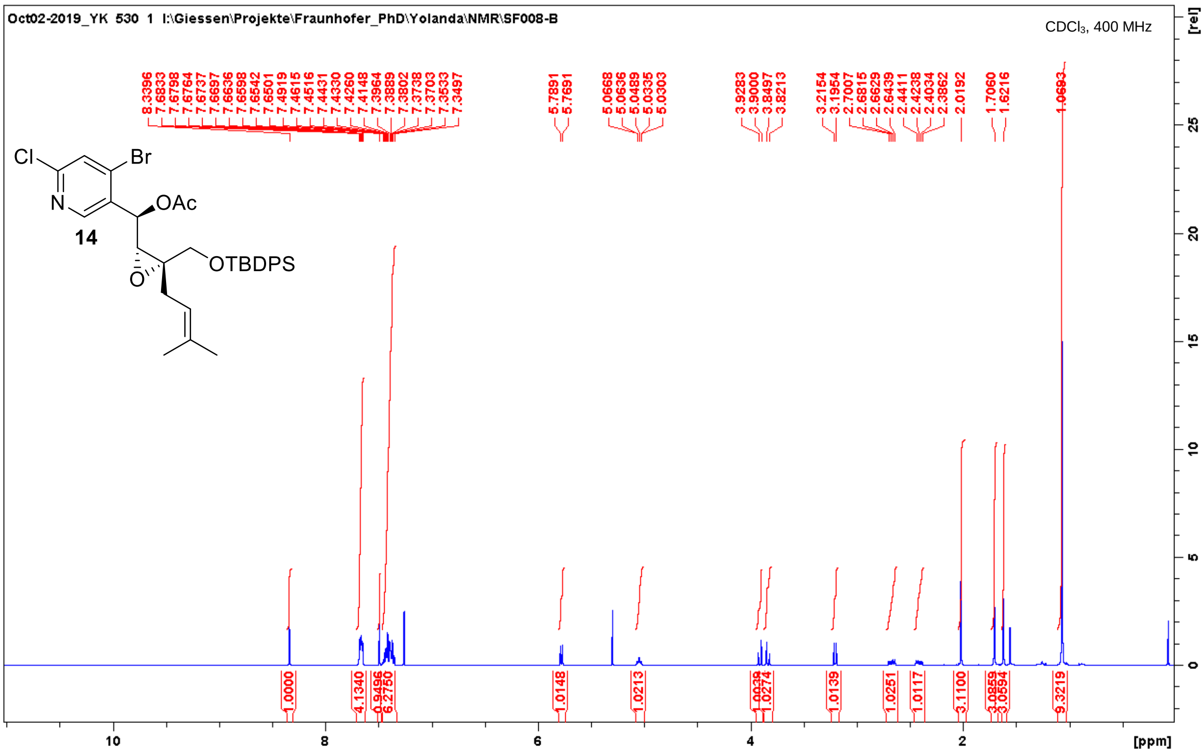
Oct02-2019_YK 530 1 I:\Giessen\Projekte\Fraunhofer_PhD\Yolanda\NMR\SF008-B
CDCl3, 400 MHz
14
OAc
OTBDPS
[ppm]
[rel]

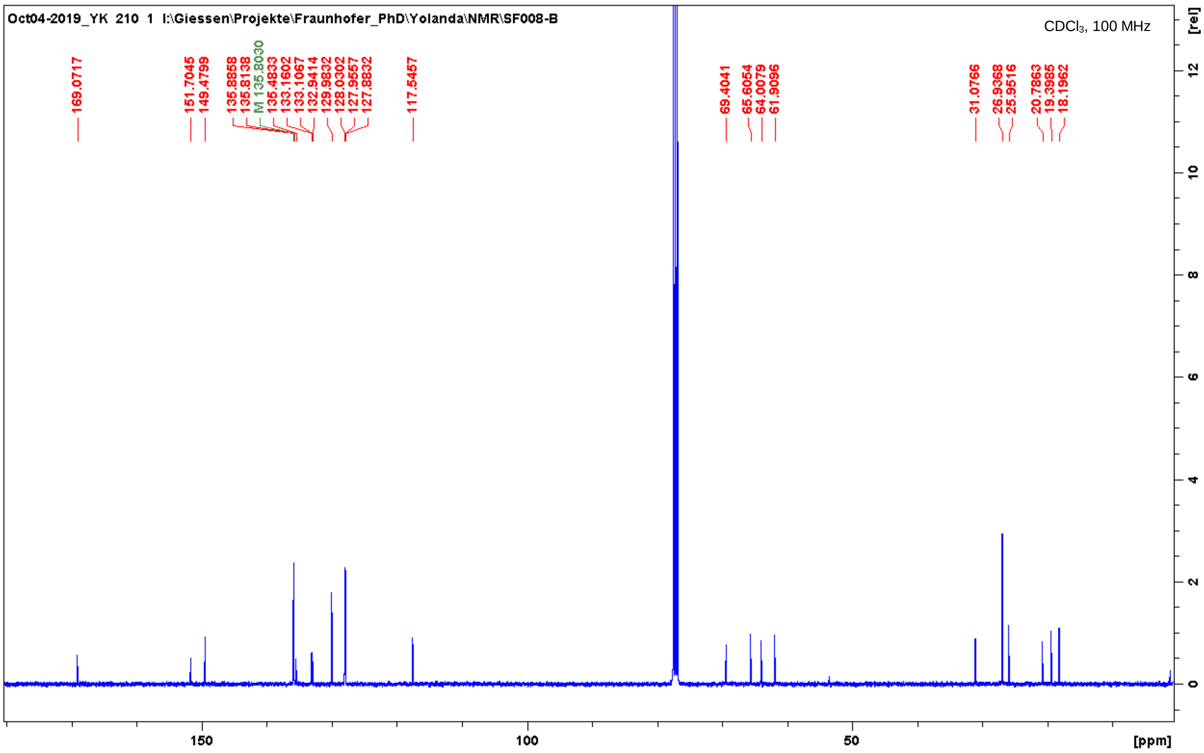
Oct04-2019_YK 210 1 I:\Giessen\Projekte\Fraunhofer_PhD\Yolanda\NMR\SF008-B
CDCl3, 100 MHz
[ppm]
[rel]

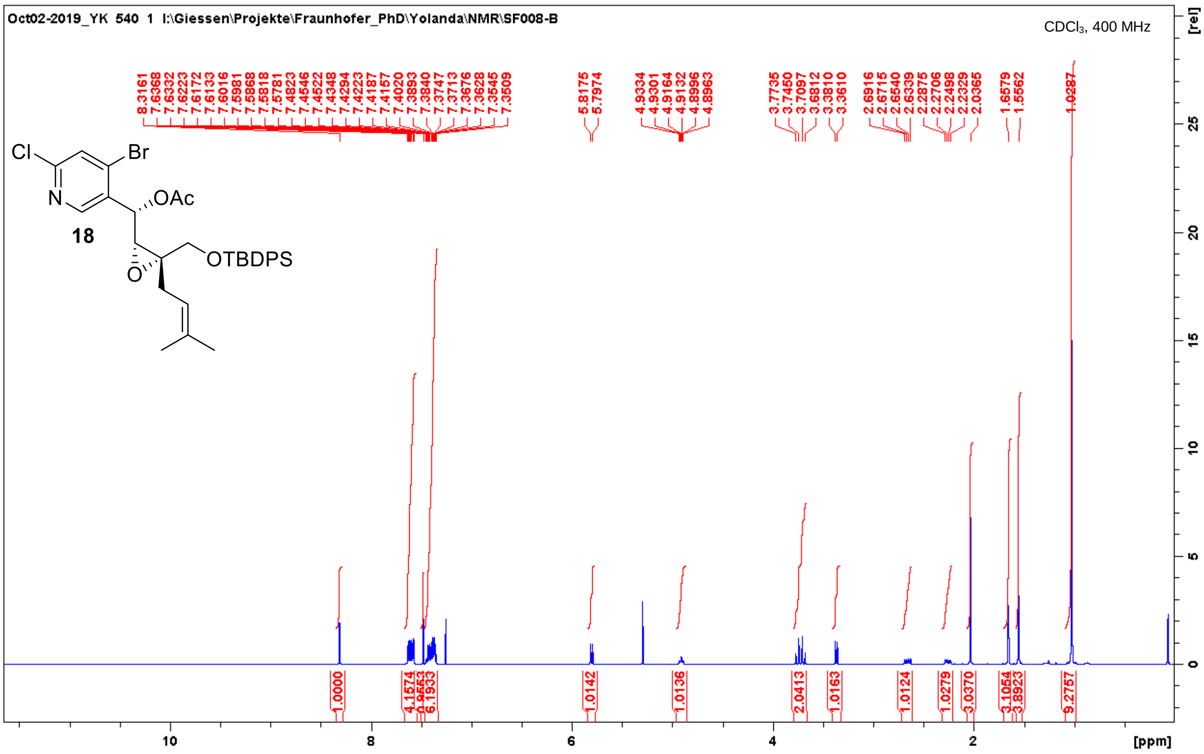
Oct02-2019_YK 540 1 I:\Giessen\Projekte\Fraunhofer_PhD\Yolanda\NMR\SF008-B
CDCl3, 400 MHz
18
OAc
OTBDPS
[ppm]
[rel]

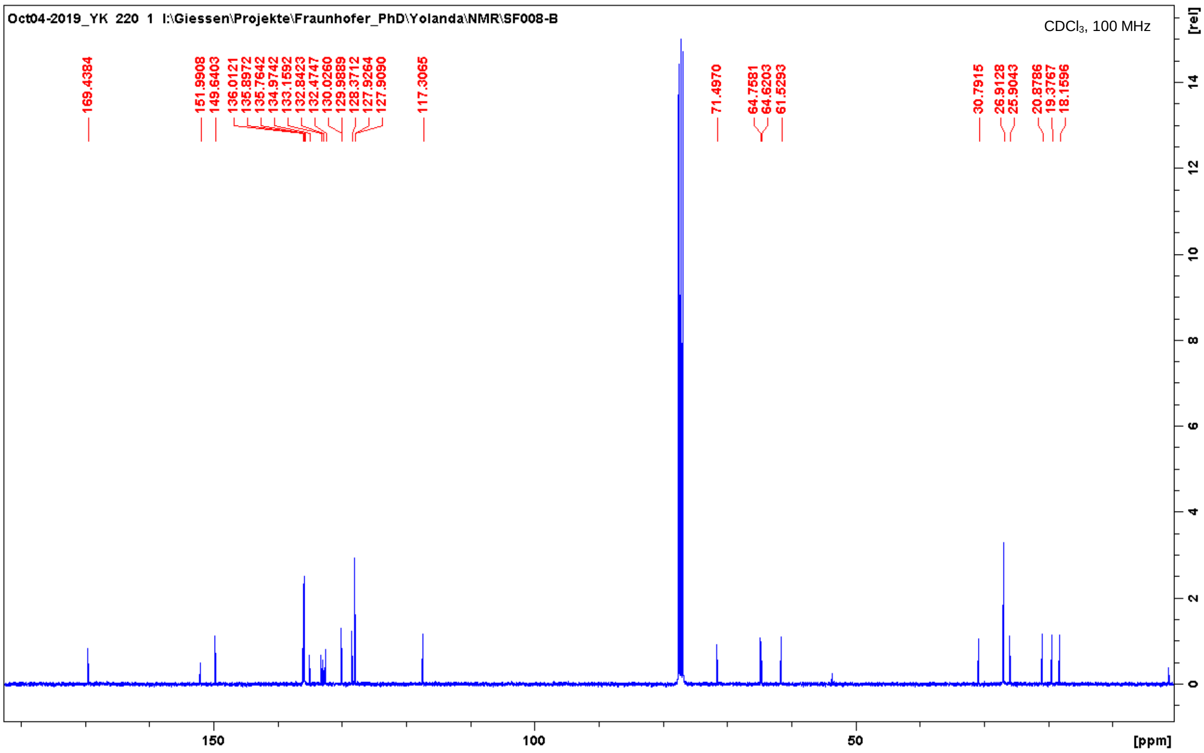
Oct04-2019_YK 220 1 I:\Giessen\Projekte\Fraunhofer_PhD\Yolanda\NMR\SF008-B
CDCl3, 100 MHz
[ppm]
[rel]

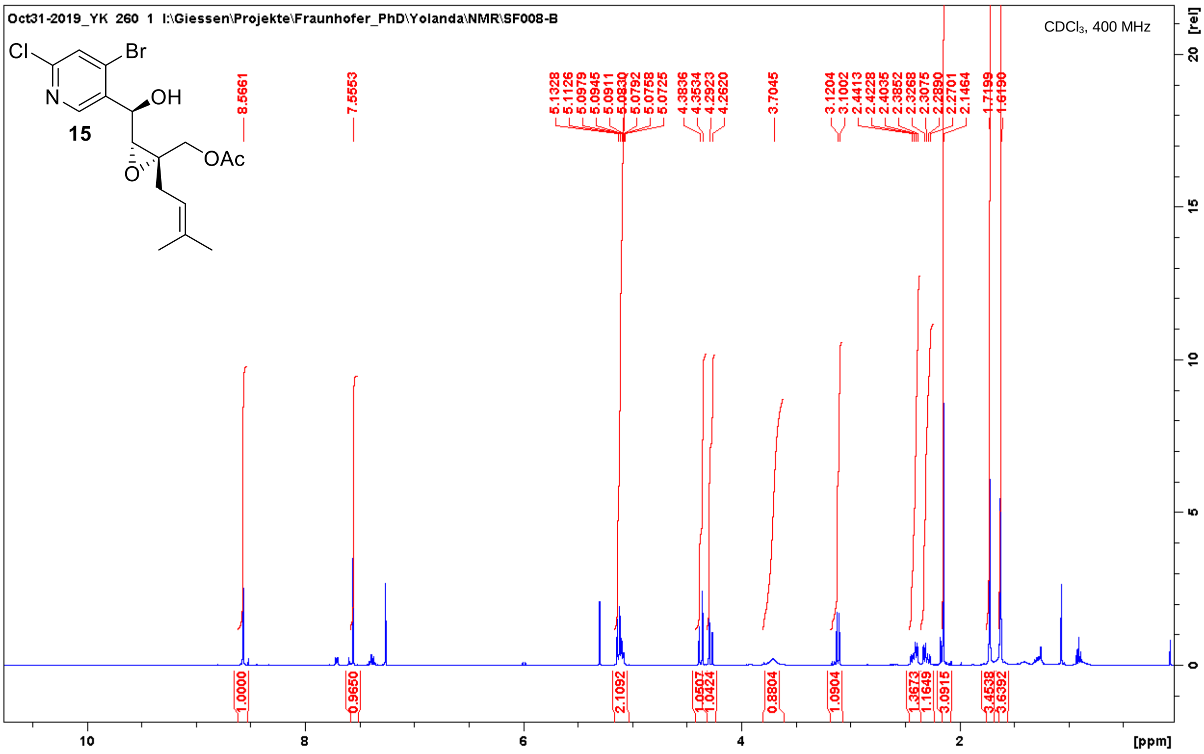
Oct31-2019_YK 260 1 I:\Giessen\Projekte\Fraunhofer_PhD\Yolanda\NMR\SF008-B
CDCl3, 400 MHz
15
OH
OAc
8.5661
7.5553
5.1328 5.1126 5.0979 5.0945 5.0911 5.0830 5.0792 5.0758 5.0725
4.3836 4.3534 4.2923 4.2620
3.7045
3.1204 3.1002
2.4413 2.4228 2.4035 2.3852 2.3268 2.3075 2.2890 2.2701 2.1464
1.7199 1.6190
1.0000 0.9650 2.1092 1.0507 1.0424 0.8804 1.0904 1.3673 1.1649 3.0915 3.4538 3.6392
[ppm]
[rel]

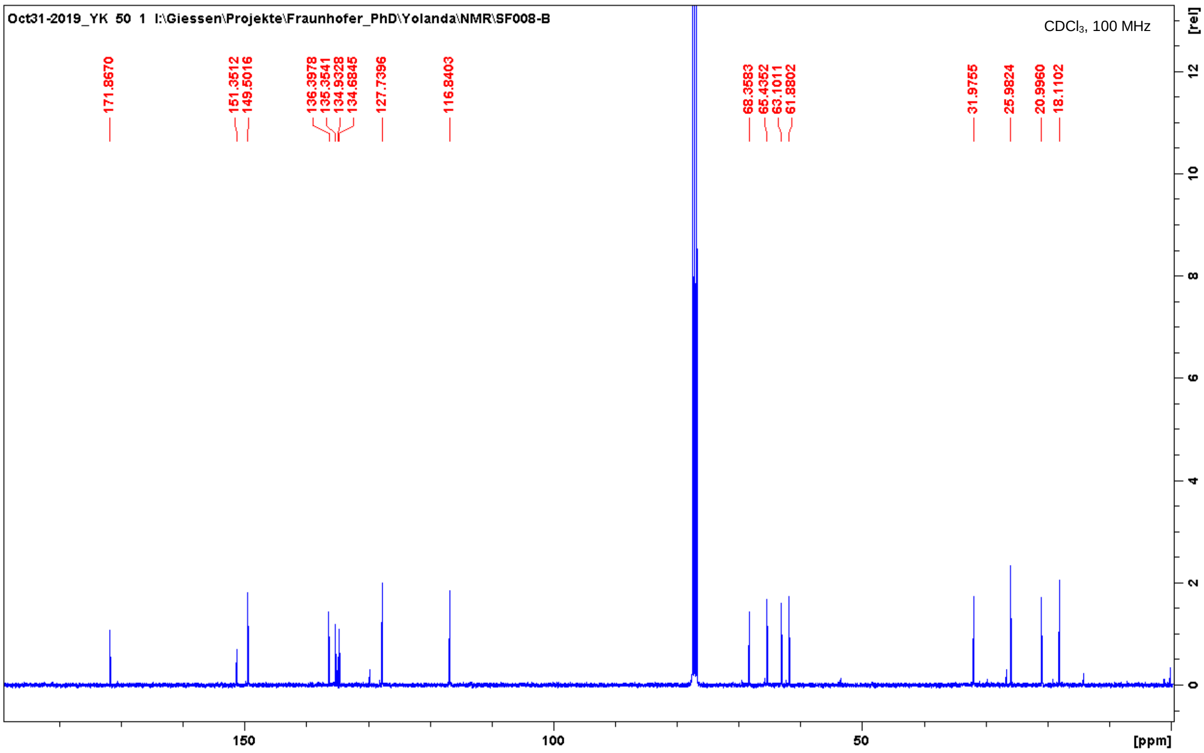
Oct31-2019_YK 50 1 I:\Giessen\Projekte\Fraunhofer_PhD\Yolanda\NMR\SF008-B
CDCl3, 100 MHz
171.8670
151.3512 149.5016
136.3978 135.3541 134.9328 134.6845
127.7396
116.8403
68.3583 65.4352 63.1011 61.8802
31.9755 25.9824 20.9960 18.1102
[ppm]
[rel]

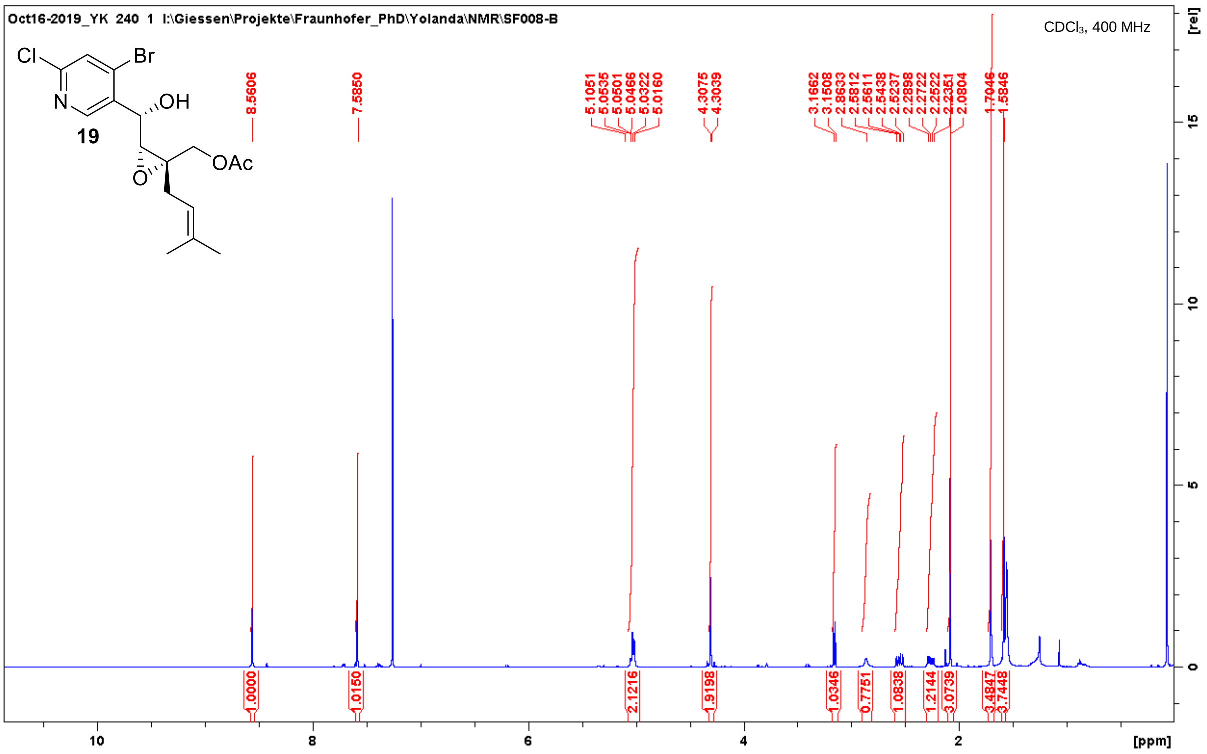
Oct16-2019_YK 240 1 I:\Giessen\Projekte\Fraunhofer_PhD\Yolanda\NMR\SF008-B
CDCl3, 400 MHz
19
Cl
Br
N
OH
O
OAc
8.5606
7.5850
5.1051
5.0535
5.0501
5.0466
5.0322
5.0160
4.3075
4.3039
3.1662
3.1608
2.8633
2.5812
2.5611
2.5438
2.5237
2.2898
2.2722
2.2522
2.2351
2.0804
1.7046
1.5846
1.0000
1.0150
2.1216
1.9198
1.0346
0.7751
1.0838
1.2144
3.0739
3.4847
3.7448
[ppm]
[rel]
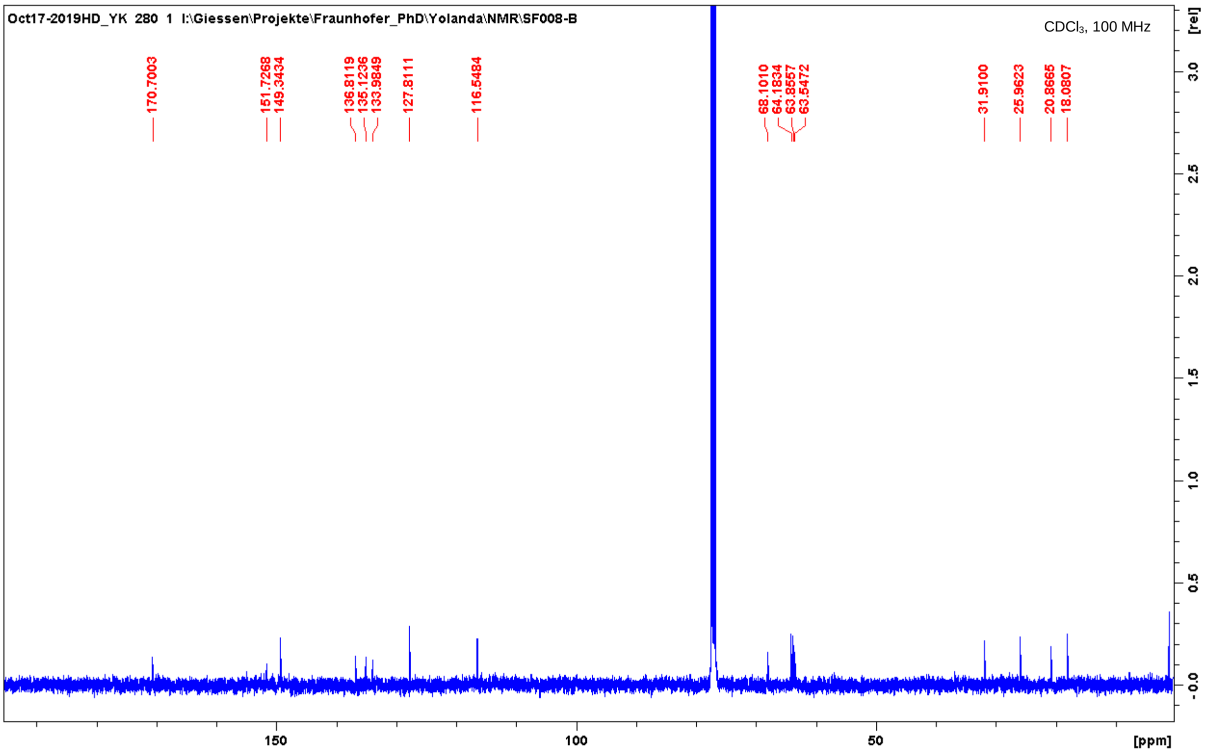
Oct17-2019HD_YK 280 1 I:\Giessen\Projekte\Fraunhofer_PhD\Yolanda\NMR\SF008-B
CDCl3, 100 MHz
170.7003
151.7268
149.3434
136.8119
135.1236
133.9849
127.8111
116.5484
68.1010
64.1834
63.8557
63.5472
31.9100
25.9623
20.8665
18.0807
[ppm]
[rel]

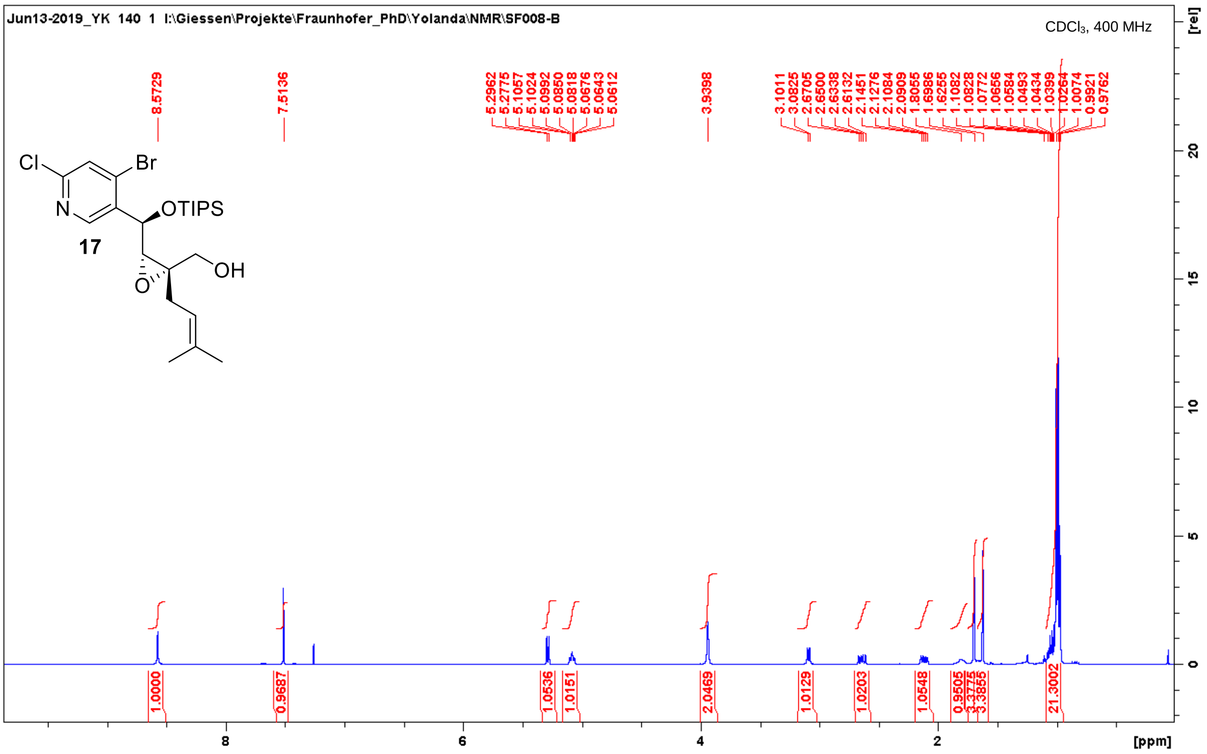

17

Jun13-2019_YK 140 1 I:\Giessen\Projekte\Fraunhofer_PhD\Yolanda\NMR\SF008-B
CDCl3, 400 MHz

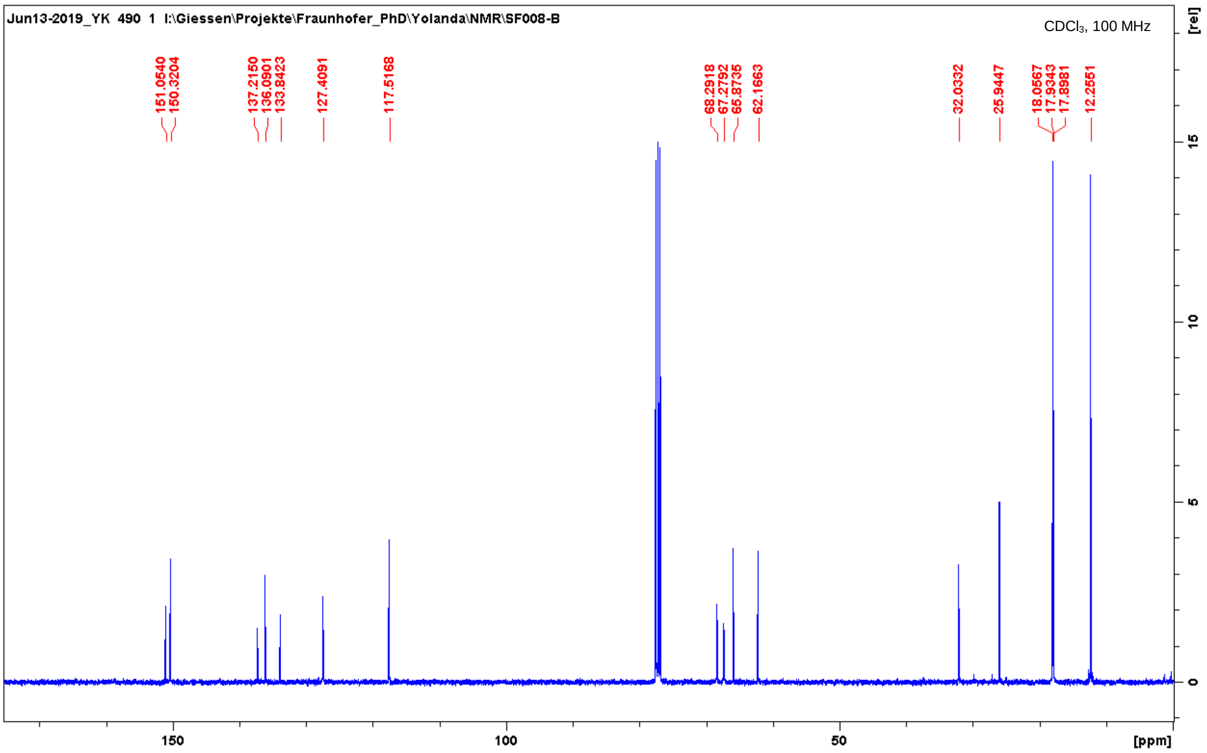

Jun13-2019_YK 490 1 I:\Giessen\Projekte\Fraunhofer_PhD\Yolanda\NMR\SF008-B
CDCl3, 100 MHz

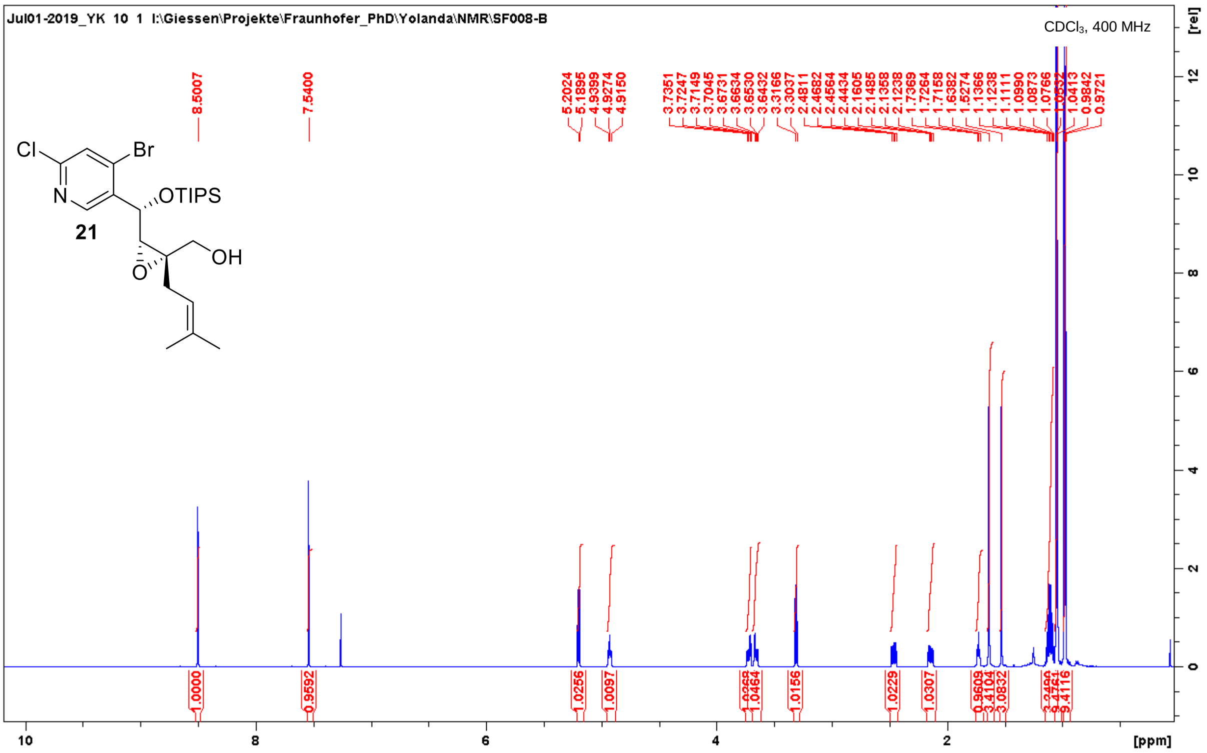
Jul01-2019_YK 10 1 I:\Giessen\Projekte\Fraunhofer_PhD\Yolanda\NMR\SF008-B
CDCl3, 400 MHz
21
OTIPS
OH
[ppm]
[rel]

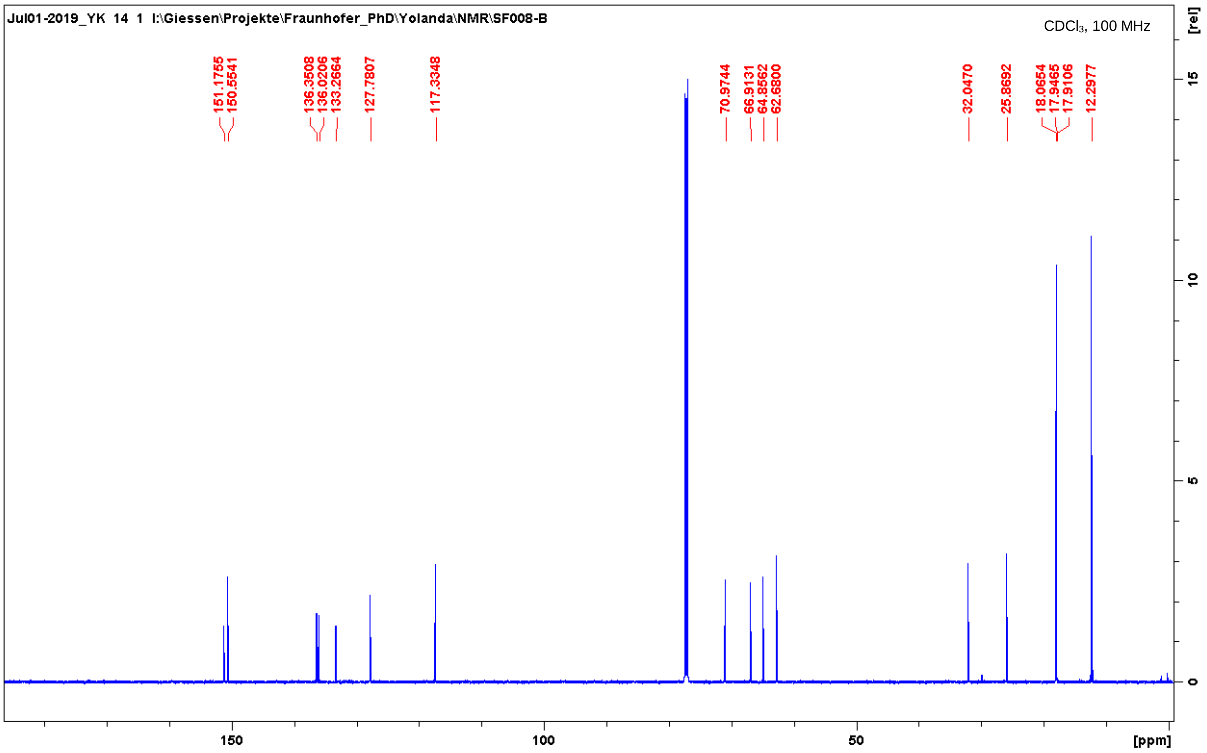
Jul01-2019_YK 14 1 I:\Giessen\Projekte\Fraunhofer_PhD\Yolanda\NMR\SF008-B
CDCl3, 100 MHz
[ppm]
[rel]

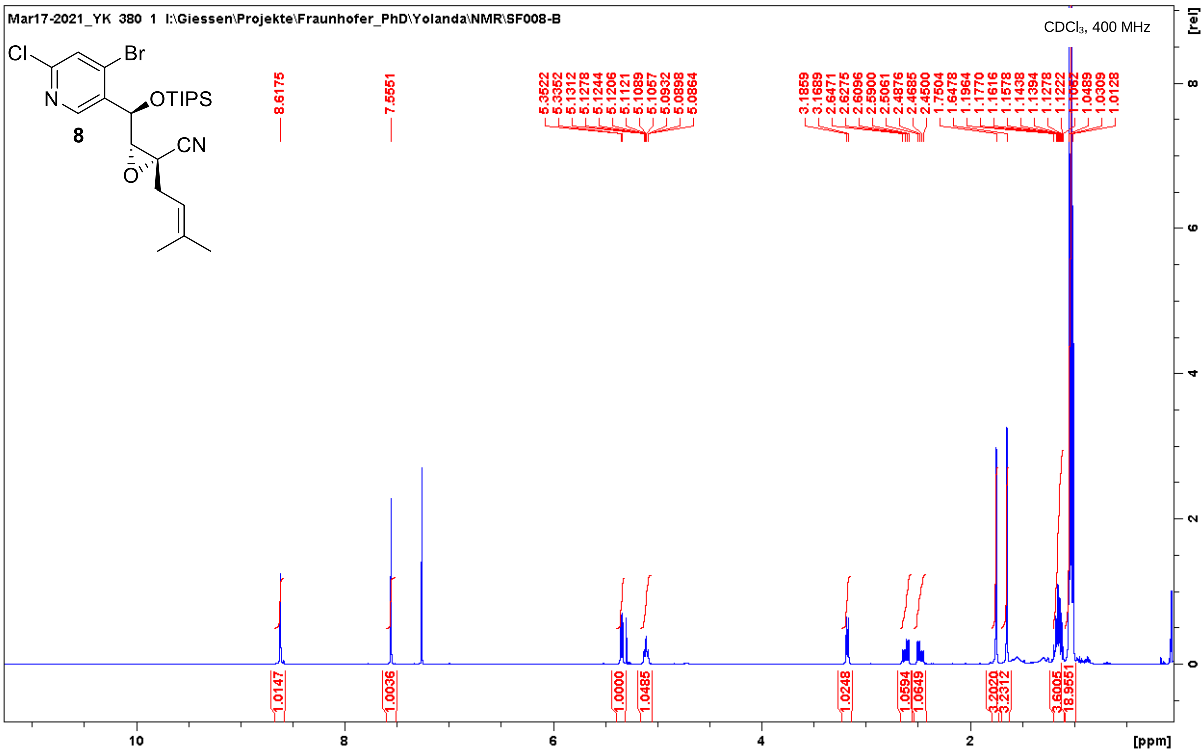

Mar17-2021_YK 380 1 I:\Giessen\Projekte\Fraunhofer_PhD\Yolanda\NMR\SF008-B
CDCl3, 400 MHz
Cl
Br
N
OTIPS
8
CN
O
8.6175
7.5551
5.3522 5.3352 5.1312 5.1278 5.1244 5.1206 5.1121 5.1089 5.1057 5.0932 5.0898 5.0864
3.1859 3.1689 2.6471 2.6275 2.6096 2.5900 2.5061 2.4876 2.4685 2.4500 1.7504 1.6478 1.1964 1.1770 1.1616 1.1578 1.1438 1.1394 1.1278 1.1222 1.1062 1.0489 1.0309 1.0128
1.0147
1.0036
1.0000
1.0485
1.0248
1.0594
1.0649
3.2020
3.2312
3.6005
18.9551
[ppm]
[rel]

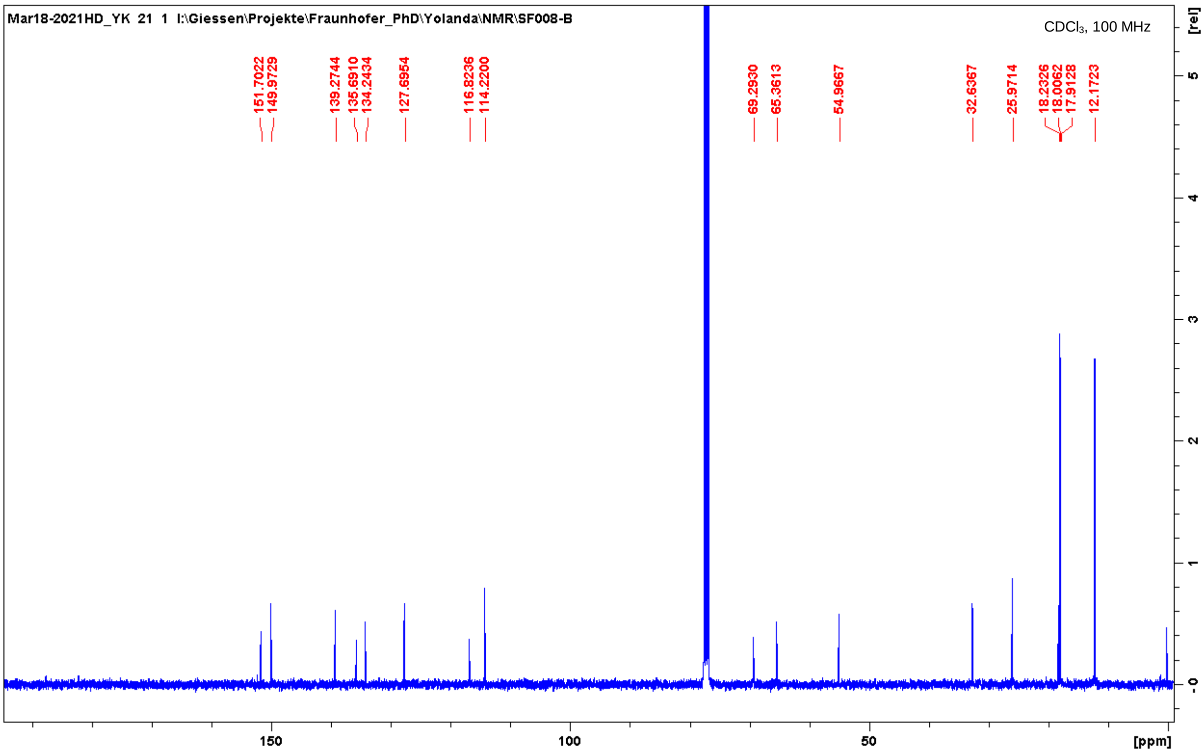

Mar18-2021HD_YK 21 1 I:\Giessen\Projekte\Fraunhofer_PhD\Yolanda\NMR\SF008-B
CDCl3, 100 MHz
151.7022
149.9729
139.2744
135.6910
134.2434
127.6954
116.8236
114.2200
69.2930
65.3613
54.9667
32.6367
25.9714
18.2326
18.0062
17.9128
12.1723
[ppm]
[rel]

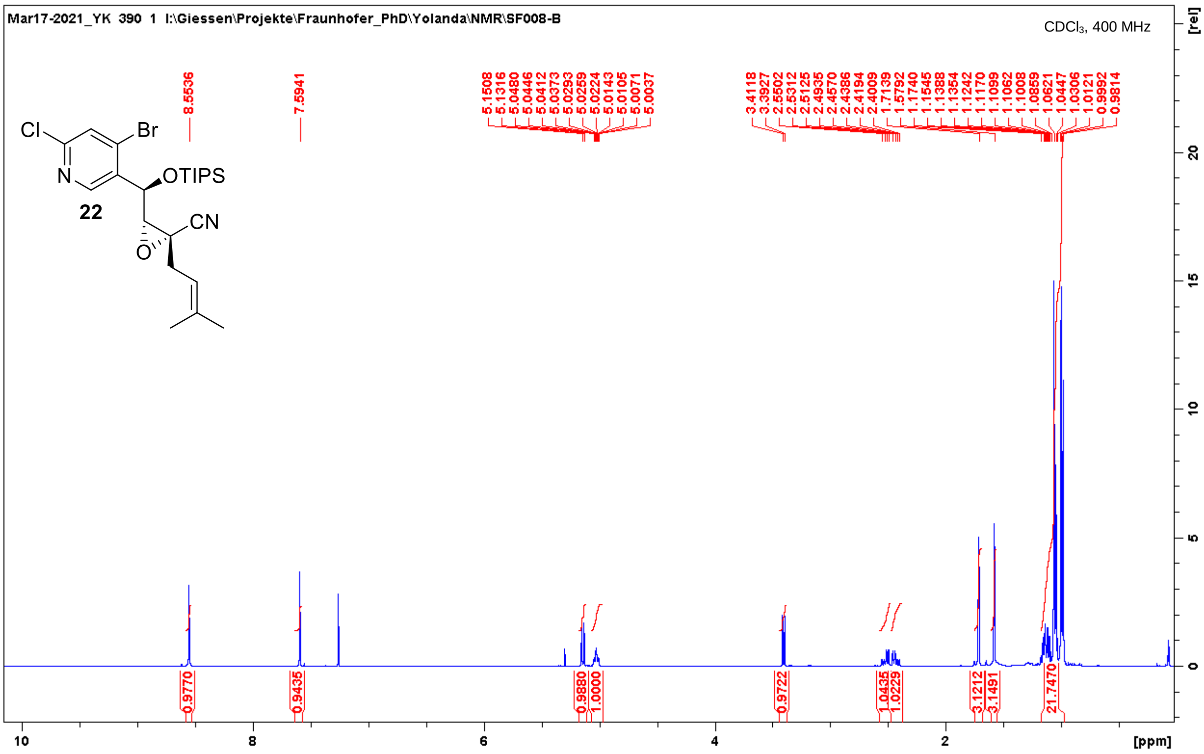

Mar17-2021_YK 390 1 I:\Giessen\Projekte\Fraunhofer_PhD\Yolanda\NMR\SF008-B
CDCl3, 400 MHz
22
OTIPS
CN

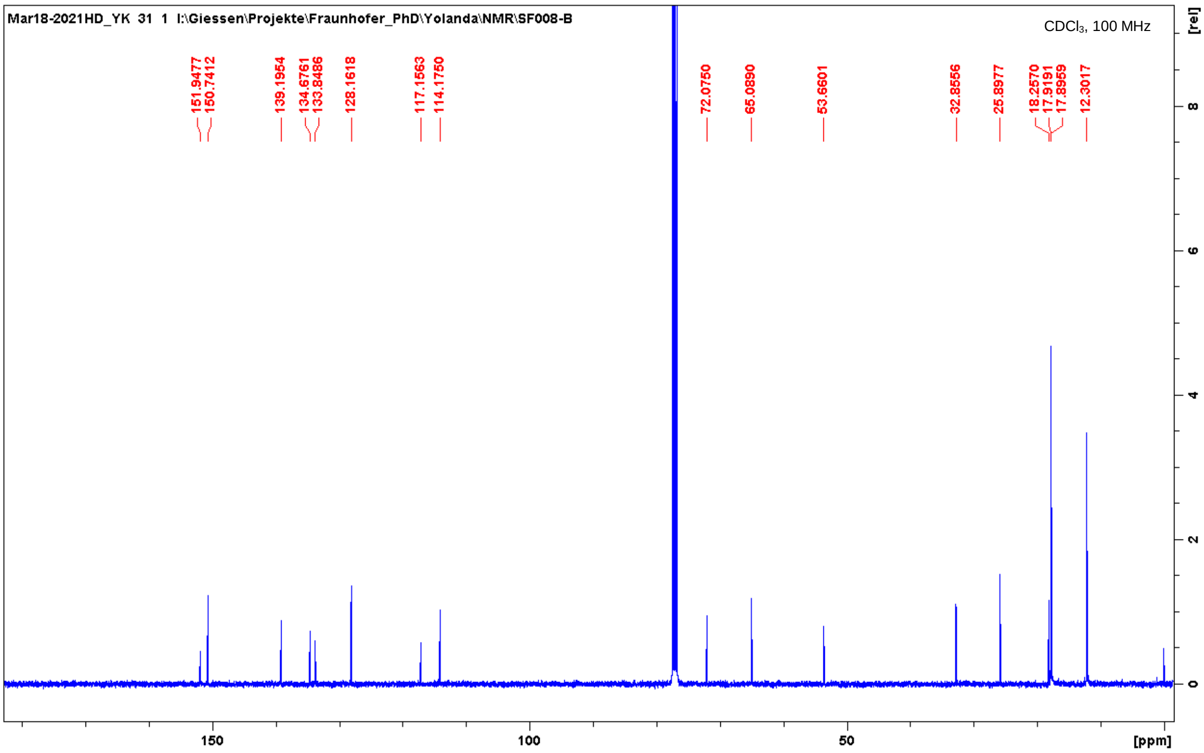

Mar18-2021HD_YK 31 1 I:\Giessen\Projekte\Fraunhofer_PhD\Yolanda\NMR\SF008-B
CDCl3, 100 MHz
151.9477
150.7412
139.1954
134.6761
133.8486
128.1618
117.1563
114.1750
72.0750
65.0890
53.6601
32.8556
25.8977
18.2570
17.9191
17.8959
12.3017

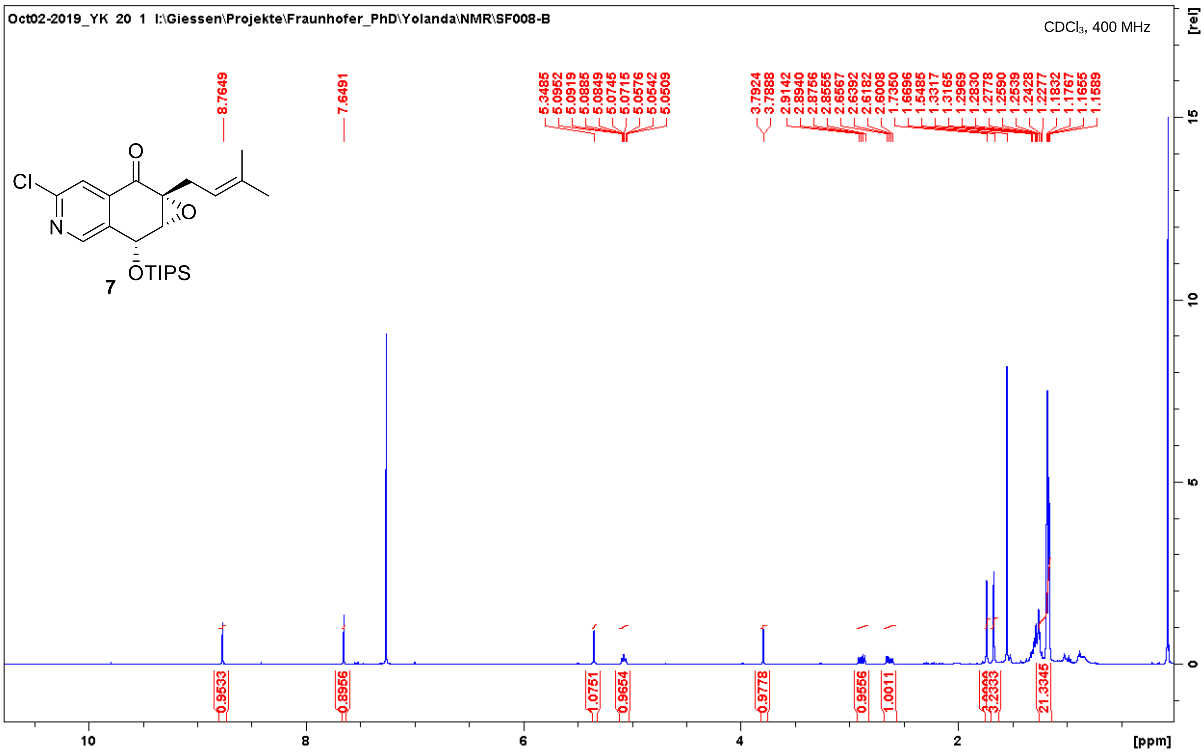
Oct02-2019_YK 20 1 I:\Giessen\Projekte\Fraunhofer_PhD\Yolanda\NMR\SF008-B
CDCl3, 400 MHz
8.7649
7.6491
5.3485 5.0952 5.0919 5.0885 5.0849 5.0745 5.0715 5.0576 5.0542 5.0509
3.7924 3.7888 2.9142 2.8940 2.8756 2.8555 2.6567 2.6392 2.6182 2.6008 1.7350 1.6696 1.5485 1.3317 1.3165 1.2969 1.2830 1.2778 1.2590 1.2539 1.2428 1.2277 1.1832 1.1767 1.1655 1.1589
0.9533 0.8956 1.0751 0.9654 0.9778 0.9556 1.0011 3.2333 21.3345
Cl
N
O
OTIPS
7
[ppm]
[rel]

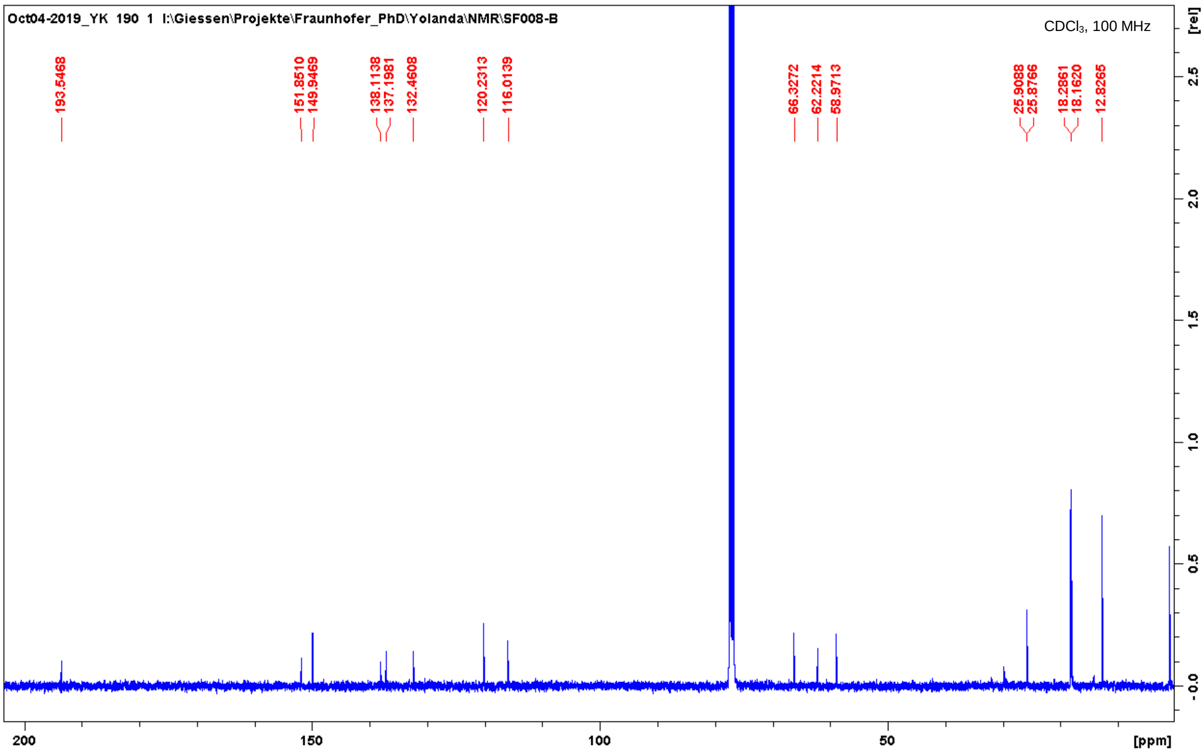
Oct04-2019_YK 190 1 I:\Giessen\Projekte\Fraunhofer_PhD\Yolanda\NMR\SF008-B
CDCl3, 100 MHz
193.5468
151.8510 149.9469
138.1138 137.1981 132.4608
120.2313 116.0139
66.3272 62.2214 58.9713
25.9088 25.8766 18.2861 18.1620 12.8265
[ppm]
[rel]

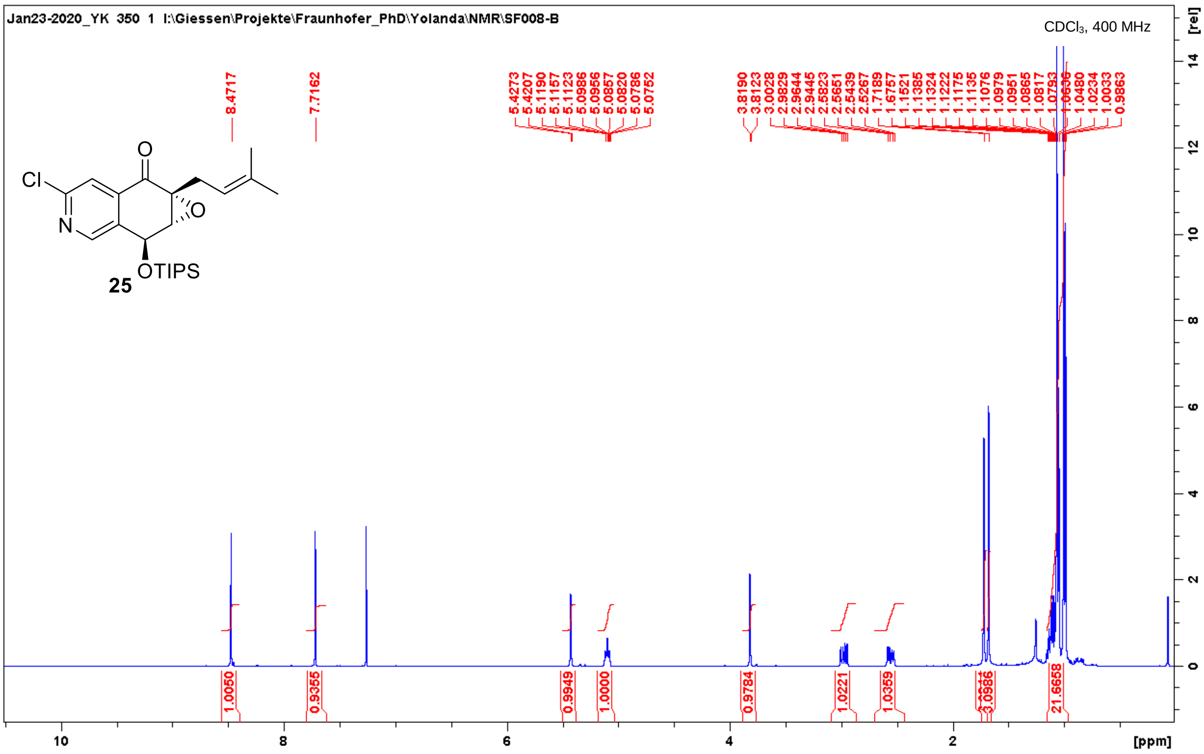
Jan23-2020_YK 350 1 I:\Giessen\Projekte\Fraunhofer_PhD\Yolanda\NMR\SF008-B
CDCl3, 400 MHz
25
OTIPS
Cl
N
O
[ppm]
[rel]

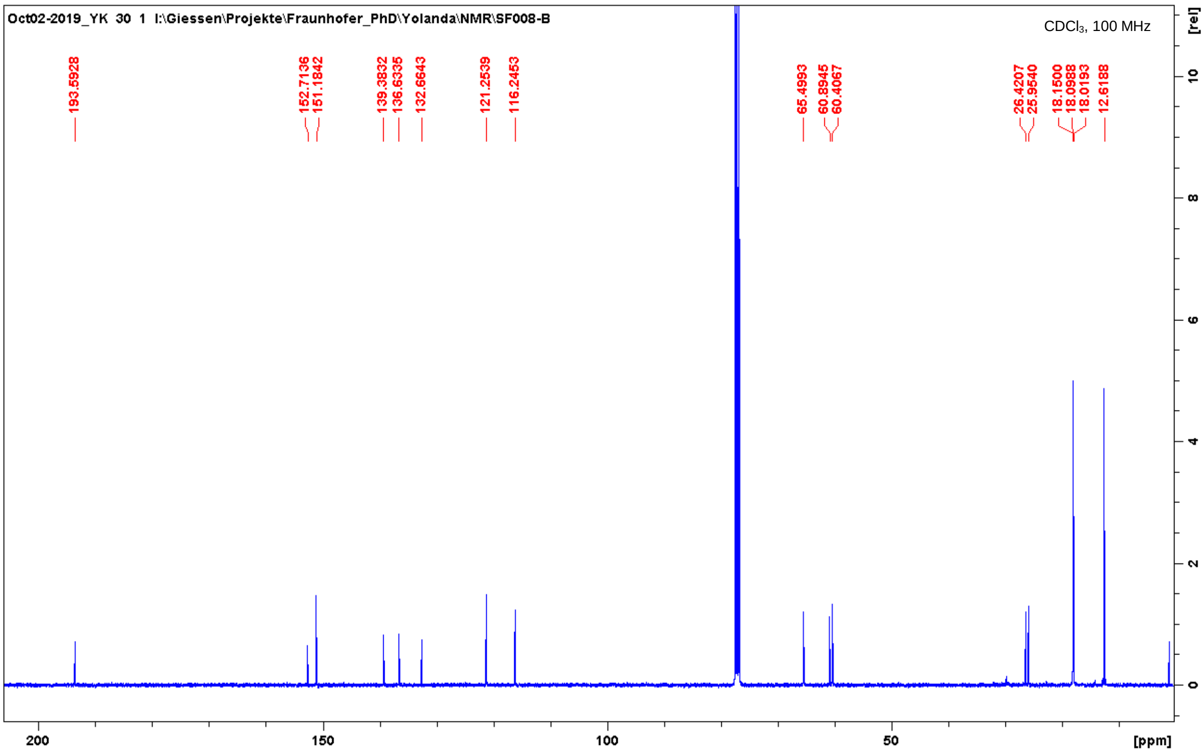
Oct02-2019_YK 30 1 I:\Giessen\Projekte\Fraunhofer_PhD\Yolanda\NMR\SF008-B
CDCl3, 100 MHz
193.5928
152.7136
151.1842
139.3832
136.6335
132.6643
121.2539
116.2453
65.4993
60.8945
60.4067
26.4207
25.9540
18.1500
18.0988
18.0193
12.6188
[ppm]
[rel]

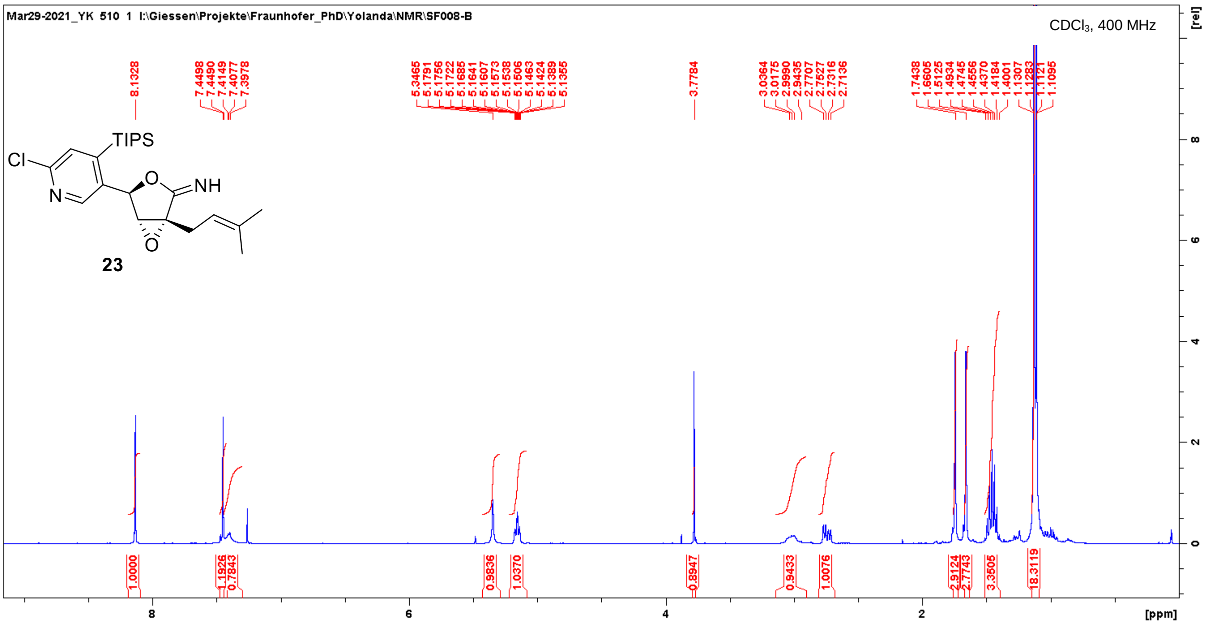

Mar29-2021_YK 510 1 I:\Giessen\Projekte\Fraunhofer_PhD\Yolanda\NMR\SF008-B
CDCl3, 400 MHz
8.1328
7.4498 7.4490 7.4149 7.4077 7.3978
5.3465 5.1791 5.1756 5.1722 5.1685 5.1641 5.1607 5.1573 5.1538 5.1506 5.1463 5.1424 5.1389 5.1355
3.7784
3.0364 3.0175 2.9950 2.9436 2.7707 2.7527 2.7316 2.7136
1.7438 1.6605 1.5125 1.4934 1.4745 1.4556 1.4370 1.4184 1.4001 1.1307 1.1283 1.1121 1.1095
TIPS
Cl
N
O
NH
O
23
1.0000 1.1925 0.7843 0.9836 1.0370 0.8947 0.9433 1.0076 2.9124 2.7743 3.3505 18.3119
8 6 4 2 [ppm]
[rel]

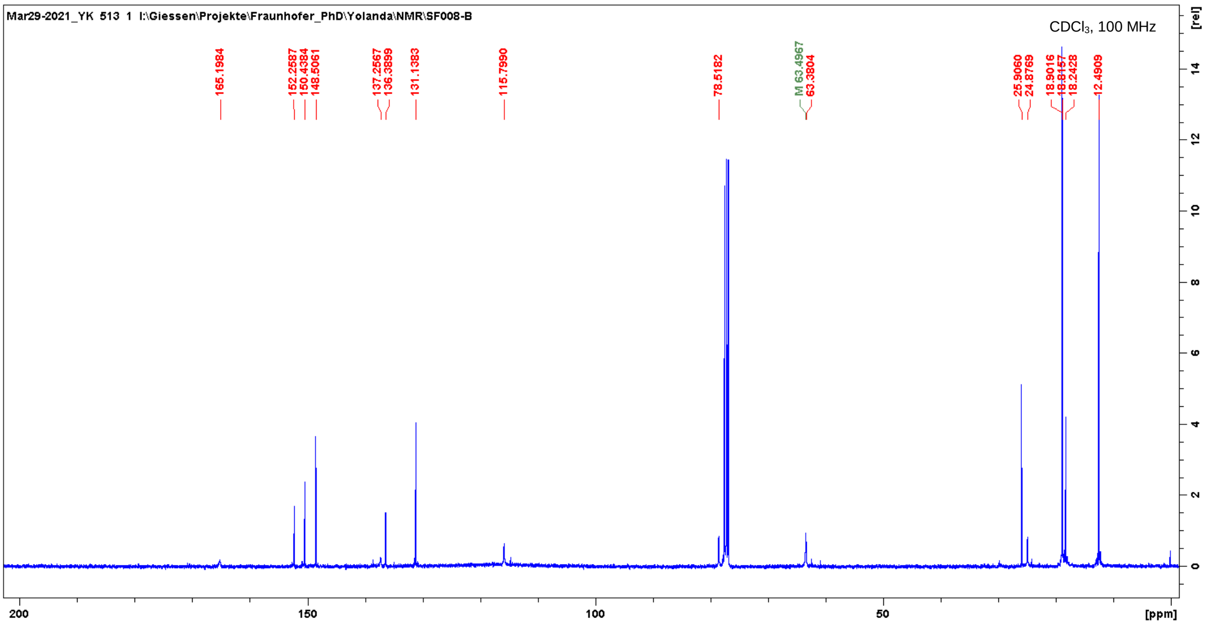

Mar29-2021_YK 513 1 I:\Giessen\Projekte\Fraunhofer_PhD\Yolanda\NMR\SF008-B
CDCl3, 100 MHz
165.1984
152.2587 150.4384 148.5061
137.2567 136.3899
131.1383
115.7990
78.5182
M 63.4967
63.3804
25.9060 24.8769
18.9016 18.8157 18.2428
12.4909
200 150 100 50 [ppm]
[rel]

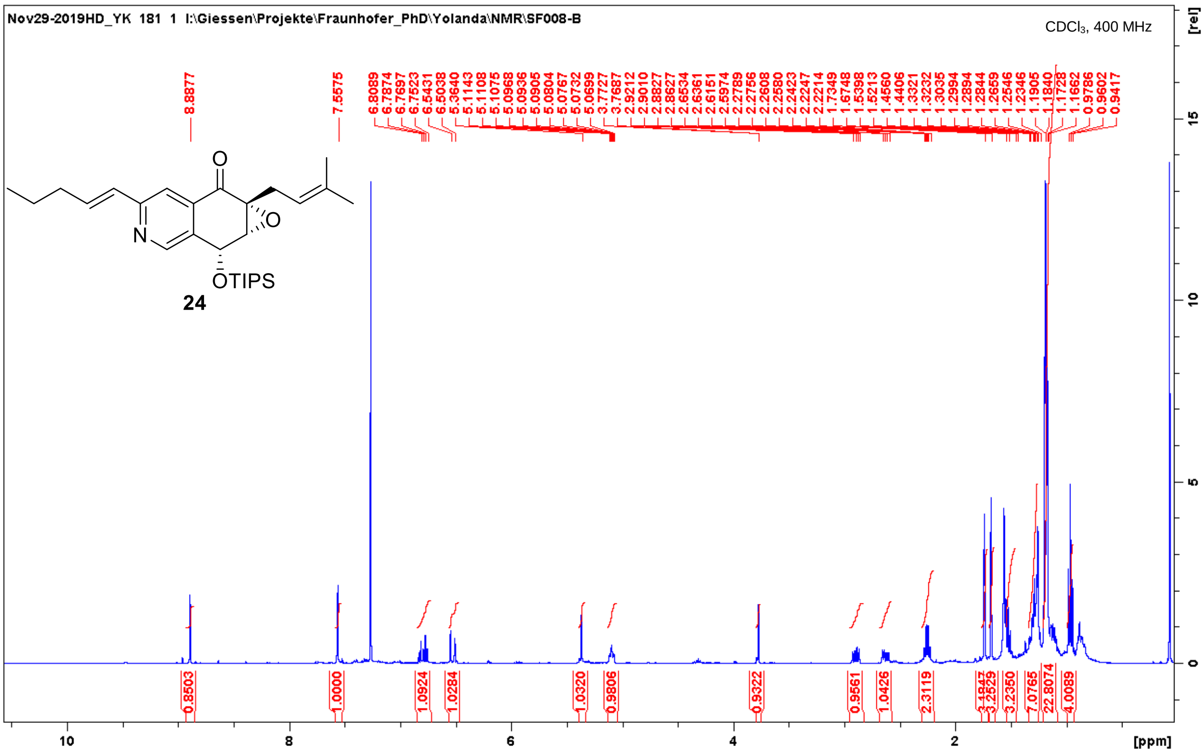
Nov29-2019HD_YK 181 1 I:\Giessen\Projekte\Fraunhofer_PhD\Yolanda\NMR\SF008-B
CDCl3, 400 MHz
8.8877
7.5575
6.8089 6.7874 6.7697 6.7523 6.5431 6.5038 5.3640 5.1143 5.1108 5.1075 5.0968 5.0936 5.0905 5.0804 5.0767 5.0732 5.0699 3.7727 3.7687 2.9212 2.9010 2.8827 2.8627 2.6534 2.6361 2.6151 2.5974 2.2789 2.2756 2.2608 2.2580 2.2423 2.2247 2.2214 1.7349 1.6748 1.5398 1.5213 1.4560 1.4406 1.3321 1.3232 1.3035 1.2994 1.2894 1.2844 1.2659 1.2546 1.2346 1.1905 1.1840 1.1728 1.1662 0.9786 0.9602 0.9417
O
N
O
OTIPS
24
0.8503 1.0000 1.0924 1.0284 1.0320 0.9806 0.9322 0.9561 1.0426 2.3119 3.1847 3.2529 3.2350 7.0765 22.8074 4.0089
[rel]
[ppm]

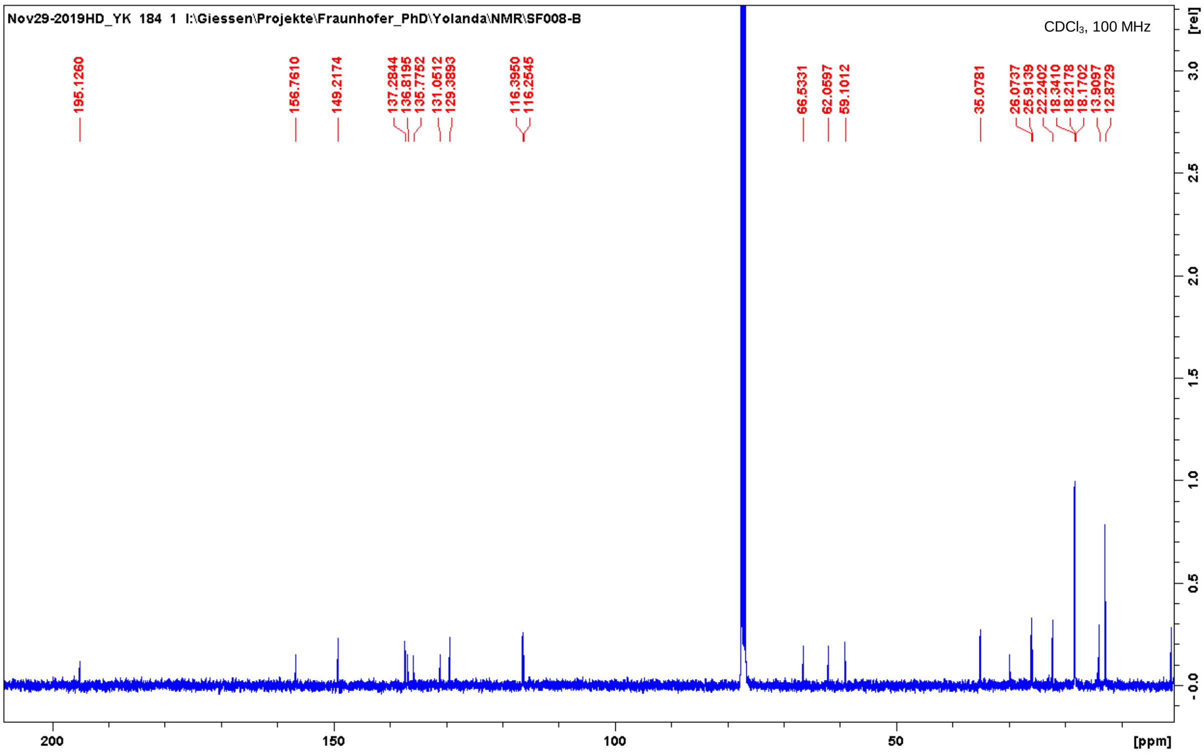
Nov29-2019HD_YK 184 1 I:\Giessen\Projekte\Fraunhofer_PhD\Yolanda\NMR\SF008-B
CDCl3, 100 MHz
195.1260
156.7610
149.2174
137.2844 136.8195 135.7752 131.0512 129.3893
116.3950 116.2545
66.5331 62.0597 59.1012
35.0781
26.0737 25.9139 22.2402 18.3410 18.2178 18.1702 13.9097 12.8729
[rel]
[ppm]

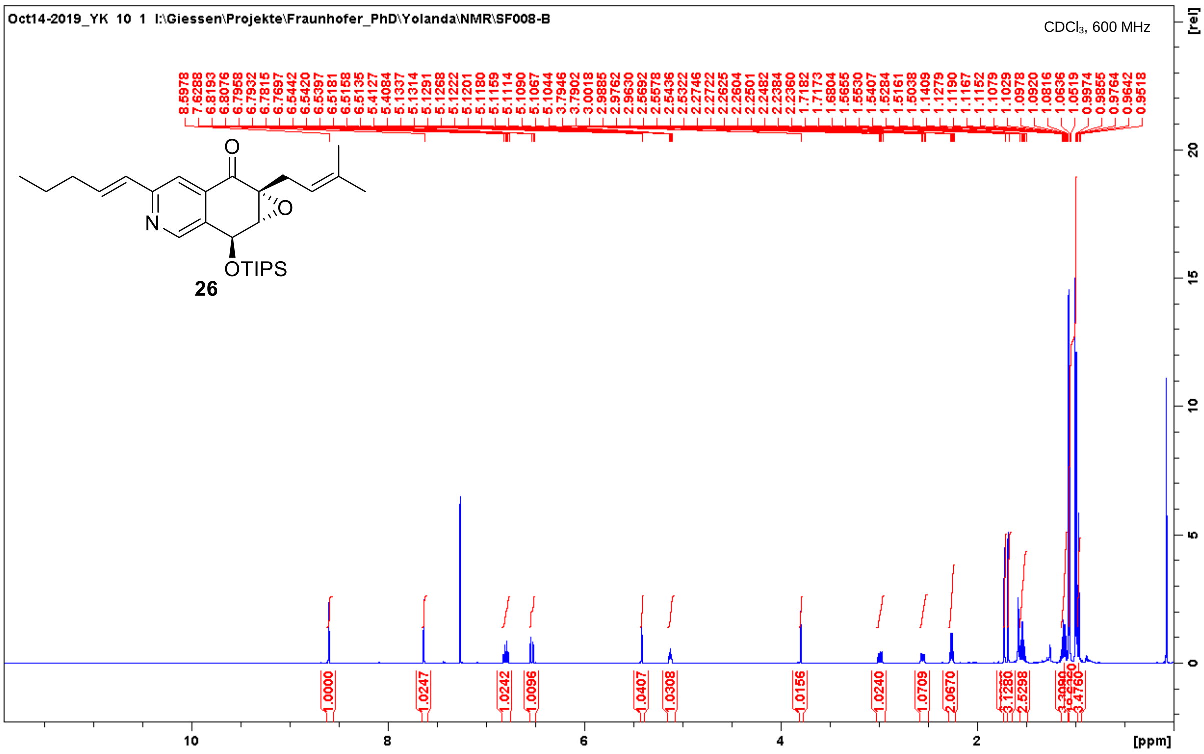
Oct14-2019_YK 10 1 I:\Giessen\Projekte\Fraunhofer_PhD\Yolanda\NMR\SF008-B
CDCl3, 600 MHz
OTIPS
26
[ppm]
[rel]

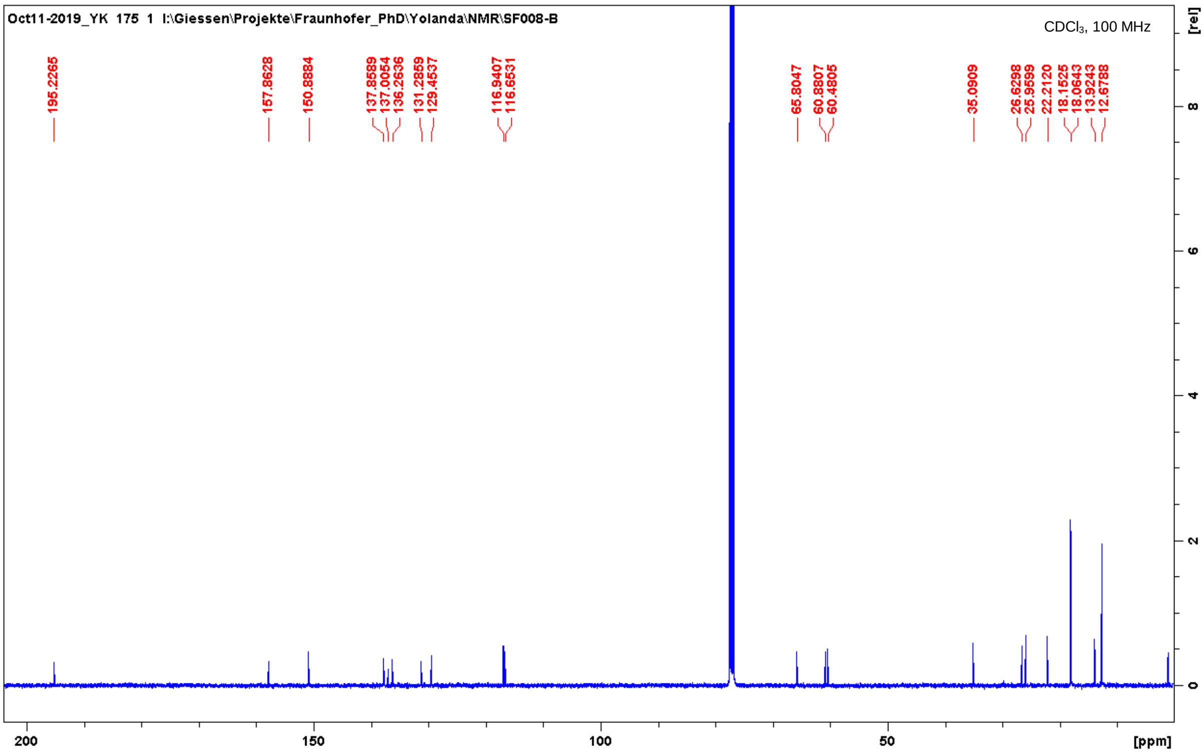
Oct11-2019_YK 175 1 I:\Giessen\Projekte\Fraunhofer_PhD\Yolanda\NMR\SF008-B
CDCl3, 100 MHz
195.2265
157.8628
150.8884
137.8589
137.0054
136.2636
131.2859
129.4537
116.9407
116.6531
65.8047
60.8807
60.4805
35.0909
26.6298
25.9599
22.2120
18.1525
18.0643
13.9243
12.6788
[ppm]
[rel]

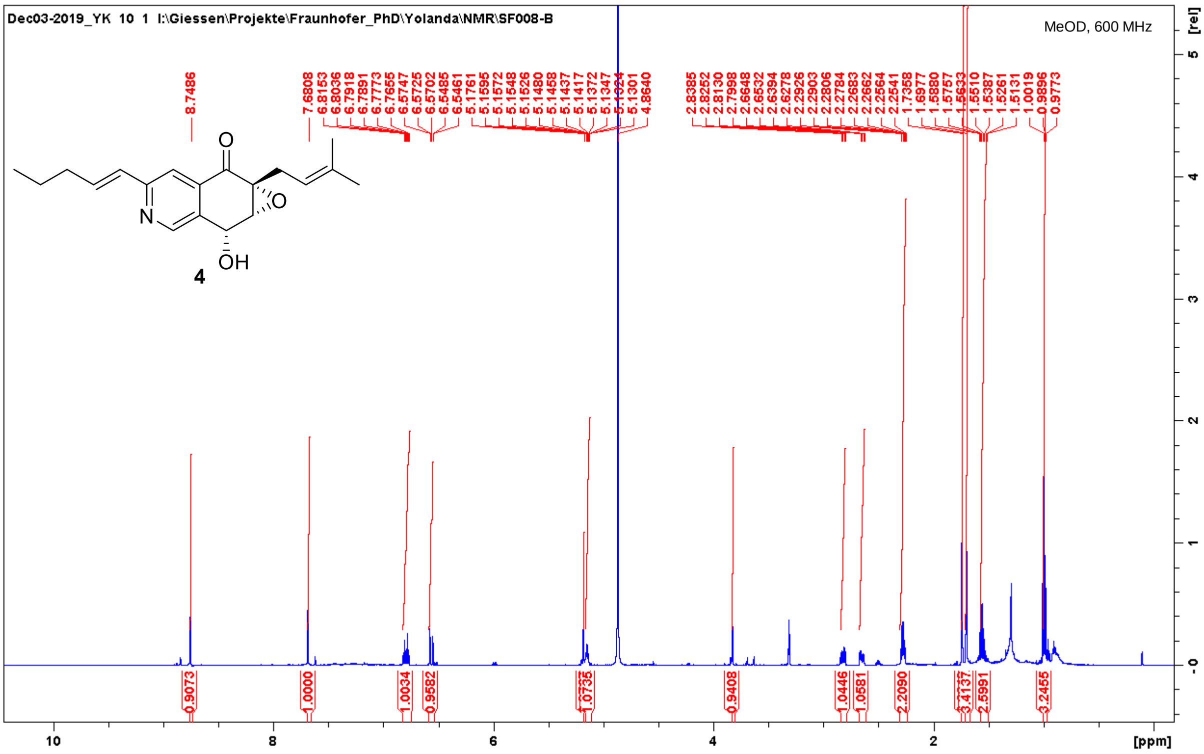

Dec03-2019_YK 10 1 I:\Giessen\Projekte\Fraunhofer_PhD\Yolanda\NMR\SF008-B
MeOD, 600 MHz
8.7486
7.6808
6.8153
6.8036
6.7918
6.7891
6.7773
6.7655
6.5747
6.5725
6.5702
6.5485
6.5461
5.1761
5.1595
5.1572
5.1548
5.1526
5.1480
5.1458
5.1437
5.1417
5.1372
5.1347
5.1324
5.1301
4.8640
2.8385
2.8252
2.8130
2.7998
2.6648
2.6532
2.6394
2.6278
2.2926
2.2903
2.2806
2.2784
2.2683
2.2662
2.2564
2.2541
1.7358
1.6977
1.5880
1.5757
1.5633
1.5510
1.5387
1.5261
1.5131
1.0019
0.9896
0.9773
O
N
O
OH
4
0.9073
1.0000
1.0034
0.9582
1.0735
0.9408
1.0446
1.0581
2.2090
3.4137
2.5991
3.2455
10
8
6
4
2
[ppm]
[rel]

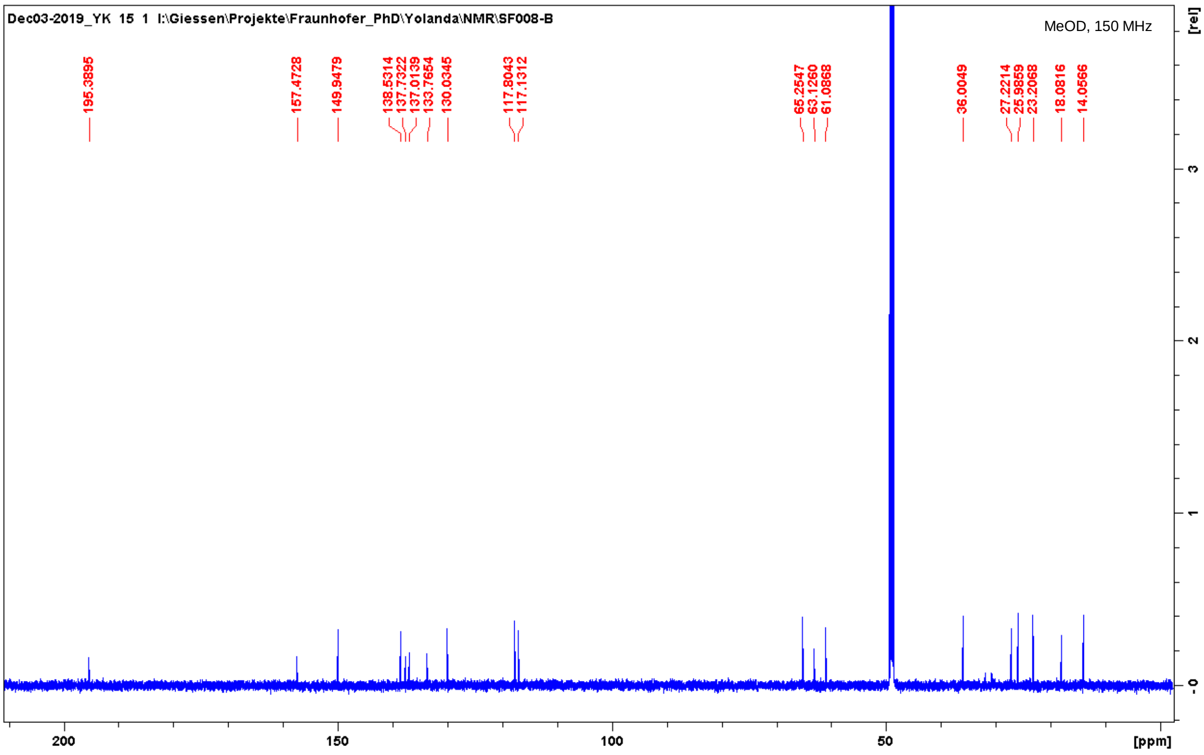

Dec03-2019_YK 15 1 I:\Giessen\Projekte\Fraunhofer_PhD\Yolanda\NMR\SF008-B
MeOD, 150 MHz
195.3895
157.4728
149.9479
138.5314
137.7322
137.0139
133.7654
130.0345
117.8043
117.1312
65.2547
63.1260
61.0868
36.0049
27.2214
25.9859
23.2068
18.0816
14.0566
200
150
100
50
[ppm]
[rel]

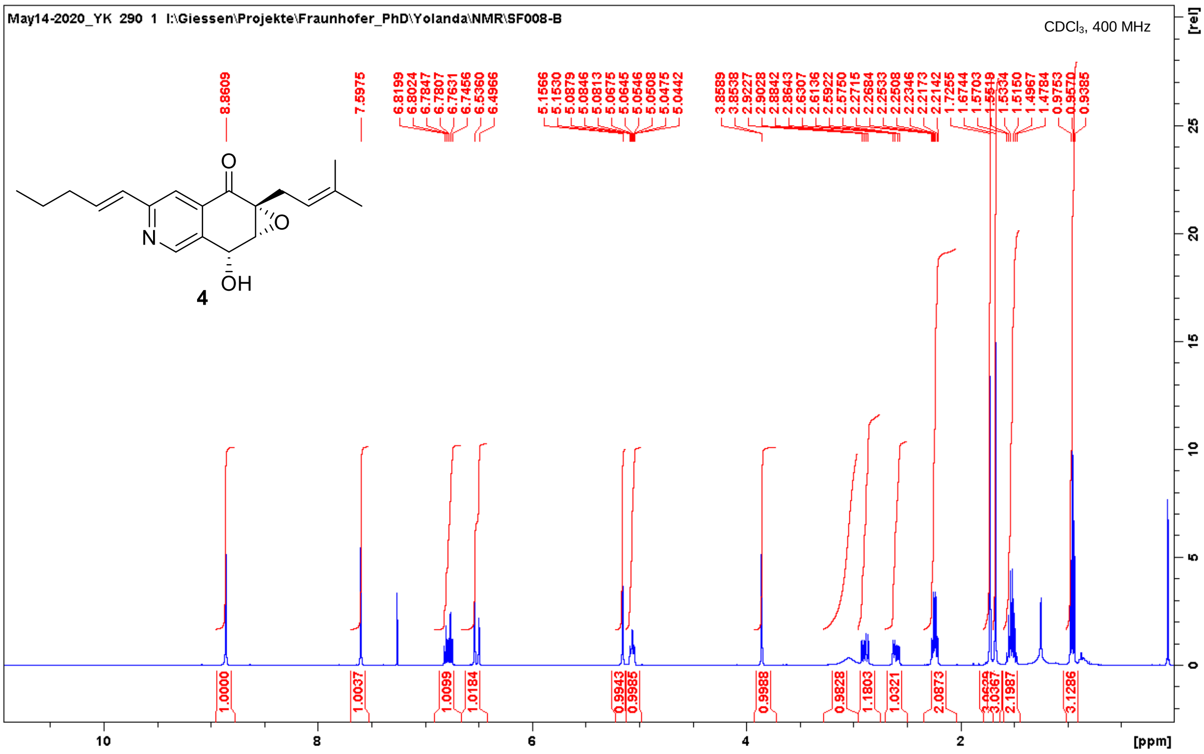
May14-2020_YK 290 1 I:\Giessen\Projekte\Fraunhofer_PhD\Yolanda\NMR\SF008-B
CDCl3, 400 MHz
4
[ppm]
[rel]

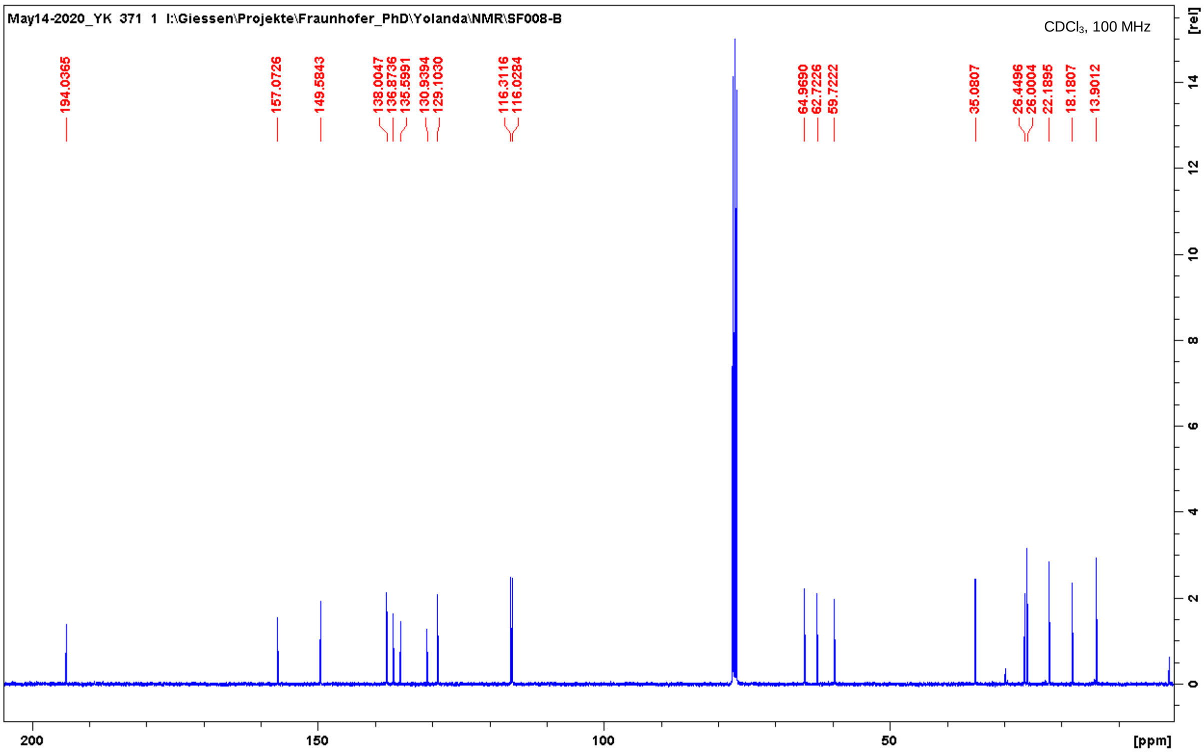
May14-2020_YK 371 1 I:\Giessen\Projekte\Fraunhofer_PhD\Yolanda\NMR\SF008-B
CDCl3, 100 MHz
194.0365
157.0726
149.5843
138.0047
136.8736
135.5991
130.9394
129.1030
116.3116
116.0284
64.9690
62.7226
59.7222
35.0807
26.4496
26.0004
22.1895
18.1807
13.9012
[ppm]
[rel]

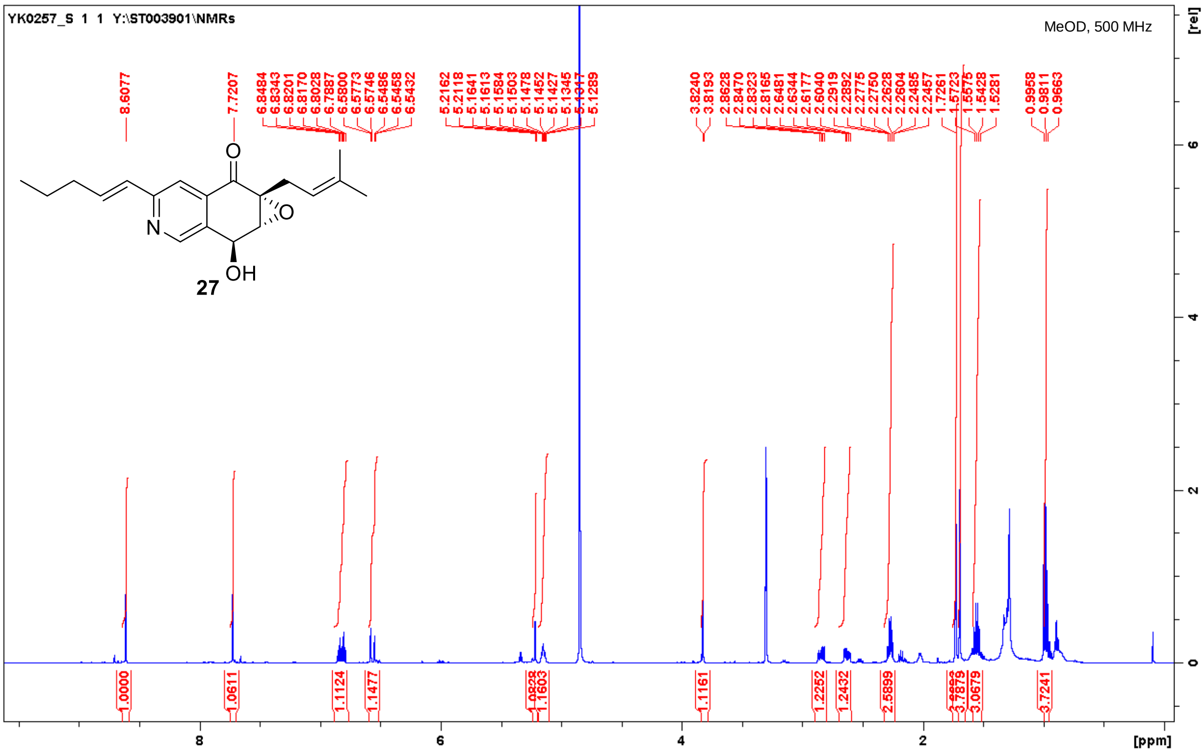
YK0257_S 1 1 Y:\ST003901\NMRs
MeOD, 500 MHz
27
[ppm]
[rel]

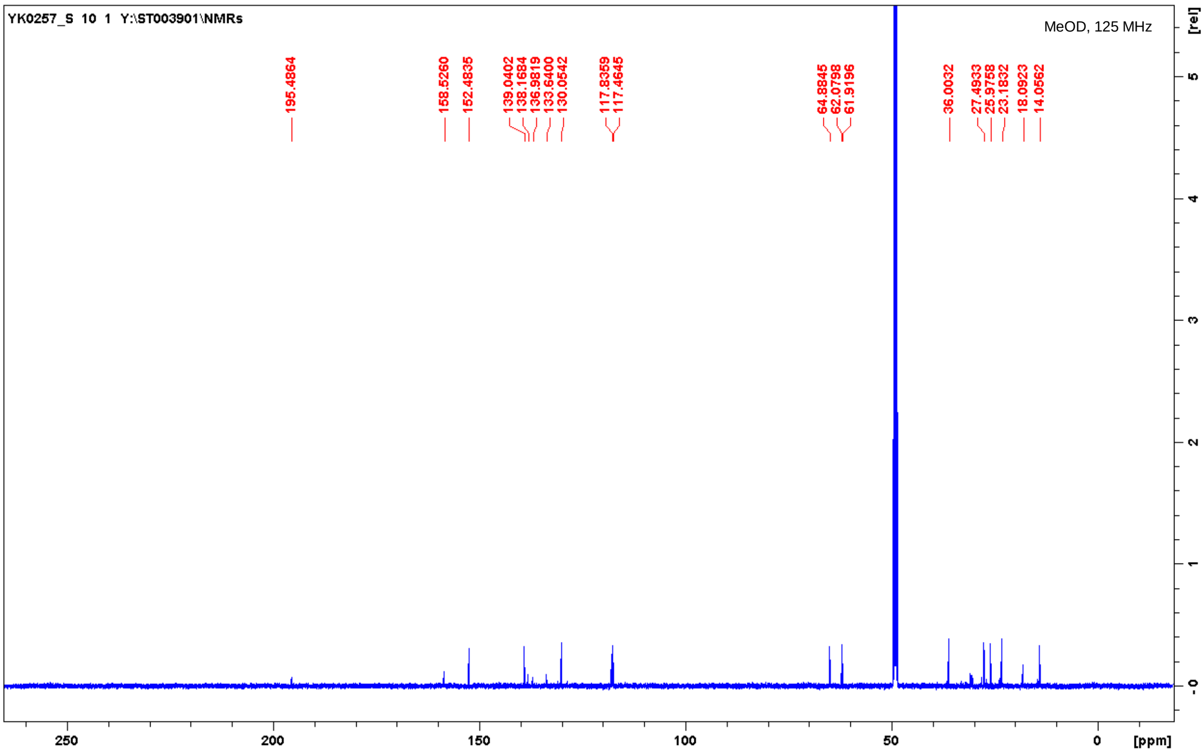
YK0257_S 10 1 Y:\ST003901\NMRs
MeOD, 125 MHz
195.4864
158.5260
152.4835
139.0402
138.1684
136.9819
133.6400
130.0542
117.8359
117.4645
64.8845
62.0798
61.9196
36.0032
27.4933
25.9758
23.1832
18.0923
14.0562
[ppm]
[rel]

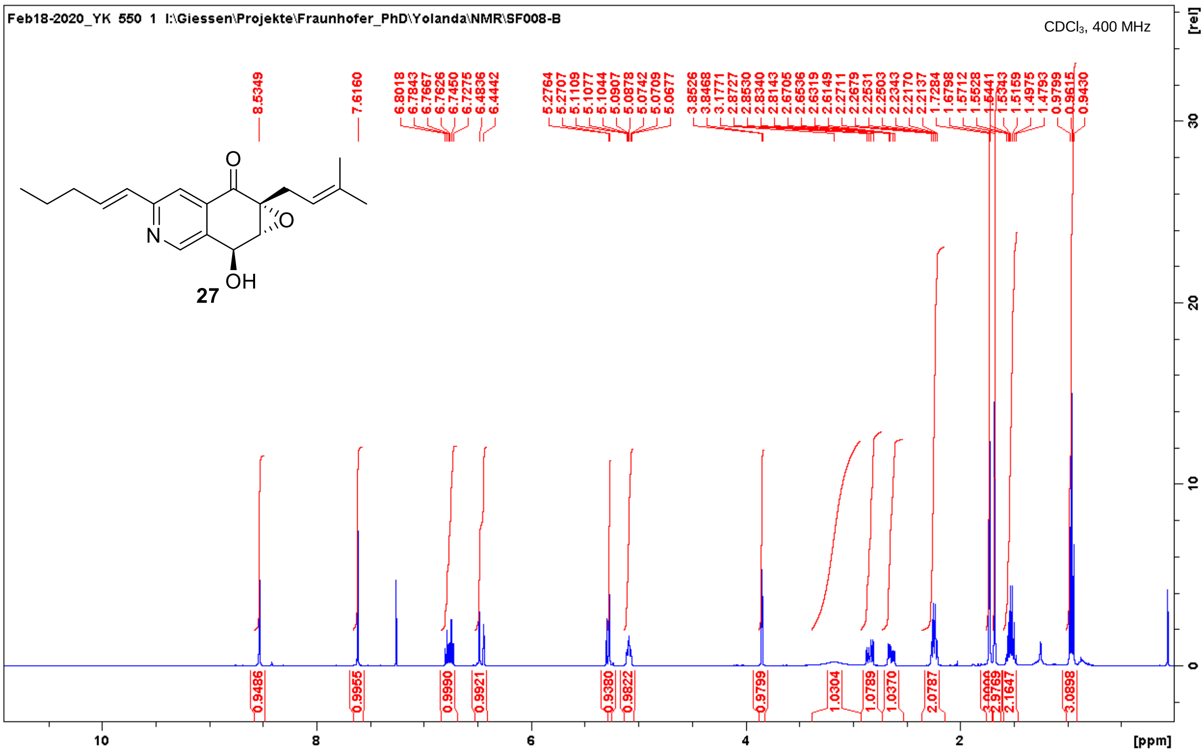
Feb18-2020_YK 550 1 I:\Giessen\Projekte\Fraunhofer_PhD\Yolanda\NMR\SF008-B
CDCl3, 400 MHz
27
OH
O
N
[ppm]
[rel]

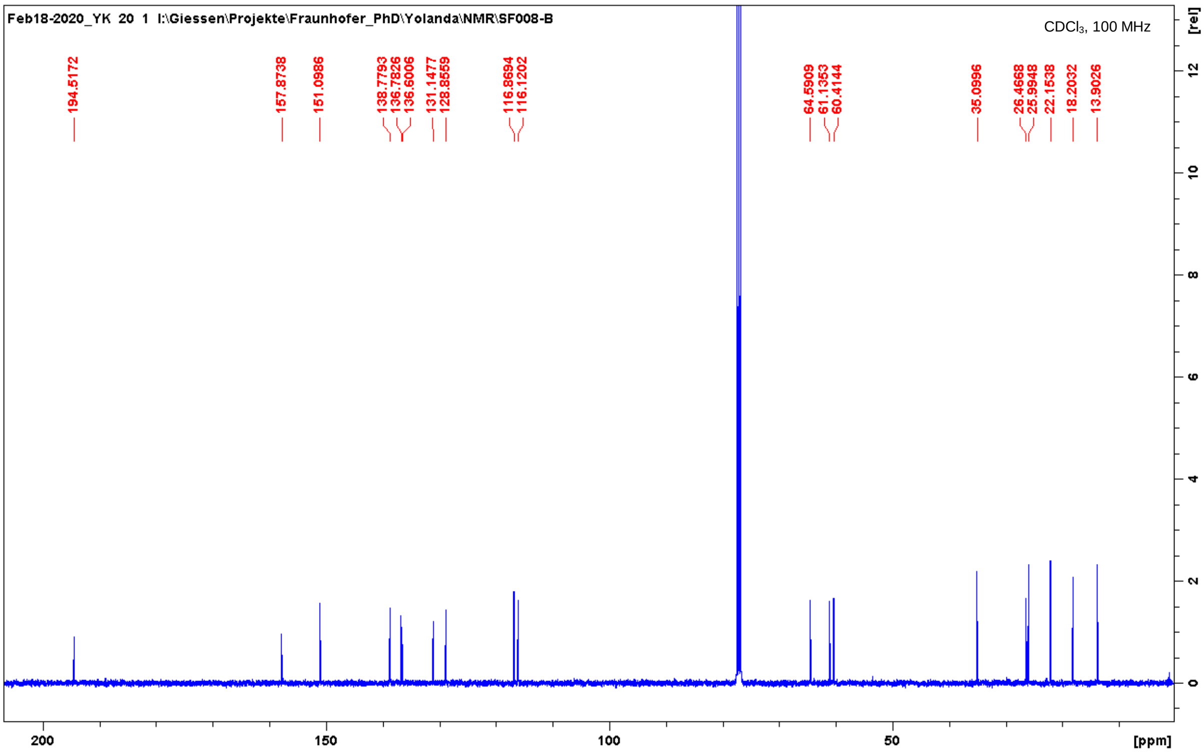
Feb18-2020_YK 20 1 I:\Giessen\Projekte\Fraunhofer_PhD\Yolanda\NMR\SF008-B
CDCl3, 100 MHz
194.5172
157.8738
151.0986
138.7793
136.7826
136.6006
131.1477
128.8559
116.8694
116.1202
64.5909
61.1353
60.4144
35.0996
26.4668
25.9948
22.1538
18.2032
13.9026
[ppm]
[rel]

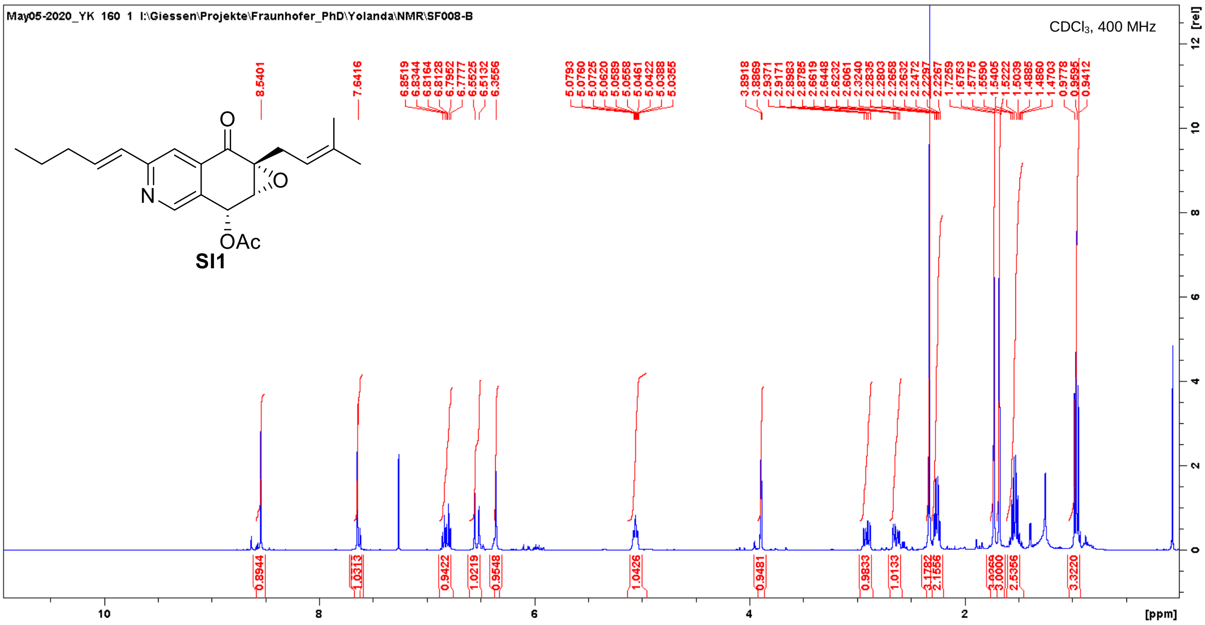

May05-2020_YK 160 1 I:\Giessen\Projekte\Fraunhofer_PhD\Yolanda\NMR\SF008-B
CDCl3, 400 MHz
8.5401
7.6416
6.8519 6.8344 6.8164 6.8128 6.7952 6.7777 6.5525 6.5132 6.3556
5.0793 5.0760 5.0725 5.0620 5.0589 5.0558 5.0461 5.0422 5.0388 5.0355
3.8918 3.8869 2.9371 2.9171 2.8983 2.8785 2.6619 2.6448 2.6232 2.6061 2.3240 2.2835 2.2803 2.2658 2.2632 2.2472 2.2297 2.2267 1.7259 1.6753 1.5775 1.5590 1.5405 1.5222 1.5039 1.4885 1.4860 1.4703 0.9778 0.9595 0.9412
O
N
O
OAc
SI1
0.8944
1.0313
0.9422
1.0219
0.9548
1.0426
0.9481
0.9833
1.0133
3.1782
2.1556
3.0000
2.5356
3.3220
10
8
6
4
2
[ppm]
[rel]

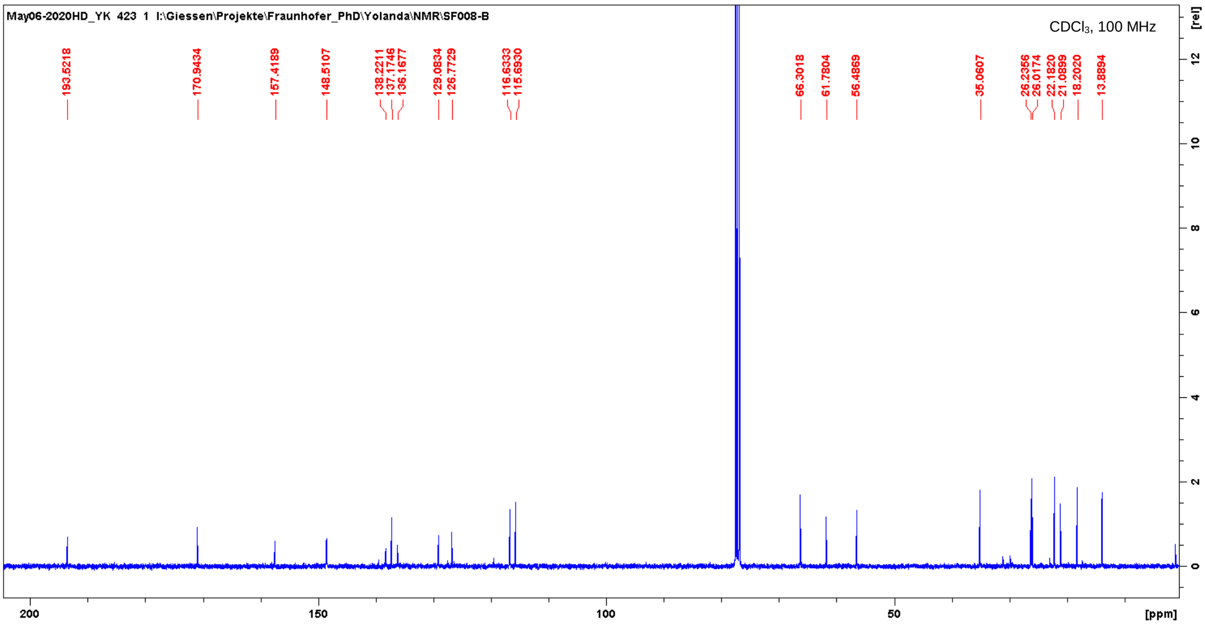

May06-2020HD_YK 423 1 I:\Giessen\Projekte\Fraunhofer_PhD\Yolanda\NMR\SF008-B
CDCl3, 100 MHz
193.5218
170.9434
157.4189
148.5107
138.2211 137.1746 136.1677
129.0834 126.7729
116.6333 115.6930
66.3018
61.7804
56.4869
35.0607
26.2356 26.0174 22.1820 21.0899 18.2020
13.8894
200
150
100
50
[ppm]
[rel]

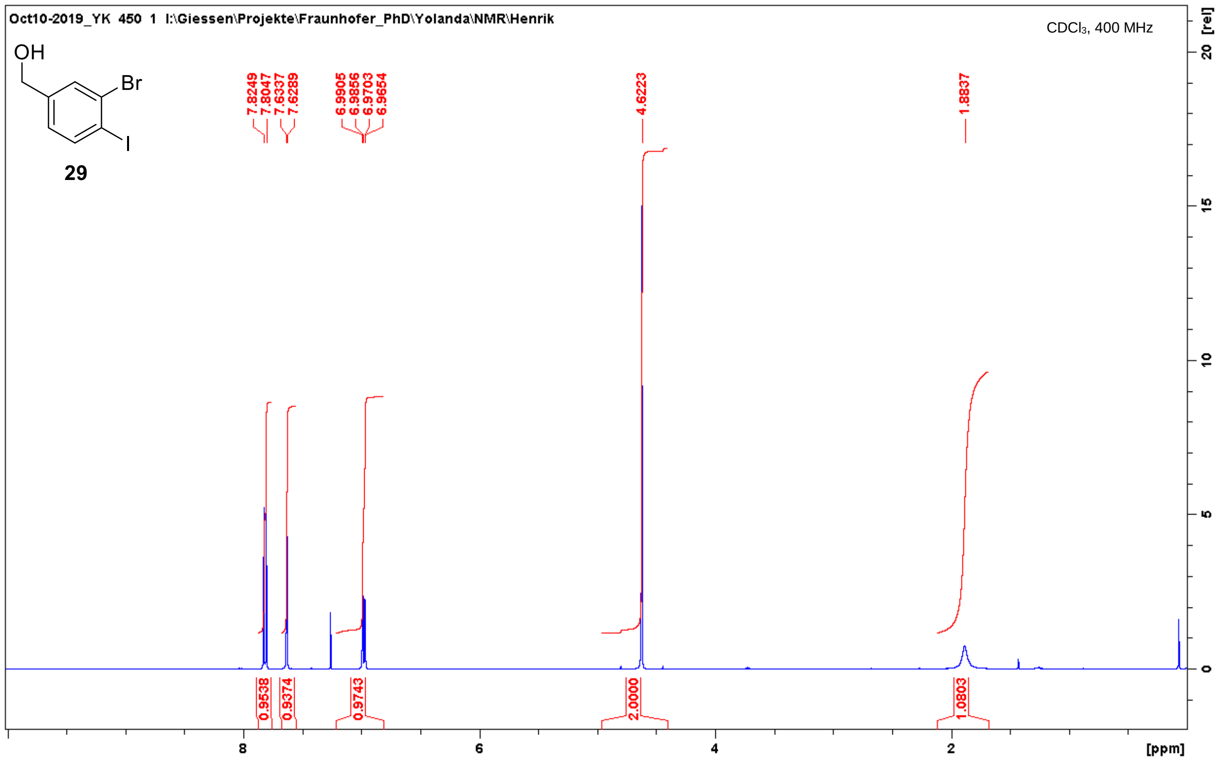

Oct10-2019_YK 450 1 I:\Giessen\Projekte\Fraunhofer_PhD\Yolanda\NMR\Henrik
CDCl3, 400 MHz
OH
Br
I
29
7.8249
7.8047
7.6337
7.6289
6.9905
6.9856
6.9703
6.9654
4.6223
1.8837
0.9538
0.9374
0.9743
2.0000
1.0803
[ppm]
[rel]

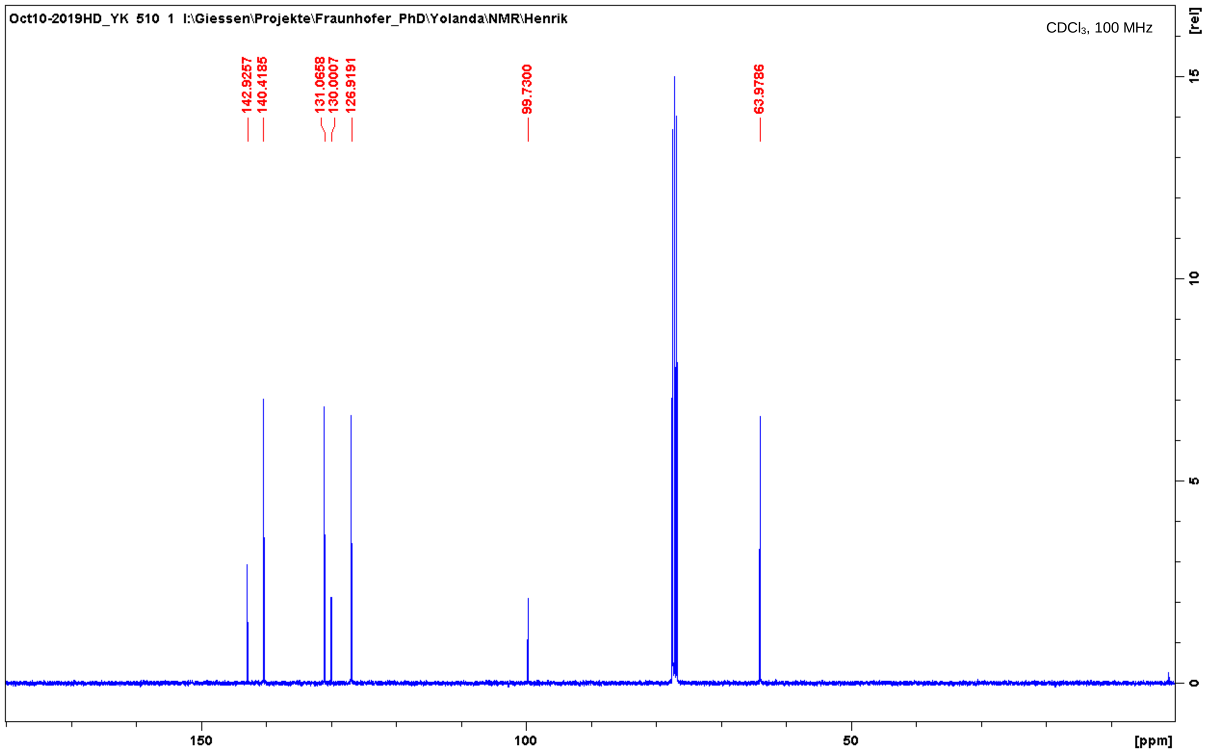

Oct10-2019HD_YK 510 1 I:\Giessen\Projekte\Fraunhofer_PhD\Yolanda\NMR\Henrik
CDCl3, 100 MHz
142.9257
140.4185
131.0658
130.0007
126.9191
99.7300
63.9786
[ppm]
[rel]

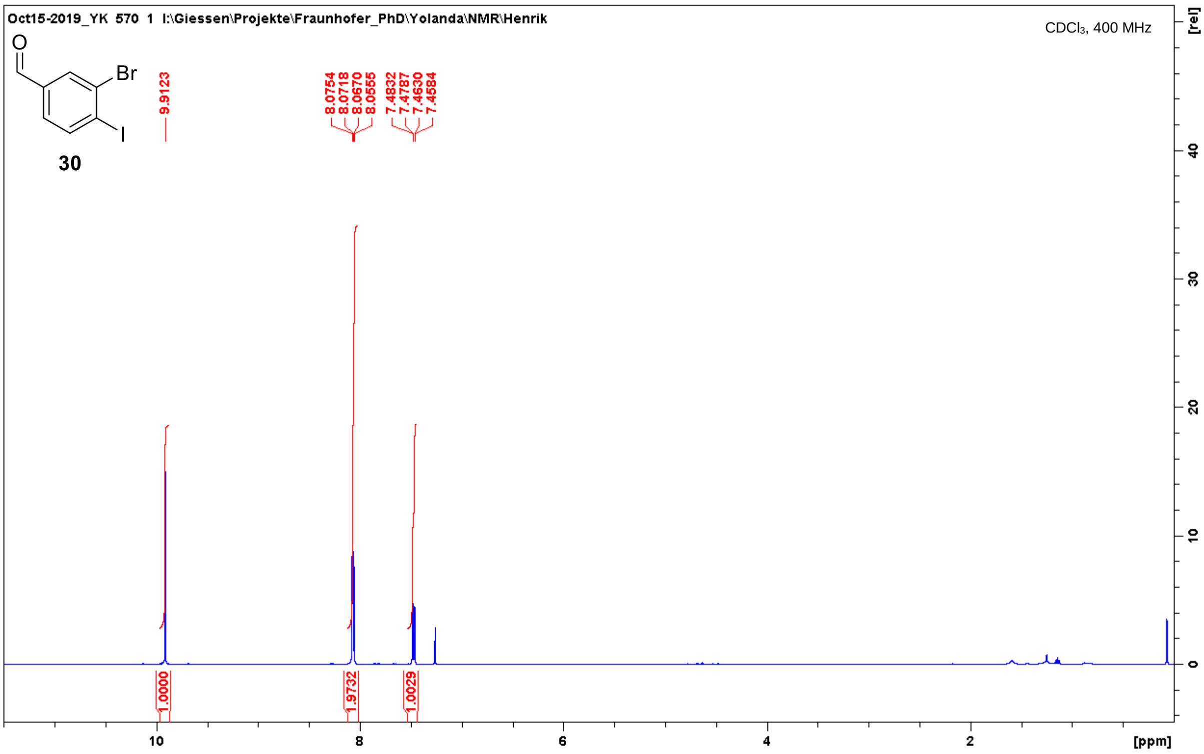
Oct15-2019_YK 570 1 I:\Giessen\Projekte\Fraunhofer_PhD\Yolanda\NMR\Henrik
CDCl3, 400 MHz
O
Br
I
30
9.9123
8.0754
8.0718
8.0670
8.0555
7.4832
7.4787
7.4630
7.4584
1.0000
1.9732
1.0029
[rel]
[ppm]

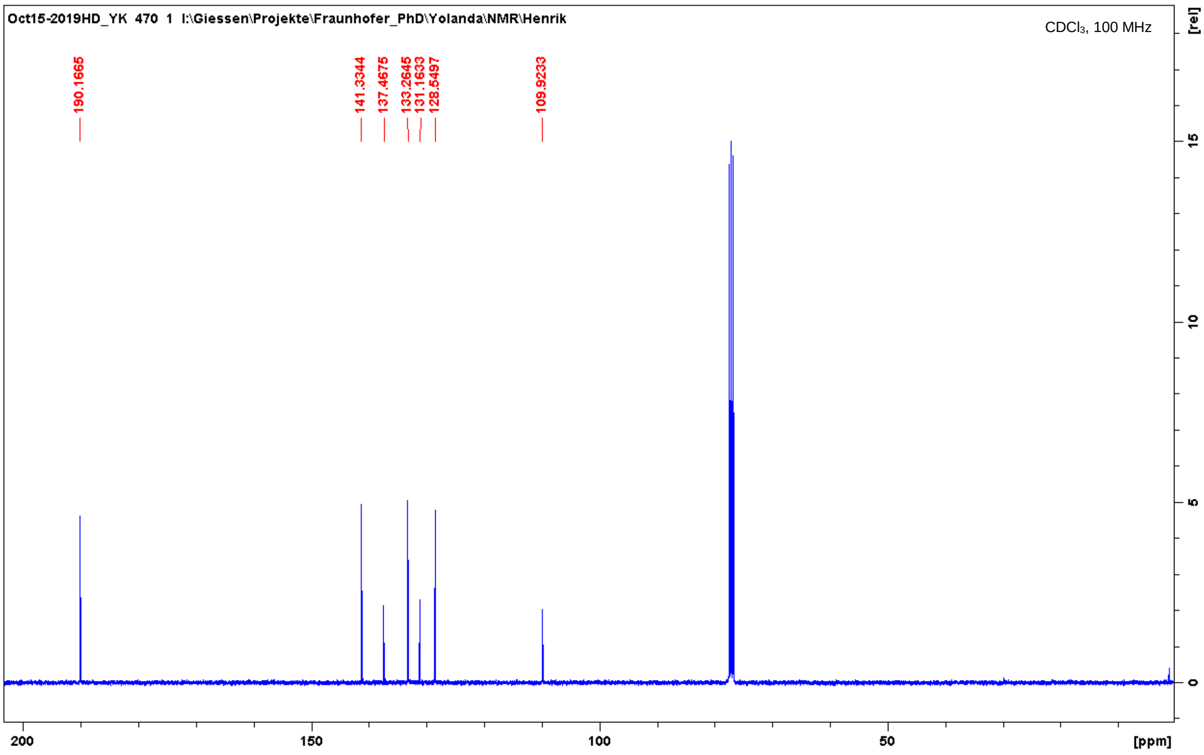
Oct15-2019HD_YK 470 1 I:\Giessen\Projekte\Fraunhofer_PhD\Yolanda\NMR\Henrik
CDCl3, 100 MHz
190.1665
141.3344
137.4675
133.2645
131.1633
128.5497
109.9233
[rel]
[ppm]

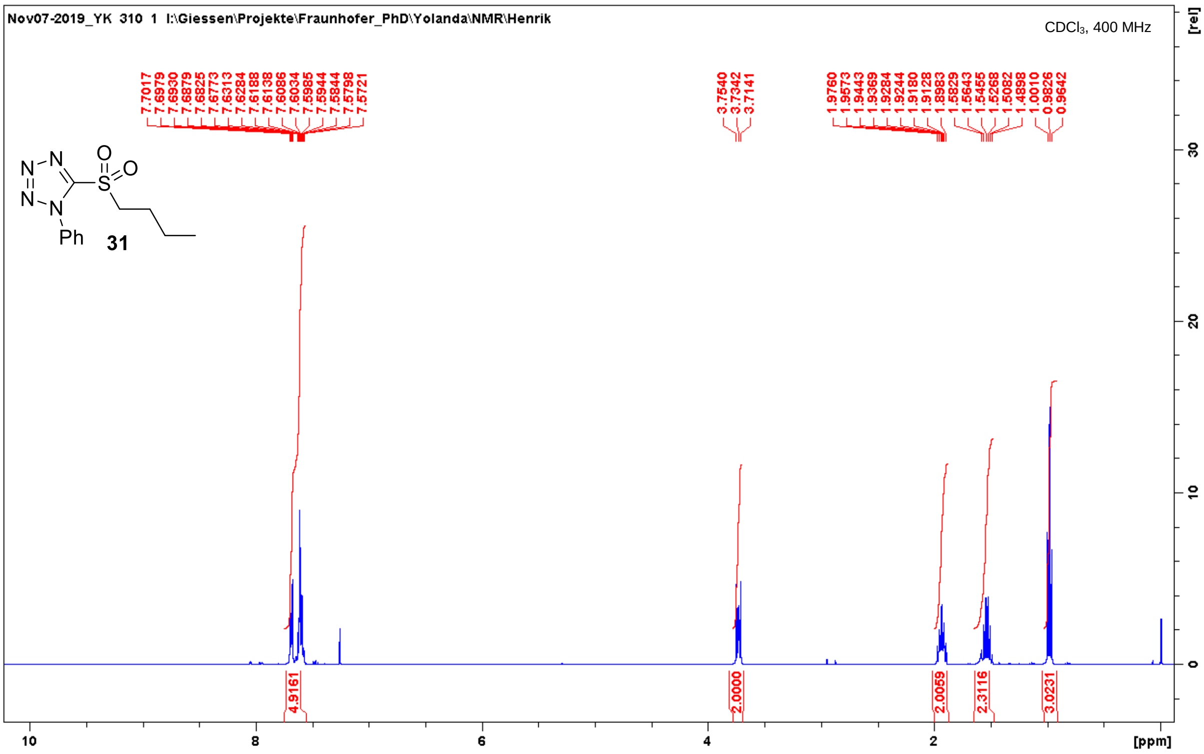

Nov07-2019_YK 310 1 I:\Giessen\Projekte\Fraunhofer_PhD\Yolanda\NMR\Henrik
CDCl3, 400 MHz
7.7017 7.6979 7.6930 7.6879 7.6825 7.6773 7.6313 7.6284 7.6188 7.6138 7.6086 7.6034 7.5985 7.5944 7.5844 7.5798 7.5721
3.7540 3.7342 3.7141
1.9760 1.9573 1.9443 1.9369 1.9284 1.9244 1.9180 1.9128 1.8983 1.5829 1.5643 1.5455 1.5268 1.5082 1.4898 1.0010 0.9826 0.9642
31
Ph
4.9161
2.0000
2.0059
2.3116
3.0231
[ppm]
[rel]

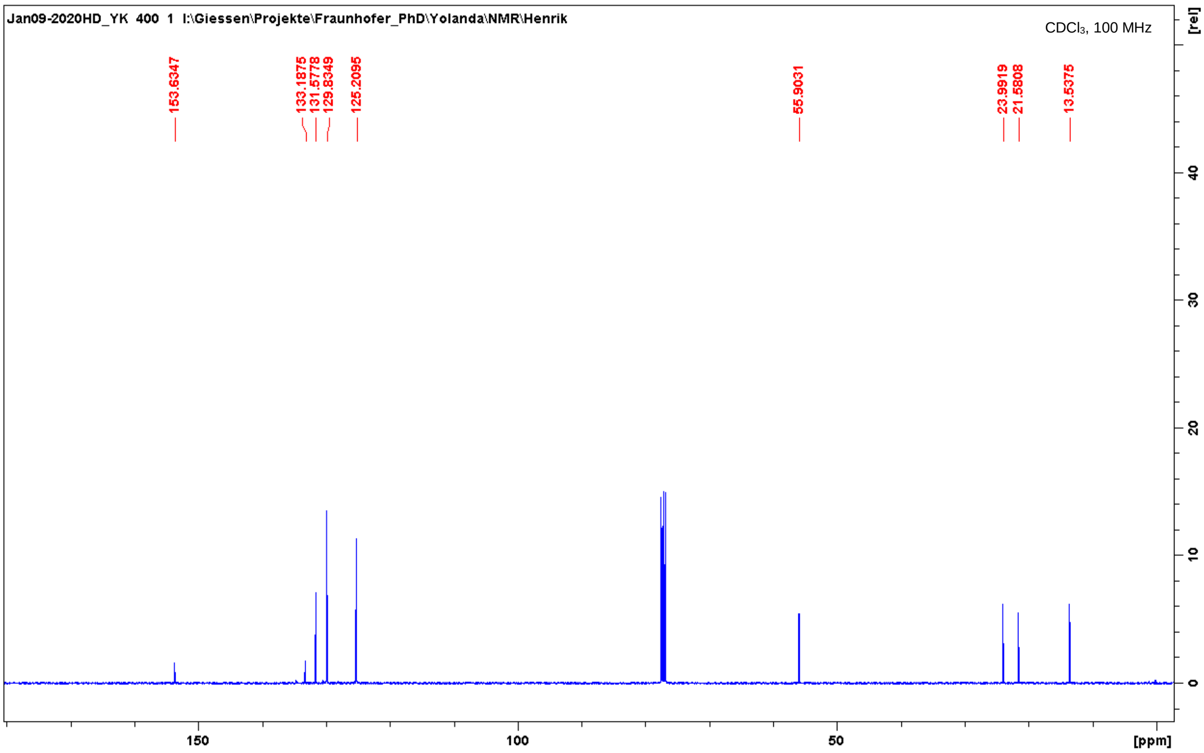

Jan09-2020HD_YK 400 1 I:\Giessen\Projekte\Fraunhofer_PhD\Yolanda\NMR\Henrik
CDCl3, 100 MHz
153.6347
133.1875 131.5778 129.8349 125.2095
55.9031
23.9919 21.5808 13.5375
[ppm]
[rel]

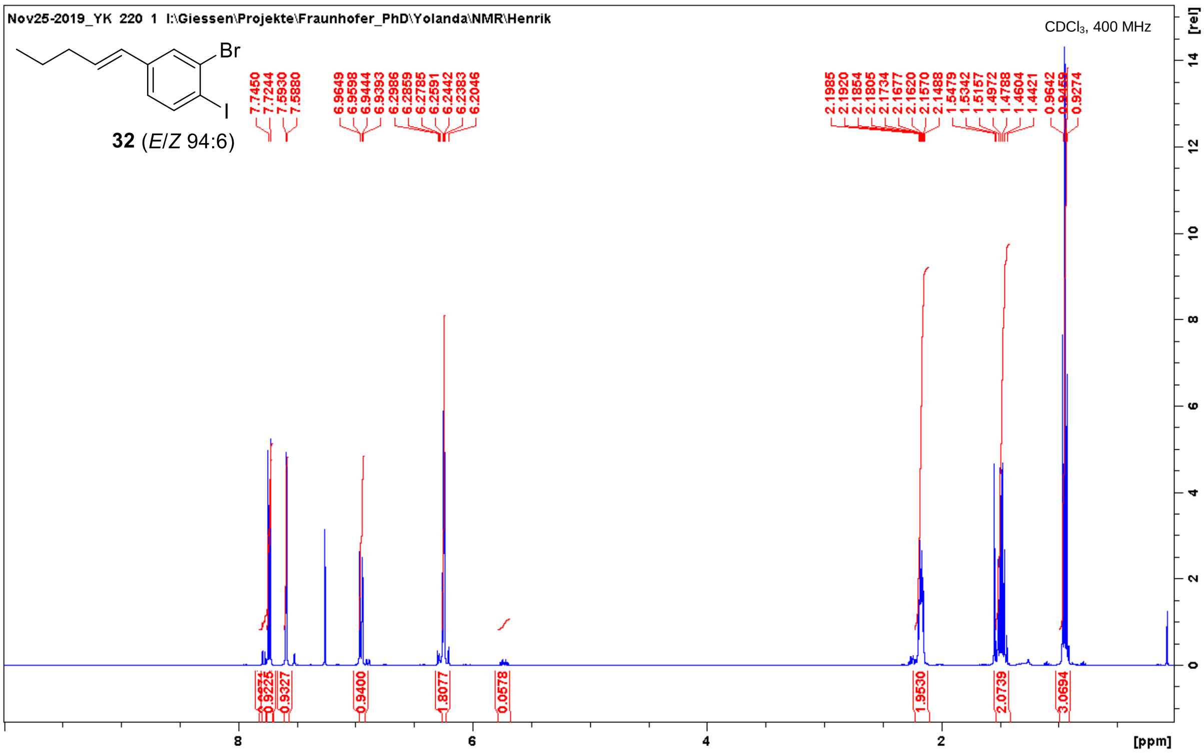
Nov25-2019_YK 220 1 I:\Giessen\Projekte\Fraunhofer_PhD\Yolanda\NMR\Henrik
CDCl3, 400 MHz
Br
I
32 (E/Z 94:6)
7.7450
7.7244
7.5930
7.5880
6.9649
6.9598
6.9444
6.9393
6.2986
6.2859
6.2785
6.2591
6.2442
6.2383
6.2046
2.1985
2.1920
2.1854
2.1805
2.1734
2.1677
2.1620
2.1570
2.1488
1.5479
1.5342
1.5157
1.4972
1.4788
1.4604
1.4421
0.9642
0.9274
0.9225
0.9327
0.9400
1.8077
0.0578
1.9530
2.0739
3.0694
[ppm]
[rel]

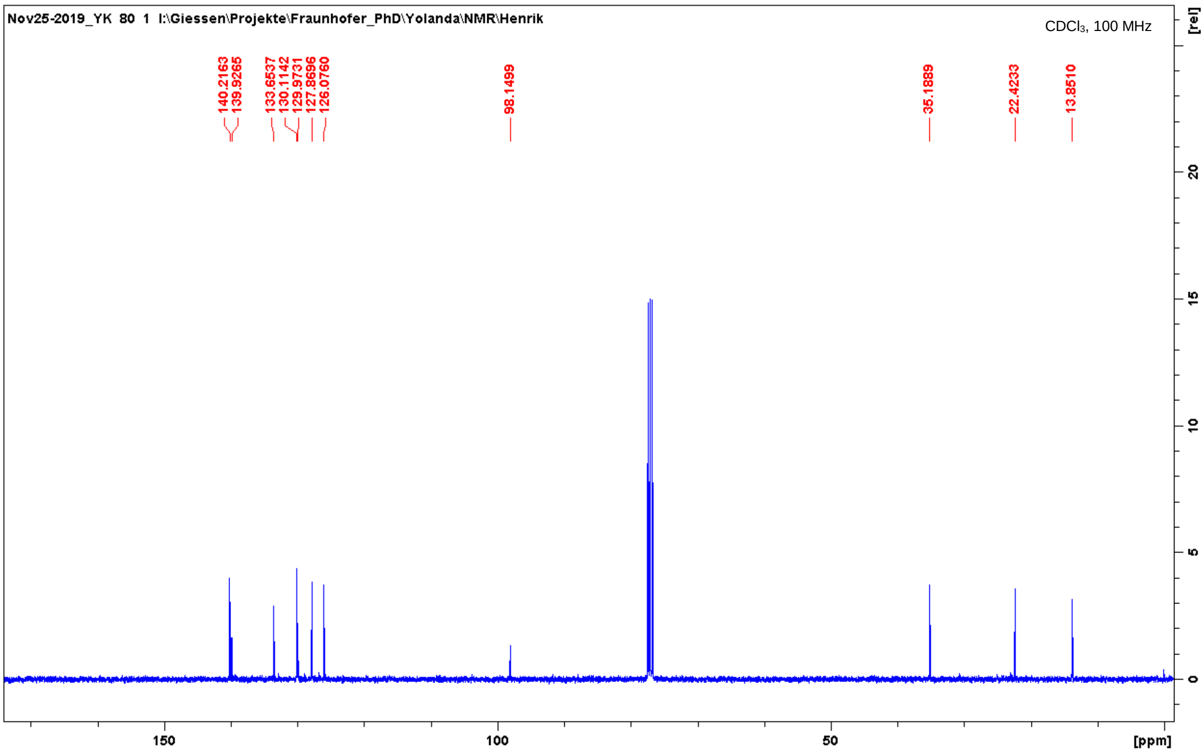
Nov25-2019_YK 80 1 I:\Giessen\Projekte\Fraunhofer_PhD\Yolanda\NMR\Henrik
CDCl3, 100 MHz
140.2163
139.9265
133.6537
130.1142
129.9731
127.8696
126.0760
98.1499
35.1889
22.4233
13.8510
[ppm]
[rel]

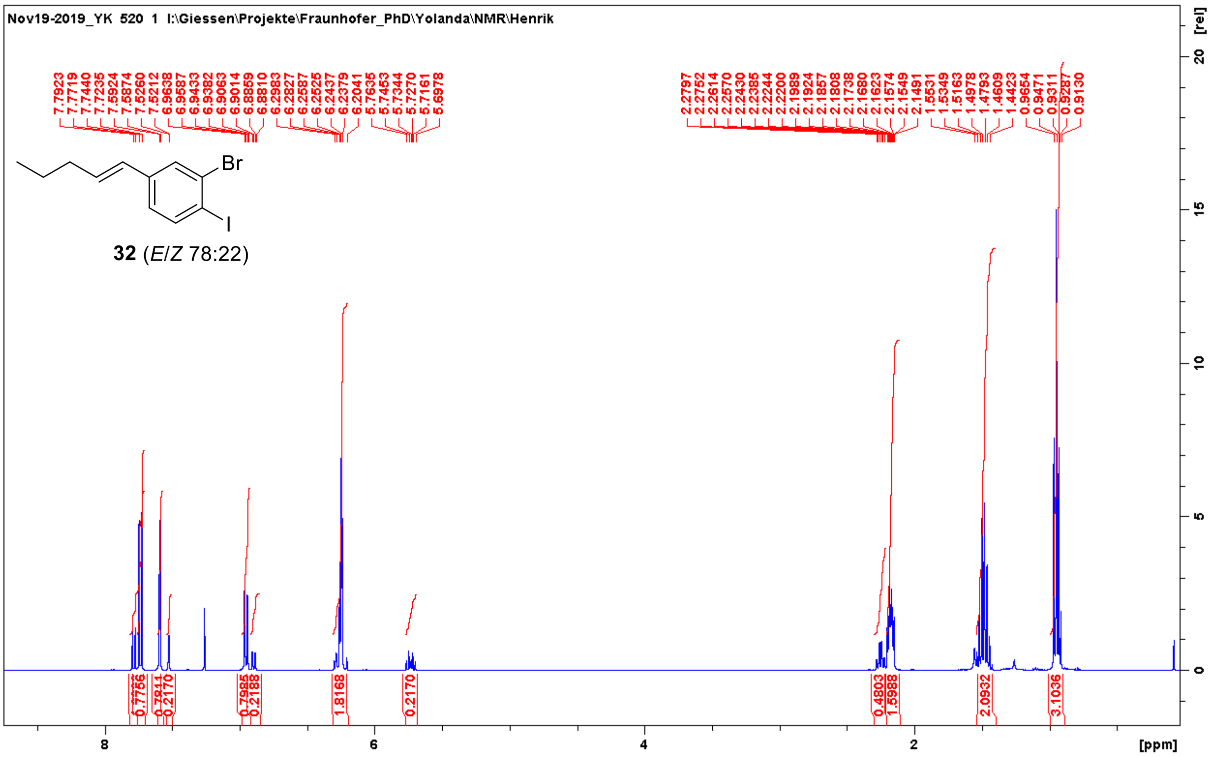
Nov19-2019_YK 520 1 I:\Giessen\Projekte\Fraunhofer_PhD\Yolanda\NMR\Henrik
Br
I
32 (E/Z 78:22)
[ppm]
[rel]

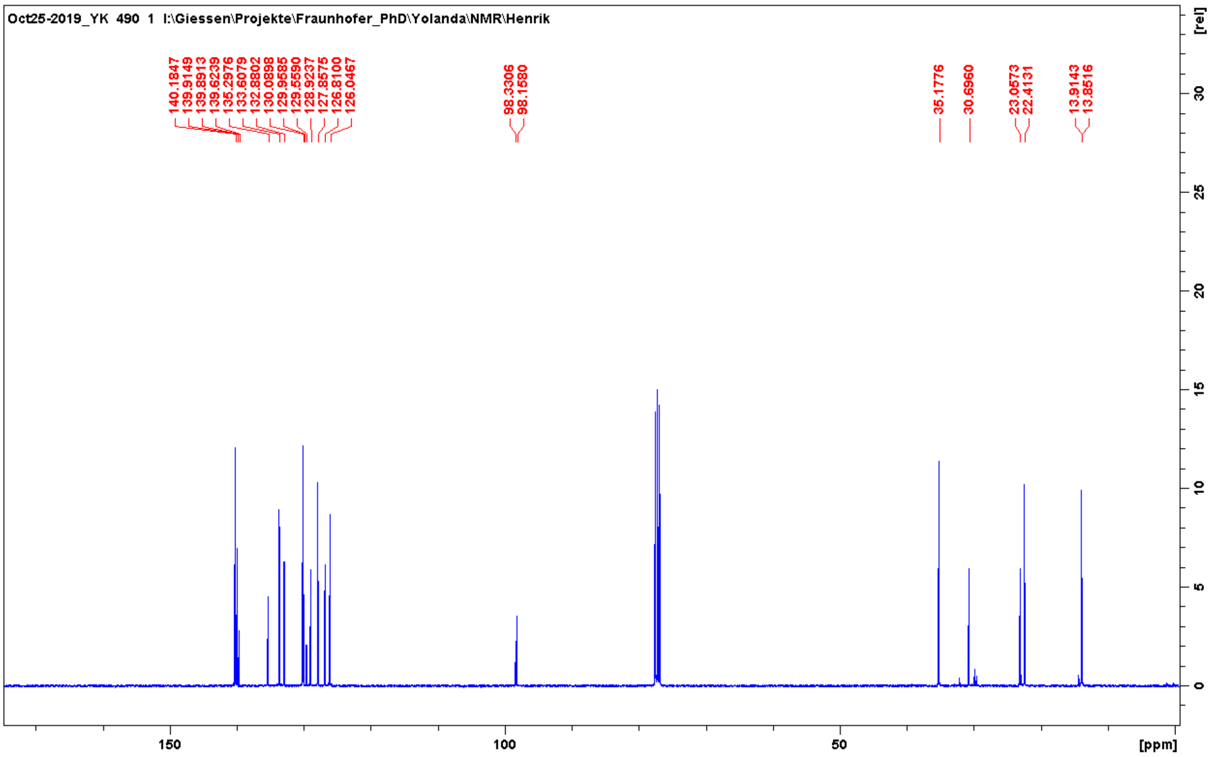
Oct25-2019_YK 490 1 I:\Giessen\Projekte\Fraunhofer_PhD\Yolanda\NMR\Henrik
140.1847
139.9149
139.8913
139.6239
135.2976
133.6079
132.8802
130.0898
129.9585
129.5590
128.9237
127.8575
126.8100
126.0467
98.3306
98.1580
35.1776
30.6960
23.0573
22.4131
13.9143
13.8516
[ppm]
[rel]

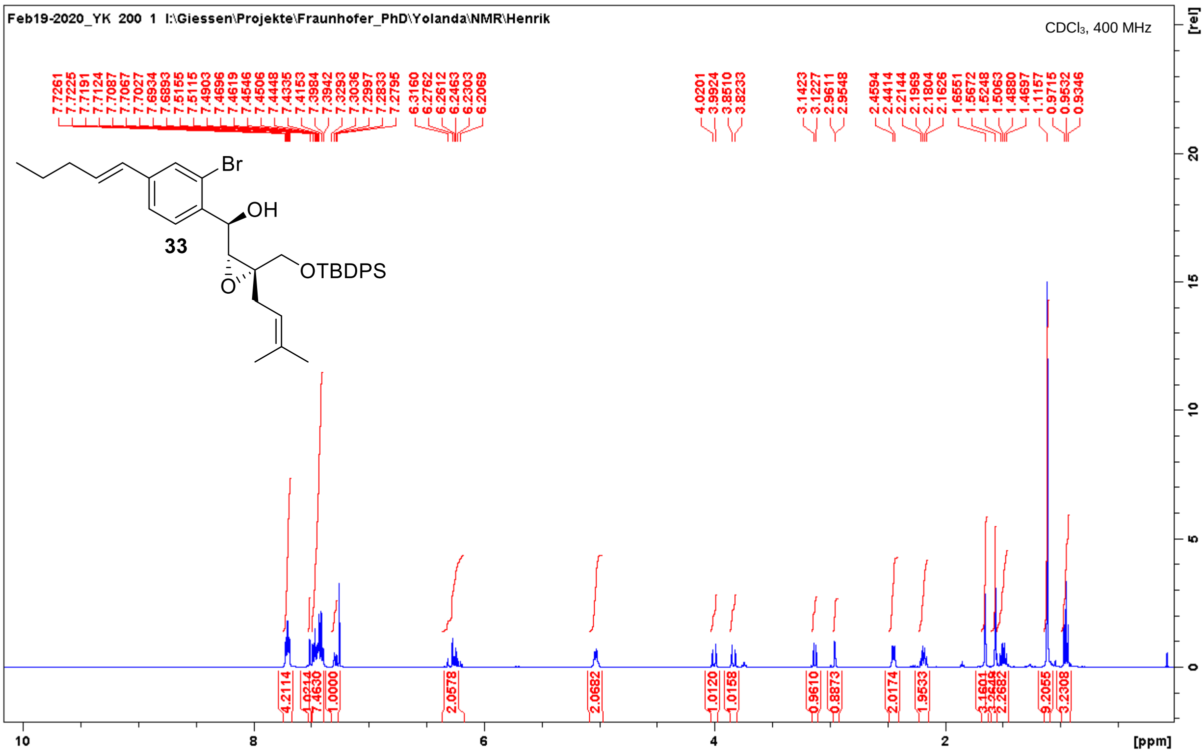

Feb19-2020_YK 200 1 I:\Giessen\Projekte\Fraunhofer_PhD\Yolanda\NMR\Henrik
CDCl3, 400 MHz
Br
OH
33
OTBDPS
O
[ppm]
[rel]

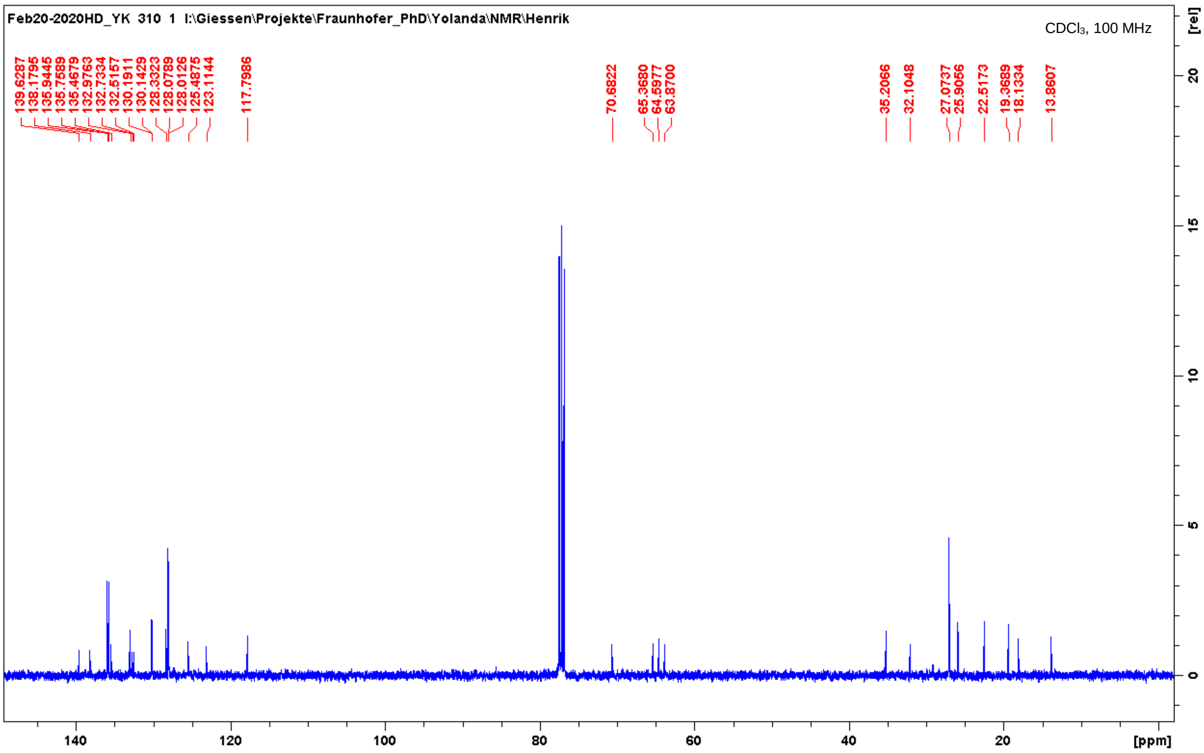

Feb20-2020HD_YK 310 1 I:\Giessen\Projekte\Fraunhofer_PhD\Yolanda\NMR\Henrik
CDCl3, 100 MHz
139.6287
138.1795
135.9445
135.7589
135.4679
132.9763
132.7334
132.5157
130.1911
130.1429
128.3323
128.0789
128.0126
125.4875
123.1144
117.7986
70.6822
65.3680
64.5977
63.8700
35.2066
32.1048
27.0737
25.9056
22.5173
19.3689
18.1334
13.8607
[ppm]
[rel]

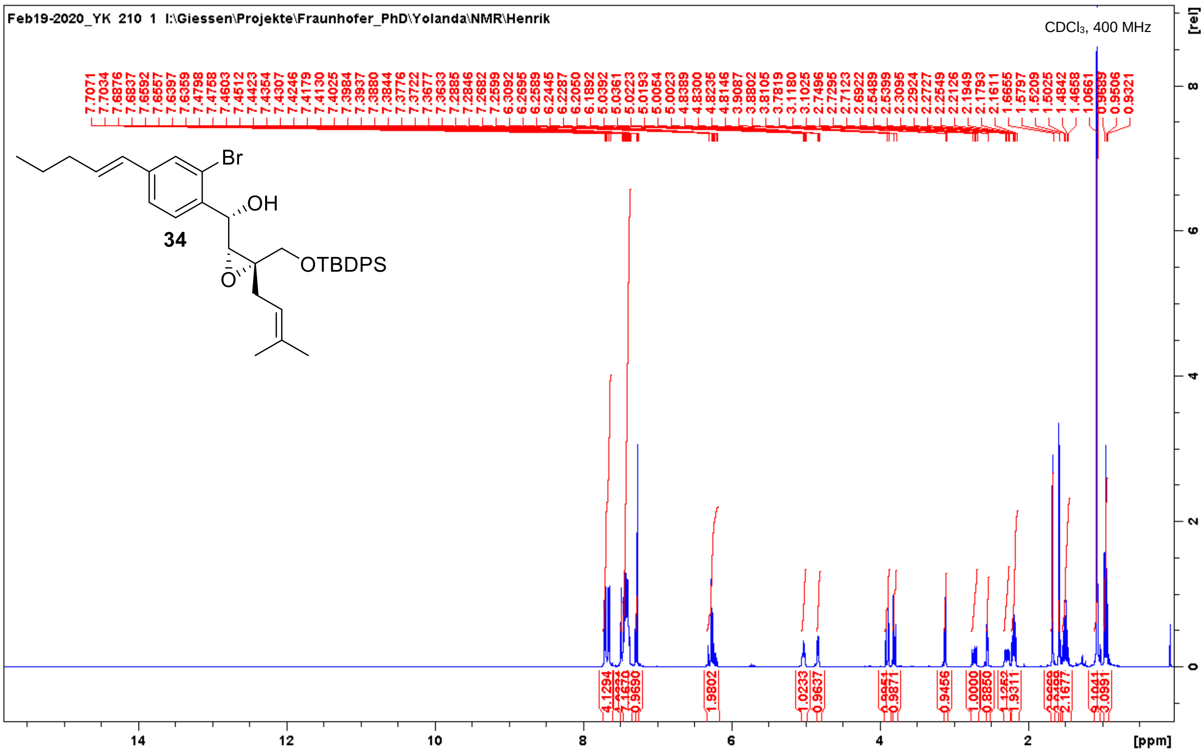
Feb19-2020_YK 210 1 I:\Giessen\Projekte\Fraunhofer_PhD\Yolanda\NMR\Henrik
CDCl3, 400 MHz
Br
OH
34
OTBDPS
O
[ppm]
[rel]

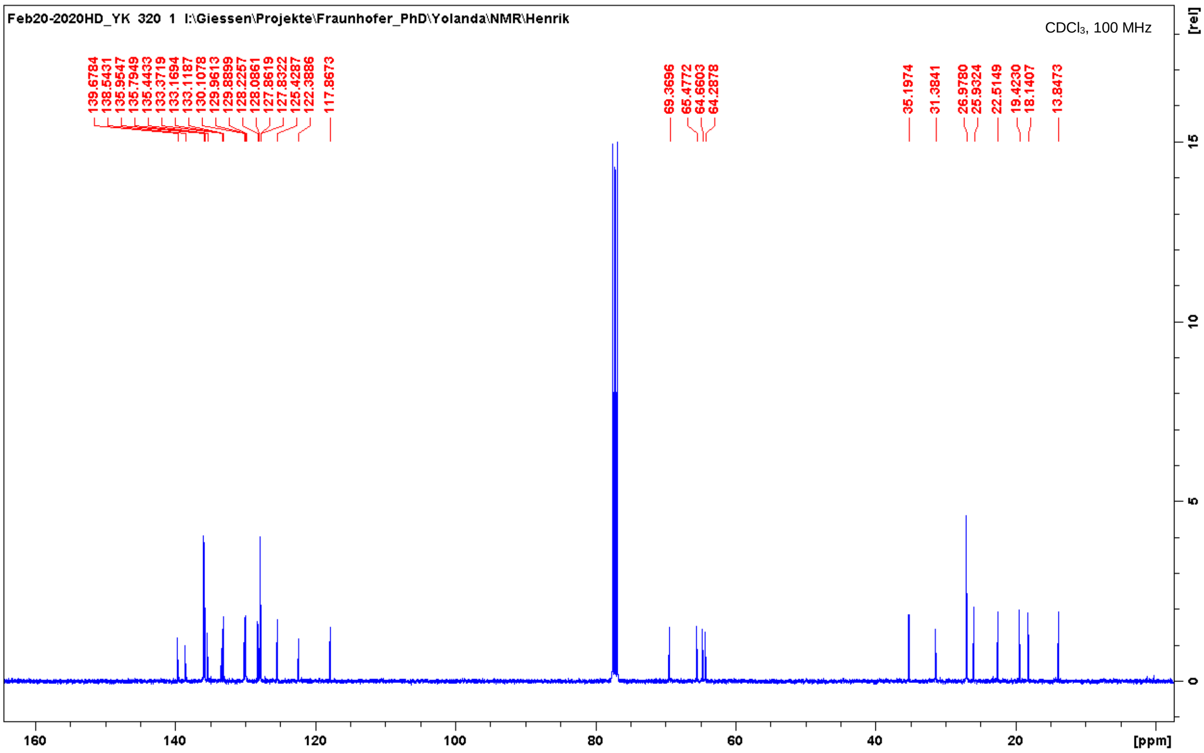
Feb20-2020HD_YK 320 1 I:\Giessen\Projekte\Fraunhofer_PhD\Yolanda\NMR\Henrik
CDCl3, 100 MHz
139.6784
138.5431
135.9547
135.7949
135.4433
133.3719
133.1694
133.1187
130.1078
129.9613
129.8899
128.2257
128.0861
127.8619
127.8322
125.4287
122.3886
117.8673
69.3696
65.4772
64.6603
64.2878
35.1974
31.3841
26.9780
25.9324
22.5149
19.4230
18.1407
13.8473
[ppm]
[rel]

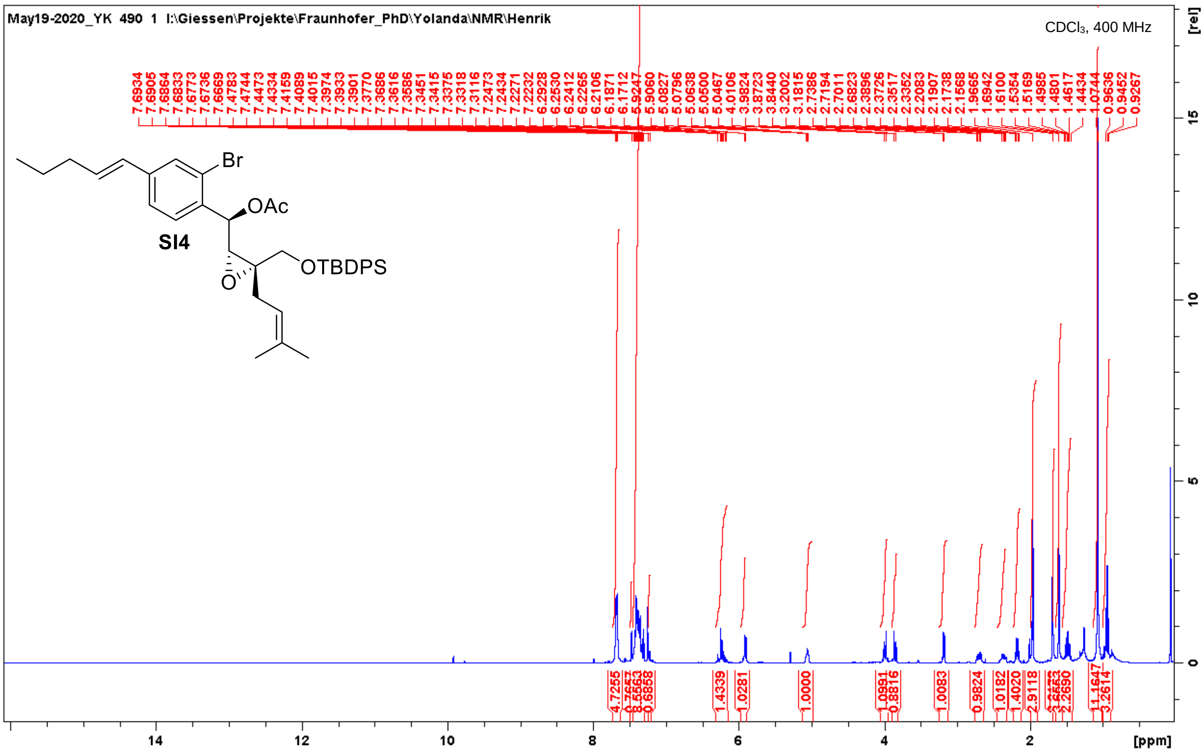
May19-2020_YK 490 1 I:\Giessen\Projekte\Fraunhofer_PhD\Yolanda\NMR\Henrik
CDCl3, 400 MHz
SI4
Br
OAc
OTBDPS
O
[ppm]
[rel]

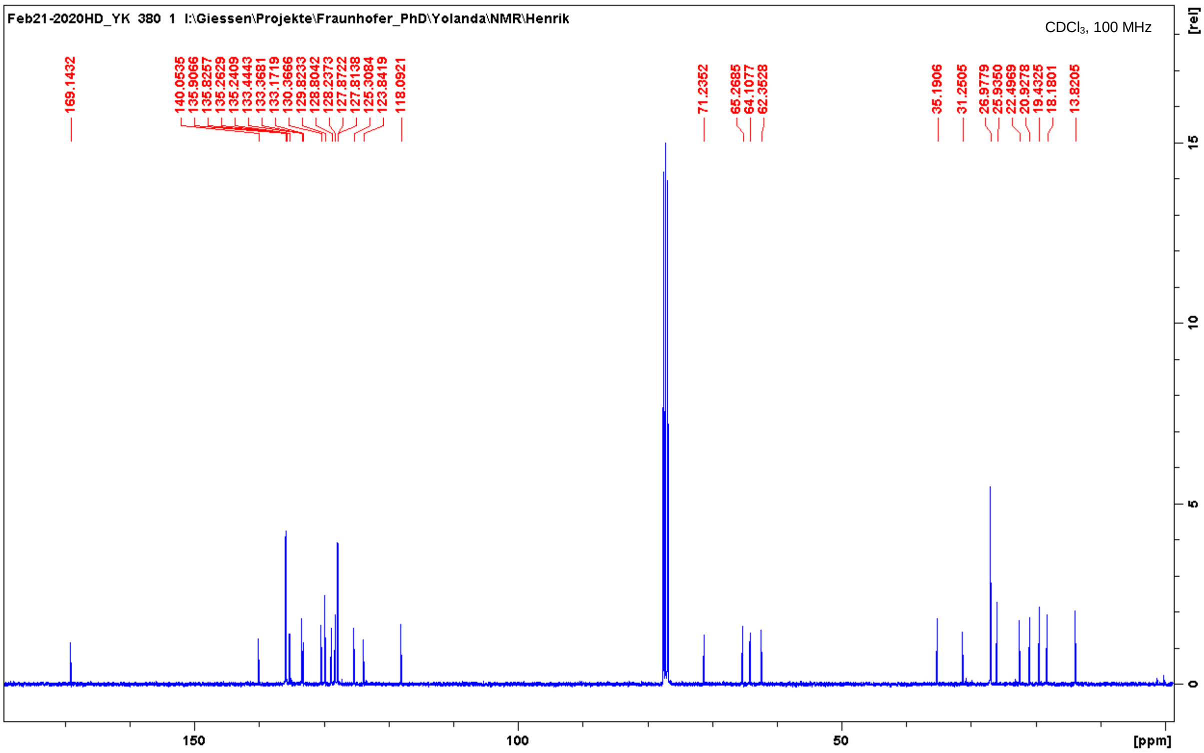
Feb21-2020HD_YK 380 1 I:\Giessen\Projekte\Fraunhofer_PhD\Yolanda\NMR\Henrik
CDCl3, 100 MHz
169.1432
140.0535
135.9066
135.8257
135.2629
135.2409
133.4443
133.3681
133.1719
130.3666
129.8233
128.8042
128.2373
127.8722
127.8138
125.3084
123.8419
118.0921
71.2352
65.2685
64.1077
62.3528
35.1906
31.2505
26.9779
25.9350
22.4969
20.9278
19.4325
18.1801
13.8205
[ppm]
[rel]

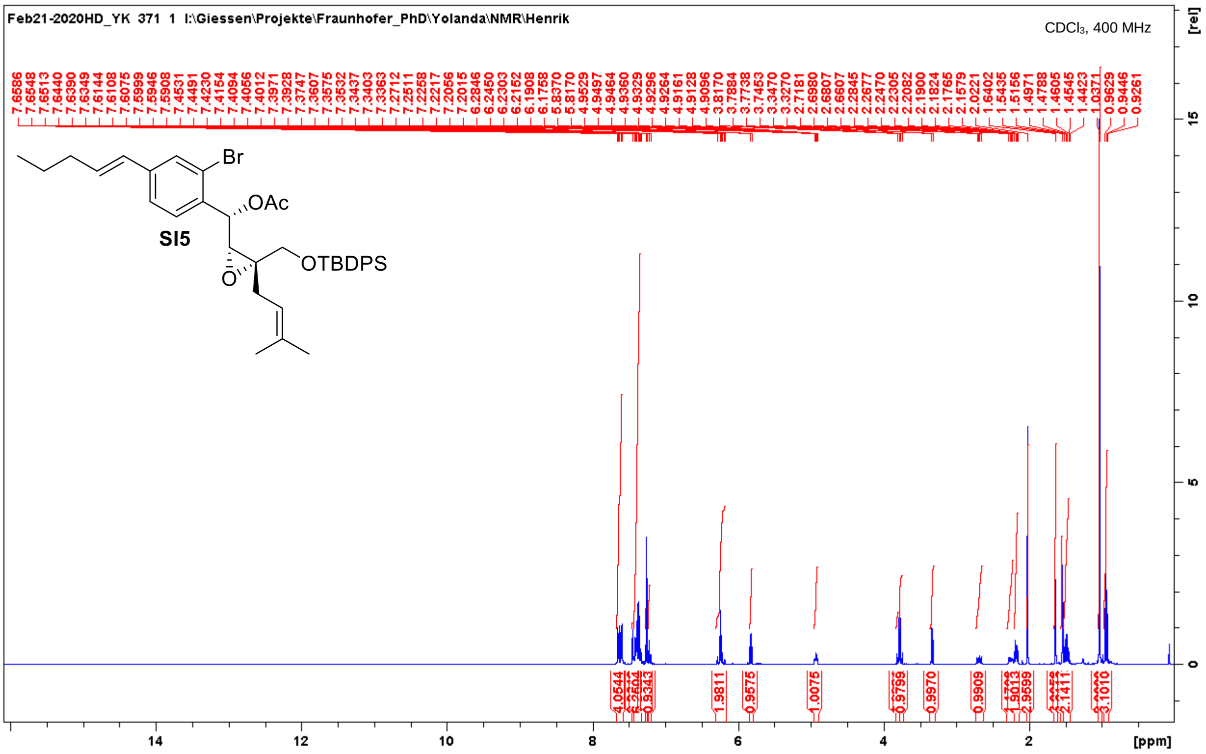
Feb21-2020HD_YK 371 1 I:\Giessen\Projekte\Fraunhofer_PhD\Yolanda\NMR\Henrik
CDCl$_3$, 400 MHz
7.6586 7.6548 7.6513 7.6440 7.6390 7.6349 7.6144 7.6108 7.6075 7.5999 7.5946 7.5908 7.4531 7.4491 7.4230 7.4154 7.4094 7.4056 7.4012 7.3971 7.3928 7.3747 7.3607 7.3575 7.3532 7.3437 7.3403 7.3363 7.2712 7.2511 7.2258 7.2217 7.2056 7.2015 6.2846 6.2450 6.2303 6.2152 6.1908 6.1758 5.8370 5.8170 4.9529 4.9497 4.9464 4.9360 4.9329 4.9296 4.9264 4.9161 4.9128 4.9096 3.8170 3.7884 3.7738 3.7453 3.3470 3.3270 2.7181 2.6980 2.6807 2.2845 2.2677 2.2470 2.2305 2.2082 2.1900 2.1824 2.1765 2.1579 2.0221 1.6402 1.5435 1.5156 1.4971 1.4788 1.4605 1.4545 1.4423 1.0371 0.9629 0.9446 0.9261
Br
OAc
SI5
O
OTBDPS
4.0544 0.9799 1.9811 0.9575 1.0075 0.9970 0.9909 1.9013 2.9599 2.1411 3.1010
[rel]
[ppm]

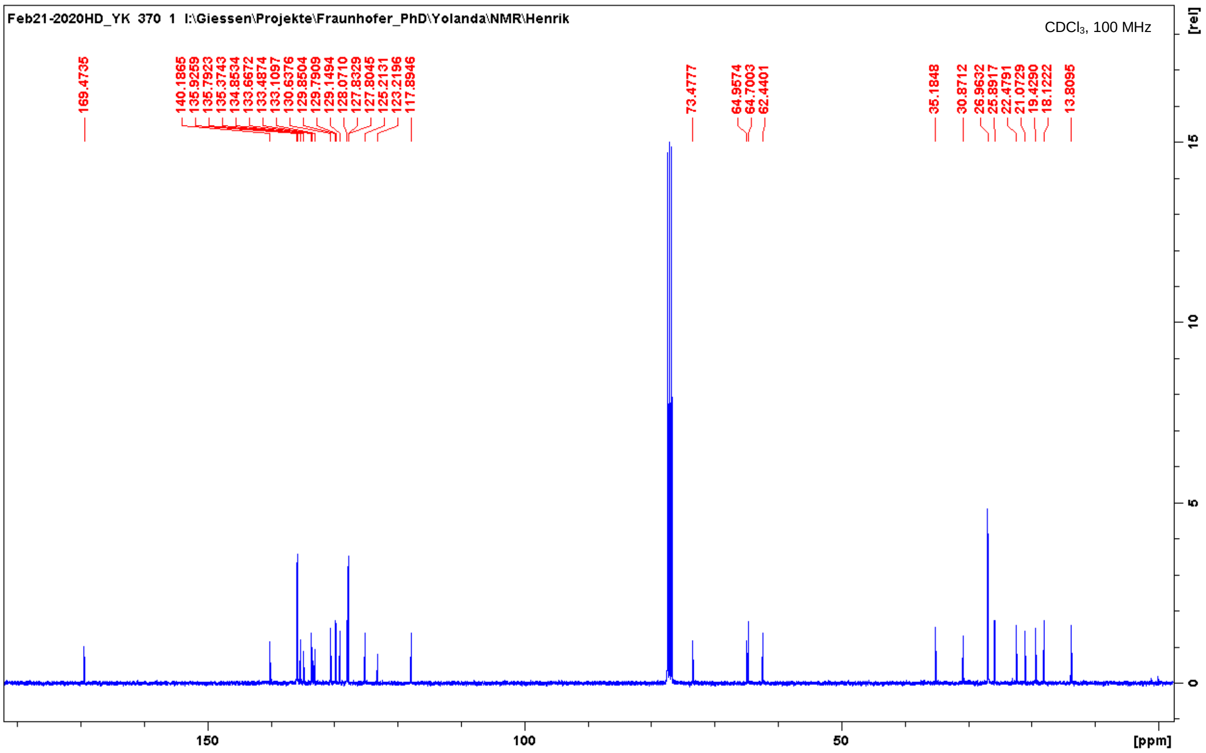
Feb21-2020HD_YK 370 1 I:\Giessen\Projekte\Fraunhofer_PhD\Yolanda\NMR\Henrik
CDCl$_3$, 100 MHz
169.4735
140.1865 135.9259 135.7923 135.3743 134.8534 133.6672 133.4874 133.1097 130.6376 129.8504 129.7909 129.1494 128.0710 127.8329 127.8045 125.2131 123.2196 117.8946
73.4777
64.9574 64.7003 62.4401
35.1848 30.8712 26.9632 25.8917 22.4791 21.0729 19.4290 18.1222 13.8095
[rel]
[ppm]

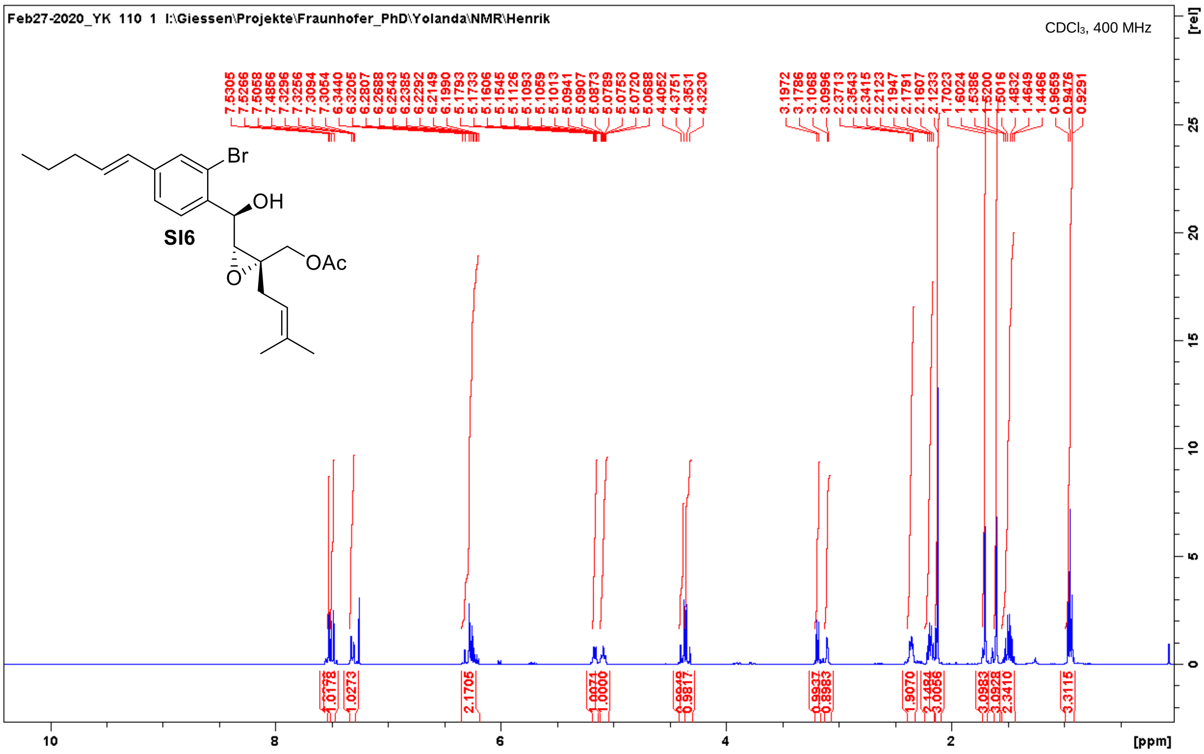
Feb27-2020_YK 110 1 I:\Giessen\Projekte\Fraunhofer_PhD\Yolanda\NMR\Henrik
CDCl3, 400 MHz
[rel]
7.5305 7.5266 7.5058 7.4856 7.3296 7.3256 7.3094 7.3054 6.3440 6.3205 6.2807 6.2688 6.2543 6.2385 6.2292 6.2149 6.1990 5.1793 5.1733 5.1606 5.1545 5.1126 5.1093 5.1059 5.1013 5.0941 5.0907 5.0873 5.0789 5.0753 5.0720 5.0688 4.4052 4.3751 4.3531 4.3230 3.1972 3.1786 3.1068 3.0996 2.3713 2.3543 2.3415 2.2123 2.1947 2.1791 2.1607 2.1233 1.7023 1.6024 1.5386 1.5200 1.5016 1.4832 1.4649 1.4466 0.9659 0.9476 0.9291
Br
OH
SI6
O
OAc
1.0178 1.0273 2.1705 1.0071 1.0000 0.9849 0.9817 0.9937 0.8983 1.9070 2.1484 3.0056 3.0983 3.0928 2.3410 3.3115
10 8 6 4 2 [ppm]

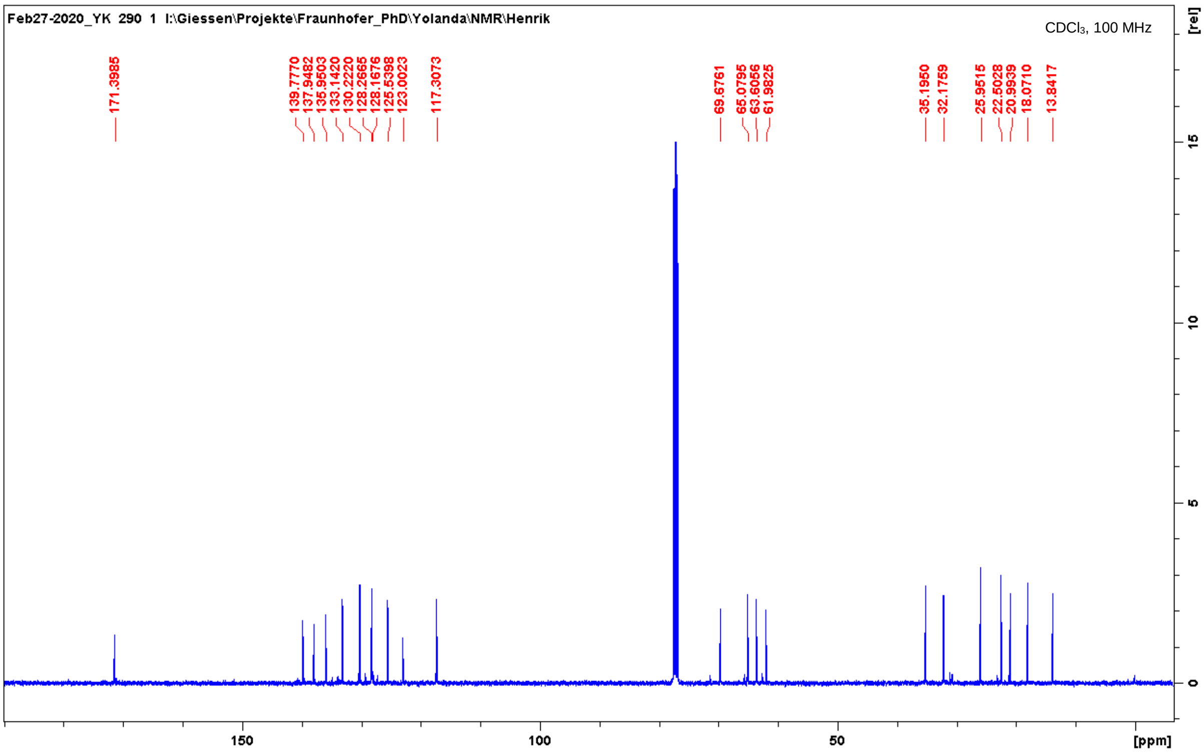
Feb27-2020_YK 290 1 I:\Giessen\Projekte\Fraunhofer_PhD\Yolanda\NMR\Henrik
CDCl3, 100 MHz
[rel]
171.3985 139.7770 137.9482 135.9503 133.1420 130.2220 128.2665 128.1676 125.5398 123.0023 117.3073 69.6761 65.0795 63.6056 61.9825 35.1950 32.1759 25.9515 22.5028 20.9939 18.0710 13.8417
150 100 50 [ppm]

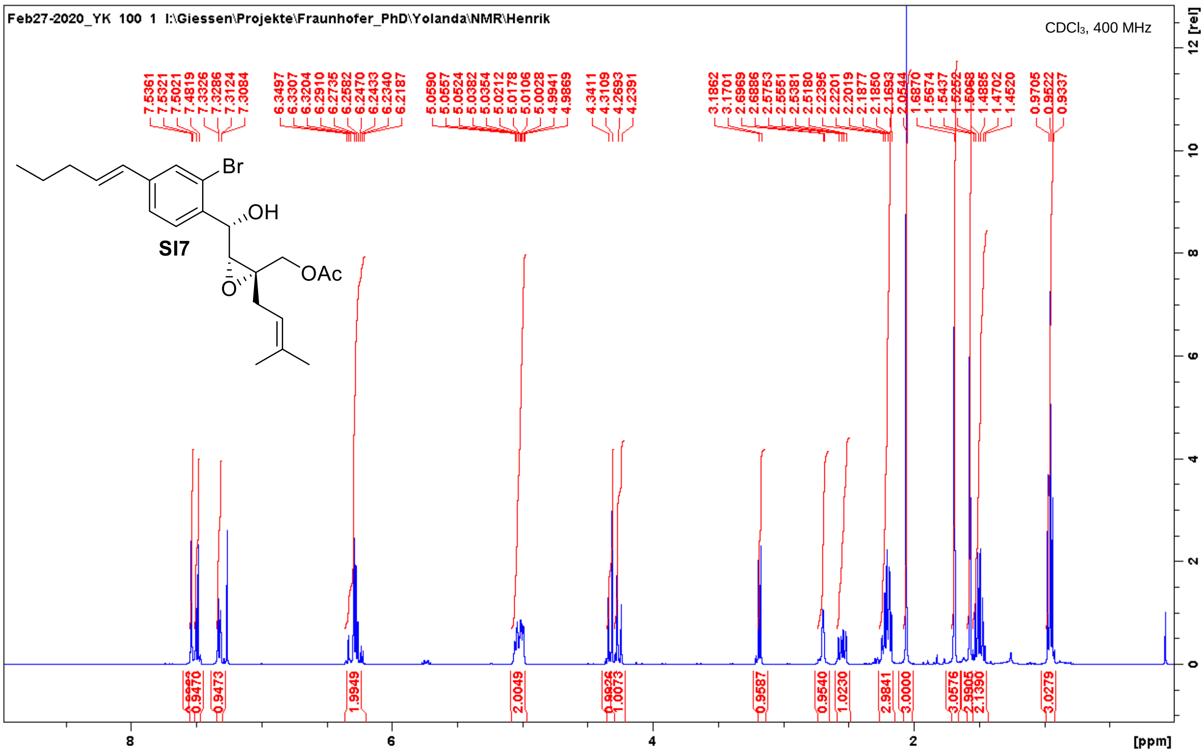
Feb27-2020_YK 100 1 I:\Giessen\Projekte\Fraunhofer_PhD\Yolanda\NMR\Henrik
CDCl3, 400 MHz
Br
OH
SI7
OAc
O
[ppm]
[rel]

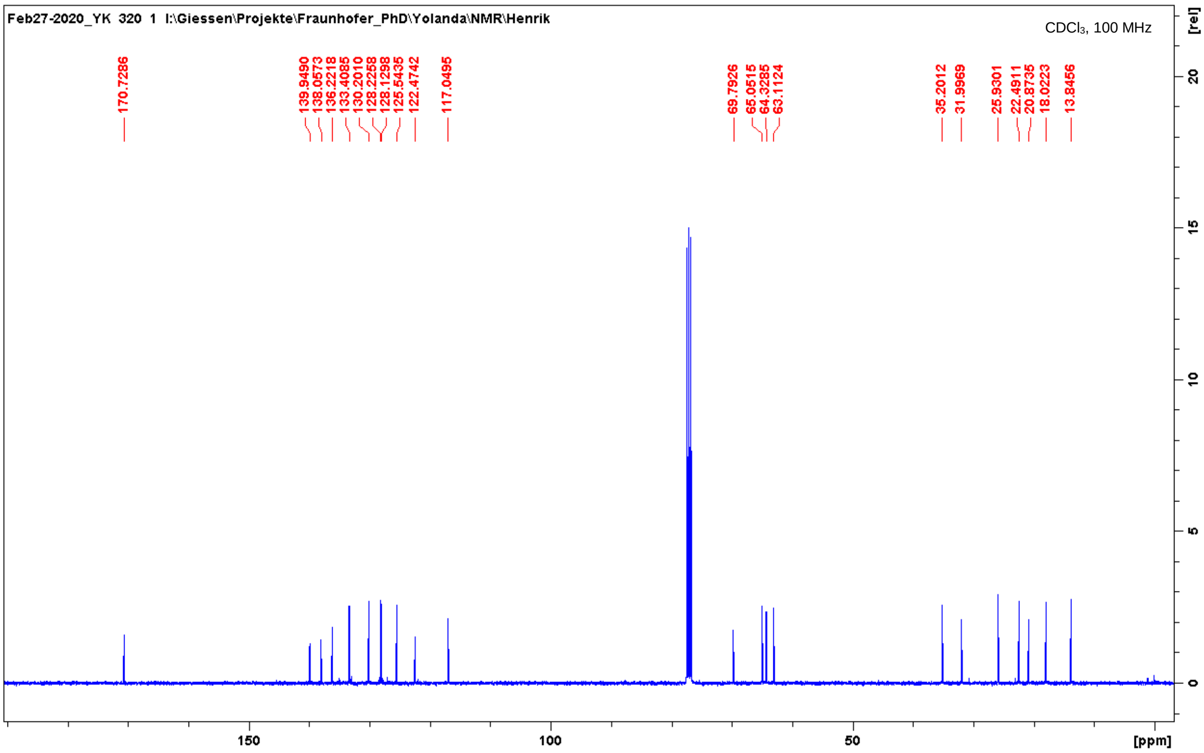
Feb27-2020_YK 320 1 I:\Giessen\Projekte\Fraunhofer_PhD\Yolanda\NMR\Henrik
CDCl3, 100 MHz
170.7286
139.9490
138.0573
136.2218
133.4085
130.2010
128.2258
128.1298
125.5435
122.4742
117.0495
69.7926
65.0515
64.3285
63.1124
35.2012
31.9969
25.9301
22.4911
20.8735
18.0223
13.8456
[ppm]
[rel]

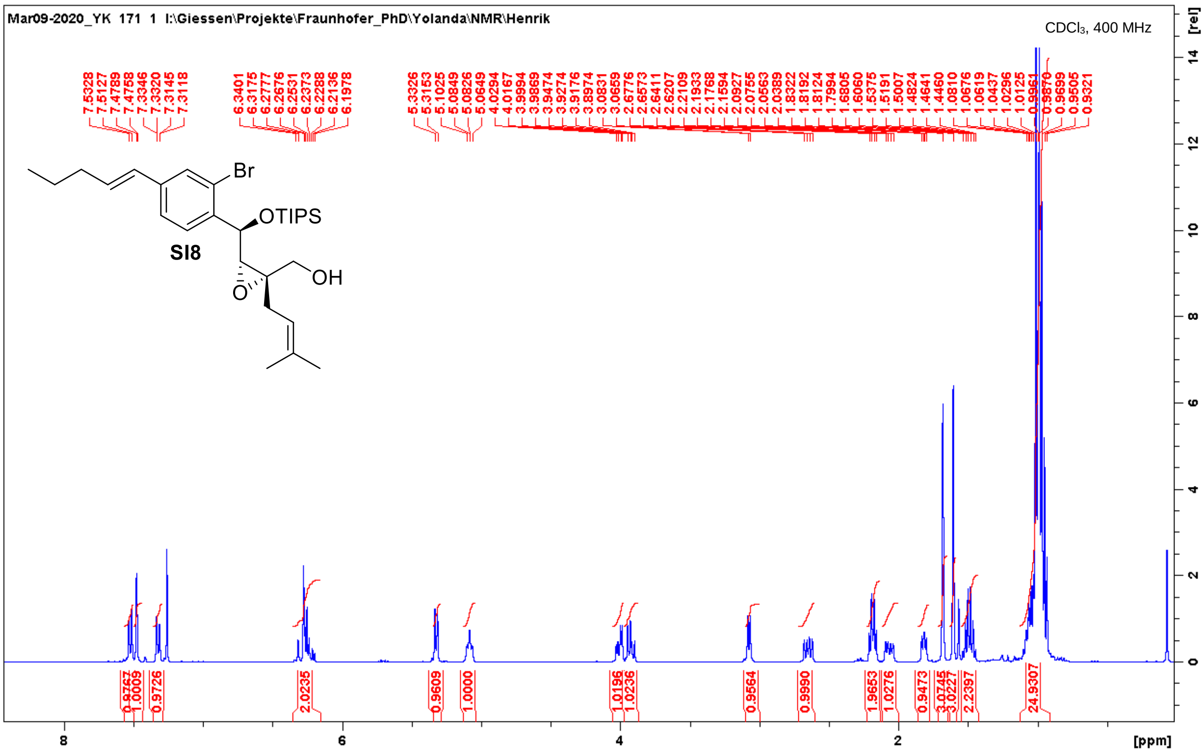
Mar09-2020_YK 171 1 I:\Giessen\Projekte\Fraunhofer_PhD\Yolanda\NMR\Henrik
CDCl3, 400 MHz
SI8
Br
OTIPS
OH
O
[ppm]
[rel]

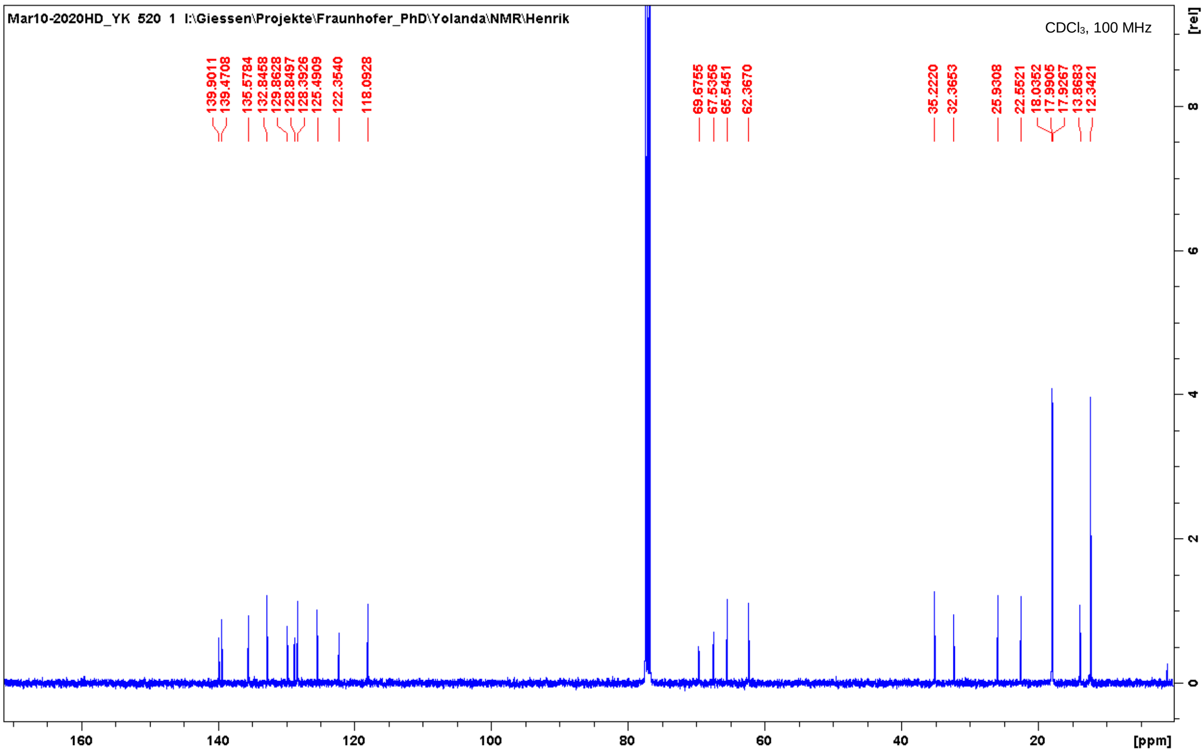
Mar10-2020HD_YK 520 1 I:\Giessen\Projekte\Fraunhofer_PhD\Yolanda\NMR\Henrik
CDCl3, 100 MHz
139.9011
139.4708
135.5784
132.8458
129.8628
128.8497
128.3926
125.4909
122.3540
118.0928
69.6755
67.5356
65.5451
62.3670
35.2220
32.3653
25.9308
22.5521
18.0352
17.9905
17.9267
13.8683
12.3421
[ppm]
[rel]

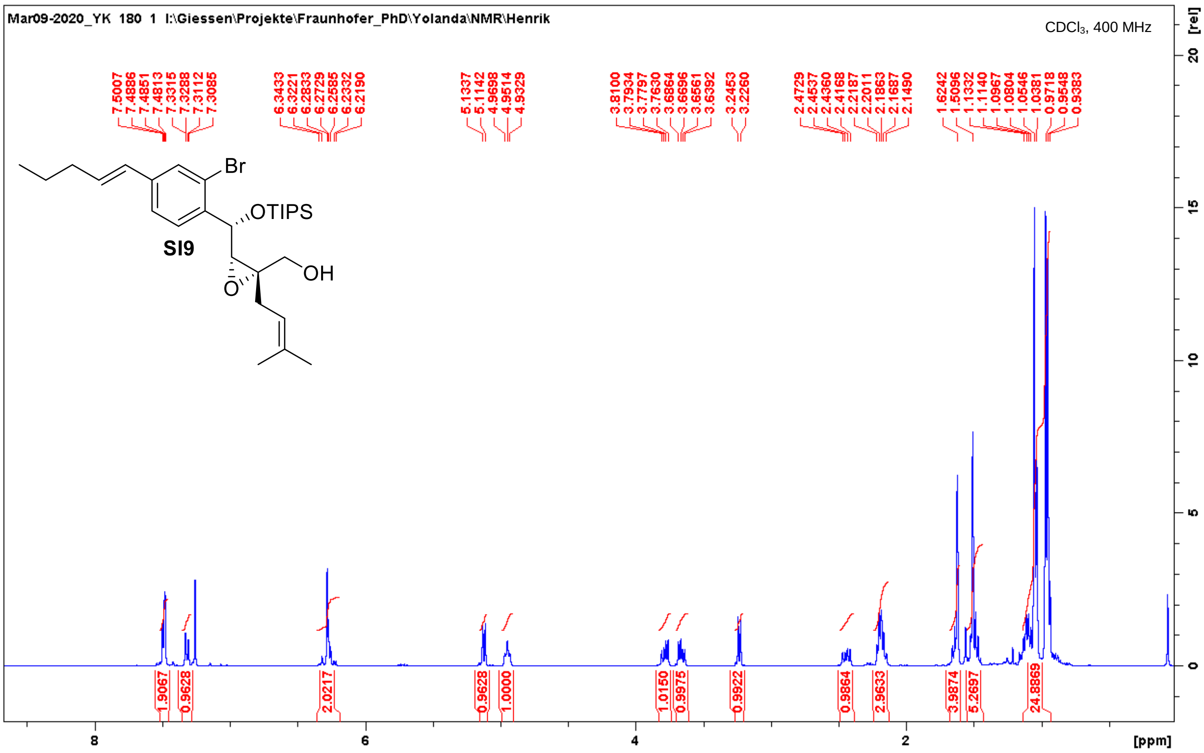
Mar09-2020_YK 180 1 I:\Giessen\Projekte\Fraunhofer_PhD\Yolanda\NMR\Henrik
CDCl3, 400 MHz
Br
OTIPS
SI9
O
OH
[rel]
[ppm]

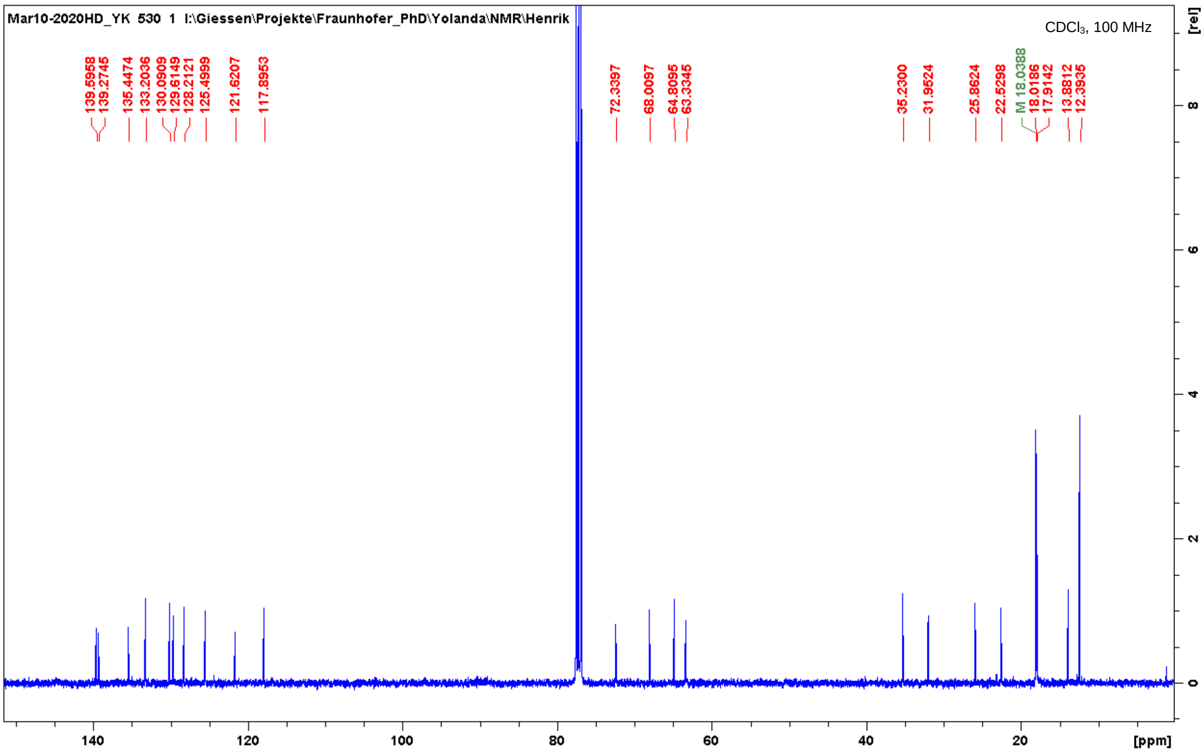
Mar10-2020HD_YK 530 1 I:\Giessen\Projekte\Fraunhofer_PhD\Yolanda\NMR\Henrik
CDCl3, 100 MHz
[rel]
[ppm]

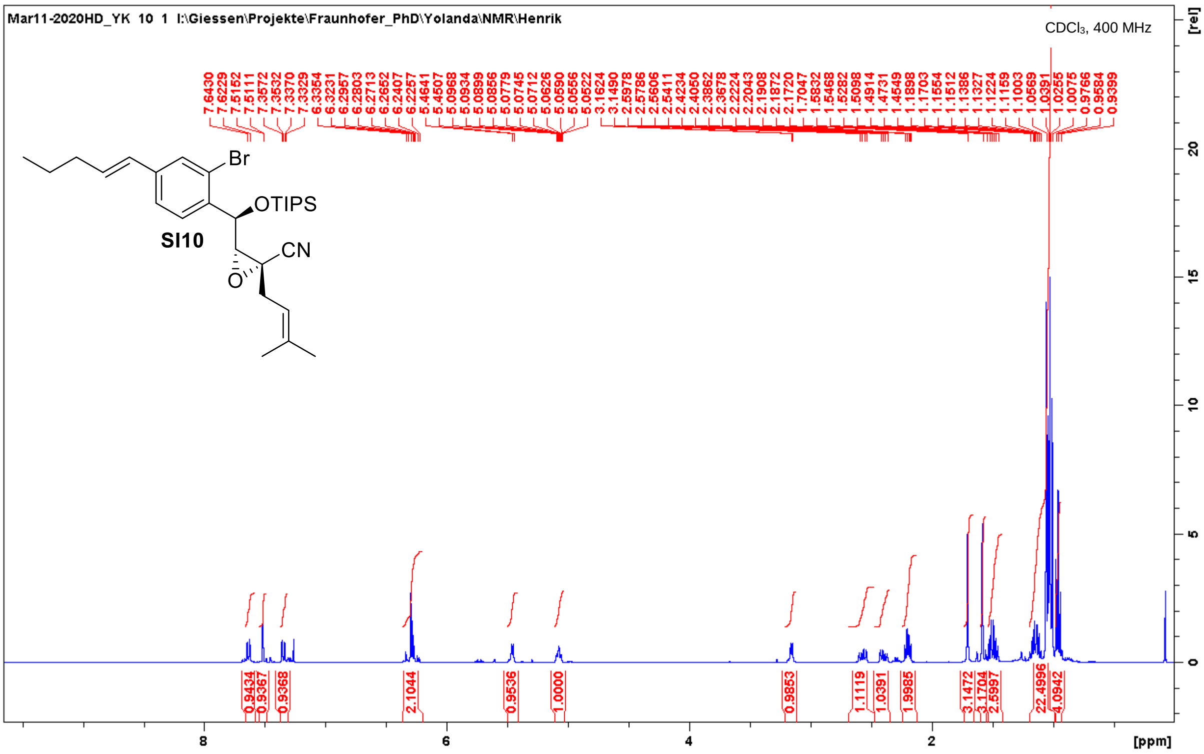
Mar11-2020HD_YK 10 1 I:\Giessen\Projekte\Fraunhofer_PhD\Yolanda\NMR\Henrik
CDCl$_3$, 400 MHz
[rel]
7.6430 7.6229 7.5152 7.5111 7.3572 7.3532 7.3370 7.3329 6.3354 6.3231 6.2957 6.2803 6.2713 6.2652 6.2407 6.2257 5.4641 5.4507 5.0968 5.0934 5.0899 5.0856 5.0779 5.0745 5.0712 5.0626 5.0590 5.0556 5.0522 3.1624 3.1490 2.5978 2.5786 2.5606 2.5411 2.4234 2.4050 2.3862 2.3678 2.2224 2.2043 2.1908 2.1872 2.1720 1.7047 1.5832 1.5468 1.5282 1.5098 1.4914 1.4731 1.4549 1.1898 1.1703 1.1554 1.1512 1.1386 1.1327 1.1224 1.1159 1.1003 1.0569 1.0391 1.0255 1.0076 0.9766 0.9584 0.9399
Br
OTIPS
CN
O
SI10
0.9434 0.9367 0.9368 2.1044 0.9536 1.0000 0.9853 1.1119 1.0391 1.9985 3.1472 3.1704 2.5997 22.4996 4.0942
8
6
4
2
[ppm]
0
5
10
15
20

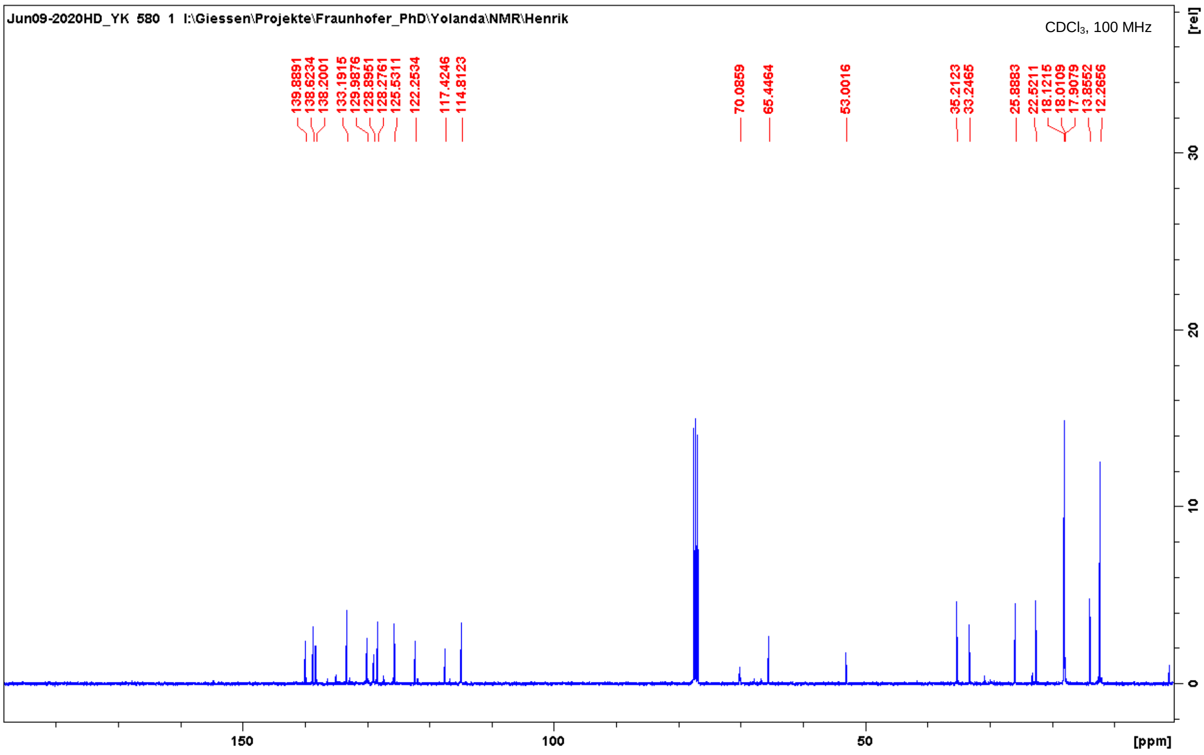
Jun09-2020HD_YK 580 1 I:\Giessen\Projekte\Fraunhofer_PhD\Yolanda\NMR\Henrik
CDCl$_3$, 100 MHz
[rel]
139.8891 138.6234 138.2001 133.1915 129.9876 128.8951 128.2761 125.5311 122.2534 117.4246 114.8123 70.0859 65.4464 53.0016 35.2123 33.2465 25.8883 22.5211 18.1215 18.0109 17.9079 13.8552 12.2656
150
100
50
[ppm]
0
10
20
30

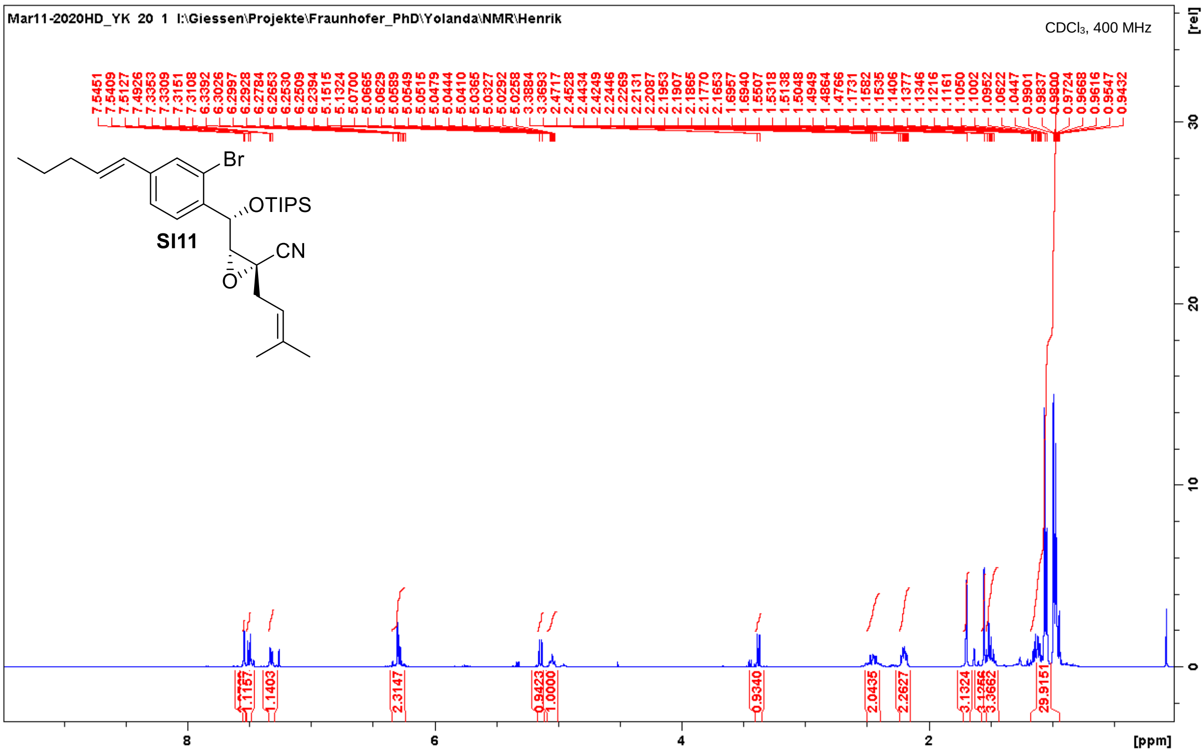
Mar11-2020HD_YK 20 1 I:\Giessen\Projekte\Fraunhofer_PhD\Yolanda\NMR\Henrik
CDCl3, 400 MHz
7.5451 7.5409 7.5127 7.4926 7.3353 7.3309 7.3151 7.3108 6.3392 6.3026 6.2997 6.2928 6.2784 6.2653 6.2530 6.2509 6.2394 5.1515 5.1324 5.0700 5.0665 5.0629 5.0589 5.0549 5.0515 5.0479 5.0444 5.0410 5.0365 5.0327 5.0292 5.0258 3.3884 3.3693 2.4717 2.4528 2.4434 2.4249 2.2446 2.2269 2.2131 2.2087 2.1953 2.1907 2.1865 2.1770 2.1653 1.6957 1.6940 1.5507 1.5318 1.5138 1.5048 1.4949 1.4864 1.4766 1.1731 1.1582 1.1535 1.1406 1.1377 1.1346 1.1216 1.1161 1.1050 1.1002 1.0952 1.0622 1.0447 0.9901 0.9837 0.9800 0.9724 0.9668 0.9616 0.9547 0.9432
Br
OTIPS
CN
O
SI11
1.1157 1.1403 2.3147 0.9423 1.0000 0.9340 2.0435 2.2627 3.1324 3.1256 3.3662 29.9151
[rel]
[ppm]

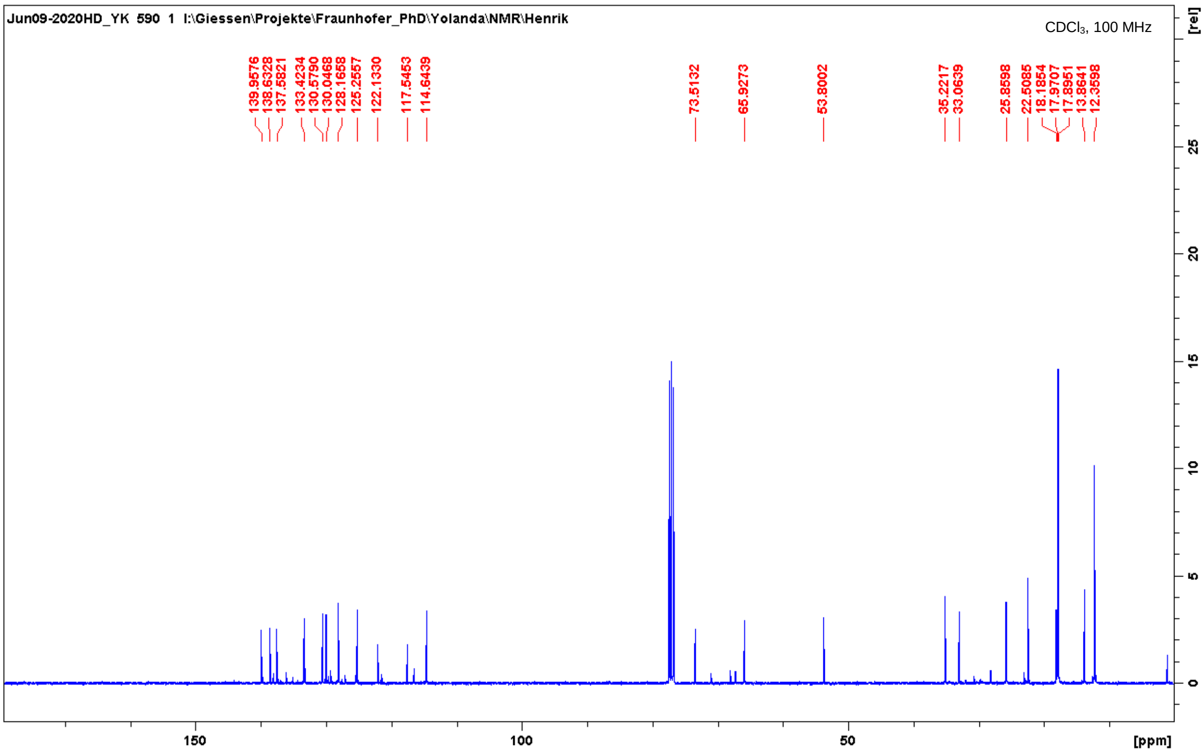
Jun09-2020HD_YK 590 1 I:\Giessen\Projekte\Fraunhofer_PhD\Yolanda\NMR\Henrik
CDCl3, 100 MHz
139.9576 138.6328 137.5821 133.4234 130.5790 130.0468 128.1658 125.2557 122.1330 117.5453 114.6439 73.5132 65.9273 53.8002 35.2217 33.0639 25.8598 22.5085 18.1854 17.9707 17.8951 13.8641 12.3598
[rel]
[ppm]

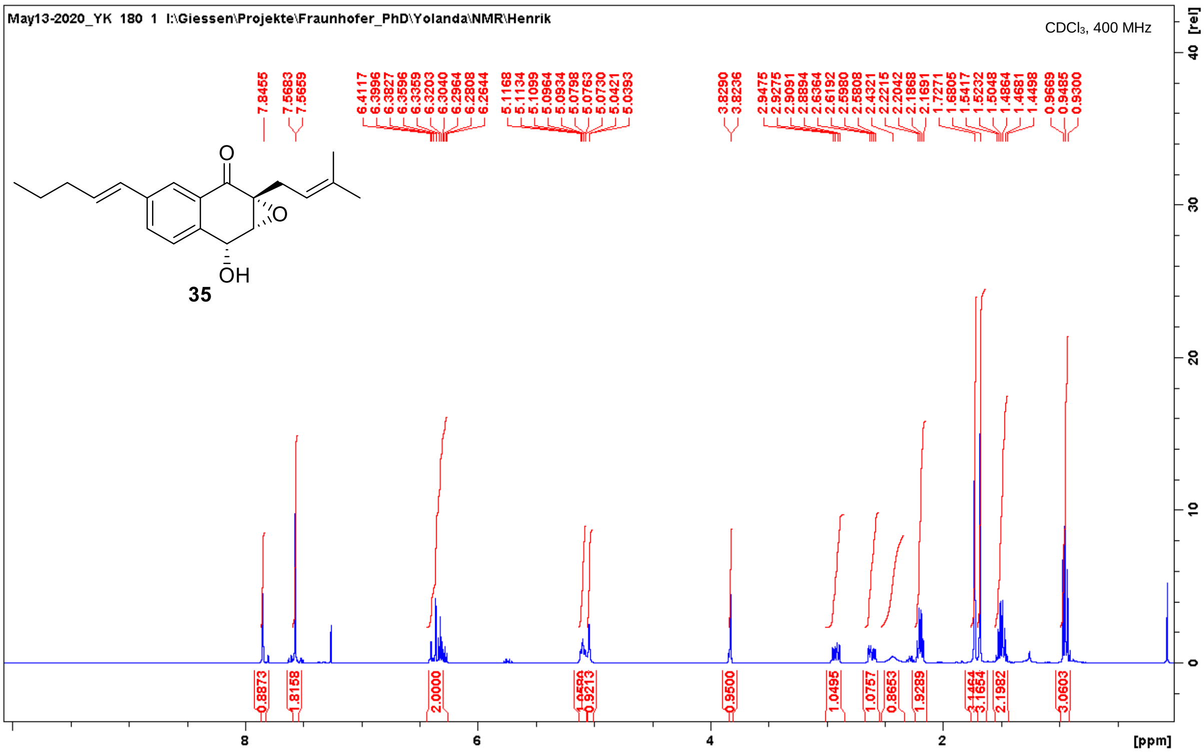
May13-2020_YK 180 1 I:\Giessen\Projekte\Fraunhofer_PhD\Yolanda\NMR\Henrik
CDCl3, 400 MHz
35
[ppm]
[rel]

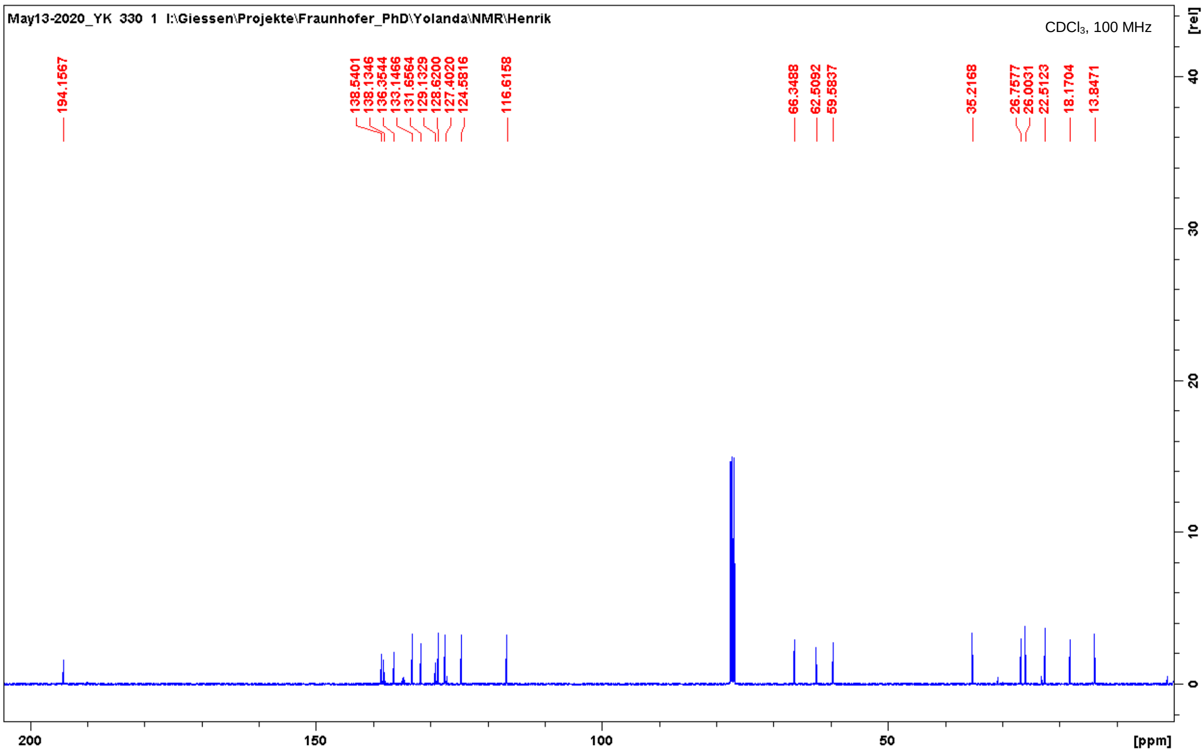
May13-2020_YK 330 1 I:\Giessen\Projekte\Fraunhofer_PhD\Yolanda\NMR\Henrik
CDCl3, 100 MHz
194.1567
138.5401
138.1346
136.3544
133.1466
131.6564
129.1329
128.6200
127.4020
124.5816
116.6158
66.3488
62.5092
59.5837
35.2168
26.7577
26.0031
22.5123
18.1704
13.8471
[ppm]
[rel]

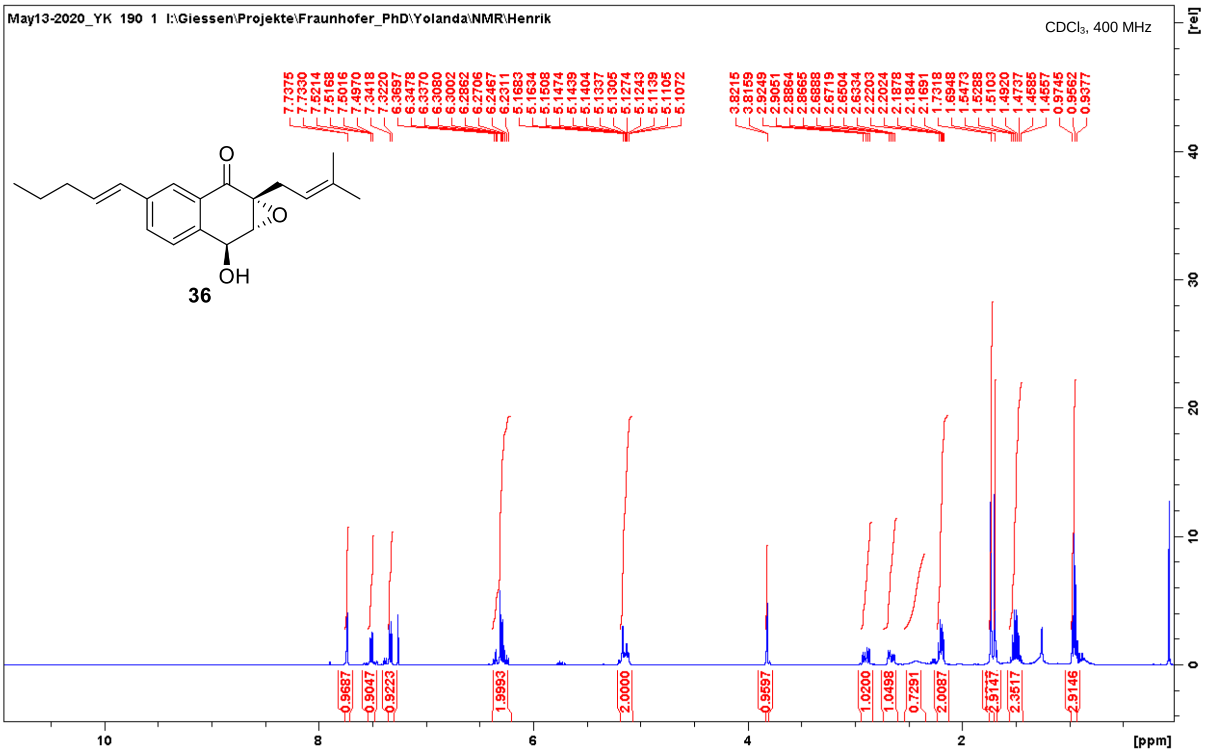
May13-2020_YK 190 1 I:\Giessen\Projekte\Fraunhofer_PhD\Yolanda\NMR\Henrik
CDCl3, 400 MHz
36
OH
O
[rel]
[ppm]

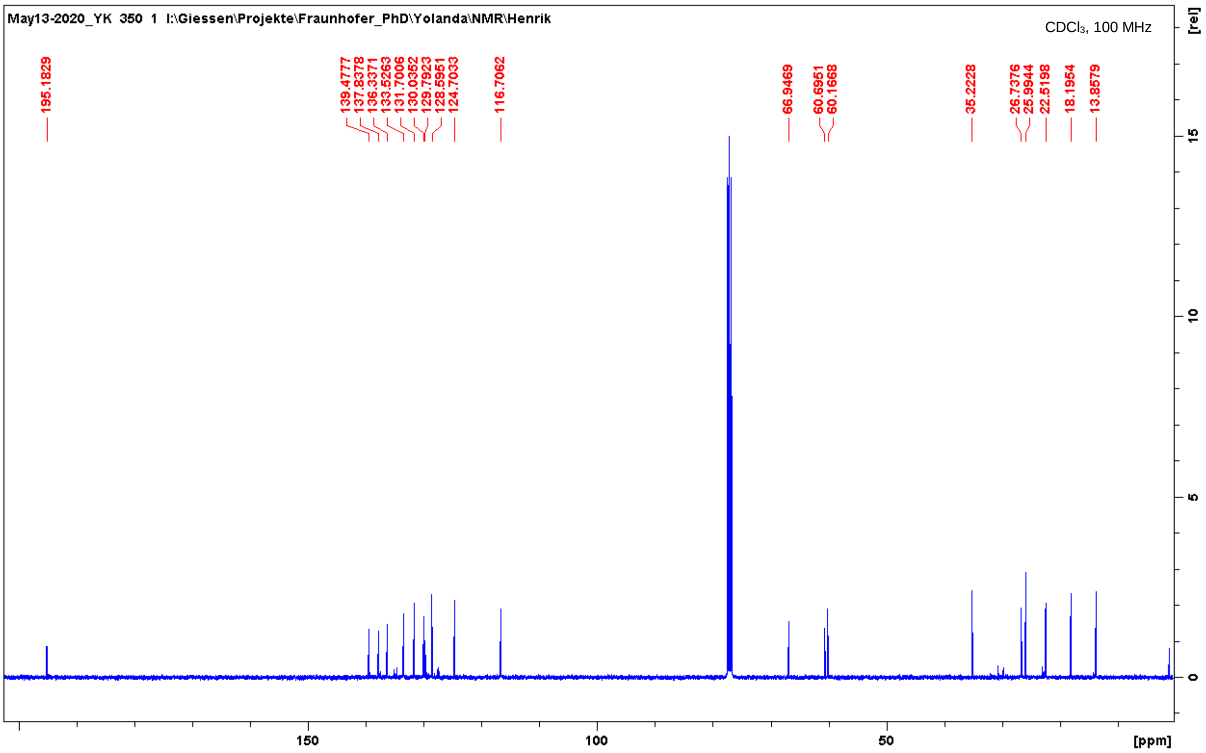
May13-2020_YK 350 1 I:\Giessen\Projekte\Fraunhofer_PhD\Yolanda\NMR\Henrik
CDCl3, 100 MHz
195.1829
139.4777
137.8378
136.3371
133.5263
131.7006
130.0352
129.7923
128.5951
124.7033
116.7062
66.9469
60.6951
60.1668
35.2228
26.7376
25.9944
22.5198
18.1954
13.8579
[rel]
[ppm]

6. Determination of the enantiomeric excesses (*ee*)

Operator:ge1585 Timebase:HPLC_1 Sequence:Y.KLEINER

Page 1-1
19.5.2020 1:30 PM

84 Y.KLEINER YK0352_S

CHIRALPAK IC | 1ml/min. | 85% Hexan, 15% IPA

Sample Name:	**Y.KLEINER YK0352_S**	*Injection Volume:*	**5,0**
Vial Number:	**85**	*Channel:*	**UV_VIS_2**
Sample Type:	**unknown**	*Wavelength:*	**270**
Control Program:	**Analyse03_F10_A85_B00_C15**	*Bandwidth:*	**1**
Quantif. Method:	**default**	*Dilution Factor:*	**1,0000**
Recording Time:	**19.5.2020 12:44**	*Sample Weight:*	**1,0000**
Run Time (min):	**20,00**	*Sample Amount:*	**1,0000**

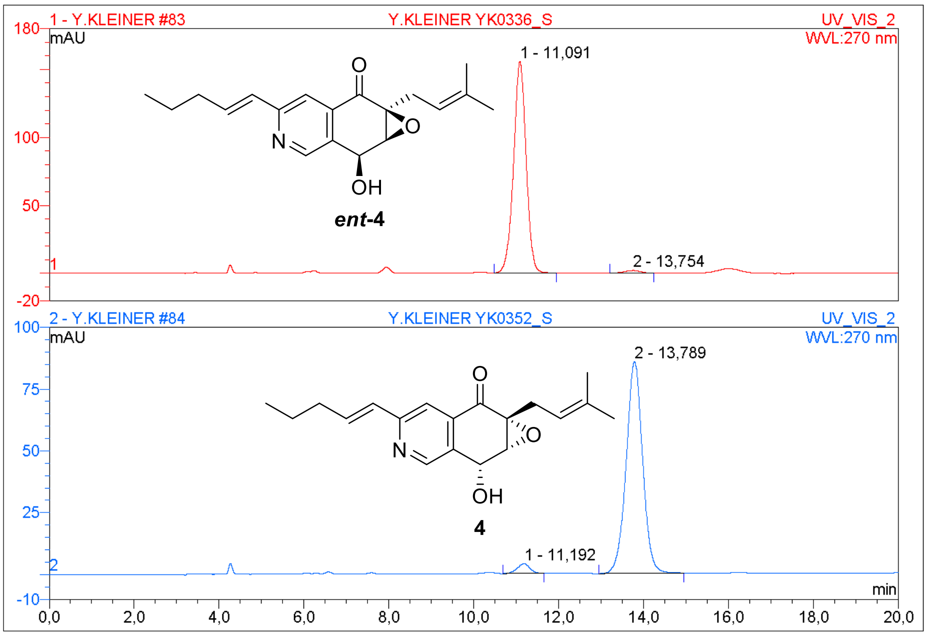

No.	Ret.Time min	Peak Name	Height mAU	Area mAU*min	Rel.Area %	Amount	Type
1	11,19	n.a.	3,865	1,334	3,35	n.a.	BMB
2	13,79	n.a.	85,720	38,537	96,65	n.a.	BMB
**Total:**			89,585	39,872	100,00	0,000	

default/Integration

Chromeleon (c) Dionex 1996-2001
Version 6.80 SR15 Build 4656 (243203)

83 Y.KLEINER YK0336_S

CHIRALPAK IC | 1ml/min. | 85% Hexan, 15% IPA

Sample Name:	**Y.KLEINER YK0336_S**	*Injection Volume:*	**5,0**
Vial Number:	**84**	*Channel:*	**UV_VIS_2**
Sample Type:	**unknown**	*Wavelength:*	**270**
Control Program:	**Analyse03_F10_A85_B00_C15**	*Bandwidth:*	**1**
Quantif. Method:	**default**	*Dilution Factor:*	**1,0000**
Recording Time:	**19.5.2020 12:17**	*Sample Weight:*	**1,0000**
Run Time (min):	**20,00**	*Sample Amount:*	**1,0000**

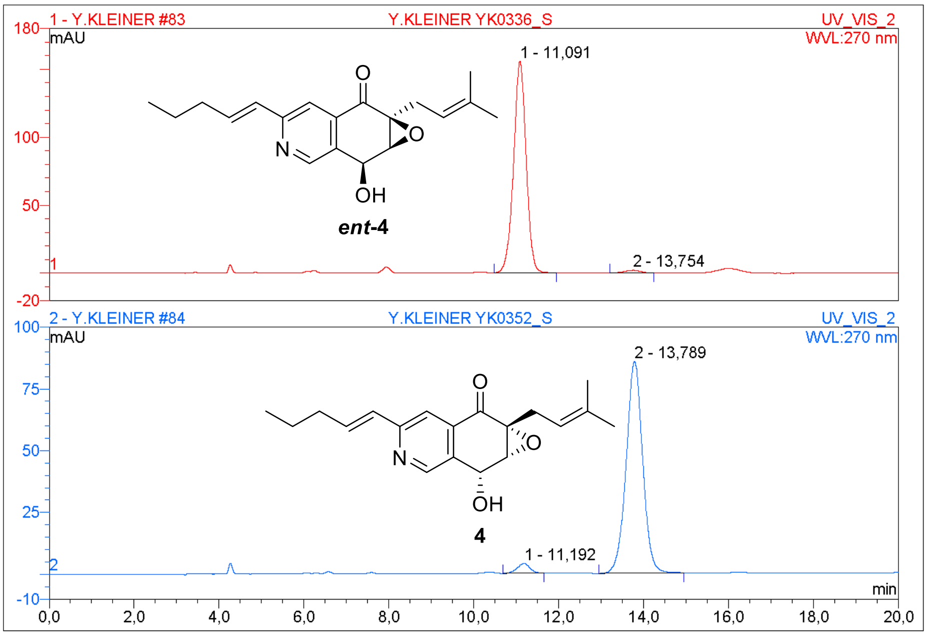

No.	Ret.Time min	Peak Name	Height mAU	Area mAU*min	Rel.Area %	Amount	Type
1	11,09	n.a.	155,502	53,258	98,54	n.a.	BMB
2	13,75	n.a.	1,900	0,787	1,46	n.a.	BMB
**Total:**			157,402	54,045	100,00	0,000	

default/Integration

Chromeleon (c) Dionex 1996-2001
Version 6.80 SR15 Build 4656 (243203)

80 Y.KLEINER YK0323_S

CHIRALPAK IC | 1ml/min. | 85% Hexan, 15% IPA

Sample Name:	**Y.KLEINER YK0323_S**	*Injection Volume:*	**2,5**
Vial Number:	**81**	*Channel:*	**UV_VIS_2**
Sample Type:	**unknown**	*Wavelength:*	**270**
Control Program:	**Analyse03_F10_A85_B00_C15**	*Bandwidth:*	**1**
Quantif. Method:	**default**	*Dilution Factor:*	**1,0000**
Recording Time:	**18.5.2020 9:40**	*Sample Weight:*	**1,0000**
Run Time (min):	**20,00**	*Sample Amount:*	**1,0000**

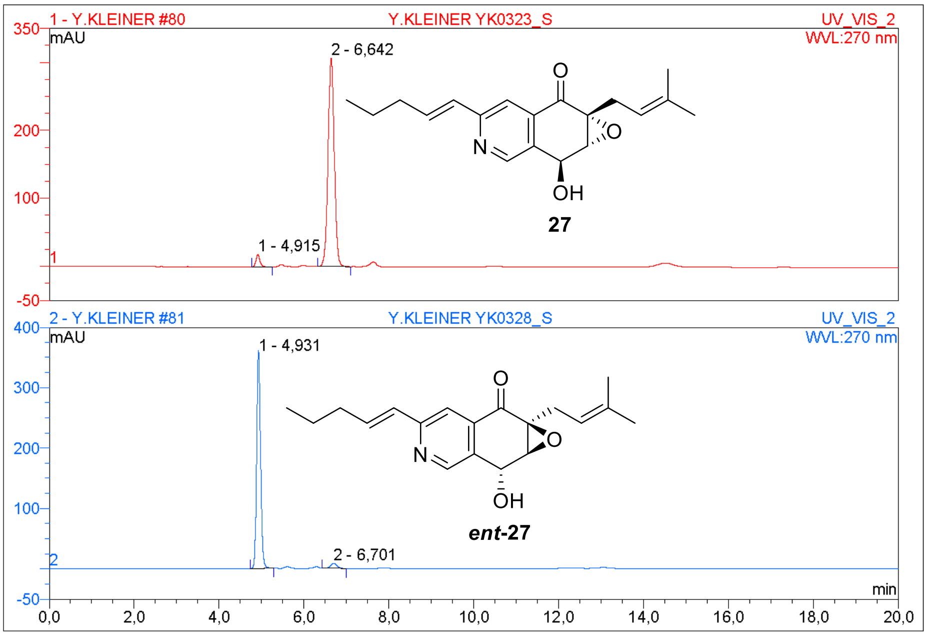

No.	Ret.Time min	Peak Name	Height mAU	Area mAU*min	Rel.Area %	Amount	Type
1	4,92	n.a.	18,151	1,921	3,55	n.a.	BMB
2	6,64	n.a.	306,543	52,173	96,45	n.a.	BMB
**Total:**			324,694	54,095	100,00	0,000	

81 Y.KLEINER YK0328_S

CHIRALPAK IC | 1ml/min. | 85% Hexan, 15% IPA

Sample Name:	**Y.KLEINER YK0328_S**	*Injection Volume:*	**2,5**
Vial Number:	**82**	*Channel:*	**UV_VIS_2**
Sample Type:	**unknown**	*Wavelength:*	**270**
Control Program:	**Analyse03_F10_A85_B00_C15**	*Bandwidth:*	**1**
Quantif. Method:	**default**	*Dilution Factor:*	**1,0000**
Recording Time:	**18.5.2020 10:26**	*Sample Weight:*	**1,0000**
Run Time (min):	**20,00**	*Sample Amount:*	**1,0000**

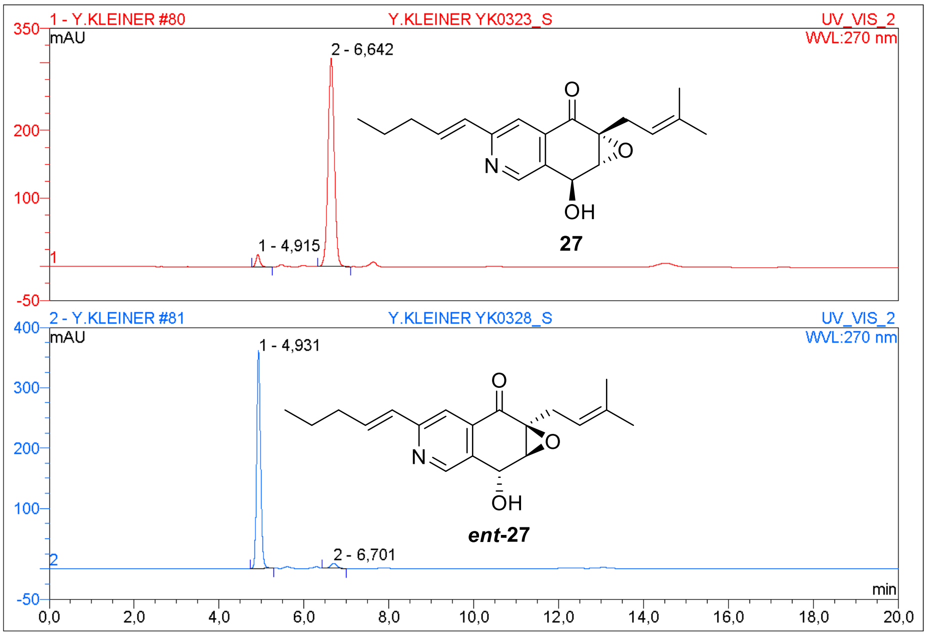

No.	Ret.Time min	Peak Name	Height mAU	Area mAU*min	Rel.Area %	Amount	Type
1	4,93	n.a.	360,673	38,359	96,46	n.a.	BMB
2	6,70	n.a.	8,238	1,409	3,54	n.a.	BMB
**Total:**			368,912	39,768	100,00	0,000	
